# Tumor proliferation associates with greater sensitivity to androgen receptor pathway inhibition in metastatic prostate cancer

**DOI:** 10.1172/JCI203201

**Published:** 2026-03-31

**Authors:** Larissa Mendes, Peter F. Dutey-Magni, Emily Grist, Ashwin Sachdeva, Sara Santos Vidal, Sharanpreet Lall, Marina A. Parry, Claire L. Amos, Nafisah Atako, Anna Wingate, Daniel Wetterskog, Matthew R. Sydes, Chris C. Parker, Noel Clarke, Christopher J. Sweeney, Mahesh K. Parmar, Louise C. Brown, Nicholas D. James, Daniel M. Berney, Gerhardt Attard

**Affiliations:** 1 University College London, London, United Kingdom.; 2 The University of Manchester, Manchester, United Kingdom.; 3 Royal Marsden Hospital and The Institute of Cancer Research, London, United Kingdom.; 4 University of South Australia, Adelaide, Australia.; 5 Queen Mary University London, London, United Kingdom.

**Keywords:** Clinical Research, Oncology, Biomarkers, Clinical trials, Prostate cancer

## Abstract

<article data-scroll-anchor=”false” data-testid=”conversation-turn-47” data-turn=”user” data-turn-id=”be25b520-cadc-4509-8230-4988b5406e4d” dir=”auto” tabindex=”-1”> Prostate cancers with high proliferation rates have shorter survival times but when spread has occurred (metastatic), there is increased sensitivity to hormone therapy with abiraterone. </article>

**To the Editor:** Proliferation is a hallmark of cancer (1). Androgens increase proliferation of prostate cancer cells in vitro and inhibition of androgens markedly reduces proliferation. Observational cohorts have suggested that increased proliferation associates with a poor response to hormone therapy, including the androgen receptor pathway inhibitors (ARPI) abiraterone or enzalutamide (2). To better understand the relationship between tumor proliferation and the effect of ARPI when added to androgen deprivation therapy (ADT), we analyzed prostate cancer biopsies from patients on ADT randomized to abiraterone in the STAMPEDE platform.

Between November 2011 and January 2014, 914 nonmetastatic and 1,003 metastatic patients enrolled into the STAMPEDE abiraterone trial (3). To enhance statistical power in nonmetastatic disease given a lower event rate, 1,060 nonmetastatic patients enrolled between July 2014 and March 2016 in the abiraterone and enzalutamide trial were combined in this analysis, similar to the primary report (4). Of the 2,977 patients, 2,963 were recruited in the United Kingdom (115 centers) with consent for tissue collection from 2,912. Ki-67 was scored for 1,605 cases (Figure 1A and Supplemental Figure 1A; supplemental material available online with this article; https://doi.org/10.1172/JCI203201DS1). Tumors biopsied after ADT were excluded in a sensitivity analysis restricted to 1,413 ADT-naive cancers (88%).

Baseline characteristics for the analytical cohort were consistent with the full trial cohorts reported previously (3, 4) (Supplemental Table 1). By February 2024, 34% (*n* = 364) of the nonmetastatic and 75% (*n* = 397) of the metastatic cohort had died.

Ki-67 was higher in the presence of lymph node involvement in nonmetastatic disease (*F*_1,1074_=28, *P* < 0.001) but there was no difference between low- and high-volume metastatic disease (*F*_1,510_=0.45, *P* = 0.504, Figure 1B). Ki-67 was positively associated with Gleason score (*F*_7,1593_=9, *P* < 0.001, Figure 1C) and tumor stage (*F*_5,1599_=4, *P* < 0.001 Figure 1D), but not pre-ADT serum PSA (Spearman’s ρ = –0.02, Supplemental Figure 1C). ADT-exposed tumors had lower Ki-67 scores (Supplemental Figure 1D).

Ki-67 score was linearly associated with shorter survival. In nonmetastatic disease, adjusted for baseline characteristics, a 10-percentage point increment in Ki-67 was associated with a 23% increase in hazards of death (95% CI: 12%–37%; *P* < 0.001) with ADT and 25% (95% CI: 8%–45%; *P* = 0.004) with ADT and abiraterone (Figure 1E). In metastatic disease, every 10-percentage point increased hazards of death by 31% (95% CI: 19%–44%; *P* < 0.001) with ADT, but only 6% (95% CI: −2%–16%, *P* = 0.172) with ADT and abiraterone (Figure 1F and Supplemental Table 2).

Given this strong attenuation, we identified that abiraterone effectiveness was significantly greater in metastatic cancers with higher Ki-67 scores (interaction *P* < 0.001, Figure 1G, Supplemental Table 3).

In contrast, and despite a similar number of events, we did not identify treatment effect heterogeneity in nonmetastatic patients (HR=0.97; 95% CI: 0.85–1.11; Figure 1H). This interaction was unchanged when using metastasis progression-free survival as the endpoint or when excluding patients biopsied after ADT (Supplemental Table 3).

The Statistical Analysis Plan prespecified dichotomizing Ki-67 around the median value in metastatic patients (<15%, ≥15%, Figure 1, I and J). There was attenuation of the interaction effect with dichotomization, suggesting that linear modelling may better capture the biological gradient of response.

Our study has some limitations. Tumor collection started after completion of accrual, but the large patient numbers reduce the risk of confounding factors from retrieval. Tumors could have been biopsied after treatment started, affecting Ki-67 expression: sensitivity analyses excluding these cases confirmed the same clinical associations. Scores in our study were independently reviewed by uropathologists using a standardized validated scoring methodology (5), but may not fully capture intratumoral heterogeneity. This could be addressed in future work that could also incorporate additional morphological information.

In conclusion, highly proliferating tumors are more aggressive and more sensitive to ARPI. In contrast, our recent study on an overlapping set of patients identified no interaction of proliferation with docetaxel sensitivity (6). While all patients with metastatic disease derived a meaningful survival benefit from abiraterone, the greater benefit in highly proliferative disease identifies a potential cancer vulnerability that could be exploited to further increase the anticancer effect of ARPI.

## Conflict of interest

Conflicts of interest are included in the Supplemental Data.

## Funding support

- Cancer Research UK’s Clinical Research Committee (formerly the Clinical Trials Advisory Awards Committee, CRUK_A12459 to NDJ; funded the STAMPEDE trial).
- Novartis, Sanofi-Aventis, Pfizer, Janssen Pharma NV, Astellas, and Clovis Oncology provided educational grants.
- Janssen and Astellas provided free drugs for the conduct of the trials included in this study.
- The MRC Clinical Trials Unit at UCL received core funding from the Medical Research Council (grant codes MC_UU_12023/25 and MC_UU_00004/01 to MKP).
- A precision award from Prostate Cancer UK (MA-PM16-001, GA).
- The Prostate Cancer Foundation (Challenge Award: 2019CHAL2729, GA and JBCF Young Investigator Award, EG).
- The John Black Charitable Foundation (GA).
- Cancer Research UK (A22744, GA).
- A Cancer Research UK-UCL Centre Award/Clinical Training Award (A27436, EG).
- The Orchid Foundation (BTXG1A1S, D.M.B.).
- The UK National Institute for Health Research funding to the UCL Hospital’s Biomedical Research Centre (GA).
- Cancer Research UK funding to the City of London Centre (GA).

## Supplementary Material

Supplemental data

Supporting data values

## Figures and Tables

**Figure 1:**
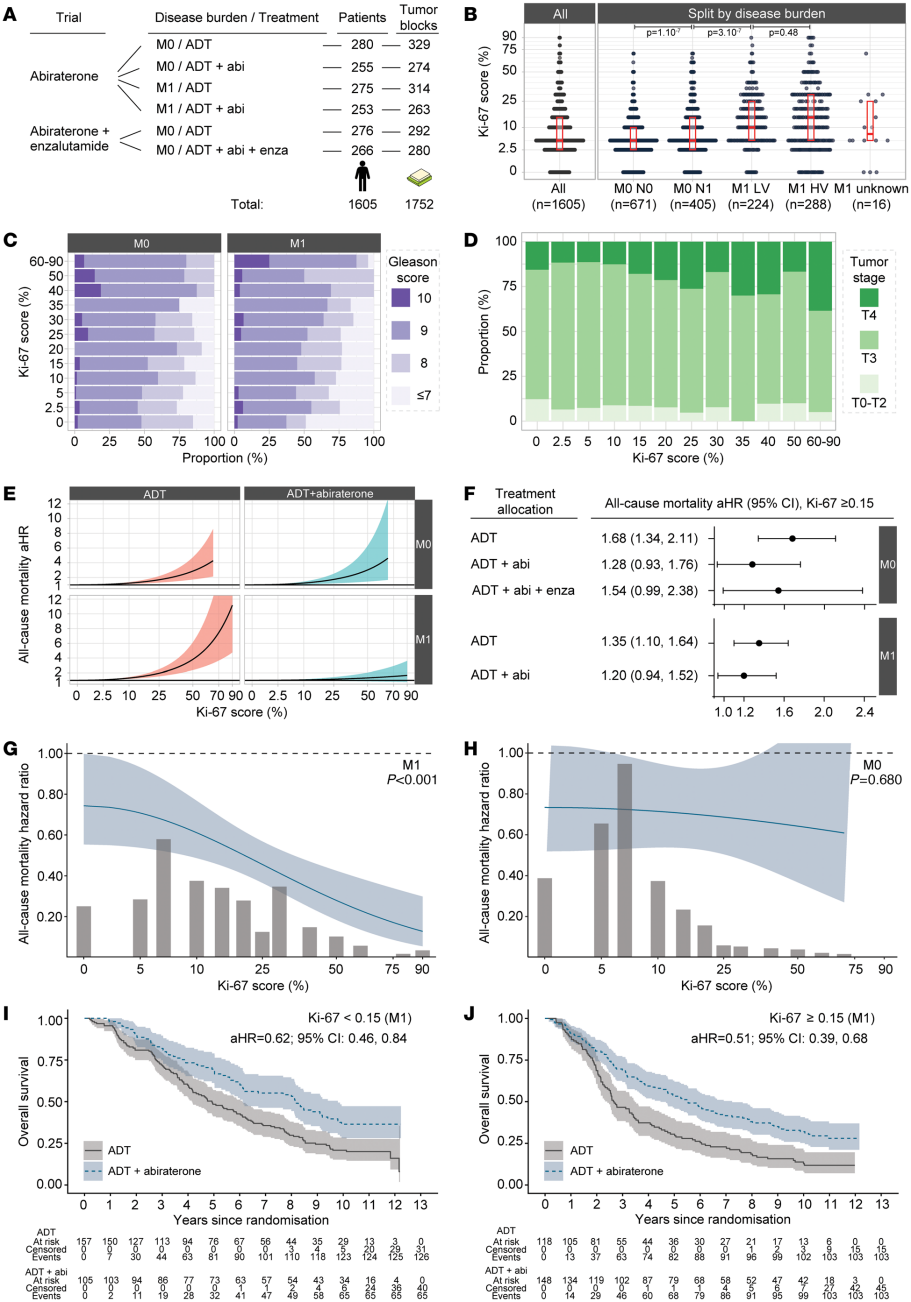
Ki-67 proliferation score in advanced prostate cancer. (**A**) Sample flow (see Supplemental Figure 1A for more information). (**B**) Bee swarm probability mass density plot of Ki-67 score; pairwise 1-way ANOVA significance test *P*-values are reported. (**C**) Gleason score by Ki-67 score and metastatic stage (M0, non-metastatic, M1LV: low-volume metastatic, M1HV: high-volume metastatic). (**D**) Tumor stage distribution by Ki-67 score. (**E**) Multivariable mortality adjusted treatment effect hazard ratio (aHR) conditional on semiquantitative Ki-67 score (reference: Ki-67=0). (**F**) Forest plot of multivariable mortality hazard ratios. (**G** and **H**). Multivariable model-based conditional average treatment effect point across Ki-67 scores, with Ki-67 frequency bars: *P* values for partial deviance tests of interaction between Ki-67 score and addition of abiraterone. (**I** and **J**) Kaplan-Meier curves (overall survival) by allocated treatment and Ki-67 subgroup in metastatic disease, with subgroup aHR.

## Version 1 03/31/2026

In-Press Preview

## Version 2 05/15/2026

Electronic publication

## References

[1] Hanahan D, Weinberg RA (2000). The hallmarks of cancer. Cell.

[2] Rajpar S (2025). The benefit of combining docetaxel with androgen deprivation therapy in localized and metastatic hormone-sensitive prostate cancer is predicted by ERG expression: an analysis of two GETUG Phase 3 Trials. Eur Urol Oncol.

[3] Attard G (2023). Abiraterone acetate plus prednisolone with or without enzalutamide for patients with metastatic prostate cancer starting androgen deprivation therapy: final results from two randomised phase 3 trials of the STAMPEDE platform protocol. Lancet Oncol.

[4] Attard G (2022). Abiraterone acetate and prednisolone with or without enzalutamide for high-risk non-metastatic prostate cancer: a meta-analysis of primary results from two randomised controlled phase 3 trials of the STAMPEDE platform protocol. Lancet.

[5] Berney DM (2009). Ki-67 and outcome in clinically localised prostate cancer: analysis of conservatively treated prostate cancer patients from the Trans-Atlantic Prostate Group study. Br J Cancer.

[6] Grist E (2025). Tumor transcriptome-wide expression classifiers predict treatment sensitivity in advanced prostate cancers. Cell.

