# Supplementary material for: Tumor proliferation associates with greater sensitivity to androgen receptor pathway inhibition in metastatic prostate cancer
Source: J Clin Invest. 2026 Mar 31;136(10):e203201. doi: 10.1172/JCI203201 (PMC13178641; doi:10.1172/JCI203201)
Supplement: Supplemental data [file jci-136-203201-s027.pdf]

SUPPLEMENTAL DATA

Proliferation associates with greater sensitivity to androgen receptor pathway inhibition for metastatic prostate cancer

Larissa Mendes, Peter Dutey-Magni, Emily Grist, et al.

INDEX

Supplemental Figure 1	2
Supplemental Table 1: patient characteristics	4
Supplemental Table 2: prognostic analyses	5
Supplemental Table 3: predictive	6
Supplemental Methods	10
Data Availability	13
Author Contributions	14
Acknowledgements	14
References	15
Statistical Analysis Plan	17
STAMPEDE oversight committees, staff and collaborators	57

Supplemental Figure S1:

(**A**) Study sample flowchart. (**B**) Dot plot of Ki-67 score by trial arm by metastatic stage. (**C**) Scatter plot of serum PSA to Ki-67 score by metastatic stage. (**D**) Proportion of cases exposed to androgen deprivation therapy (ADT) prior to tissue collection by Ki-67 score and metastatic stage.

Supplemental Figure 1

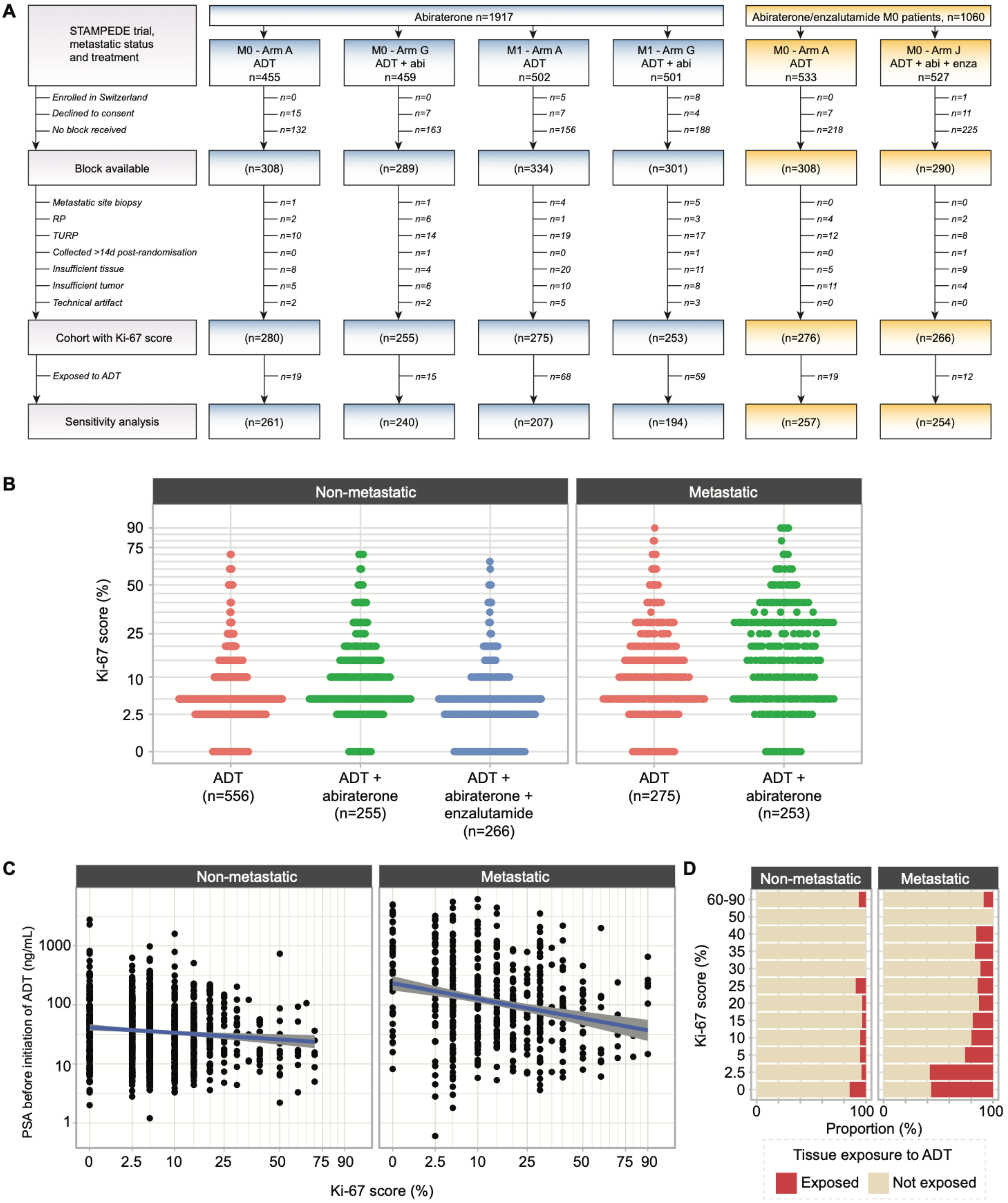

Supplemental Table 1: Patient characteristics in analytical versus overall intention-to-treat cohort

	Abiraterone trial				Abiraterone + enzalutamide trial	
	Non-metastatic patients		Metastatic patients		Non-metastatic patients	
**Characteristic, n (%); Median (IQR)**	**Biomarker cohort N = 535**	**Overall N = 914**	**Biomarker cohort N = 528**	**Overall N = 1,003**	**Biomarker cohort N = 542**	**Overall N = 1,060**
Treatment arm						
ADT	280 (52%)	455 (50%)	275 (52%)	502 (50%)	276 (51%)	533 (50%)
ADT + abiraterone	255 (48%)	459 (50%)	253 (48%)	501 (50%)		
ADT + abiraterone + enzalutamide	-	-	-	-	266 (49%)	527 (50%)
Age						
Under 70 years	315 (59%)	547 (60%)	346 (66%)	651 (65%)	309 (57%)	604 (57%)
70 years and over	220 (41%)	367 (40%)	182 (34%)	352 (35%)	233 (43%)	456 (43%)
Previously treated/relapsed	12 (2.2%)	37 (4.0%)	23 (4.4%)	62 (6.2%)	9 (1.7%)	31 (2.9%)
Nodal stage						
N+	214 (40%)	383 (42%)	302 (57%)	587 (59%)	191 (35%)	391 (37%)
N0	321 (60%)	530 (58%)	193 (37%)	350 (35%)	350 (65%)	667 (63%)
NX	0 (0%)	1 (0.1%)	33 (6.3%)	66 (6.6%)	1 (0.2%)	2 (0.2%)
Metastastic volume						
Low	0 (NA%)	0 (NA%)	224 (44%)	408 (44%)	-	-
High	0 (NA%)	0 (NA%)	288 (56%)	510 (56%)	-	-
Unknown	535	914	16	85	-	-
Local Gleason Score						
<=6	10 (1.9%)	17 (1.9%)	8 (1.5%)	19 (2.0%)	5 (0.9%)	14 (1.3%)
7	14 (2.6%)	22 (2.4%)	20 (3.8%)	41 (4.2%)	14 (2.6%)	37 (3.5%)
8	113 (21%)	195 (21%)	117 (22%)	214 (22%)	80 (15%)	179 (17%)
9	139 (26%)	231 (25%)	121 (23%)	223 (23%)	165 (30%)	271 (26%)
10	259 (48%)	446 (49%)	259 (49%)	476 (49%)	277 (51%)	556 (53%)
Unknown	0	3	3	30	1	3
T stage						
T0-T2	38 (7.2%)	69 (7.6%)	58 (12%)	107 (12%)	30 (5.6%)	56 (5.4%)
T3	442 (84%)	746 (83%)	296 (60%)	559 (61%)	455 (85%)	884 (85%)
T4	48 (9.1%)	88 (9.7%)	139 (28%)	255 (28%)	52 (9.7%)	105 (10%)
Unknown	7	11	35	82	5	15
Planned RT	442 (83%)	744 (81%)	20 (3.8%)	44 (4.4%)	484 (89%)	940 (89%)
Baseline PSA	40 (17, 78)	36 (15, 74)	101 (33, 370)	97 (27, 363)	33 (13, 78)	33 (14, 74)
Deaths (including linked records)	212 (40%)	368 (40%)	397 (75%)	744 (74%)	152 (28%)	285 (27%)
MPFS events (without linked records)	128 (24%)	216 (24%)	347 (66%)	657 (66%)	80 (15%)	150 (14%)
Time from biopsy to ADT (days)	26 (14, 41)	26 (14, 41)	14 (0, 29)	14 (0, 29)	22 (13, 36)	22 (13, 36)
Unknown	17	396	9	484	9	527
Trial treatment effect (hazard ratio for abiraterone +/- enzalutamide [95% confidence interval]	0.72 [0.55, 0.95]	0.72 [0.58, 0.88]	0.61 [0.50, 0.75]	0.61 [0.53, 0.70]	0.79 [0.57, 1.08]	0.70 [0.55, 0.89]

Supplemental Table 2: Prognostic analyses

Metadata

Column	Value	Definition
analysis	All cases	Main analysis using all samples
analysis	Cases not exposed to ADT	Sensitivity analysis of subset of participants not exposed to ADT before tissue collection
mstage	M1	Metastatic cohort
mstage	M1 Low	Low-volume metastases cohort
mstage	M1 High	High-volume metastases cohort
mstage	M0	Very high-risk locally advanced cohort
endpoint	OS	Overall survival
endpoint	MPFS	Metastatic progression-free survival
arms_cat	All	All STAMPEDE arms
arms_cat	ADT	Control arm (ADT only)
arms_cat	ADT + abiraterone	Abiraterone in addition to ADT arm
arms_cat	ADT + abiraterone + enzalutam	Abiraterone and enzalutamide in addition to ADT arm
adjustment	UVA	Univariable (unadjusted) model
adjustment	MVA multiple imputation	Multivariable (adjusted) model in multiple imputed dataset
HR	-	Hazard ratio of the signature
HR_lb	-	Lower bound of the 95% confidence interval of the hazard ratio
HR_ub	-	Upper bound of the 95% confidence interval of the hazard ratio
LRT	-	P-value of the partial likelihood ratio test of the addition of the prognostic signature into the survival model (chi2 with 1 degree of freedom).
sample_size	-	Sample size for the fitted model

feature	analysis	scale	metastatic_s	endpoint	arms_cat	adjustment	HR	HR_lb	HR_ub	LRT	sample_size
Ki67 (0.10 increments)	All cases	continuous	M0	OS	ADT	UVA	1.29	1.17	1.41	0.000	556
Ki67 (0.10 increments)	All cases	continuous	M0	OS	ADT	MVA multiple imputation	1.23	1.12	1.37	0.000	556
Ki67 (0.10 increments)	All cases	continuous	M0	OS	ADT + abiraterone	UVA	1.32	1.16	1.50	0.000	255
Ki67 (0.10 increments)	All cases	continuous	M0	OS	ADT + abiraterone	MVA multiple imputation	1.25	1.08	1.45	0.004	255
Ki67 (0.10 increments)	All cases	continuous	M0	OS	ADT + abiraterone + enzaluta	UVA	1.29	1.07	1.54	0.016	266
Ki67 (0.10 increments)	All cases	continuous	M0	OS	ADT + abiraterone + enzaluta	MVA multiple imputation	1.18	0.97	1.43	0.116	265
Ki67 (0.10 increments)	All cases	continuous	M1	OS	ADT	UVA	1.32	1.21	1.43	0.000	275
Ki67 (0.10 increments)	All cases	continuous	M1	OS	ADT	MVA multiple imputation	1.31	1.19	1.44	0.000	275
Ki67 (0.10 increments)	All cases	continuous	M1	OS	ADT + abiraterone	UVA	1.10	1.02	1.19	0.020	253
Ki67 (0.10 increments)	All cases	continuous	M1	OS	ADT + abiraterone	MVA multiple imputation	1.06	0.98	1.16	0.172	253
Ki67 (0.10 increments)	All cases	continuous	M1 High	OS	ADT	UVA	1.38	1.24	1.53	0.000	157
Ki67 (0.10 increments)	All cases	continuous	M1 High	OS	ADT	MVA multiple imputation	1.33	1.19	1.50	0.000	163
Ki67 (0.10 increments)	All cases	continuous	M1 Low	OS	ADT	UVA	1.22	1.04	1.43	0.021	108
Ki67 (0.10 increments)	All cases	continuous	M1 Low	OS	ADT	MVA multiple imputation	1.18	0.99	1.41	0.079	112
Ki67 ≥ 0.15	All cases	categorical	M0	OS	ADT	UVA	1.81	1.45	2.24	0.000	556
Ki67 ≥ 0.15	All cases	categorical	M0	OS	ADT	MVA multiple imputation	1.68	1.34	2.11	0.000	556
Ki67 ≥ 0.15	All cases	categorical	M0	OS	ADT + abiraterone	UVA	1.39	1.03	1.87	0.033	255
Ki67 ≥ 0.15	All cases	categorical	M0	OS	ADT + abiraterone	MVA multiple imputation	1.28	0.93	1.76	0.127	255
Ki67 ≥ 0.15	All cases	categorical	M0	OS	ADT + abiraterone + enzaluta	UVA	1.72	1.15	2.59	0.015	266
Ki67 ≥ 0.15	All cases	categorical	M0	OS	ADT + abiraterone + enzaluta	MVA multiple imputation	1.54	0.99	2.38	0.061	265
Ki67 ≥ 0.15	All cases	categorical	M1	OS	ADT	UVA	1.35	1.12	1.63	0.002	275
Ki67 ≥ 0.15	All cases	categorical	M1	OS	ADT	MVA multiple imputation	1.35	1.10	1.64	0.003	275
Ki67 ≥ 0.15	All cases	categorical	M1	OS	ADT + abiraterone	UVA	1.24	1.00	1.55	0.052	253
Ki67 ≥ 0.15	All cases	categorical	M1	OS	ADT + abiraterone	MVA multiple imputation	1.20	0.94	1.52	0.140	253
Ki67 ≥ 0.15	All cases	categorical	M1 High	OS	ADT	UVA	1.57	1.24	2.00	0.000	157
Ki67 ≥ 0.15	All cases	categorical	M1 High	OS	ADT	MVA multiple imputation	1.61	1.25	2.08	0.000	163
Ki67 ≥ 0.15	All cases	categorical	M1 Low	OS	ADT	UVA	1.13	0.82	1.55	0.456	108
Ki67 ≥ 0.15	All cases	categorical	M1 Low	OS	ADT	MVA multiple imputation	1.01	0.70	1.45	0.919	112
Ki67 (0.10 increments)	All cases	continuous	M0	MPFS	ADT	UVA	1.33	1.19	1.48	0.000	556
Ki67 (0.10 increments)	All cases	continuous	M0	MPFS	ADT	MVA multiple imputation	1.26	1.12	1.43	0.001	556
Ki67 (0.10 increments)	All cases	continuous	M1	MPFS	ADT	UVA	1.21	1.11	1.32	0.000	275
Ki67 (0.10 increments)	All cases	continuous	M1	MPFS	ADT	MVA multiple imputation	1.15	1.05	1.27	0.006	275
Ki67 (0.10 increments)	All cases	continuous	M1 High	MPFS	ADT	UVA	1.32	1.18	1.47	0.000	157
Ki67 (0.10 increments)	All cases	continuous	M1 High	MPFS	ADT	MVA multiple imputation	1.20	1.05	1.36	0.008	163
Ki67 (0.10 increments)	All cases	continuous	M1 Low	MPFS	ADT	UVA	1.16	1.00	1.34	0.063	108
Ki67 (0.10 increments)	All cases	continuous	M1 Low	MPFS	ADT	MVA multiple imputation	1.05	0.88	1.25	0.602	112
Ki67 ≥ 0.15	All cases	categorical	M0	MPFS	ADT	UVA	1.74	1.33	2.27	0.000	556
Ki67 ≥ 0.15	All cases	categorical	M0	MPFS	ADT	MVA multiple imputation	1.53	1.15	2.03	0.004	556
Ki67 ≥ 0.15	All cases	categorical	M1	MPFS	ADT	UVA	1.36	1.12	1.64	0.002	275
Ki67 ≥ 0.15	All cases	categorical	M1	MPFS	ADT	MVA multiple imputation	1.23	1.00	1.52	0.048	275
Ki67 ≥ 0.15	All cases	categorical	M1 High	MPFS	ADT	UVA	1.43	1.12	1.82	0.005	157
Ki67 ≥ 0.15	All cases	categorical	M1 High	MPFS	ADT	MVA multiple imputation	1.30	1.00	1.69	0.051	163
Ki67 ≥ 0.15	All cases	categorical	M1 Low	MPFS	ADT	UVA	1.29	0.93	1.80	0.129	108
Ki67 ≥ 0.15	All cases	categorical	M1 Low	MPFS	ADT	MVA multiple imputation	1.08	0.74	1.59	0.684	112

feature	analysis	scale	metastatic_s	endpoint	arms_cat	adjustment	HR	HR_lb	HR_ub	LRT	sample_size
Ki67 (0.10 increments)	Cases not exposed to ADT	continuous	M0	OS	ADT	UVA	1.29	1.17	1.42	0.000	518
Ki67 (0.10 increments)	Cases not exposed to ADT	continuous	M0	OS	ADT	MVA multiple imputation	1.23	1.11	1.36	0.000	518
Ki67 (0.10 increments)	Cases not exposed to ADT	continuous	M0	OS	ADT + abiraterone	UVA	1.31	1.15	1.49	0.000	240
Ki67 (0.10 increments)	Cases not exposed to ADT	continuous	M0	OS	ADT + abiraterone	MVA multiple imputation	1.22	1.05	1.41	0.012	240
Ki67 (0.10 increments)	Cases not exposed to ADT	continuous	M0	OS	ADT + abiraterone + enzaluta	UVA	1.40	1.15	1.72	0.004	254
Ki67 (0.10 increments)	Cases not exposed to ADT	continuous	M0	OS	ADT + abiraterone + enzaluta	MVA multiple imputation	1.33	1.06	1.65	0.018	253
Ki67 (0.10 increments)	Cases not exposed to ADT	continuous	M1	OS	ADT	UVA	1.37	1.24	1.50	0.000	207
Ki67 (0.10 increments)	Cases not exposed to ADT	continuous	M1	OS	ADT	MVA multiple imputation	1.34	1.20	1.49	0.000	207
Ki67 (0.10 increments)	Cases not exposed to ADT	continuous	M1	OS	ADT + abiraterone	UVA	1.11	1.01	1.21	0.030	194
Ki67 (0.10 increments)	Cases not exposed to ADT	continuous	M1	OS	ADT + abiraterone	MVA multiple imputation	1.03	0.93	1.15	0.526	194
Ki67 (0.10 increments)	Cases not exposed to ADT	continuous	M1 High	OS	ADT	UVA	1.44	1.26	1.65	0.000	102
Ki67 (0.10 increments)	Cases not exposed to ADT	continuous	M1 High	OS	ADT	MVA multiple imputation	1.38	1.20	1.58	0.000	105
Ki67 (0.10 increments)	Cases not exposed to ADT	continuous	M1 Low	OS	ADT	UVA	1.23	1.04	1.45	0.020	99
Ki67 (0.10 increments)	Cases not exposed to ADT	continuous	M1 Low	OS	ADT	MVA multiple imputation	1.19	0.99	1.43	0.080	102
Ki67 ≥ 0.15	Cases not exposed to ADT	categorical	M0	OS	ADT	UVA	1.85	1.48	2.31	0.000	518
Ki67 ≥ 0.15	Cases not exposed to ADT	categorical	M0	OS	ADT	MVA multiple imputation	1.70	1.35	2.15	0.000	518
Ki67 ≥ 0.15	Cases not exposed to ADT	categorical	M0	OS	ADT + abiraterone	UVA	1.30	0.96	1.77	0.097	240
Ki67 ≥ 0.15	Cases not exposed to ADT	categorical	M0	OS	ADT + abiraterone	MVA multiple imputation	1.17	0.84	1.64	0.341	240
Ki67 ≥ 0.15	Cases not exposed to ADT	categorical	M0	OS	ADT + abiraterone + enzaluta	UVA	1.74	1.16	2.61	0.014	254
Ki67 ≥ 0.15	Cases not exposed to ADT	categorical	M0	OS	ADT + abiraterone + enzaluta	MVA multiple imputation	1.59	1.03	2.45	0.044	253
Ki67 ≥ 0.15	Cases not exposed to ADT	categorical	M1	OS	ADT	UVA	1.37	1.11	1.70	0.004	207
Ki67 ≥ 0.15	Cases not exposed to ADT	categorical	M1	OS	ADT	MVA multiple imputation	1.28	1.02	1.61	0.033	207
Ki67 ≥ 0.15	Cases not exposed to ADT	categorical	M1	OS	ADT + abiraterone	UVA	1.26	0.97	1.65	0.079	194
Ki67 ≥ 0.15	Cases not exposed to ADT	categorical	M1	OS	ADT + abiraterone	MVA multiple imputation	1.13	0.84	1.52	0.416	194
Ki67 ≥ 0.15	Cases not exposed to ADT	categorical	M1 High	OS	ADT	UVA	1.47	1.09	1.97	0.010	102
Ki67 ≥ 0.15	Cases not exposed to ADT	categorical	M1 High	OS	ADT	MVA multiple imputation	1.49	1.07	2.07	0.015	105
Ki67 ≥ 0.15	Cases not exposed to ADT	categorical	M1 Low	OS	ADT	UVA	1.12	0.81	1.56	0.501	99
Ki67 ≥ 0.15	Cases not exposed to ADT	categorical	M1 Low	OS	ADT	MVA multiple imputation	0.99	0.67	1.46	0.922	102
Ki67 (0.10 increments)	Cases not exposed to ADT	continuous	M0	MPFS	ADT	UVA	1.35	1.21	1.51	0.000	518
Ki67 (0.10 increments)	Cases not exposed to ADT	continuous	M0	MPFS	ADT	MVA multiple imputation	1.26	1.11	1.42	0.001	518
Ki67 (0.10 increments)	Cases not exposed to ADT	continuous	M1	MPFS	ADT	UVA	1.25	1.14	1.37	0.000	207
Ki67 (0.10 increments)	Cases not exposed to ADT	continuous	M1	MPFS	ADT	MVA multiple imputation	1.17	1.05	1.30	0.005	207
Ki67 (0.10 increments)	Cases not exposed to ADT	continuous	M1 High	MPFS	ADT	UVA	1.37	1.20	1.57	0.000	102
Ki67 (0.10 increments)	Cases not exposed to ADT	continuous	M1 High	MPFS	ADT	MVA multiple imputation	1.23	1.06	1.44	0.007	105
Ki67 (0.10 increments)	Cases not exposed to ADT	continuous	M1 Low	MPFS	ADT	UVA	1.17	1.00	1.36	0.056	99
Ki67 (0.10 increments)	Cases not exposed to ADT	continuous	M1 Low	MPFS	ADT	MVA multiple imputation	1.04	0.87	1.25	0.667	102
Ki67 ≥ 0.15	Cases not exposed to ADT	categorical	M0	MPFS	ADT	UVA	1.81	1.37	2.38	0.000	518
Ki67 ≥ 0.15	Cases not exposed to ADT	categorical	M0	MPFS	ADT	MVA multiple imputation	1.54	1.15	2.07	0.004	518
Ki67 ≥ 0.15	Cases not exposed to ADT	categorical	M1	MPFS	ADT	UVA	1.43	1.14	1.79	0.002	207
Ki67 ≥ 0.15	Cases not exposed to ADT	categorical	M1	MPFS	ADT	MVA multiple imputation	1.21	0.95	1.55	0.115	207
Ki67 ≥ 0.15	Cases not exposed to ADT	categorical	M1 High	MPFS	ADT	UVA	1.30	0.96	1.76	0.085	102
Ki67 ≥ 0.15	Cases not exposed to ADT	categorical	M1 High	MPFS	ADT	MVA multiple imputation	1.20	0.86	1.68	0.271	105
Ki67 ≥ 0.15	Cases not exposed to ADT	categorical	M1 Low	MPFS	ADT	UVA	1.32	0.93	1.87	0.125	99
Ki67 ≥ 0.15	Cases not exposed to ADT	categorical	M1 Low	MPFS	ADT	MVA multiple imputation	1.10	0.72	1.67	0.667	102

Supplemental Table 3: Predictive

analyses Metadata

Column	Value	Definition
trial	abiraterone	Trial investigating the addition of abiraterone to ADT
trial	abiraterone +/- enzalutamide	Trial investigating the addition of abiraterone (with or without enzalutamide) to ADT
analysis	All cases	Main analysis using all samples
analysis	Cases not exposed to ADT	Sensitivity analysis of subset of participants not exposed to ADT before tissue collection
mstage	M1	Metastatic cohort
mstage	M0	Non-metastatic cohort
endpoint	OS	Overall survival
endpoint	MPFS	Metastatic progression-free survival
adjustment	MVA multiple imputation	Multivariable (adjusted) model in multiple imputed dataset
effect	-	Description of the effect corresponding to the hazard ratio
HR	-	Hazard ratio
HR_lb	-	Lower bound of the 95% confidence interval of the hazard ratio
HR_ub	-	Upper bound of the 95% confidence interval of the hazard ratio
sample_size	-	Sample size for the fitted model
LRT	-	P-value of the partial likelihood ratio test of the addition of the predictive effect into the survival model as an interaction term (chi2 with 1 degree of freedom).

trial	feature	analysis	scale	mstage	endpoint	adjustment	effect	HR	HR_lb	HR_ub	LRT	sample_size
abiraterone	Ki67 (continuous, 0.1 unit)	All cases	Continuous	M0	OS	MVA multiple imputation	Main effect of Ki-67 (per 10 perc. point incr.)	1.20	1.07	1.34	0.666	535
abiraterone	Ki67 (continuous, 0.1 unit)	All cases	Continuous	M0	OS	MVA multiple imputation	Main effect of abiraterone	0.66	0.45	0.97	0.666	535
abiraterone	Ki67 (continuous, 0.1 unit)	All cases	Continuous	M0	OS	MVA multiple imputation	Interaction effect of Ki-67 x abiraterone	1.04	0.87	1.24	0.666	535
abiraterone	Ki67 (continuous, 0.1 unit)	All cases	Continuous	M1	OS	MVA multiple imputation	Main effect of Ki-67 (per 10 perc. point incr.)	1.29	1.18	1.41	0.001	528
abiraterone	Ki67 (continuous, 0.1 unit)	All cases	Continuous	M1	OS	MVA multiple imputation	Main effect of abiraterone	0.74	0.55	1.00	0.001	528
abiraterone	Ki67 (continuous, 0.1 unit)	All cases	Continuous	M1	OS	MVA multiple imputation	Interaction effect of Ki-67 x abiraterone	0.82	0.73	0.92	0.001	528
abiraterone +/- enzalutamide	Ki67 (continuous, 0.1 unit)	All cases	Continuous	M0	OS	MVA multiple imputation	Main effect of Ki-67 (per 10 perc. point incr.)	1.25	1.14	1.38	0.680	1076
abiraterone +/- enzalutamide	Ki67 (continuous, 0.1 unit)	All cases	Continuous	M0	OS	MVA multiple imputation	Main effect of abiraterone	0.75	0.57	0.98	0.680	1076
abiraterone +/- enzalutamide	Ki67 (continuous, 0.1 unit)	All cases	Continuous	M0	OS	MVA multiple imputation	Interaction effect of Ki-67 x abiraterone	0.97	0.85	1.11	0.680	1076
abiraterone	Ki67 (≥ 0.15)	All cases	Binary	M0	OS	MVA multiple imputation	Main effect of Ki67 (≥ 0.15)	1.62	1.24	2.12	0.202	535
abiraterone	Ki67 (≥ 0.15)	All cases	Binary	M0	OS	MVA multiple imputation	Main effect of abiraterone	0.80	0.56	1.15	0.202	535
abiraterone	Ki67 (≥ 0.15)	All cases	Binary	M0	OS	MVA multiple imputation	Interaction effect of Ki-67 x abiraterone	0.55	0.35	0.86	0.202	535
abiraterone	Ki67 (≥ 0.15)	All cases	Binary	M1	OS	MVA multiple imputation	Main effect of Ki67 (≥ 0.15)	1.35	1.11	1.63	0.385	528
abiraterone	Ki67 (≥ 0.15)	All cases	Binary	M1	OS	MVA multiple imputation	Main effect of abiraterone	0.62	0.46	0.84	0.385	528
abiraterone	Ki67 (≥ 0.15)	All cases	Binary	M1	OS	MVA multiple imputation	Interaction effect of Ki-67 x abiraterone	0.51	0.39	0.68	0.385	528
abiraterone +/- enzalutamide	Ki67 (≥ 0.15)	All cases	Binary	M0	OS	MVA multiple imputation	Main effect of Ki67 (≥ 0.15)	1.71	1.37	2.12	0.122	1076
abiraterone +/- enzalutamide	Ki67 (≥ 0.15)	All cases	Binary	M0	OS	MVA multiple imputation	Main effect of abiraterone	0.80	0.62	1.03	0.122	1076
abiraterone +/- enzalutamide	Ki67 (≥ 0.15)	All cases	Binary	M0	OS	MVA multiple imputation	Interaction effect of Ki-67 x abiraterone	0.56	0.39	0.81	0.122	1076
abiraterone	Ki67 (continuous, 0.1 unit)	All cases	Continuous	M0	MPFS	MVA multiple imputation	Main effect of Ki-67 (per 10 perc. point incr.)	1.28	1.13	1.46	0.283	535
abiraterone	Ki67 (continuous, 0.1 unit)	All cases	Continuous	M0	MPFS	MVA multiple imputation	Main effect of abiraterone	0.40	0.23	0.67	0.283	535
abiraterone	Ki67 (continuous, 0.1 unit)	All cases	Continuous	M0	MPFS	MVA multiple imputation	Interaction effect of Ki-67 x abiraterone	1.12	0.91	1.37	0.283	535
abiraterone	Ki67 (continuous, 0.1 unit)	All cases	Continuous	M1	MPFS	MVA multiple imputation	Main effect of Ki-67 (per 10 perc. point incr.)	1.17	1.07	1.28	0.187	528
abiraterone	Ki67 (continuous, 0.1 unit)	All cases	Continuous	M1	MPFS	MVA multiple imputation	Main effect of abiraterone	0.47	0.34	0.65	0.187	528
abiraterone	Ki67 (continuous, 0.1 unit)	All cases	Continuous	M1	MPFS	MVA multiple imputation	Interaction effect of Ki-67 x abiraterone	0.92	0.82	1.04	0.187	528
abiraterone +/- enzalutamide	Ki67 (continuous, 0.1 unit)	All cases	Continuous	M0	MPFS	MVA multiple imputation	Main effect of Ki-67 (per 10 perc. point incr.)	1.28	1.14	1.44	0.611	1076
abiraterone +/- enzalutamide	Ki67 (continuous, 0.1 unit)	All cases	Continuous	M0	MPFS	MVA multiple imputation	Main effect of abiraterone	0.47	0.32	0.69	0.611	1076
abiraterone +/- enzalutamide	Ki67 (continuous, 0.1 unit)	All cases	Continuous	M0	MPFS	MVA multiple imputation	Interaction effect of Ki-67 x abiraterone	1.04	0.88	1.23	0.611	1076

trial	feature	analysis	scale	mstage	endpoint	adjustment	effect	HR	HR_lb	HR_ub	LRT	sample_size
abiraterone	Ki67 (continuous, 0.1 unit)	Cases not exposed to ADT	Continuous	M0	OS	MVA multiple imputation	Main effect of Ki-67 (per 10 perc. point incr.)	1.20	1.07	1.35	0.751	501
abiraterone	Ki67 (continuous, 0.1 unit)	Cases not exposed to ADT	Continuous	M0	OS	MVA multiple imputation	Main effect of abiraterone	0.66	0.45	0.99	0.751	501
abiraterone	Ki67 (continuous, 0.1 unit)	Cases not exposed to ADT	Continuous	M0	OS	MVA multiple imputation	Interaction effect of Ki-67 x abiraterone	1.03	0.86	1.23	0.751	501
abiraterone	Ki67 (continuous, 0.1 unit)	Cases not exposed to ADT	Continuous	M1	OS	MVA multiple imputation	Main effect of Ki-67 (per 10 perc. point incr.)	1.30	1.18	1.44	0.001	401
abiraterone	Ki67 (continuous, 0.1 unit)	Cases not exposed to ADT	Continuous	M1	OS	MVA multiple imputation	Main effect of abiraterone	0.80	0.55	1.15	0.001	401
abiraterone	Ki67 (continuous, 0.1 unit)	Cases not exposed to ADT	Continuous	M1	OS	MVA multiple imputation	Interaction effect of Ki-67 x abiraterone	0.80	0.70	0.91	0.001	401
abiraterone +/- enzalutamide	Ki67 (continuous, 0.1 unit)	Cases not exposed to ADT	Continuous	M0	OS	MVA multiple imputation	Main effect of Ki-67 (per 10 perc. point incr.)	1.26	1.14	1.39	0.768	1011
abiraterone +/- enzalutamide	Ki67 (continuous, 0.1 unit)	Cases not exposed to ADT	Continuous	M0	OS	MVA multiple imputation	Main effect of abiraterone	0.75	0.57	1.00	0.768	1011
abiraterone +/- enzalutamide	Ki67 (continuous, 0.1 unit)	Cases not exposed to ADT	Continuous	M0	OS	MVA multiple imputation	Interaction effect of Ki-67 x abiraterone	0.98	0.85	1.13	0.768	1011
abiraterone	Ki67 (≥ 0.15)	Cases not exposed to ADT	Binary	M0	OS	MVA multiple imputation	Main effect of Ki67 (≥ 0.15)	1.68	1.27	2.22	0.090	501
abiraterone	Ki67 (≥ 0.15)	Cases not exposed to ADT	Binary	M0	OS	MVA multiple imputation	Main effect of abiraterone	0.84	0.57	1.22	0.090	501
abiraterone	Ki67 (≥ 0.15)	Cases not exposed to ADT	Binary	M0	OS	MVA multiple imputation	Interaction effect of Ki-67 x abiraterone	0.50	0.32	0.79	0.090	501
abiraterone	Ki67 (≥ 0.15)	Cases not exposed to ADT	Binary	M1	OS	MVA multiple imputation	Main effect of Ki67 (≥ 0.15)	1.27	1.02	1.59	0.538	401
abiraterone	Ki67 (≥ 0.15)	Cases not exposed to ADT	Binary	M1	OS	MVA multiple imputation	Main effect of abiraterone	0.61	0.41	0.89	0.538	401
abiraterone	Ki67 (≥ 0.15)	Cases not exposed to ADT	Binary	M1	OS	MVA multiple imputation	Interaction effect of Ki-67 x abiraterone	0.52	0.38	0.71	0.538	401
abiraterone +/- enzalutamide	Ki67 (≥ 0.15)	Cases not exposed to ADT	Binary	M0	OS	MVA multiple imputation	Main effect of Ki67 (≥ 0.15)	1.75	1.40	2.18	0.048	1011
abiraterone +/- enzalutamide	Ki67 (≥ 0.15)	Cases not exposed to ADT	Binary	M0	OS	MVA multiple imputation	Main effect of abiraterone	0.84	0.65	1.10	0.048	1011
abiraterone +/- enzalutamide	Ki67 (≥ 0.15)	Cases not exposed to ADT	Binary	M0	OS	MVA multiple imputation	Interaction effect of Ki-67 x abiraterone	0.53	0.36	0.77	0.048	1011
abiraterone	Ki67 (continuous, 0.1 unit)	Cases not exposed to ADT	Continuous	M0	MPFS	MVA multiple imputation	Main effect of Ki-67 (per 10 perc. point incr.)	1.29	1.13	1.47	0.337	501
abiraterone	Ki67 (continuous, 0.1 unit)	Cases not exposed to ADT	Continuous	M0	MPFS	MVA multiple imputation	Main effect of abiraterone	0.40	0.23	0.70	0.337	501
abiraterone	Ki67 (continuous, 0.1 unit)	Cases not exposed to ADT	Continuous	M0	MPFS	MVA multiple imputation	Interaction effect of Ki-67 x abiraterone	1.11	0.90	1.36	0.337	501
abiraterone	Ki67 (continuous, 0.1 unit)	Cases not exposed to ADT	Continuous	M1	MPFS	MVA multiple imputation	Main effect of Ki-67 (per 10 perc. point incr.)	1.17	1.06	1.30	0.118	401
abiraterone	Ki67 (continuous, 0.1 unit)	Cases not exposed to ADT	Continuous	M1	MPFS	MVA multiple imputation	Main effect of abiraterone	0.49	0.32	0.73	0.118	401
abiraterone	Ki67 (continuous, 0.1 unit)	Cases not exposed to ADT	Continuous	M1	MPFS	MVA multiple imputation	Interaction effect of Ki-67 x abiraterone	0.90	0.78	1.03	0.118	401
abiraterone +/- enzalutamide	Ki67 (continuous, 0.1 unit)	Cases not exposed to ADT	Continuous	M0	MPFS	MVA multiple imputation	Main effect of Ki-67 (per 10 perc. point incr.)	1.29	1.15	1.45	0.659	1011
abiraterone +/- enzalutamide	Ki67 (continuous, 0.1 unit)	Cases not exposed to ADT	Continuous	M0	MPFS	MVA multiple imputation	Main effect of abiraterone	0.48	0.33	0.72	0.659	1011
abiraterone +/- enzalutamide	Ki67 (continuous, 0.1 unit)	Cases not exposed to ADT	Continuous	M0	MPFS	MVA multiple imputation	Interaction effect of Ki-67 x abiraterone	1.04	0.88	1.23	0.659	1011

SUPPLEMENTAL METHODS

Sex as a biological variable

All participants had a diagnosis of prostate adenocarcinoma and were consequently biologically male.

Trial design and participants

The STAMPEDE multi-arm, multi-stage platform protocol (1,2) (ISRCTN 78818544 NCT00268476) enrolled participants to the abiraterone and abiraterone with enzalutamide trials. All participants had a diagnosis of prostate adenocarcinoma and were consequently biologically male. Participants were to receive a minimum of two years ADT for newly diagnosed localised high-risk disease node-negative (M0N0) who had at least two of tumor stage category T3/4, PSA ≥40ng/ml, Gleason score 8-10; or node-positive (M0N1); or life-long ADT if they had metastatic disease (M1) confirmed on conventional whole-body computed tomography and/or technetium bone scans. Metastatic disease volume was classified after completion of accrual using baseline imaging, with 'high-volume' disease defined as per the CHAARTED trial (presence of visceral metastases and/or at least four bone lesions with at least one lesion outside of the vertebral column and/or pelvis) (3). Serum PSA, prior to hormone therapy, was obtained up to 6 months before randomisation.

Study approvals

This ancillary study was approved by an independent Research Ethics Committee (REC reference 18/LO/1235) and the STAMPEDE Trial Management Group and Trial Steering Committee. It includes participants recruited to United Kingdom sites and

who signed informed consent to participate in the trial and to donate their tissue for research (REC reference 04/MRE07/35).

Sample retrieval and Ki-67 scoring

Specimens from transrectal needle core biopsies were eligible (to ensure consistency in the Ki-67 staining and scoring methodology) if collected ≤14 days after randomisation. Diagnostic formalin-fixed paraffin-embedded tissue blocks were retrieved from STAMPEDE trial sites and centralised in the Wales Cancer Biobank, where all identifying details were removed. Samples and reports were labelled with the individual's STAMPEDE trial number. Tumor samples were transferred to the UCL Cancer Institute (London, UK) for processing and where sample type was recorded. Freshly-cut haematoxylin and eosin slides were centrally assessed by two Uropathologists (LM and DB) for tumor cellularity and scored using contemporary Gleason score and corresponding grade groups (ISUP2014/WHO2016(4)). The adjacent section of the tumor block with the highest Gleason score (index lesion) was immunostained for Ki-67 using MIB-1 antibody (DAKO, CARPInteria, CA, USA) and normal tonsil as a positive control. We followed technical recommendations established by the Ki-67 working group in breast cancer (5,6) and analytically validated for prostate cancer (7,8). Areas of intraductal carcinoma and intraepithelial neoplasia were excluded from the score and a minimum of 500 tumor cells were required. The Ki-67 score was defined as the percentage of positively stained tumor cells amongst the total number of malignant cells, known as the unweighted global assessment method, common practice for the assessment of proliferation index in other organs (5,9,10). Scoring was semi-quantitative in 5%: numerical values were assigned to bins as follows: 0 for '≤0.01'; 0.025 for '>0.01–<0.05'; 0.05 for '≥0.05–<0.10'; 0.10 for

‘≥0.10–<0.15’; thereafter continuing in 5% increments using the value of the lower bound of scoring intervals.

Outcomes

The primary outcome measure was overall survival (time from randomisation until death from any cause). Dates of death were obtained from trial case report forms or, in the case of participants in England and Wales with valid consent, linked civil registrations of death obtained in February 2024 to gather long-term mortality data. The secondary outcome was metastatic progression-free survival defined as the time from randomisation until first of: radiologically confirmed distant metastases, skeletal-related event with confirmed disease progression, or death from prostate cancer.

Statistical analysis

Associations between the Ki-67 cell proliferation index and baseline disease stage and Gleason score were tested using one-way analyses of variances on the square-root normalised score. We hypothesised that an increased Ki-67 score was associated with shorter survival (prognostic effect) and a greater benefit from treatment intensification (predictive effect). The prognostic effect of the Ki-67 score on survival outcomes was estimated using Cox Proportional Hazards models separately in each trial arm. The target of inference was the change of deviance following the introduction of a main effect for the Ki-67 score into the regression and tested with a χ^z test (partial likelihood ratio test). The predictive effect was estimated by adding an interaction effect between trial allocation and the Ki-67 score and testing the change in deviance again. The proportional hazards assumption was evaluated using a Grambsch-Therneau test of Schoenfeld residuals against log-transformed time.

Ki-67 was analysed both as a binary variable dichotomised around the median in metastatic patients (≥0.15, <0.15), and as a continuous variable with best-fitting fractional polynomial transformation (11). Survival models were adjusted for the following baseline variables: age at randomisation (years, continuous), WHO performance status (0 vs 1-2), regular aspirin or NSAID use (no vs yes), serum PSA (prior to start of ADT and within 6 months of randomisation, continuous, log-transformed), Gleason score as assessed by the local pathologist (≤6; 7; ≥8), tumor stage (T0-T2; T3; T4), nodal stage and in metastatic cases disease volume categorisation (low volume vs high volume). Missing Gleason scores were imputed using the dominant category (≥8). Other baseline characteristics were assumed to be missing at random and handled using multiple imputation with chained equations.

Analyses were repeated in the subset of participant who had not been exposed to first-generation androgen suppression, luteinising hormone-releasing hormone (LHRH) antagonist, or more than 5 days of LHRH agonist. This was to establish whether any of the findings were sensitive to changes in protein expression induced by ADT.

DATA AVAILABILITY

Individual participant data can be requested to the corresponding author or via the general enquiries email: mrcctu.ctuenquiries@ucl.ac.uk, and will be shared as per the moderated access approach of the Medical Research Council Clinical Trials Unit at University College London. Upon approval, individual participant data that underlie the results reported in this Article, after de-identification (text, tables, figures, and appendices), will be provided with a data dictionary, protocol, and case record forms relevant to the data. Values for all data points in graphs are reported in the Supporting Data Values file

AUTHORS CONTRIBUTIONS

Conception and design: LM, DB and GA

Obtained funding: EG, CS, MP, NJ, DB and GA

Statistical analysis plan: LM PD-M, with input from LB, and GA

STRATOSPHERE protocol: LM, EG, MP and GA

STAMPEDE trial management group: CA, MS, MP, NC, LCB, NJ, and GA

Data generation: LM, AS, NA

Pathology: LM, DB, SV, SL, DW and AW

Statistical analysis: PD-M, LB First draft of manuscript: LM, PD-M, and GA. Approved manuscript: all.

Co–first authorship was assigned based on complementary, independent, and equally substantial intellectual contributions that were both essential to the conception, execution, analysis, and reporting of this study. LM performed the Ki-67 scoring with DB and co-developed the analysis plan and manuscript. PD-M co-developed the analysis plan, performed all statistical analyses, designed the analytical codebase, and co-developed the manuscript. Authorship order reflects equal intellectual contribution across biological and statistical domains. The final interpretation of the results and decision to submit was made by the STAMPEDE trial management group.

ACKNOWLEDGEMENTS

We thank the men who were diagnosed with prostate cancer and nonetheless allowed for their tissue to be included in this research project so that others may benefit. We thank the invaluable work of the Wales Cancer Biobank in retrieving, anonymising and tracking all samples. We also acknowledge the members of the Trial Steering Committee and Independent Data Monitoring Committee.

REFERENCES

1. Attard G, Murphy L, Clarke NW, et al. Abiraterone acetate plus prednisolone with or without enzalutamide for patients with metastatic prostate cancer starting androgen deprivation therapy: final results from two randomised phase 3 trials of the STAMPEDE platform protocol. *Lancet Oncol*. 2023;24(5):443-456. doi:10.1016/S1470-2045(23)00148-1
2. Attard G, Murphy L, Clarke NW, et al. Abiraterone acetate and prednisolone with or without enzalutamide for high-risk non-metastatic prostate cancer: a meta-analysis of primary results from two randomised controlled phase 3 trials of the STAMPEDE platform protocol. *The Lancet*. 2022;399(10323):447-460. doi:10.1016/S0140-6736(21)02437-5
3. Sweeney CJ, Chen YH, Carducci M, et al. Chemohormonal Therapy in Metastatic Hormone-Sensitive Prostate Cancer. *New England Journal of Medicine*. 2015;373(8):737-746. doi:10.1056/NEJMoa1503747
4. Epstein JI, Egevad L, Amin MB, Delahunt B, Srigley JR, Humphrey PA. The 2014 International Society of Urological Pathology (ISUP) Consensus Conference on Gleason Grading of Prostatic Carcinoma. *American Journal of Surgical Pathology*. 2016;40(2):244-252. doi:10.1097/PAS.0000000000000530
5. Dowsett M, Nielsen TO, A'Hern R, et al. Assessment of Ki67 in breast cancer: recommendations from the International Ki67 in Breast Cancer working group. *J Natl Cancer Inst*. 2011;103(22):1656-1664. doi:10.1093/jnci/djr393
6. Leung SCY, Nielsen TO, Zabaglo LA, et al. Analytical validation of a standardised scoring protocol for Ki67 immunohistochemistry on breast cancer excision whole sections: an international multicentre collaboration. *Histopathology*. 2019;75(2):225-235. doi:10.1111/his.13880

7. Kammerer-Jacquet SF, Ahmad A, Møller H, et al. Ki-67 is an independent predictor of prostate cancer death in routine needle biopsy samples: proving utility for routine assessments. *Modern Pathology*. 2019;32(9):1303-1309. doi:10.1038/s41379-019-0268-y
8. Berney DM, Gopalan A, Kudahetti S, et al. Ki-67 and outcome in clinically localised prostate cancer: analysis of conservatively treated prostate cancer patients from the Trans-Atlantic Prostate Group study. *Br J Cancer*. 2009;100(6):888-893. doi:10.1038/sj.bjc.6604951
9. Leung SCY, Nielsen TO, Zabaglo L, et al. Analytical validation of a standardized scoring protocol for Ki67: phase 3 of an international multicenter collaboration. *NPJ Breast Cancer*. 2016;2(1):16014. doi:10.1038/npjbcancer.2016.14
10. Polley MYC, Leung SCY, Gao D, et al. An international study to increase concordance in Ki67 scoring. *Modern Pathology*. 2015;28(6):778-786. doi:10.1038/modpathol.2015.38
11. Royston P, Sauerbrei W. A new approach to modelling interactions between treatment and continuous covariates in clinical trials by using fractional polynomials. *Stat Med*. 2004;23(16):2509-2525. doi:10.1002/sim.1815

DocuSign Envelope ID: B9663506-7921-47F0-B9D8-557D7FE39808

STAMPEDE/STRATOSPHERE STATISTICAL ANALYSIS PLAN

STAMPEDE CLINICAL TRIAL APPROVALS
MRC PR08

ISRCTN number:	ISRCTN78818544
EUDRACT number:	2004-000193-31
CTA number:	00316/0026/001-0001
NCT number:	NCT00268476

STRATOSPHERE PROTOCOL APPROVALS

REC Reference:	18/LO/1235
IRAS Project ID:	244406

Predictive and prognostic associations with transcriptome-wide classifiers and morphological features in the docetaxel and abiraterone trials of the STAMPEDE platform protocol

MRC Clinical Trials Unit at UCL
2.00
Tel: +44 (0)20 7670 4798
Fax: +44 (0)20 7670 4818
Email: mrcctu.stampede@ucl.ac.uk

Version:
2.00 (04/03/2024)

Contacts
Emily Grist
Peter Dutey
Louise Brown
Gert Attard

REVISION HISTORY

Version	Author	Date	Reason for Revision
**Draft 2.00**	**PD**	**04/03/2024**	**Finalising for signature**
Draft 1.04	PD/EG/GA	05/02/2024	Drafting major review integrating abiraterone and docetaxel comparisons into a single analysis. Eligibility criteria are unified and now include relapsed patients.
Draft 1.03	Peter Dutey	22/08/2023	Adding tumour stage to the list of covariates to be omitted in the sensitivity analysis and using consistent designation of metastasis volume.
Draft 1.02	Peter Dutey	16/08/2023	Updating gene expression sample sizes and flowcharts following discovery that two blocks were not diagnostic biopsies.
Draft 1.01	Peter Dutey	11/08/2023	Changing adjustment specifications following discovery of inconsistent estimates between the complete case analysis and multiple imputation analysis. To resolve the conflict, a sensitivity analysis without adjustment for disease burden (responsible for data missingness) is introduced.
Final 1.00	Emily Grist	03/07/2023	Finalise for signature
Draft 0.06	Peter Dutey	30/06/2023	Updating sample numbers and incorporating further methodological comments
Draft 0.05	Peter Dutey	16/06/2023	Revisions decided at team meeting on 05/06
Draft 0.04	Peter Dutey	18/05/2023	Revisions decided at team meeting on 16/05
Draft 0.03	Emily Grist	15/03/2023	Incorporating comments from Veracyte
Draft 0.02	Peter Dutey	30/03/2023	Adding flowcharts, incorporating aims and stat methodology
Draft 0.01	Emily Grist	13/03/2023	Initial version

SIGNATURES

Name	Role	Signature	Date
Emily Grist	Clinical Research Fellow, UCL Cancer Institute	DocuSigned by: [illegible] 27300E0052F1454...	05-Mar-2024
Gert Attard	Chair of Medical Oncology, UCL Cancer Institute	DocuSigned by: Gert Attard BD146D1ADAA64A5...	05-Mar-2024
Louise Brown	Professor in Medical Statistics, MRC CTU at UCL	DocuSigned by: Louise Brown B826E52C896F494...	05-Mar-2024
Peter Dutey	Senior Research Fellow, MRC CTU at UCL	DocuSigned by: Peter Dutey-Magni 3B92FBA4A8E54B0...	05-Mar-2024
James Proudfoot	Senior Statistician, Veracyte	DocuSigned by: James Proudfoot C04435601A784C8...	05-Mar-2024
Elai Davicioni	Medical Director, Veracyte	DocuSigned by: Elai Davicioni EE6E7B4C31B5494...	05-Mar-2024
Nick James	Professor of Prostate and Bladder Cancer Research, STAMPEDE Chief Investigator, Institute of Cancer Research	DocuSigned by: Nick James C37608160038[illegible]	05-Mar-2024

CONTENTS

CONTENTS IV

1 BACKGROUND 5
1.1 PREAMBLE 5
1.2 BACKGROUND AND RATIONALE 5
1.3 SUMMARY OF THE STAMPEDE TRIAL 6
1.4 BIOMARKER POPULATION: STRATOSPHERE STUDY 8
1.5 JUSTIFICATION FOR GENE EXPRESSION SIGNATURE TESTING 9
1.5.1 Decipher classifier 9
1.5.2 Luminal-basal sub-typing 9
1.5.3 Transcriptome-wide classifiers of biological interest 10
1.6 JUSTIFICATION FOR KI-67 TESTING 10

2 STUDY METHODS 12
2.1 ELIGIBILITY CRITERIA 12
2.2 RANDOMISATION 12
2.3 OUTCOMES 13
2.4 SAMPLE PROCESSING 15
2.5 KI-67 SCORING AND GLEASON SCORE ASSESSMENT 16
2.6 PATIENT COHORT 17
2.7 BIOMARKERS TO BE TESTED 17
2.8 DISEASE VOLUME ASSESSMENT 17
2.9 TIMING OF ANDROGEN DEPRIVATION 18
2.10 TRIAL DATA LOCK AND VERIFICATION 18

3 STATISTICAL PRINCIPLES 19
3.1 AIMS 19
3.2 OBJECTIVES 19
3.2.1 Prognostic effect of 59 gene signatures in the abiraterone and docetaxel trial comparisons 19
3.2.2 Basal-luminal subtyping (metastatic disease) 20
3.2.3 Basal-luminal subtyping (localised disease) 20
3.2.4 Decipher and Ki-67 20
3.2.5 Exploratory objectives 21
3.3 STATISTICAL MODELLING 21
3.4 SENSITIVITY ANALYSES 22

REFERENCES 23

APPENDIX 1: RANDOMISATION PERIODS 28

APPENDIX 2: POWER CALCULATIONS 29
3.5 PAM50 SIGNATURE 29
3.6 PSC SIGNATURE 30
3.7 KI-67 SCORE 31

APPENDIX 3: LIST OF GENE EXPRESSION SIGNATURES FOR PROGNOSTIC ANALYSES 33

1 BACKGROUND

1.1 PREAMBLE

This statistical analysis plan was developed after completion of analysis of gene expression data from patients randomised in the STAMPEDE abiraterone comparison (version 1.0, dated 21 Mar-2022). This analysis led to a number of discoveries, in summary:

1. A strong and significant association between the Decipher signature (continuous and split by median categorisation) and overall survival (OS) or metastases-free survival (MFS) in both M1 and M0 cohorts respectively,
2. A strong association between immune signatures (most notably IFN-HM) and MFS in M0 but not M1,
3. A strong association between gene expression based activation of PI3K signaling and shorter OS in M1 patients.

These results were presented in-part at ESMO 2022 and described in a pre-print [1]. In parallel with these analyses, gene expression profiling of the docetaxel comparison continued; a decision was made by the STAMPEDE translational group to combine the data from the abiraterone and docetaxel comparisons. This allowed increased power for testing unexpected findings from the abiraterone comparison. Rather than perform testing of prognostic associations in the docetaxel comparison cohort separately from the abiraterone comparison, we elected to combine them and repeat the analyses – this would provide a result with greater certainty and maximise the value of the data. A further change is the use of record linkage with death registrations to secure a greater number of death events previously missed due end of follow-up Nov 2018 and Nov 2021 for the docetaxel and abiraterone comparison trials respectively. This increases statistical power in each experiment for the primary endpoint (overall survival) and one secondary endpoint (prostate-specific cancer survival). We include details on alignment of these analyses in this SAP.

The STAMPEDE platform protocol included a number of independently-powered Phase III trials that are referred to as “comparisons” when communicating with regulators given each is covered by the same protocol number.

1.2 BACKGROUND AND RATIONALE

The survival of patients commencing long-term androgen deprivation therapy (ADT) with high-risk localised or metastatic disease is highly variable. The STAMPEDE trials, in addition to a number of other phase 3 randomised control trials, have demonstrated that treatment intensification with either an AR targeted therapy [2–7] or docetaxel chemotherapy [8–10] improves survival in patents diagnosed with advanced hormone-sensitive prostate cancer commencing long-term ADT and is now standard-of-care for patients with metastatic disease. The choice of treatment is currently dictated by which drug is available within healthcare systems and/or patient preference. More recent trial data [4,11] has suggested triple therapy with ADT in addition to both intensified AR targeted therapy and docetaxel may benefit metastatic patients (particularly those presenting with *de novo* high volume metastatic disease). It has taken longer to ascertain the benefit of treatment intensification in high-risk non-metastatic patients, largely due to this cohorts overall longer survival. A meta-analyses [3] pooling data from two randomised control phase 3 trials conducted within the STAMPEDE platform protocol demonstrated the addition of AR targeted therapy (abiraterone or abiraterone in addition to enzalutamide) was associated with significantly higher rates of metastases free survival. There is insufficient evidence to suggest docetaxel administered to patients with high-risk non-metastatic disease improves outcomes [10].

Despite clinical benefit from treatment intensification observed within overall trial cohorts, we hypothesised that patients may be further stratified into molecularly-defined subgroups that demonstrate differential clinical outcome and response to treatment. A large meta-analysis of trials randomising patients to ADT versus ADT with docetaxel has demonstrated that disease burden at baseline and presentation (synchronous versus metachronous metastatic disease) impacts the clinical benefit observed with docetaxel treatments [12]. Targeting treatments more precisely to patients with advanced disease will improve patient outcomes and quality of life, as well as reduce both physical and financial toxicity.

95% of patients recruited to the STAMPEDE platform protocol consented to use of their diagnostic tissue in additional research. Molecular data generated from this has given insights into how some molecular features impact clinical outcome. We have demonstrated that the burden of genomic copy number alteration in the primary prostate tumour associates with clinical outcome irrespective of metastatic state and that gene expression signatures also associate with clinical outcome [13,14]. We will now focus on whether gene expression classifiers can predict which patients stand to benefit the most from docetaxel treatment and plan to pool cohorts of patients randomised to both docetaxel and abiraterone STAMPEDE Phase III trials to increase our power to detect differences in molecularly defined subgroups.

This analysis plan is to be reviewed in combination with the analysis plan dated 21 March 2022 that defined the gene expression analysis performed on patients randomised in the abiraterone comparison.

1.3 SUMMARY OF THE STAMPEDE TRIAL

STAMPEDE is a multi-centre, platform protocol, including a number of independently-powered randomised controlled trials (Figure 1) that recruited patients with locally advanced (M0) or metastatic (M1) prostate cancer starting long-term ADT. Eligible patients had either newly-diagnosed disease or very high-risk relapsed disease (previously treated with radical radiotherapy or surgery).

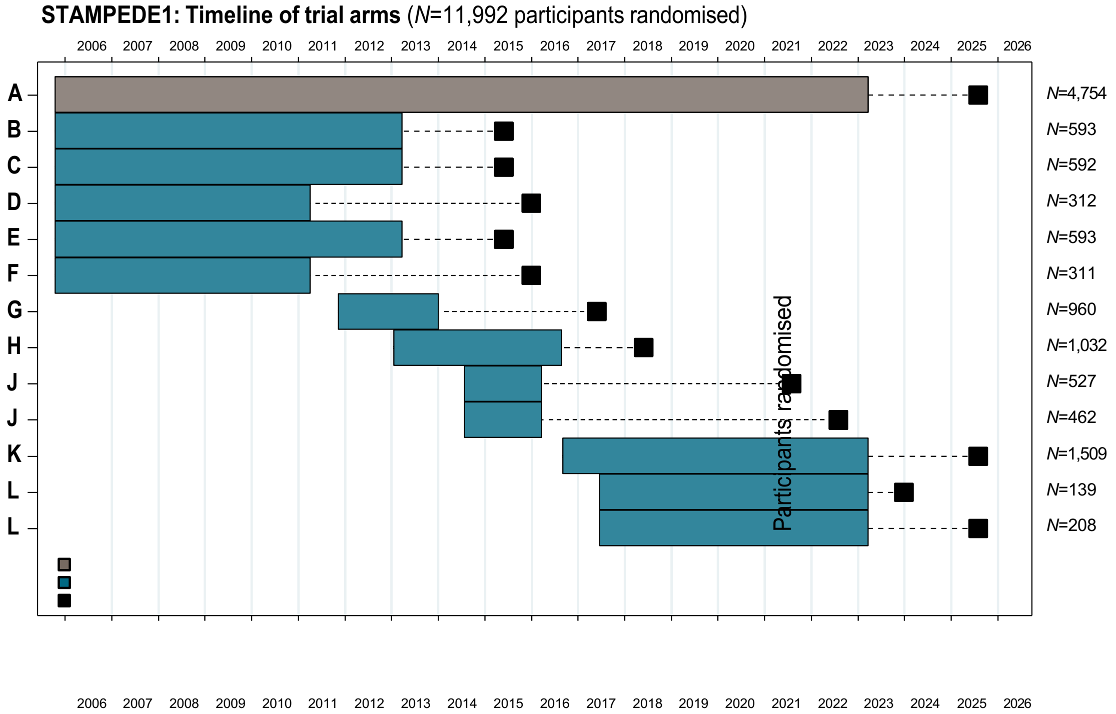

Figure 1 STAMPEDE trial arm timeline

DocuSign Envelope ID: B9663506-7921-47F0-B9D8-557D7FE39808

The trial uses multi-arm, multi-stage (MAMS) methods to simultaneously assess a number of different research treatments. The trial aims to assess the effects of adding therapies to standard-of-care (SOC). Every evaluation (10 in total) was independently powered and within the platform protocol was referred to as a “research comparison” (Table 1). The trial initially started with Arms A to F. Additional research arms have been included in the trial over time. Patients in the control arm receive standard-of-care (ADT with or without local radiotherapy); the research arms have this standard combined with additional investigational treatments, except for patients allocated to the tE2 arm who receive transdermal oestradiol in place of standard hormone treatment.

Metastatic patients included in this transcriptome-wide ancillary study were recruited into STAMPEDE when the standard-of-care was ADT alone, achieved using luteinising hormone-releasing hormone (LHRH) analogues or antagonists, dual androgen blockade (DAB: long-term anti-androgens in combination with LHRH agonist) or bilateral orchidectomy according to local practice (bicalutamide for non-metastatic patients was allowed in some early versions of the protocol). This standard-of-care is also the backbone of therapy for the research arms. For patients recruited from 2016, docetaxel was added to standard-of-care.

For non-metastatic patients, standard-of-care radiotherapy (RT) was mandated for all non-metastatic and lymph node negative (N0M0) patients and encouraged for non-metastatic local lymph node positive (N+M0) patients (unless contraindicated) in combination with up to three years ADT.

A research comparison is defined by those patients allocated to the research arm, along with the corresponding contemporaneously randomised, eligible control arm patients. For the purposes of presentation, individually-powered comparisons are referred to as Phase 3 trials.

Table 1 STAMPEDE research comparisons

COMPARISON	ARMS	ELIGIBLE PATIENTS	ACCRUAL		END OF FOLLOW UP	TIME PERIOD(S)
			START DATE	END DATE		
"Zoledronic acid comparison"	A, B	All patients	05-Oct-2005	31-Mar-2013	12-Nov-2018	1-4
"Docetaxel comparison"	A, C	All patients	05-Oct-2005	31-Mar-2013	12-Nov-2018	1-4
"Celecoxib comparison"	A, D	All patients	05-Oct-2005	06-Apr-2011	12-Nov-2018	1
"Zoledronic acid + docetaxel comparison"	A, E	All patients	05-Oct-2005	31-Mar-2013	12-Nov-2018	1-4
"Zoledronic acid + celecoxib comparison"	A, F	All patients	05-Oct-2005	06-Apr-2011	12-Nov-2018	1
"Abiraterone comparison"	A, G	All patients	15-Nov-2011	17-Jan-2014	30-Nov-2021	3-5
"M1|RT comparison"	A, H	Newly-diagnosed M1 pts, no contraindication to RT	22-Jan-2013	02-Sep-2016	31-Nov-2020	4-9
"Enzalutamide + abiraterone comparison"	A, J	All patients	29-Jul-2014	31-Mar-2016	30-Nov-2021	7-9
"Metformin comparison"	A, K	Non-diabetic pts, no contraindication to metformin	05-Sep-2016	31-Mar-2023	TBD	10-TBD
"tE2 comparison"	A, L	<8wk anti-androgen use<br>Maximum 4wk LHRH therapy<br>No bilateral orchidectomy	20-Jun-2017	31-Mar-2023	TBD	11-TBD

1.4 BIOMARKER POPULATION: STRATOSPHERE STUDY

Retrieval of tissue blocks was initiated in May 2016 with transfer from treating hospitals to the Wales Cancer Bank. In October 2017, the Prostate Cancer UK Scientific Committee recommended funding for a Precision Medicine Award to perform molecular analyses on tissue collected from patients recruited to STAMPEDE between 2005 and 2014. In July 2018 the National Research Ethics Committee approved the following protocol covering molecular analysis of the samples: "The Stratosphere Consortium Molecular Landscape Study" (Reference: 18/LO/1235). Amendments have subsequently been approved to enable whole genome expression profiling of FFPE tissue with collaborators Veracyte (previously known as Decipher Biosciences) and for plasma-based PSA and testosterone testing. A workflow has been optimised to process the tissue for morphological, transcriptomic, and genomic analyses (Figure 2).

DocuSign Envelope ID: B9663506-7921-47F0-B9D8-557D7FE39808

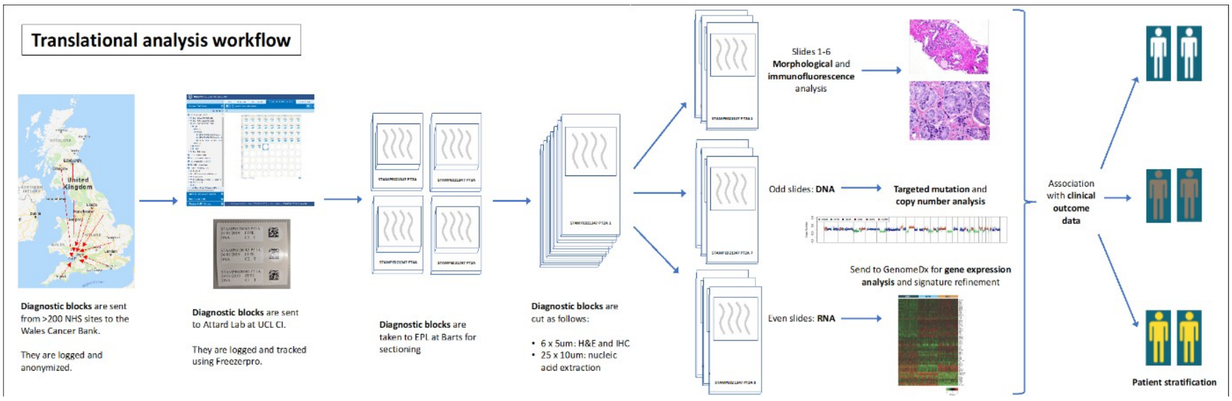

Figure 2 Diagram of the diagnostic block processing as part of STRATOSPHERE

1.5 JUSTIFICATION FOR GENE EXPRESSION SIGNATURE TESTING

Gene expression classifiers have shown prognostic utility in low- and intermediate-risk localised prostate cancer [15–19] but have not been clinically implemented in advanced prostate cancer. Transcriptome-wide profiling performed in the Veracyte (previously known as Decipher) clinical labs (in San Diego) is an analytically validated test that has a failure rate of ≤ 25% based on prior studies with similar aged formalin-fixed and paraffin-embedded tissue samples [17,20]. The failure rate to generate next generation sequencing data from DNA is typically much higher than this. Following a pre-specified analysis plan, we tested associations with survival of 59 multi-gene expression-based classifiers from patients randomized in the STAMPEDE abiraterone trial.

1.5.1 DECIPHER CLASSIFIER

In the analysis of gene expression data from the abiraterone comparison, the Decipher score classifier was the sole signature of those tested to be strongly prognostic ($p<2x10^{-5}$) in both metastatic and high-risk localised prostate cancer and identified clinically-relevant differences in absolute benefit, especially for localised cancers [1]. Analysis in CHAARTED also supports that selected transcriptomic signatures, including the Decipher score, are prognostic in metastatic patients treated with ADT or ADT with docetaxel [21]. The association of docetaxel with overall survival was observed across all Decipher score groups but the relative benefit of chemohormonal therapy appeared to vary by Decipher score group. The CHAARTED trial led us to hypothesise that the absolute benefit for overall survival with addition of docetaxel to ADT was higher for men with higher decipher score.

1.5.2 LUMINAL-BASAL SUB-TYPING

The PAM50 gene expression classifier, initially developed in breast cancer patients [22–24], is prognostic in metastatic prostate cancer but more intriguingly, in a relatively small sub-group of the CHAARTED trial, luminal B subtypes appeared to derive greater benefit from treatment with docetaxel and ADT as compared to basal subtypes [25]. Recently, Weiner *et al.* [26] reported on a novel prostate subtyping classifier (PSC) that similarly showed in the CHAARTED cohort tumours with luminal biology and a higher proliferative index (‘Luminal Proliferating’, LP) improved outcomes with chemohormonal therapy as compared to hormonal therapy alone. In addition, in an ancillary sub-study of the RTOG 0521 clinical trial Phillips *et al.* [27] have examined PSC in the context of high-risk localised disease treated with standard-of-care radiation and two years of androgen suppression with or without docetaxel. This analysis also supports LP subtype tumours as deriving greater benefit from the addition of docetaxel compared to non-LP tumours.

In both STAMPEDE and CHAARTED cohorts, luminal A subtypes (or alternative PSC luminal differentiated), which typically have a very good prognosis, were as anticipated much less prevalent in cohorts of advanced prostate cancer patients. The biomarker cohort of CHAARTED participants was

DocuSign Envelope ID: B9663506-7921-47F0-B9D8-557D7FE39808

160 and it was 183 for the RTOG 0521 cohort. Whilst hypothesis-generating, these studies have provided insufficient evidence to support prospective testing or clinical implementation. We therefore aim to provide more definitive evidence that patients classified as luminal B (or luminal proliferating) derive greater benefit from docetaxel. We calculate that despite access to tumours from ~400 metastatic patients with ~300 events, we will have insufficient power to formally test for a treatment interaction. However, we believe that together with emerging data from STAMPEDE and other cohorts (including CHAARTED, RTOG0521) we will provide the greatest possible evidence for differential treatment effect from docetaxel. This will inform on opportunities for clinical implementation and integration in future trials.

1.5.3 TRANSCRIPTOME-WIDE CLASSIFIERS OF BIOLOGICAL INTEREST

In exploratory analysis of gene expression data from patients randomised in the STAMPEDE abiraterone trial, we have found the prognostic association of a number of gene expression classifiers to differ across metastatic state (Figure 3). For example, interferon signalling alpha (IFNa_HM) showed the most statistically-significant association with worse outcome in high-risk localised disease [1]. PORTOS a gene signature associated with radiosensitivity was strongly associated with outcome in high-risk localised but not patients with metastatic disease. Gene expression signatures representative of PTEN or TP53 loss biology were significantly prognostic in metastatic but not non-metastatic disease. This suggests differential effects on outcome for distinct biological processes based on the presence or absence of metastatic disease on conventional scans at time of first presentation. We plan to confirm these observations in STAMPEDE patients randomised to docetaxel in two separate clinical trials of the STAMPEDE platform protocol (with minimal overlap of control patients). We also plan to combine gene expression data from patients randomised across abiraterone and docetaxel trials to test the associations with outcome of clinically-relevant classifiers.

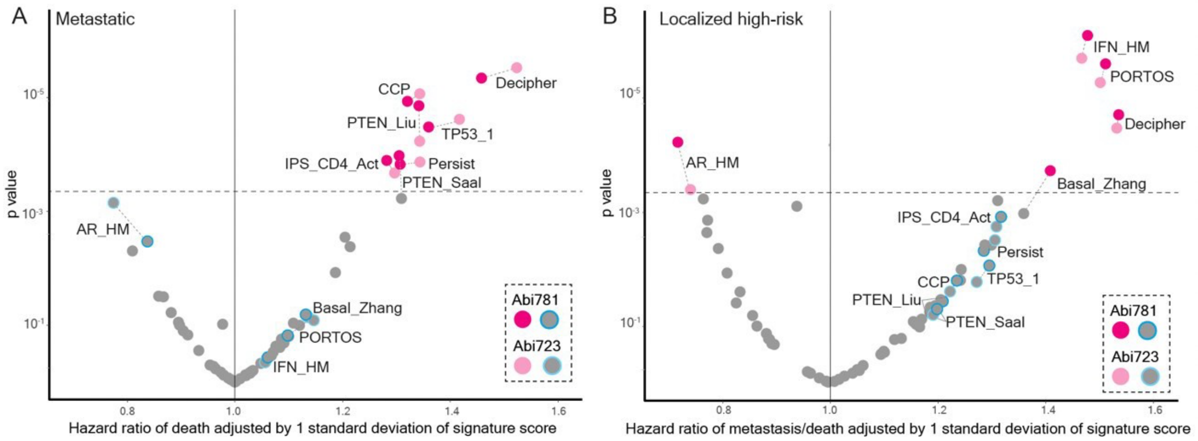

Figure 3 Scatter plot of hazard ratios (adjusted by one standard deviation of signature score) and p-values resulting from prognostic testing adjusted for clinical and pathological variables of overall survival in 57 signatures (with continuous scores) in patients with (A) metastatic disease and (B) locally advanced disease. Reproduced from Parry et al. [1]

1.6 JUSTIFICATION FOR KI-67 TESTING

High proliferation is recognized as a hallmark of neoplastic growth [28,29]. The most common methodology for assessment of proliferation involves the use of antibodies directed against the Ki-67

protein, a nuclear protein involved in cell cycle regulation, heterochromatin maintenance and assembly of the peri-chromosomal layers on mitotic chromosomes. It is expressed in all phases of the cell cycle other than the G0 phase [28]. This feature has made Ki-67 a clinically important biomarker for grading multiple types of cancers, with well-established prognostic value in several studies [30–36].

The proliferation index derived from the ratio of Ki-67 expressing tumour cells over the total of tumoural cells became the most studied immunohistochemistry marker in prostate cancer needle-biopsies [37–43]. Several studies found strong correlation with Gleason score in diagnostic biopsies [38,42,44] and radical prostatectomies [45–47]; others reported significant association with disease-free survival [41,48]; post-operative biochemical relapse [44,47,49,50], distant metastasis [49,51–53] and cancer-specific death [38,39,45,46,49,51,53,54] (Table 2). Ki-67 seems to be a robust prognostic biomarker but has not yet been incorporated in to the routine histopathological report as staining protocols are not uniform and there are no established cut-off values. The role of Ki-67 as a predictive biomarker is controversial but has been explored in breast cancer in the context of neoadjuvant chemo (NAC) in ER/PR positive disease where tumours with higher Ki-67 (at least 20% Ki-67 LI) seem to respond better to NAC [55–59].

We hypothesise that in a cohort of clinically advanced prostate cancer, Ki-67 scores will refine prognostication of men starting long-term ADT predicting early biochemical failure, progression and ultimately cancer-specific death. We hypothesise high Ki-67 scores will identify a subgroup of very-high risk prostate cancers with poorer outcomes that consequently will derive more benefit from therapy intensification. Furthermore, Ki-67-scores can be tested as a predictive biomarker. We hypothesise that on account of higher mitotic rates, patients with high Ki-67 scores will derive more benefit from spindle-cell toxins like docetaxel when compared to patients with very low Ki-67.

Table 2 Published reports of the prognostic value of Ki-67 in prostate cancer

Study author	Study type	Cohort treatment	Cohort	Cohort size	Tissue type	Scoring Methodology	Proposed cut-off (%)	Main positive findings
Kammerer-Jacquet (2019)	R (UK cancer registry)	untreated	localised PC	756	core bx	M	5.0%	CSD
Wilikins et al (2018)	R (case-control study)	RTx	Localised and locally advanced PC	173	core bx	M	continuous	BCR
Lobo et al (2018)	R	untreated	General population (consecutive)	189	core bx	DIA	5.3%	OS, CSD
Tretiakova et al (2016)	R	RP	Localised and locally advanced	1004	TMA (RP)	DIA	5.0%	OS, BCR, CSD
Tollefson et al (2014)	R	RP	General population (consecutive)	451	core bx	DIA	6.0%	CSD
Fisher et al (2013)	R (TAPG)	untreated	localised PC	293	TMA (core bx)	M	10.0%	CSD
Verhoven et al (2013)	R (trial RTOG94-08)	RTx vs RTx+STADT	locally advanced PC	468	core bx	M-DIA	6.2%	CSD, DM, BCR
Antonarakis et al (2012)	P (TAX2501 phase 2)	RP+adjuvant docetaxel	localised (high risk for progression)	57	TMA (RP)	DIA	continuous	PFS
Berney et al (2009)	R (TAPG)	RP	localised PC	693	TMA (TURP)	M-DIA	5.0%	OS
Khor et al (2009)	R (RTOG)	RTx+ADT	locally advanced PC	478	core bx and TURP	M-DIA	11.3%	OS, DM, CSD
Zellweger et al (2009)	P (consecutive series)	RP	localised PC	279	core bx	M	10.0%	BCR, EPE
Pollack et al (2003)	R (RTOG92-02)	LTADT+RTx vs STADT+RTx	locally advanced PC	537	core bx and TURP	M	7.1%	CSD, DM
Li et al (2003)	R (RTOG86-10)	RTx vs RTx + STADT	locally advanced PC	108	core bx and TURP	M	7.1%	DM, OS
Cowen et al (2002)	R	RTx	General population (consecutive)	106	core bx and TURP	M	3.5%	BCR, T3-T4 stage, GS 7-10

Notes: R: retrospective; RTx: radiotherapy; RP: radical prostatectomy; STADT: short-term ADT; LTADT: long-term ADT; ADT: androgen deprivation therapy; PC: prostate cancer; core bx: core biopsy; TMA: tissue microarray; TURP: transurethral resection of the prostate; M: manual (bright-field microscopy); DIA: digital image analysis; BCR: biochemical recurrence; OS: overall survival; CSD: cancer-specific death; DM: distant metastasis; PFS: progression free survival; EPE: extra-prostatic extension; GS: Gleason score

2 STUDY METHODS

2.1 ELIGIBILITY CRITERIA

STAMPEDE enrolled a broad population of participants unified by the need to start long-term ADT. All participants in STAMPEDE had to meet the following eligibility:

1. HIGH-RISK NEWLY DIAGNOSED NON-METASTATIC NODE-NEGATIVE DISEASE - Both:
   - At least two of: Stage T3/4, PSA ≥ 40 ng/ml or Gleason sum score 8-10
   - Intention to treat with radical radiotherapy (unless there is a contra-indication; exemption can be sought in advance of consent, after discussion with MRC CTU)

OR

2. NEWLY DIAGNOSED METASTATIC OR NODE-POSITIVE DISEASE - At least one of:
   - Stage T any N+ M0
   - Stage T any N any M+

OR

3. PREVIOUSLY TREATED WITH RADICAL SURGERY AND/OR RADIOTHERAPY, NOW RELAPSING - At least one of:
   - PSA ≥ 4 ng/ml and rising with doubling time less than 6 months
   - PSA ≥ 20 ng/ml
   - Lymph node positive
   - Metastatic disease

For inclusion into the gene expression translational ancillary study, STAMPEDE participants must meet the following **inclusion criteria**:

1. Enrolled into comparisons C (docetaxel), E (docetaxel + zoledronic acid) or G (abiraterone), including their concurrently randomised controls (arm A, see Table 1)
2. Have consented to participate in additional research
3. Have tissue from the primary tumour available for analyses

Participants must **not** meet any of the following **exclusion criteria**:

4. Tissue specimen collected >14 days after STAMPEDE randomisation

Gene expression data generated by Veracyte across additional clinically relevant cohorts recruited to randomised control trials may be used as outlined in this analysis plan, including CHAARTED and NRG Oncology/RTOG 0521.

2.2 RANDOMISATION

Patients were randomised centrally using a computerised algorithm developed and maintained by the CTU. Randomisation was performed using the method of minimisation over a number of clinically important stratification factors with an additional random element. These factors were:

Randomising centre	each centre
Metastases	M0 vs M1
Nodal involvement	N0 vs NX vs N+
Age at randomisation	up to 69yrs vs 70yrs and over
WHO performance status	PS=0 vs PS=1-2
Method of ADT[1]	Orchidectomy vs<br>LHRH agonist vs<br>LHRH antagonist vs<br>Dual Androgen Blockade (DAB)
Regular aspirin or NSAID use at baseline	yes vs no
Radiotherapy planned[2]	yes vs no

[1] Method of ADT options have changed over time, from LHRH vs orchidectomy, to then include bicalutamide, then specify LHRH agonist or antagonist and more recently exclude bicalutamide but include DAB

[2] "Radiotherapy planned" was added as a stratification factor at the start of recruitment to Efficacy Stage I for the "original comparisons" (Mar-2008)

When implementing the additional random element of the randomisation, an 80% probability of allocation was split between the (one or more) arms with the lowest strata totals (i.e. 80% probability of being allocated to one of the minimising arms); and the remaining 20% probability of allocation was split between the remaining (one or more) arms.

2.3 OUTCOMES

Full definitions of trial outcomes are included in Table 3. The primary endpoint in the present substudy is overall survival (OS). Failure-free survival (FFS), metastases-free survival (MFS), metastatic progression-free survival, and prostate cancer specific survival will be presented in secondary and exploratory analyses.

Table 3 Outcome measure definitions with censoring criteria

TERM	DEFINITION
Overall survival (OS)	Time from randomisation until date of death from any cause from a Death CRF (Form 12). For surviving patients, censor date 1 is used.<br>If record linkage to Civil Registrations of Death is successful, the registered date of death is used. For surviving patients, censor date 3 is used.
Metastases-Free Survival (MFS)	Time from randomisation until first of:<br>ï Radiologically-confirmed distant metastases<br>ï Death from any cause<br>For patients who have not had an event, censor date 2 is used (see below).
Failure-free Survival (FFS)	Time from randomisation until first of the following events:<br>ï Biochemical failure (as defined in protocol)<br>ï Local progression<br>ï Local lymph node progression<br>ï Distant metastases<br>ï Skeletal Related Event (where confirmed disease progression)<br>ï Death from prostate cancer<br>For patients who have not had an event, censor date 1 is used (see below).<br>If a suspicious event is reported for any of local progression, lymph node progression, distant metastases progression this will be counted as an FFS event.

DocuSign Envelope ID: B9663506-7921-47F0-B9D8-557D7FE39808

<table>
<tr><td>Metastatic Progression-Free Survival (MPFS)</td><td>Time from randomisation until first of:
ï Distant metastases
ï Skeletal-Related Event (where confirmed disease progression)
ï Death from prostate cancer
For patients who have not had an event, censor date is used (see below).
ï If a suspicious event is reported for distant metastases progression this will be counted as a MPFS event.
Censor date 1 is used.</td></tr>
<tr><td>Skeletal-Related Event (SRE)</td><td>ï Bone pain requiring radiotherapy and/or surgery
ï Pathological fracture
ï Metastatic spinal cord compression
Censor date 1 is used.</td></tr>
<tr><td>Prostate Cancer-Specific Survival (PCSS)</td><td>Time from randomisation until death from prostate cancer (see below).
For patients who have not had an event, censor date 1 is used.</td></tr>
<tr><td>Death from prostate cancer</td><td>An automated process assigns either PCa or non-PCa as cause of death using the following rules, based on information collected on CRFs:
<table>
<tr><th>Rule</th><th></th><th>Cause of death</th></tr>
<tr><td>1</td><td>Primary cause of death is PCa and no secondary causes are reported; progression event prior to death; no evidence of another cancer as an SAE</td><td>PCa</td></tr>
<tr><td>2</td><td>Primary cause of death is pneumonia and secondary cause of death is PCa; progression event prior to death</td><td>PCa</td></tr>
<tr><td>3</td><td>Primary cause of death is neutropenic sepsis and secondary cause of death is PCa; progression event prior to death</td><td>PCa</td></tr>
<tr><td>4</td><td>Primary cause of death is carcinomatosis and secondary cause of death is PCa; progression event prior to death</td><td>PCa</td></tr>
<tr><td>5</td><td>Death is reported as caused by PCa treatment; progression event prior to death</td><td>PCa</td></tr>
<tr><td>8</td><td>Site reports on Death CRF (version 13.0+) that responsible consultant considers death predominantly caused by PCa or protocol research / standard-of-care treatment</td><td>PCa</td></tr>
<tr><td>6</td><td>Primary cause of death is other primary cancer, and is confirmed by SAE report</td><td>Non-PCa</td></tr>
<tr><td>7</td><td>Primary cause of death is cardiovascular disease; PCa not listed as secondary cause of death</td><td>Non-PCa</td></tr>
<tr><td>9</td><td>Site reports on Death CRF (version 13.0+) that responsible consultant considers death predominantly caused by cardiovascular disease or other condition</td><td>Non-PCa</td></tr>
</table>
In participants successfully linked to Civil Registrations of Deaths, a death where the underlying cause is coded as any of the following ICD10 codes will be considered a PCa death:
- C61 Malignant neoplasm of prostate
- C77 Secondary and unspecified malignant neoplasm of lymph nodes
- C78 Secondary malignant neoplasm of respiratory and digestive organs
- C79 Secondary malignant neoplasm of other and unspecified sites
- C80 Malignant neoplasm, without specification of site.</td></tr>
</table>

Censor date 1	Date taken from the latest of the relevant variables defined below:<br>ï Date of randomisation (Form 1)<br>ï BMD assessment date (scan, blood sample, urine sample)<br>ï Date of treatment cycle (as taken from the bisphosphonate, docetaxel; Forms 4, 5, 6)<br>ï Date bloods taken (as taken from the bisphosphonate Forms 4, 5)<br>ï Date of last SOC docetaxel cycle (Form 21)<br>ï Date of any treatment action (Forms 7, 7B, 7C, 7D)<br>ï Date of any tE2 treatment action for Arm L patients (Form 25)<br>ï Date of tests recorded on hormone results log for Arm L patients (Form 24)<br>ï Dates reported on the Follow-up CRF (including date of PSA tests, date of any surgical interventions, date of any SRE, date of any metabolic or cardiovascular event; Forms 7, 7A)<br>ï Date of any reported progression event (Form 8)<br>ï Date additional treatment started or stopped (Forms 8, 8A)<br>ï Date of first/last RT fraction (Form 9A)<br>ï Date of late RT toxicity assessment (Form 10)<br>ï Date HT/research treatment ended (Form 11)<br>ï SAE date (onset, resolved, recent HT or trial treatment administration, start/end date of other treatment, test date) (Form 14)<br>ï Date of palliative RT fraction (Form 19)<br>ï Date blood or saliva sample obtained as reported on the pathology form (Form 18)<br>ï Date of co-enrolment to another trial (Form 15)<br>ï Date trial participation ended (Form 20)<br>ï Date last known alive (Form 7 from Version 13.0)<br>ï Date of death (Censoring date only for outcomes other than overall survival and disease-specific survival; Form 12)<br>Notes:<br>ï Dates from the QoL forms are no longer used as a censor date as these are completed by the patient and cannot be queried for errors.<br>ï Dates of form completion are no longer used as the CRF may have been completed retrospectively.<br>ï Any date pre-randomisation is ignored within the calculation.<br>Unusual dates which have not yet been resolved or dates after the ending of follow-up for the “abiraterone comparison” on 30-Nov-2021 will be ignored for the purposes of calculating this censor date.
Censor date 2 for MFS outcome measure	Date taken from the latest of the relevant variables defined below:<br>ï Date of randomisation (Form 1)<br>ï Date of assessment on the Follow-up CRF (unless recorded as a missed visit; Form 7)<br>ï Date of any reported progression event (If type of progression not included as event; Form 8)<br>Unusual dates which have not yet been resolved or dates after the ending of follow-up for the “abiraterone comparison” on 30-Nov-2021 will be ignored for the purposes of calculating this censor date.
Censor date 3	For patients successfully linked to Civil Registrations of Deaths, a censoring date will be set as 4 weeks before the data transfer, depending on what the data provider advises.

2.4 SAMPLE PROCESSING

The primary analyses described in this document will be performed on a single sample per patient. The minimum tumour cellularity for tumour-enriched area selection is 25% (with maximum benign cellularity of 15% in the selected region). To select a single sample from those with multiple tumour

DocuSign Envelope ID: B9663506-7921-47F0-B9D8-557D7FE39808

expression profiles, the following hierarchical selection criteria is used (moving to the next step of the hierarchy if there are ties in the criteria among multiple samples):

1. Sample(s) with the highest primary tissue Gleason pattern based on Decipher pathology review
2. Sample(s) with the highest secondary tissue Gleason pattern based on Decipher pathology review
3. Sample(s) with the longest tumour length (mm)
4. Sample(s) with the highest microarray percent positive probes above background

2.5 KI-67 SCORING AND GLEASON SCORE ASSESSMENT

All paraffin blocks with prostate needle core biopsies received at the translational site (UCL Cancer Institute) were processed at our histopathology core facility following a pre-defined protocol. Briefly, each block was sectioned at 3μm producing one section for haematoxylin and eosin (H&E) staining and 7 sequential sections for future immunohistochemical staining. Slides stained for H&E were used for assessment of tumour cellularity and re-assessment of Gleason score in keeping with up-to-date scoring methodology [60–64] (Table 4).

Sections were immunoassayed for Ki-67 using MIB-1 antibody, DAKO, Carpinteria, CA, USA, and attempted to reduce pre-analytical variables by following recommendations used in the assessment of Ki-67 in breast cancer following recommended methods and platforms [30]. Briefly, cells were scored in a semi-quantitative manner, by an expert prostatic pathologist, and the mean percentage of positive cells was estimated as the proportion of Ki-67 stained malignant nuclei over the total of malignant nuclei in the tumoural area, in a manner similar to that used in routine pathology departments for the assessment of proliferation index in other organs [30,64]. Corresponding haematoxylin and eosin slides were simultaneously reviewed. Normal tonsil was used as a positive control. This technique was designed to be robust for any pathology laboratory with experience in immunohistochemistry and has been published previously [37,39].

Table 4 Prostate Cancer Grading System (adapted from Epstein et al. [61])

Gleason Score	Grade group
GS ≤6	Grade group 1
GS 3+4=7	Grade group 2
GS 4+3=7, if (%) grade 3≥5%	Grade group 3
GS 4+4=8	Grade group 4
GS 4+3=7, if (%) grade 3<5%	
GS 3+5=8<br>GS 5+3=8	
GS 4+5=9	Grade group 5
GS 5+4=9	
GS 5+5=10	

GS: Gleason score

Ki-67 cell proliferation is scored semi-quantitatively, and recorded as multiples of 0.05 and indicate confidence that the true score is greater than or equal to the nominal value (eg 0.10), but strictly less than the next multiple of 0.05 (eg 0.15). For scores below 0.05, the pathologist may occasionally be confident that the proliferation score is strictly greater than 0, but under 0.05. In this event, an

intermediate score of 0.025 will be allocated to gain one additional semi-quantitative level in the dominant category.

Ki-67 scores by core will be provided to the MRC CTU by the UCL Cancer Institute and linked to the trial data by the Statistician. Quality and consistency checks will be performed to verify that Ki-67 scores have plausible values between 0 and 1 and that eligibility criteria are met. Problems will be queried with the UCL Cancer Institute.

2.6 PATIENT COHORT

A total of 2,369 UK patients were randomised to ADT (Arm A); ADT + docetaxel (Arm C); or ADT + docetaxel + zoledronic acid (Arm E) between 5 October 2005 and 31 March 2013 [8]. The Abiraterone comparison cohort randomised 1,917 patients from November 2011 through January 2014 [65]. Some participants (n=377) randomised to arm A between November 2013 and March 2013 act as control in both the Abiraterone comparison and the Docetaxel +/- Zoledronic acid comparison.

2.7 BIOMARKERS TO BE TESTED

Our primary predictive objectives (outlined explicitly in subsequent sections of this document) will test the association between the treatment effect of docetaxel and the following biomarkers (3 gene expression signatures and 1 immunohistochemistry assessment):

(1) **Decipher:** the commercially available Decipher test using 22 probe sets in a GLMnet model [18,66]. Decipher scores range from zero to one, with higher scores indicating a worse prognosis in localised prostate cancer [20]. Clinical Decipher test risk groups developed for localised prostate cancer are Low (<0.45), Intermediate (0.45-0.60), and High (>0.60). The Decipher signature was developed to determine the risk of metastases and therefore the majority of STAMPEDE tumours have a High risk (>0.6) Decipher score.
(2) **PAM50**: A basal/luminal subtyping model which was originally developed in breast cancer, that has prognostic utility in breast, bladder and prostate cancer [15,23,67]. Cases are split into basal, luminal A or luminal B subtypes.
(3) **PSC:** A recently developed prostate specific 'PAM50 version 2' model [68]. A multinomial subtyping classifier that predicts one of four cancer subtypes: basal immune (BI), basal neuroendocrine-like (BN), luminal differentiated (LD), and luminal proliferating (LP).
(4) **Ki-67:** the semi-quantitative immunohistochemistry assessment of Ki-67 protein expression will be used as a continuous variable in the analysis.

A further 56 gene expression signatures from the Genomics Resource Information Database (GRID; ClinicalTrials.gov reference: NCT02609269) will be included in exploratory analyses and are listed in Appendix 3.

All signatures except for Decipher quantile-normalised using the subset of the Decipher GRID cohort (~132,874 samples) classified as NCCN very high risk (~2,399 samples) as the reference population.

2.8 DISEASE VOLUME ASSESSMENT

M1 patients will be sub-classified according to metastatic volume, i.e. high or low volume, as determined by the CHAARTED definition [9], where high volume disease is defined as: four or more bone metastases on bone scan, including one or more outside the vertebral bodies or pelvis, and/or visceral metastases. These data will be incorporated into the analysis where specified using a finalised

DocuSign Envelope ID: B9663506-7921-47F0-B9D8-557D7FE39808

copy of the volume classification dataset saved by the MRC CTU. The methodology for this classification was described previously.

2.9 TIMING OF ANDROGEN DEPRIVATION

Androgen deprivation affects transcriptional activity, which may moderate the prognostic effect of transcriptomic or proteomic signatures. The study will conduct a sensitivity analysis excluding data points from specimens collected after start of LHRHa or anti-androgens. For this, STAMPEDE CRFs 1 (randomisation), 2 (baseline), and 7 (treatment log) will be used to determine the earliest date of exposure to androgen deprivation therapy, whether it is orchiectomy, luteinising hormone releasing hormone agonists/antagonists, and/or androgen receptor antagonists (eg bicalutamide to mitigate short-term testosterone flare or as maximum androgen blockage). The biopsy date (obtained from local pathology forms when available or from trial CRFs when not available) will be compared with the date of androgen deprivation therapy. Sensitivity analyses will be performed only on patients whose biopsy was performed at the most 1 day after orchiectomy or initiation of LHRH antagonists, or anti-androgens (bicalutamide, flutamide). Patients will be kept in the dataset if the biopsy occured at the most 5 days after starting LHRH agonists, to take into account the longer time to castration with LHRH agonists.

2.10 TRIAL DATA LOCK AND VERIFICATION

At the time of analysis:

- The Statistician will extract from Macro a dataset of all data stored in the database. This will act as the frozen dataset. It is the responsibility of the Statistician to accurately record the date of freezing and ensure all data is retrieved.
- New data can continue to be entered onto Macro database.
- If any outstanding data queries are resolved during the analysis that relate to data in the frozen dataset (e.g. problems that are found during analysis or amended CRFs that are returned to CTU), the main Macro database should be changed under the oversight of the Trial Manager.

Data verification, consistency and range checks will have been performed at the data entry stage by the MRC CTU, as well as checks for missing data (copies can be found in the Trial Master File). Additional range, consistency and missing data checks will be performed, as appropriate, when the analysis is performed (and when the datasets for analysis are constructed). All variables will be examined for unusual, outlying, unlabelled or inconsistent values.

Given the thorough nature of our follow-up procedure we expect the issue of missing data to be relatively minimal. We anticipate high compliance with initial data collection as this is close to the time of patient registration. If any data is missing imputation will not be done. Any problems with trial data will be queried with the Trial Managers, Data Managers, or statisticians, as appropriate. If possible, data queries will be resolved, although it is accepted that due to administrative reasons and data availability a small number of problems will continue to exist. This will be minimised.

DocuSign Envelope ID: B9663506-7921-47F0-B9D8-557D7FE39808

3 STATISTICAL PRINCIPLES

3.1 AIMS

This analysis aims to investigate:
(1) the prognostic effect of 59 signatures on OS in locally advanced patients, depending on systemic treatment allocation (ADT only, ADT + abiraterone, ADT + docetaxel)
(2) the prognostic effect of 59 signatures on OS in metastatic patients, depending on systemic treatment allocation (ADT only, ADT + abiraterone, ADT + docetaxel)
(3) whether the prognostic effect of PORTOS and interferon alpha signatures on OS is different depending on metastatic status in patients commencing long-term ADT
(4) whether the Decipher score predicts the efficacy of docetaxel in metastatic and locally advanced patients commencing long-term ADT
(5) whether the efficacy of docetaxel in metastatic and locally advanced patients commencing long-term ADT is greater in luminal proliferating subtypes compared to basal immune subtypes based on the PSC signature
(6) whether the efficacy of docetaxel in metastatic and locally advanced patients commencing long-term ADT is greater in luminal B subtypes compared to basal subtypes based on the PAM50 signature
(7) whether the immunohistochemistry Ki-67 score predicts the efficacy of docetaxel in metastatic and locally advanced patients commencing long-term ADT.

3.2 OBJECTIVES

3.2.1 PROGNOSTIC EFFECT OF 59 GENE SIGNATURES IN THE ABIRATERONE AND DOCETAXEL TRIAL COMPARISONS

The prognostic effect of 59 gene signatures (see Appendix 3) on OS will be evaluated in 8 cohorts:

- M0 patients in all arms combined
- M0 patients allocated to arm A (ADT only)
- M0 patients allocated to arms C or E (ADT + docetaxel +/- zoledronic acid)
- M0 patients allocated to arm G (ADT + abiraterone)
- M1 patients in all arms combined
- M1 patients allocated to arm A (ADT only)
- M1 patients allocated to arms C or E (ADT + docetaxel +/- zoledronic acid)
- M1 patients allocated to arm G (ADT + abiraterone)

Univariable and multivariable Cox Proportional Hazards models will be fitted as described in section 3.3. Continuous signatures will be standardised to provide more comparable effect sizes: they will be divided by the standard deviation estimated from the complete biomarker cohort. In the event of nonproportional hazards, other transformations or dichotomisation may be considered. Bimodal signatures will be converted to a categorical variable using the median for the M1 docetaxel and abiraterone cohort as a threshold.

For Kaplan-Meier curves, continuous signatures will be split using the median for the M1 docetaxel and abiraterone cohort as a threshold.

The subset of signatures with statistically significant associations with OS at the 95% confidence level will be evaluated across secondary endpoints (see Table 3) in the following cohorts:

- M0 patients in all arms combined
- M1 patients in all arms combined.

3.2.2 PREDICTIVE EFFECT OF BASAL-LUMINAL SUBTYPING (METASTATIC DISEASE)

We will test the evidence of an interaction between luminal-basal subtyping and addition of docetaxel for metastatic prostate cancer patients in relation to OS. Comparisons AC and AE (see Table 1) will be combined into a Cox proportional hazards regression model with:

- a binary variable indicating allocation to SOC + docetaxel (+/- zoledronic acid)
- a binary variable for the basal-luminal subtype.
- covariate adjustments and hazard stratification as set out in section 3.3

PAM50 classes that have not been quantile-normalised will be associated with clinical outcome to evaluate the hypothesis-generating data from the CHAARTED trial [21]. The potential utility of quantile-matched PAM50 classes is uncertain and will be associated with clinical outcome in exploratory analyses outlined in section 3.2.5.

A two-sided partial likelihood ratio test will evaluate the addition of an interaction term between the basal-luminal variable and addition of docetaxel. The hypothesis will be tested at the 5% significance level. Subgroup-specific hazard ratios and 95% confidence intervals will be reported as relative treatment effect measures.

This approach will be used to test:

1. the PAM50 classifier, where the reference category will be basal subtypes, hypothesising the luminal B subtype derives benefit. Luminal A types will be excluded given good prognosis and under-representation in advanced disease.
2. the PSC classifier, where the reference category will be basal immune, compared with luminal proliferating, hypothesising the luminal proliferating subtype derives benefit. The other two types will be excluded. The quantile-normalised PSC classifier will be used in this analysis as we seek to validate the direction of association between luminal proliferating subtypes and clinical outcome published by Weiner et al. [26] in the CHAARTED trial.

Given the limited power to detect even strong effects (see appendix 2), data will be reported in greater detail provided that (a) the likelihood ratio test p-value falls below a more liberal threshold of 0.10 (10% significance level); and (b) the direction of effect matches that hypothesised in section 3.1. In that event, hazard ratios or RMST differences will also be estimated for secondary endpoints to provide a more complete characterisation of the moderation observed by basal-luminal subtypes.

3.2.3 PREDICTIVE EFFECT OF BASAL-LUMINAL SUBTYPING (LOCALISED DISEASE)

In the event the likelihood ratio test significance for PAM50 and/or PSC is below 0.1 in the STAMPEDE M1 cohort, and the direction of effect is congruent with that hypothesised in section 3.1, the same analyses will be conducted in the localised disease (M0) cohort, which is smaller and has lower statistical power (see appendix 3). We will also compare to the analysis using the same parameters performed in the phase III NRG Oncology/RTOG 0521 trial [71] and consider a meta-analysis.

3.2.4 PREDICTIVE EFFECT OF DECIPHER AND KI-67

The effect of Decipher and Ki-67 on OS cohorts will be tested in M0 and M1 cohort separately. We hypothesise that the higher the Decipher score or Ki67 score, the greater the benefit of Docetaxel. Comparisons AC and AE (see Table 1) will be combined into a Cox proportional hazards regression model with:

- a binary variable indicating allocation to SOC + docetaxel (+/- zoledronic acid)
- a continuous variable for the score
- covariate adjustments and hazard stratification as set out in section 3.3.

A two-sided partial likelihood ratio test will evaluate the addition of an interaction term between the biomarker of interest and addition of docetaxel. The hypothesis will be tested at the 5% significance level.

In addition, the predictive effect will be examined for dichotomised transformations of each factor, using the median in the metastatic subgroup (from the M1 ACEG biomarker cohort) as a threshold. Comparisons AC and AE will be combined into a Cox proportional hazards regression model with:

- a binary variable indicating allocation to SOC + docetaxel (+/- zoledronic acid)
- a binary variable for the score (<= M1 median value; > M1 median value)
- covariate adjustments and hazard stratification as set out in section 3.3.

A two-sided partial likelihood ratio test will evaluate the addition of an interaction term between the biomarker of interest and addition of docetaxel. Subgroup-specific hazard ratios for the treatment effect of docetaxel and 95% confidence intervals will be reported.

Similar estimates will be produced for secondary endpoints listed in section 2.3 if (a) the likelihood ratio test p-value falls below a more liberal threshold of 0.10 (10% significance level); and (b) the direction of effect matches that hypothesised.

3.2.5 EXPLORATORY OBJECTIVES

The overarching aim of this exploratory work is to identify novel prognostic and predictive biomarkers in advanced prostate cancer patients commencing long-term ADT. To achieve this, STAMPEDE cohorts A/C/E and A/G [1] may be combined to increase our power to detect potentially clinically meaningful transcriptome classifiers. Additional randomised control trial cohorts may be leveraged to validate discoveries as they become available or to incorporate into meta-analyses, including CHAARTED [9] and NRG RTOG 0521 [71].

Exploratory Objective 1: Determine the clinical utility of multi-modal data including additional GRID signatures, morphological features and IHC data representative of distinct biological processes in the ACE and AG cohorts. Secondary trial endpoints may be included if an association with OS is observed

Exploratory Objective 2: Test for differential treatment effect with ADT +/- docetaxel or ADT +/- abiraterone for the gene expression signatures of interest in M1 cohorts and separately in M0 cohorts

Exploratory Objective 3: Evaluate the heterogeneity in GRID signature assignment in cases where multiple tissue samples are available.

3.3 STATISTICAL MODELLING

Hypotheses will be tested in models adjusted for the following adjustment variables: age at randomization (years, continuous), WHO performance status (0 vs 1-2), regular aspirin or NSAID use (no vs yes), serum PSA (prior to start of ADT and within 6 months of randomisation, continuous, log-transformed), Gleason score as assessed by the local pathologist (<=6; 7; >=8), tumour stage (T0-T2; T3; T4), nodal stage and in M1 cases metastasis volume categorisation (low volume vs high volume as per CHAARTED definition). Metastasis volume is affected by data missingness, which will be investigated. In the event evidence show data are not missing completely at random, multiple imputation with chained equations will be used to address any risk of bias. Missing Gleason scores will be imputed using the dominant category. Baseline hazards will be stratified by periods of randomisation as described in Appendix 1. Univariable and multivariable hazard ratios will be reported.

Evidence of nonproportional hazards by trial arm and signature will be assessed in a Grambsch-Therneau test and graphical inspection of Kaplan-Meier curves. In the event of nonproportional hazards, restricted mean survival time (RMST) difference will be emphasised as the main treatment effect measure. The difference in **5-year** RMSTD between categories will be estimated under a bias-corrected bootstrap, with the expectation that this difference should be positive if in patients with a score greater than the median compared to patients with a score lower than the median, in absolute terms.

3.4 SENSITIVITY ANALYSES

Sensitivity analyses will examine the robustness of findings against gene expression measurement error caused by the initiation of androgen deprivation. Primary analyses will be repeated in the subset of participants whose therapy began after collection of the biopsy specimen, and model coefficients will be compared with the main analysis to detect changes in effect sizes, such as attenuation.

REFERENCES

[1] Parry M, Grist E, Mendes L, Dutey-Magni P, Sachdeva A, Brawley C, et al. Clinical testing of transcriptome-wide expression profiles in high-risk localized and metastatic prostate cancer starting androgen deprivation therapy: an ancillary study of the STAMPEDE abiraterone Phase 3 trial. Res Sq 2023. https://doi.org/10.21203/rs.3.rs-2488586/v1.

[2] James ND, de Bono JS, Spears MR, Clarke NW, Mason MD, Dearnaley DP, et al. Abiraterone for Prostate Cancer Not Previously Treated with Hormone Therapy. N Engl J Med 2017;377:338–51. https://doi.org/10.1056/nejmoa1702900.

[3] Attard G, Murphy L, Clarke NW, Cross W, Jones RJ, Parker CC, et al. Abiraterone acetate and prednisolone with or without enzalutamide for high-risk non-metastatic prostate cancer: a meta-analysis of primary results from two randomised controlled phase 3 trials of the STAMPEDE platform protocol. Lancet 2022;399:447–60. https://doi.org/10.1016/S0140-6736(21)02437-5.

[4] Smith MR, Hussain M, Saad F, Fizazi K, Sternberg CN, Crawford ED, et al. Darolutamide and Survival in Metastatic, Hormone-Sensitive Prostate Cancer. N Engl J Med 2022;386:1132–42. https://doi.org/10.1056/NEJMoa2119115.

[5] Fizazi K, Tran N, Fein L, Matsubara N, Rodriguez-Antolin A, Alekseev BY, et al. Abiraterone plus Prednisone in Metastatic, Castration-Sensitive Prostate Cancer. N Engl J Med 2017;377:352–60. https://doi.org/10.1056/NEJMoa1704174.

[6] Chi KN, Agarwal N, Bjartell A, Chung BH, Pereira de Santana Gomes AJ, Given R, et al. Apalutamide for Metastatic, Castration-Sensitive Prostate Cancer. N Engl J Med 2019;381:13–24. https://doi.org/10.1056/NEJMoa1903307.

[7] Davis ID, Martin AJ, Stockler MR, Begbie S, Chi KN, Chowdhury S, et al. Enzalutamide with Standard First-Line Therapy in Metastatic Prostate Cancer. N Engl J Med 2019;381:121–31. https://doi.org/10.1056/NEJMoa1903835.

[8] James ND, Sydes MR, Clarke NW, Mason MD, Dearnaley DP, Spears MR, et al. Addition of docetaxel, zoledronic acid, or both to first-line long-term hormone therapy in prostate cancer (STAMPEDE): Survival results from an adaptive, multiarm, multistage, platform randomised controlled trial. Lancet 2016;387:1163–77. https://doi.org/10.1016/S0140-6736(15)01037-5.

[9] Sweeney CJ, Chen Y-H, Carducci M, Liu G, Jarrard DF, Eisenberger M, et al. Chemohormonal Therapy in Metastatic Hormone-Sensitive Prostate Cancer. N Engl J Med 2015;373:737–46. https://doi.org/10.1056/NEJMoa1503747.

[10] James ND, Ingleby FC, Clarke NW, Amos CL, Attard G, Brawley CD, et al. Docetaxel for Nonmetastatic Prostate Cancer: Long-Term Survival Outcomes in the STAMPEDE Randomized Controlled Trial. JNCI Cancer Spectr 2022;6:1–10. https://doi.org/10.1093/jncics/pkac043.

[11] Fizazi K, Foulon S, Carles J, Roubaud G, McDermott R, Fléchon A, et al. Abiraterone plus prednisone added to androgen deprivation therapy and docetaxel in de novo metastatic castration-sensitive prostate cancer (PEACE-1): a multicentre, open-label, randomised, phase 3 study with a 2 × 2 factorial design. Lancet 2022;399:1695–707. https://doi.org/10.1016/S0140-6736(22)00367-1.

[12] Vale CL, Fisher D, Godolphin P, Rydzewska LH, Boher J-M, Burdett S, et al. Defining more precisely the effects of docetaxel plus ADT for men with mHSPC: Meta-analysis of individual participant data from randomized trials. J Clin Oncol 2022;40:5070–5070. https://doi.org/10.1200/JCO.2022.40.16_suppl.5070.

[13] Grist E, Friedrich S, Brawley C, Mendes L, Parry M, Ali A, et al. Accumulation of copy number alterations and clinical progression across advanced prostate cancer. Genome Med 2022;14:102. https://doi.org/10.1186/s13073-022-01080-4.

[14] Parry M, Grist E, Brawley C, Proudfoot JA, Mendes L, Lall S, et al. 1358O Clinical qualification of transcriptome signatures for advanced prostate cancer (APC) starting androgen deprivation therapy (ADT) with or without abiraterone acetate and prednisolone (AAP): An

DocuSign Envelope ID: B9663506-7921-47F0-B9D8-557D7FE39808

ancillary study of the STAMPEDE AAP trial. Ann Oncol 2022;33:S1161. https://doi.org/10.1016/j.annonc.2022.07.1491.

[15] Zhao SG, Chang SL, Erho N, Yu M, Lehrer J, Alshalalfa M, et al. Associations of Luminal and Basal Subtyping of Prostate Cancer With Prognosis and Response to Androgen Deprivation Therapy. JAMA Oncol 2017;3:1663–72. https://doi.org/10.1001/jamaoncol.2017.0751.

[16] Spratt DE, Alshalalfa M, Fishbane N, Weiner AB, Mehra R, Mahal BA, et al. Transcriptomic Heterogeneity of Androgen Receptor Activity Defines a de novo low AR-Active Subclass in Treatment Naïve Primary Prostate Cancer. Clin Cancer Res an Off J Am Assoc Cancer Res 2019;25:6721–30. https://doi.org/10.1158/1078-0432.CCR-19-1587.

[17] Erho N, Crisan A, Vergara IA, Mitra AP, Ghadessi M, Buerki C, et al. Discovery and Validation of a Prostate Cancer Genomic Classifier that Predicts Early Metastasis Following Radical Prostatectomy. PLoS One 2013;8:e66855. https://doi.org/10.1371/journal.pone.0066855.

[18] Dal Pra A, Ghadjar P, Hayoz S, Spratt DE, Liu VYT, Todorovic T, et al. Validation of the decipher genomic classifier (GC) in SAKK 09/10: A phase III randomized trial of dose-escalated salvage radiotherapy (SRT) after radical prostatectomy (RP). J Clin Oncol 2021;39:5010. https://doi.org/10.1200/JCO.2021.39.15_suppl.5010.

[19] Zhao SG, Chang SL, Spratt DE, Erho N, Yu M, Ashab HA-D, et al. Development and validation of a 24-gene predictor of response to postoperative radiotherapy in prostate cancer: a matched, retrospective analysis. Lancet Oncol 2016;17:1612–20. https://doi.org/10.1016/S1470-2045(16)30491-0.

[20] Jairath NK, Dal Pra A, Vince R, Dess RT, Jackson WC, Tosoian JJ, et al. A Systematic Review of the Evidence for the Decipher Genomic Classifier in Prostate Cancer. Eur Urol 2021;79:374–83. https://doi.org/10.1016/j.eururo.2020.11.021.

[21] Hamid AA, Huang HC, Wang V, Chen YH, Feng F, Den R, et al. Transcriptional profiling of primary prostate tumor in metastatic hormone-sensitive prostate cancer and association with clinical outcomes: correlative analysis of the E3805 CHAARTED trial. Ann Oncol 2021;32:1157–66. https://doi.org/10.1016/j.annonc.2021.06.003.

[22] Rueda OM, Sammut S-J, Seoane JA, Chin S-F, Caswell-Jin JL, Callari M, et al. Dynamics of breast-cancer relapse reveal late-recurring ER-positive genomic subgroups. Nature 2019;567:399–404. https://doi.org/10.1038/s41586-019-1007-8.

[23] Sørlie T, Perou CM, Tibshirani R, Aas T, Geisler S, Johnsen H, et al. Gene expression patterns of breast carcinomas distinguish tumor subclasses with clinical implications. Proc Natl Acad Sci 2001;98:10869–74. https://doi.org/10.1073/pnas.191367098.

[24] Parker JS, Mullins M, Cheang MCU, Leung S, Voduc D, Vickery T, et al. Supervised Risk Predictor of Breast Cancer Based on Intrinsic Subtypes. J Clin Oncol 2009;27:1160–7. https://doi.org/10.1200/JCO.2008.18.1370.

[25] Hamid AA, Gray KP, Shaw G, MacConaill LE, Evan C, Bernard B, et al. Compound Genomic Alterations of TP53, PTEN, and RB1 Tumor Suppressors in Localized and Metastatic Prostate Cancer. Eur Urol 2019;76:89–97. https://doi.org/10.1016/j.eururo.2018.11.045.

[26] Weiner AB, Liu Y, Hakansson A, Zhao X, Proudfoot JA, Ho J, et al. A novel prostate cancer subtyping classifier based on luminal and basal phenotypes. Cancer 2023. https://doi.org/10.1002/cncr.34790.

[27] Phillips R, Proudfoot J, Davicioni E, Liu Y, Spratt D, Feng F, et al. Basal-luminal subtyping of localized high-risk prostate cancer identifies benefit from adding docetaxel to definitive radiotherapy with androgen suppression in the NRG Oncology/RTOG 0521 phase III trial. Abstract 5094. 2023 ASCO Annu. Meet., 2023.

[28] Sobecki M, Mrouj K, Colinge J, Gerbe F, Jay P, Krasinska L, et al. Cell-Cycle Regulation Accounts for Variability in Ki-67 Expression Levels. Cancer Res 2017;77:2722–34. https://doi.org/10.1158/0008-5472.CAN-16-0707.

[29] Sun X, Kaufman PD. Ki-67: more than a proliferation marker. Chromosoma 2018;127:175–86. https://doi.org/10.1007/s00412-018-0659-8.

[30] Dowsett M, Nielsen TO, A'Hern R, Bartlett J, Coombes RC, Cuzick J, et al. Assessment of Ki67

DocuSign Envelope ID: B9663506-7921-47F0-B9D8-557D7FE39808

in breast cancer: recommendations from the International Ki67 in Breast Cancer working group. J Natl Cancer Inst 2011;103:1656–64. https://doi.org/10.1093/jnci/djr393.
[31] Nadler A, Cukier M, Rowsell C, Kamali S, Feinberg Y, Singh S, et al. Ki-67 is a reliable pathological grading marker for neuroendocrine tumors. Virchows Arch 2013;462:501–5. https://doi.org/10.1007/s00428-013-1410-8.
[32] Hashmi AA, Iftikhar SN, Nargus G, Ahmed O, Asghar IA, Shirazi UA, et al. Ki67 Proliferation Index in Germinal and Non-Germinal Subtypes of Diffuse Large B-Cell Lymphoma. Cureus 2021;13:e13120. https://doi.org/10.7759/cureus.13120.
[33] Luo Y, Ren F, Liu Y, Shi Z, Tan Z, Xiong H, et al. Clinicopathological and prognostic significance of high Ki-67 labeling index in hepatocellular carcinoma patients: a meta-analysis. Int J Clin Exp Med 2015;8:10235–47.
[34] Pezzilli R, Partelli S, Cannizzaro R, Pagano N, Crippa S, Pagnanelli M, et al. Ki-67 prognostic and therapeutic decision driven marker for pancreatic neuroendocrine neoplasms (PNENs): A systematic review. Adv Med Sci 2016;61:147–53. https://doi.org/10.1016/j.advms.2015.10.001.
[35] Pyo J-S, Kang G, Sohn JH. Ki-67 labeling index can be used as a prognostic marker in gastrointestinal stromal tumor: a systematic review and meta-analysis. Int J Biol Markers 2016;31:e204-10. https://doi.org/10.5301/jbm.5000183.
[36] Richards-Taylor S, Ewings SM, Jaynes E, Tilley C, Ellis SG, Armstrong T, et al. The assessment of Ki-67 as a prognostic marker in neuroendocrine tumours: a systematic review and meta-analysis. J Clin Pathol 2016;69:612–8. https://doi.org/10.1136/jclinpath-2015-203340.
[37] Berney DM, Gopalan A, Kudahetti S, Fisher G, Ambroisine L, Foster CS, et al. Ki-67 and outcome in clinically localised prostate cancer: analysis of conservatively treated prostate cancer patients from the Trans-Atlantic Prostate Group study. Br J Cancer 2009;100:888–93. https://doi.org/10.1038/sj.bjc.6604951.
[38] Fisher G, Yang ZH, Kudahetti S, Møller H, Scardino P, Cuzick J, et al. Prognostic value of Ki-67 for prostate cancer death in a conservatively managed cohort. Br J Cancer 2013;108:271–7. https://doi.org/10.1038/bjc.2012.598.
[39] Kammerer-Jacquet S-F, Ahmad A, Møller H, Sandu H, Scardino P, Soosay G, et al. Ki-67 is an independent predictor of prostate cancer death in routine needle biopsy samples: proving utility for routine assessments. Mod Pathol 2019;32:1303–9. https://doi.org/10.1038/s41379-019-0268-y.
[40] Mesko S, Kupelian P, Demanes DJ, Huang J, Wang P-C, Kamrava M. Quantifying the Ki-67 heterogeneity profile in prostate cancer. Prostate Cancer 2013;2013:717080. https://doi.org/10.1155/2013/717080.
[41] Rubio J, Ramos D, López-Guerrero JA, Iborra I, Collado A, Solsona E, et al. Immunohistochemical expression of Ki-67 antigen, cox-2 and Bax/Bcl-2 in prostate cancer; prognostic value in biopsies and radical prostatectomy specimens. Eur Urol 2005;48:745–51. https://doi.org/10.1016/j.eururo.2005.06.014.
[42] Tolonen TT, Tammela TLJ, Kujala PM, Tuominen VJ, Isola JJ, Visakorpi T. Histopathological variables and biomarkers enhancer of zeste homologue 2, Ki-67 and minichromosome maintenance protein 7 as prognosticators in primarily endocrine-treated prostate cancer. BJU Int 2011;108:1430–8. https://doi.org/10.1111/j.1464-410X.2011.10253.x.
[43] Laitinen S, Martikainen PM, Tolonen T, Isola J, Tammela TLJ, Visakorpi T. EZH2, Ki-67 and MCM7 are prognostic markers in prostatectomy treated patients. Int J Cancer 2008;122:595–602. https://doi.org/10.1002/ijc.23145.
[44] Cowen D, Troncoso P, Khoo VS, Zagars GK, von Eschenbach AC, Meistrich ML, et al. Ki-67 staining is an independent correlate of biochemical failure in prostate cancer treated with radiotherapy. Clin Cancer Res 2002;8:1148–54.
[45] Tollefson MK, Karnes RJ, Kwon ED, Lohse CM, Rangel LJ, Mynderse LA, et al. Prostate cancer Ki-67 (MIB-1) expression, perineural invasion, and gleason score as biopsy-based predictors of prostate cancer mortality: the Mayo model. Mayo Clin Proc 2014;89:308–18.

DocuSign Envelope ID: B9663506-7921-47F0-B9D8-557D7FE39808

https://doi.org/10.1016/j.mayocp.2013.12.001.

[46] Tretiakova MS, Wei W, Boyer HD, Newcomb LF, Hawley S, Auman H, et al. Prognostic value of Ki67 in localized prostate carcinoma: A multi-institutional study of >1000 prostatectomies. Prostate Cancer Prostatic Dis 2016;19:264–70. https://doi.org/10.1038/pcan.2016.12.

[47] Zellweger T, Günther S, Zlobec I, Savic S, Sauter G, Moch H, et al. Tumour growth fraction measured by immunohistochemical staining of Ki67 is an independent prognostic factor in preoperative prostate biopsies with small-volume or low-grade prostate cancer. Int J Cancer 2009;124:2116–23. https://doi.org/10.1002/ijc.24174.

[48] Antonarakis ES, Keizman D, Zhang Z, Gurel B, Lotan TL, Hicks JL, et al. An immunohistochemical signature comprising PTEN, MYC, and Ki67 predicts progression in prostate cancer patients receiving adjuvant docetaxel after prostatectomy. Cancer 2012;118:6063–71. https://doi.org/10.1002/cncr.27689.

[49] Verhoven B, Yan Y, Ritter M, Khor L-Y, Hammond E, Jones C, et al. Ki-67 is an independent predictor of metastasis and cause-specific mortality for prostate cancer patients treated on Radiation Therapy Oncology Group (RTOG) 94-08. Int J Radiat Oncol Biol Phys 2013;86:317–23. https://doi.org/10.1016/j.ijrobp.2013.01.016.

[50] Wilkins AC, Gusterson B, Szijgyarto Z, Haviland J, Griffin C, Stuttle C, et al. Ki67 Is an Independent Predictor of Recurrence in the Largest Randomized Trial of 3 Radiation Fractionation Schedules in Localized Prostate Cancer. Int J Radiat Oncol Biol Phys 2018;101:309–15. https://doi.org/10.1016/j.ijrobp.2018.01.072.

[51] Khor L-Y, Bae K, Paulus R, Al-Saleem T, Hammond ME, Grignon DJ, et al. MDM2 and Ki-67 predict for distant metastasis and mortality in men treated with radiotherapy and androgen deprivation for prostate cancer: RTOG 92-02. J Clin Oncol 2009;27:3177–84. https://doi.org/10.1200/JCO.2008.19.8267.

[52] Li R, Heydon K, Hammond ME, Grignon DJ, Roach M, Wolkov HB, et al. Ki-67 staining index predicts distant metastasis and survival in locally advanced prostate cancer treated with radiotherapy: an analysis of patients in radiation therapy oncology group protocol 86-10. Clin Cancer Res 2004;10:4118–24. https://doi.org/10.1158/1078-0432.CCR-1052-03.

[53] Pollack A, Cowen D, Troncoso P, Zagars GK, von Eschenbach AC, Meistrich ML, et al. Molecular markers of outcome after radiotherapy in patients with prostate carcinoma: Ki-67, bcl-2, bax, and bcl-x. Cancer 2003;97:1630–8. https://doi.org/10.1002/cncr.11230.

[54] Lobo J, Rodrigues Â, Antunes L, Graça I, Ramalho-Carvalho J, Vieira FQ, et al. High immunoexpression of Ki67, EZH2, and SMYD3 in diagnostic prostate biopsies independently predicts outcome in patients with prostate cancer. Urol Oncol 2018;36:161.e7-161.e17. https://doi.org/10.1016/j.urolonc.2017.10.028.

[55] Tao M, Chen S, Zhang X, Zhou Q. Ki-67 labeling index is a predictive marker for a pathological complete response to neoadjuvant chemotherapy in breast cancer: A meta-analysis. Medicine (Baltimore) 2017;96:e9384. https://doi.org/10.1097/MD.0000000000009384.

[56] Zhang A, Wang X, Fan C, Mao X. The Role of Ki67 in Evaluating Neoadjuvant Endocrine Therapy of Hormone Receptor-Positive Breast Cancer. Front Endocrinol (Lausanne) 2021;12:687244. https://doi.org/10.3389/fendo.2021.687244.

[57] Penault-Llorca F, Radosevic-Robin N. Ki67 assessment in breast cancer: an update. Pathology 2017;49:166–71. https://doi.org/10.1016/j.pathol.2016.11.006.

[58] Smith IE, Dowsett M, Ebbs SR, Dixon JM, Skene A, Blohmer J-U, et al. Neoadjuvant treatment of postmenopausal breast cancer with anastrozole, tamoxifen, or both in combination: the Immediate Preoperative Anastrozole, Tamoxifen, or Combined with Tamoxifen (IMPACT) multicenter double-blind randomized trial. J Clin Oncol 2005;23:5108–16. https://doi.org/10.1200/JCO.2005.04.005.

[59] Smith I, Robertson J, Kilburn L, Wilcox M, Evans A, Holcombe C, et al. Long-term outcome and prognostic value of Ki67 after perioperative endocrine therapy in postmenopausal women with hormone-sensitive early breast cancer (POETIC): an open-label, multicentre, parallel-group, randomised, phase 3 trial. Lancet Oncol 2020;21:1443–54.

DocuSign Envelope ID: B9663506-7921-47F0-B9D8-557D7FE39808

https://doi.org/10.1016/S1470-2045(20)30458-7.

[60] Gordetsky J, Epstein J. Grading of prostatic adenocarcinoma: current state and prognostic implications. Diagn Pathol 2016;11:25. https://doi.org/10.1186/s13000-016-0478-2.

[61] Epstein JI, Zelefsky MJ, Sjoberg DD, Nelson JB, Egevad L, Magi-Galluzzi C, et al. A Contemporary Prostate Cancer Grading System: A Validated Alternative to the Gleason Score. Eur Urol 2016;69:428–35. https://doi.org/10.1016/j.eururo.2015.06.046.

[62] Epstein JI. Prostate cancer grading: a decade after the 2005 modified system. Mod Pathol 2018;31:47–63. https://doi.org/10.1038/modpathol.2017.133.

[63] Berney DM, Beltran L, Fisher G, North B V, Greenberg D, Møller H, et al. Validation of a contemporary prostate cancer grading system using prostate cancer death as outcome. Br J Cancer 2016;114:1078–83. https://doi.org/10.1038/bjc.2016.86.

[64] Raap M, Ließem S, Rüschoff J, Fisseler-Eckhoff A, Reiner A, Dirnhofer S, et al. Quality assurance trials for Ki67 assessment in pathology. Virchows Arch 2017;471:501–8. https://doi.org/10.1007/s00428-017-2142-y.

[65] James ND, de Bono JS, Spears MR, Clarke NW, Mason MD, Dearnaley DP, et al. Abiraterone for Prostate Cancer Not Previously Treated with Hormone Therapy. N Engl J Med 2017;377:338–51. https://doi.org/10.1056/NEJMoa1702900.

[66] Nguyen PL, Huang HC, Davicioni E, Sandler HM, Shipley WU, Efstathiou JA, et al. Validation of a 22-gene Genomic Classifier in the NRG Oncology/RTOG 9202, 9413 and 9902 Phase III Randomized Trials: A Biopsy-Based Individual Patient Meta-Analysis in High-Risk Prostate Cancer. Int J Radiat Oncol 2021;111:S50. https://doi.org/10.1016/j.ijrobp.2021.07.133.

[67] Choi W, Porten S, Kim S, Willis D, Plimack ER, Hoffman-Censits J, et al. Identification of Distinct Basal and Luminal Subtypes of Muscle-Invasive Bladder Cancer with Different Sensitivities to Frontline Chemotherapy. Cancer Cell 2014;25:152–65. https://doi.org/10.1016/j.ccr.2014.01.009.

[68] Weiner A, Liu Y, Ross A, Feng F, Tran P, Zaorsky N, et al. Molecular Subtyping of >80,000 Prostate Cancer Transcriptomes Identifies Four Classes With Distinct Biological and Clinical Characteristics With Implications for Targeted Therapies. J Urol 2022;207. https://doi.org/10.1097/ju.0000000000002526.07.

[69] Tian L, Zhao L, Wei LJ. Predicting the restricted mean event time with the subject's baseline covariates in survival analysis. Biostatistics 2014;15:222–33. https://doi.org/10.1093/biostatistics/kxt050.

[70] Xie W, Regan MM, Buyse M, Halabi S, Kantoff PW, Sartor O, et al. Metastasis-Free Survival Is a Strong Surrogate of Overall Survival in Localized Prostate Cancer. J Clin Oncol 2017;35:3097–104. https://doi.org/10.1200/JCO.2017.73.9987.

[71] Rosenthal SA, Hu C, Sartor O, Gomella LG, Amin MB, Purdy J, et al. Effect of Chemotherapy With Docetaxel With Androgen Suppression and Radiotherapy for Localized High-Risk Prostate Cancer: The Randomized Phase III NRG Oncology RTOG 0521 Trial. J Clin Oncol 2019;37:1159–68. https://doi.org/10.1200/JCO.18.02158.

[72] Schmoor C, Sauerbrei W, Schumacher M. Sample size considerations for the evaluation of prognostic factors in survival analysis. Stat Med 2000;19:441–52. https://doi.org/10.1002/(SICI)1097-0258(20000229)19:4<441::AID-SIM349>3.0.CO;2-N.

APPENDIX 1: RANDOMISATION PERIODS

Where stated, hazards in survival models will be stratified by the time periods patients were randomized to, as per earlier analyses. Time periods (see Table 5 below) are defined by trial arms opening or closing, a change to the standard-of-care, or another fundamental aspect which may affect the patient population being randomized. Due to the limited number of patients randomised during periods 4 and 6 in the analysis sample, we will collapse strata 3-4 and 6-7 for this purpose.

Table 5 STAMPEDE multi-stage periods used for hazard stratification

Time Period	Definition	Accrual Start Date	Accrual End Date	Co-Recruiting Research Arms
1	From the start of the trial up to the stopping of the celecoxib-containing research Arms D & F	05-Oct-2005	06-Apr-2011	B C D E F
2	Post-closure of Arms D & F up to the opening of the abiraterone research Arm G	06-Apr-2011	14-Nov-2011	B C E
3	Post-opening of Arm G up to the opening of the M1 radiotherapy research Arm H	15-Nov-2011	21-Jan-2013	B C E G
4 (merged into 3)	Post-opening of Arm H up to the closure of the remaining original research Arms B, C & E	22-Jan-2013	31-Mar-2013	B C E G H
5	Post-closure of Arms B, C & E up to the closure of abiraterone research Arm G	01-Apr-2013	17-Jan-2014	G H
6 (merged into 7)	Post-closure of Arm G up to the opening of the enzalutamide+abiraterone research Arm J	18-Jan-2014	28-Jul-2014	H
7	Post-opening Arm J until docetaxel becomes SOC	29-Jul-2014	15-Dec-2015	H J
8	Post-opening Arm J after docetaxel becomes SOC, until close of Arm J	16-Dec-2015	31-Mar-2016	H J

DocuSign Envelope ID: B9663506-7921-47F0-B9D8-557D7FE39808

APPENDIX 2: POWER CALCULATIONS

3.5 PAM50 SIGNATURE

The first hypothesis tested as part of the primary objective is the existence of an interaction effect between luminal B (PAM50) and allocation to docetaxel in de novo metastatic patients enrolled into comparisons C or E (N=1,387). Among those, 524 patients (1) had not opted out to tissue acquisition and processing; (2) had specimens obtained and subjected to expression profiling; (3) had specimens within -270 days and +14 days of randomisation; and (4) had specimens taken before initiation of ADT. Assuming balanced allocation between control and treatment; that 85% pass quality control; and that 0% are luminal A, 39% luminal B, and 61% basal, we expect the analytical cohort to consist of:

ï 524 * 0.88 * 1 = 461 patients after exclusion of luminal A (50% allocated to ADT, 50% allocated to ADT + docetaxel +/- zoledronic acid)

ï 524 * 0.88 * 0.39 = 180 luminal B patients

ï 524 * 0.88 * 0.61 = 281 basal patients

Assuming independence between allocation to treatment and luminal/basal subtyping, and that 69% of the cohort has an all-cause death event record, estimated power for plausible effect sizes are plotted on Figure 4 below based on exponential approximation formulae by Schmoor et al. [72].

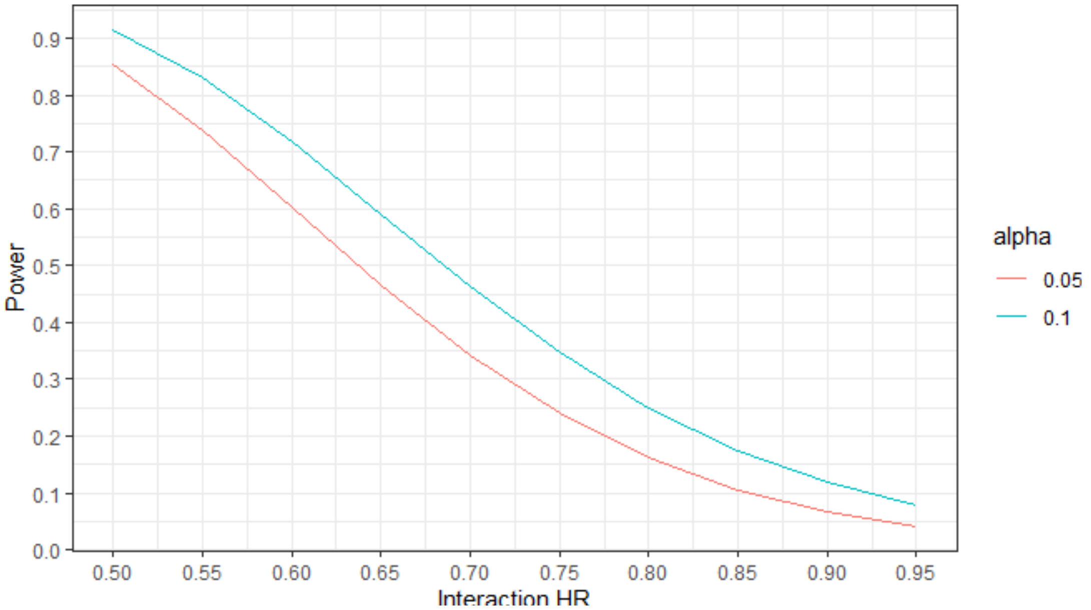

Figure 4 Power to detect a treatment effect interaction with PAM50 in the metastatic cohort allocated to ADT or ADT + docetaxel +/- zoledronic acid (ACE)

In localised disease, power was estimated for two possible endpoints: overall survival (assuming 35% of the cohort has an event) and metastases-free survival (assuming 43% of the cohort has an event), assuming 2% are luminal A, 45% luminal B, and 54% basal (Figure 5 below).

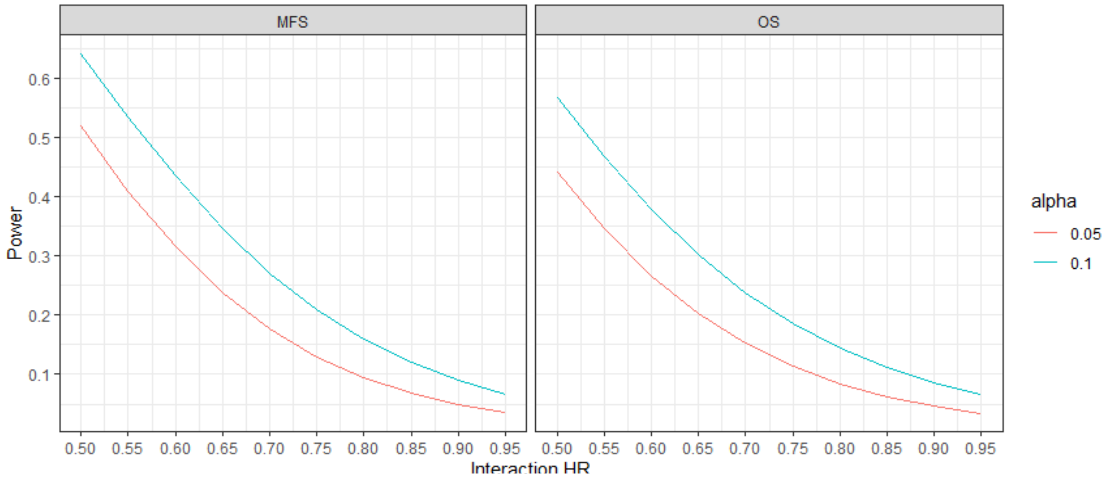

Figure 5 Power to detect a treatment effect interaction with PAM50 (luminal B vs basal) in the localised cohort allocated to ADT or ADT + docetaxel +/- zoledronic acid (ACE) by endpoint (OS vs MFS)

3.6 PSC SIGNATURE

The second hypothesis tested as part of the primary objective is the existence of an interaction effect between luminal B (PSC) and allocation to docetaxel in de novo metastatic patients enrolled into comparisons C or E. Assuming a class distribution of: 3% Luminal Differentiated (LD); 33% Luminal Proliferating (LP); 52% Basal Immune (BI); 12% Basal Neuroendocrine-Like (BN), we expect the analytical cohort to consist of:

- 524 * 0.88 * 0.85 = 392 patients after exclusion of LD and BN (50% allocated to ADT, 50% allocated to ADT + docetaxel +/- zoledronic acid)
- 524 * 0.88 * 0.33 = 152 LP patients
- 524 * 0.88 * 0.52 = 240 BI patients.

Assuming independence between allocation to treatment and luminal/basal subtyping, and that 69% of the cohort has an all-cause death event record, estimated power for plausible effect sizes are plotted on Figure 6 below.

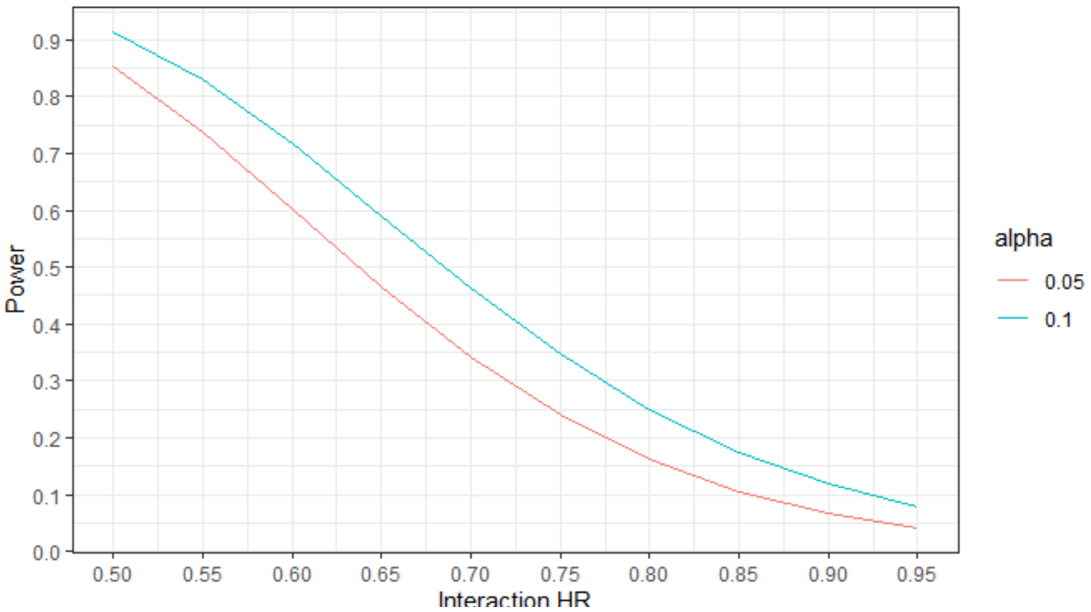

Figure 6 Power to detect a treatment effect interaction with PSC (LP vs BI) in the metastatic cohort allocated to ADT or ADT + docetaxel +/- zoledronic acid (ACE)

In localised disease, power was estimated for two possible endpoints: overall survival (assuming 35% of the cohort has an event) and metastases-free survival (assuming 43% of the cohort has an event), assuming a class distribution of: 31% Luminal Proliferating (LP) and 52% Basal Immune (BI).

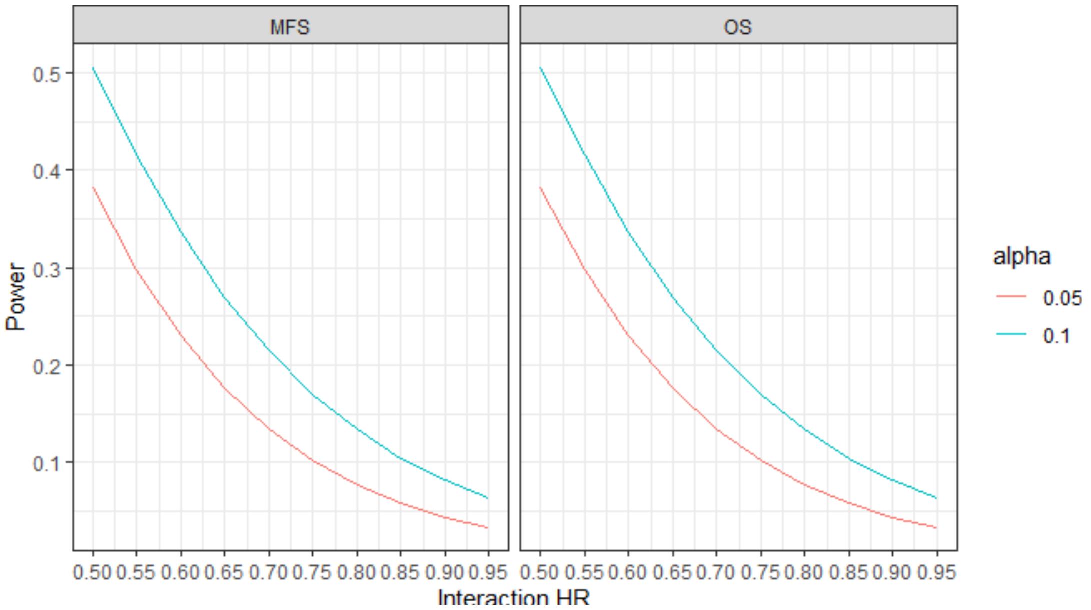

Figure 7 Power to detect a treatment effect interaction with PSC (LP vs BI) in the localised cohort allocated to ADT or ADT + docetaxel +/- zoledronic acid (ACE)

3.7 KI-67 SCORE

No closed form formulae are available for the power of detecting interaction effects with continuous variables in survival analysis models, but it is possible to examine power for a binary reclassification

of Ki-67 around the median. Simulation studies have shown the power to detect treatment effect modification with dichotomised factors is severely reduced. The estimates presented below at the 5% significance level can therefore be considered to be conservative (Figure 8).

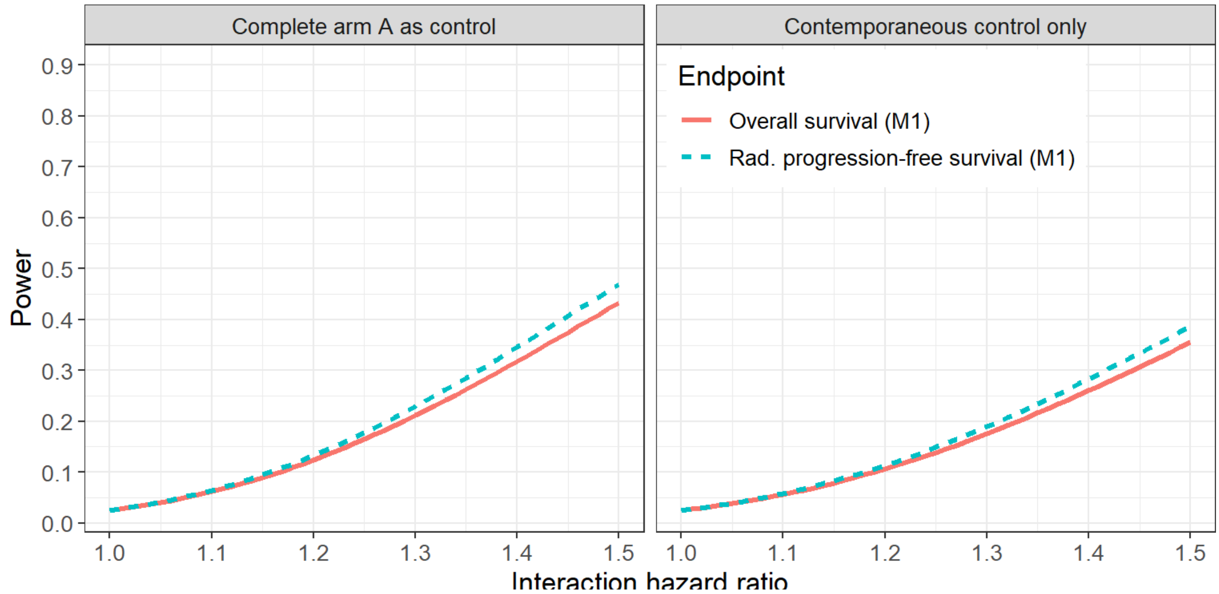

Figure 8 Statistical power for the docetaxel predictive hypothesis in metastatic patients (arms A+C) with significance level of 0.05 depending on the magnitude of the Ki-67 (<= median) hazard ratio parameter and the choice of endpoint. Overall survival, the chosen endpoint, is compared with radiological progression-free survival.

APPENDIX 3: LIST OF GENE EXPRESSION SIGNATURES FOR PROGNOSTIC ANALYSES

abi_sens
ADT_sens
Angiogenesis
AR_HM
AR-A
ARv7
ARv7_Sharp
Basal_Zhang
Bromo10
CCP
CHD1_Liu
cholesterol_HM
chrom_instab
Decipher
EMT_HM
ERE_HM
ERG
ERL_HM
EST_Purity
EST_Stromal
FA
FA_HM
Glycolysis
GR
Hieronymus
Hieronymus_repr
HR
HRdef
Hypoxia_HM
IFN_HM
Imm190
inflammation_HM
IPS_CD4_Act
IPS_CD8_Act
IPS_CTLA4
IPS_MDSC
IPS_PDL2
IPS_Treg
KRAS_HM
Kumar
Mtorc1_HM
NE_Alshalalfa
NE_Balanis
NE_Beltran
NE_Kumar
NE_Tsai
PAM50
Persist

PI3K_HM
PORTOS
Prosgenesis
PSC
PTEN_Liu
PTEN_Saal
RT_sens
SPOP_Liu
TLS
TP53_1
TP53_2

DocuSign

Certificate Of Completion

Envelope Id: B9663506792147F0B9D8557D7FE39808

Status: Completed

Subject: Complete with DocuSign: SAP_docetaxel_transcriptomics_v2.0.pdf

Source Envelope:

Document Pages: 34

Certificate Pages: 5

AutoNav: Enabled

EnvelopeId Stamping: Enabled

Time Zone: (UTC) Dublin, Edinburgh, Lisbon, London

Signatures: 7

Initials: 0

Envelope Originator:
Asiyya Tahsin
90 High Holborn 2nd Floor London
London, London WC1V 6LJ
asiyya.tahsin.21@ucl.ac.uk
IP Address: 128.40.216.243

Record Tracking

Status: Original
05 March 2024 | 11:01

Holder: Asiyya Tahsin
asiyya.tahsin.21@ucl.ac.uk

Location: DocuSign

Signer Events | Signature | Timestamp

Elai Davicioni
Elai.Davicioni@veracyte.com
Security Level: Email, Account Authentication (Optional)

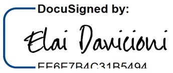

Signature Adoption: Pre-selected Style
Using IP Address: 151.41.89.150

Sent: 05 March 2024 | 11:16
Viewed: 05 March 2024 | 12:58
Signed: 05 March 2024 | 13:02

Electronic Record and Signature Disclosure:
Accepted: 05 March 2024 | 12:58
ID: 7e405222-c1c5-4af4-b15d-8b5c51ba61c5

Emily Grist
e.grist@ucl.ac.uk
Security Level: Email, Account Authentication (Optional)

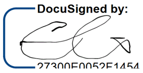

Signature Adoption: Drawn on Device
Using IP Address: 90.209.121.220
Signed using mobile

Sent: 05 March 2024 | 11:16
Viewed: 05 March 2024 | 20:49
Signed: 05 March 2024 | 20:50

Electronic Record and Signature Disclosure:
Accepted: 05 March 2024 | 20:49
ID: f449fb2f-5e09-462b-b8e8-8badc8f9c8f0

Gert Attard
g.attard@ucl.ac.uk
Security Level: Email, Account Authentication (Optional)

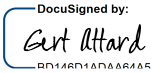

Signature Adoption: Pre-selected Style
Using IP Address: 82.132.215.231
Signed using mobile

Sent: 05 March 2024 | 11:16
Viewed: 05 March 2024 | 17:01
Signed: 05 March 2024 | 17:01

Electronic Record and Signature Disclosure:
Accepted: 17 April 2023 | 17:05
ID: f8d3c507-5a2a-4659-8c94-185c1388028b

James Proudfoot
james.proudfoot@veracyte.com
Security Level: Email, Account Authentication (Optional)

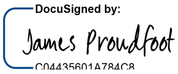

Signature Adoption: Pre-selected Style
Using IP Address: 68.6.248.37

Sent: 05 March 2024 | 11:16
Viewed: 05 March 2024 | 16:17
Signed: 05 March 2024 | 21:45

Electronic Record and Signature Disclosure:
Accepted: 05 March 2024 | 16:17

ID: 35d8834b-7dda-4d7e-ba84-70ecd47e27f5

Signer Events	Signature	Timestamp
Louise Brown<br>l.brown@ucl.ac.uk<br>Security Level: Email, Account Authentication (Optional)	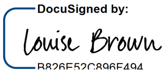<br>Signature Adoption: Pre-selected Style<br>Using IP Address: 128.40.217.95	Sent: 05 March 2024 I 11:16<br>Viewed: 05 March 2024 I 11:21<br>Signed: 05 March 2024 I 11:21

Electronic Record and Signature Disclosure:
Accepted: 05 March 2024 I 11:21
ID: d10be963-67d6-471b-97a9-9beec1f201c0

Nick James<br>nick.james@icr.ac.uk<br>Professor of Prostate and Bladder Cancer Research<br>Security Level: Email, Account Authentication (Optional)	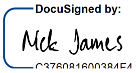<br>Signature Adoption: Pre-selected Style<br>Using IP Address: 193.63.217.166	Sent: 05 March 2024 I 11:16<br>Viewed: 05 March 2024 I 16:32<br>Signed: 05 March 2024 I 16:32

Electronic Record and Signature Disclosure:
Accepted: 09 December 2020 I 10:41
ID: 13ee3ebf-5cd5-48e3-8ba2-b612deb1e4e0

Peter Dutey-Magni<br>p.dutey-magni@ucl.ac.uk<br>Security Level: Email, Account Authentication (Optional)	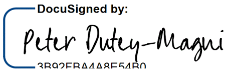<br>Signature Adoption: Pre-selected Style<br>Using IP Address: 128.40.216.253	Sent: 05 March 2024 I 11:16<br>Viewed: 05 March 2024 I 11:19<br>Signed: 05 March 2024 I 11:20

Electronic Record and Signature Disclosure:
Accepted: 05 March 2024 I 11:19
ID: 838c1147-5594-4a86-8364-58f8bf03142d

In Person Signer Events	Signature	Timestamp

Editor Delivery Events	Status	Timestamp

Agent Delivery Events	Status	Timestamp

Intermediary Delivery Events	Status	Timestamp

Certified Delivery Events	Status	Timestamp

Carbon Copy Events	Status	Timestamp

Witness Events	Signature	Timestamp

Notary Events	Signature	Timestamp

Envelope Summary Events	Status	Timestamps
Envelope Sent	Hashed/Encrypted	05 March 2024 I 11:16
Certified Delivered	Security Checked	05 March 2024 I 11:19
Signing Complete	Security Checked	05 March 2024 I 11:20
Completed	Security Checked	05 March 2024 I 21:45

Payment Events	Status	Timestamps

Electronic Record and Signature Disclosure

ELECTRONIC RECORD AND SIGNATURE DISCLOSURE

From time to time, MRC Clinical Trials Unit at UCL (we, us or Company) may be required by law to provide to you certain written notices or disclosures. Described below are the terms and conditions for providing to you such notices and disclosures electronically through the DocuSign system. Please read the information below carefully and thoroughly, and if you can access this information electronically to your satisfaction and agree to this Electronic Record and Signature Disclosure (ERSD), please confirm your agreement by selecting the check-box next to 'I agree to use electronic records and signatures' before clicking 'CONTINUE' within the DocuSign system.

Getting paper copies

At any time, you may request from us a paper copy of any record provided or made available electronically to you by us. You will have the ability to download and print documents we send to you through the DocuSign system during and immediately after the signing session and, if you elect to create a DocuSign account, you may access the documents for a limited period of time (usually 30 days) after such documents are first sent to you. After such time, if you wish for us to send you paper copies of any such documents from our office to you, you will be charged a $0.00 per-page fee. You may request delivery of such paper copies from us by following the procedure described below.

Withdrawing your consent

If you decide to receive notices and disclosures from us electronically, you may at any time change your mind and tell us that thereafter you want to receive required notices and disclosures only in paper format. How you must inform us of your decision to receive future notices and disclosure in paper format and withdraw your consent to receive notices and disclosures electronically is described below.

Consequences of changing your mind

If you elect to receive required notices and disclosures only in paper format, it will slow the speed at which we can complete certain steps in transactions with you and delivering services to you because we will need first to send the required notices or disclosures to you in paper format, and then wait until we receive back from you your acknowledgment of your receipt of such paper notices or disclosures. Further, you will no longer be able to use the DocuSign system to receive required notices and consents electronically from us or to sign electronically documents from us.

All notices and disclosures will be sent to you electronically

Unless you tell us otherwise in accordance with the procedures described herein, we will provide electronically to you through the DocuSign system all required notices, disclosures, authorizations, acknowledgements, and other documents that are required to be provided or made available to you during the course of our relationship with you. To reduce the chance of you inadvertently not receiving any notice or disclosure, we prefer to provide all of the required notices and disclosures to you by the same method and to the same address that you have given us. Thus, you can receive all the disclosures and notices electronically or in paper format through the paper mail delivery system. If you do not agree with this process, please let us know as described below. Please also see the paragraph immediately above that describes the consequences of your electing not to receive delivery of the notices and disclosures electronically from us.

How to contact MRC Clinical Trials Unit at UCL:

You may contact us to let us know of your changes as to how we may contact you electronically, to request paper copies of certain information from us, and to withdraw your prior consent to receive notices and disclosures electronically as follows:
To contact us by email send messages to: s.assam@ucl.ac.uk

To advise MRC Clinical Trials Unit at UCL of your new email address

To let us know of a change in your email address where we should send notices and disclosures electronically to you, you must send an email message to us at s.assam@ucl.ac.uk and in the body of such request you must state: your previous email address, your new email address. We do not require any other information from you to change your email address.

If you created a DocuSign account, you may update it with your new email address through your account preferences.

To request paper copies from MRC Clinical Trials Unit at UCL

To request delivery from us of paper copies of the notices and disclosures previously provided by us to you electronically, you must send us an email to s.assam@ucl.ac.uk and in the body of such request you must state your email address, full name, mailing address, and telephone number. We will bill you for any fees at that time, if any.

To withdraw your consent with MRC Clinical Trials Unit at UCL

To inform us that you no longer wish to receive future notices and disclosures in electronic format you may:

i. decline to sign a document from within your signing session, and on the subsequent page, select the check-box indicating you wish to withdraw your consent, or you may;

ii. send us an email to s.assam@ucl.ac.uk and in the body of such request you must state your email, full name, mailing address, and telephone number. We do not need any other information from you to withdraw consent.. The consequences of your withdrawing consent for online documents will be that transactions may take a longer time to process..

Required hardware and software

The minimum system requirements for using the DocuSign system may change over time. The current system requirements are found here: https://support.docusign.com/guides/signer-guide-signing-system-requirements.

Acknowledging your access and consent to receive and sign documents electronically

To confirm to us that you can access this information electronically, which will be similar to other electronic notices and disclosures that we will provide to you, please confirm that you have read this ERSD, and (i) that you are able to print on paper or electronically save this ERSD for your future reference and access; or (ii) that you are able to email this ERSD to an email address where you will be able to print on paper or save it for your future reference and access. Further, if you consent to receiving notices and disclosures exclusively in electronic format as described herein, then select the check-box next to 'I agree to use electronic records and signatures' before clicking 'CONTINUE' within the DocuSign system.

By selecting the check-box next to 'I agree to use electronic records and signatures', you confirm that:

- You can access and read this Electronic Record and Signature Disclosure; and
- You can print on paper this Electronic Record and Signature Disclosure, or save or send this Electronic Record and Disclosure to a location where you can print it, for future reference and access; and
- Until or unless you notify MRC Clinical Trials Unit at UCL as described above, you consent to receive exclusively through electronic means all notices, disclosures, authorizations, acknowledgements, and other documents that are required to be provided or made available to you by MRC Clinical Trials Unit at UCL during the course of your relationship with MRC Clinical Trials Unit at UCL.

STAMPEDE OVERSIGHT COMMITTEES, STAFF AND COLLABORATORS

Version: 16-Jan-2025

CONTENTS

NOTES 2

TRIAL PARTICIPANTS.....2

TRIAL MANAGEMENT GROUP.....3

INDEPENDENT DATA MONITORING COMMITTEE.....4

TRIAL STEERING COMMITTEE.....5

MRC CLINICAL TRIALS UNIT AT UCL STAFF.....6

SWISS GROUP FOR CANCER CLINICAL RESARCH (SAKK) STAFF.....10

BIOLOGY AND IMAGING SUBGROUPS.....11

INVESTIGATORS AND COLLABORATORS: SITE STAFF.....12

INDUSTRY COLLABORATORS.....201

NOTES

Investigators and site staff are those who have formally appeared at any time on a site's Delegation Logs.

CTU staff are those who have worked on or contributed to the trial any time from the outset until the date of this report.

The independent members of the Independent Data Monitoring Committee and Trial Oversight Committee play an important role in the conduct of the trial.

Industry collaborators are a subset of the people who have worked, on relevant sections, with the trial staff to ensure the trial runs efficiently.

TRIAL PARTICIPANTS

Nearly 12,000 people have chosen to participate in STAMPEDE. In addition to their clinical teams, they have been supported by family, friends and other key people. Every person who has participated in the trial is appreciated by the trial team and should be appreciated by the wider public. The findings from clinical trials can change practice for the future, but clinical trials only happen because people find the time and make the effort to support them. Thank you.

TRIAL MANAGEMENT GROUP

(Listing only member external to the trials unit or senior staff at the trials unit)

Area	Status	Member	Geography	Role
Clinical/Surgical	Current	Gerhardt Attard	London (UCL), UK	CCI
~	~	Simon Chowdhury	London (Guys), UK	
~	~	Noel Clarke	Manchester, UK	Deputy CI, Co-CCI
~	~	William Cross	Leeds, UK	
~	~	David Dearnaley	Sutton, UK	
~	~	Silke Gillessen	Lugano, Switzerland[1]	CCI
~	~	Nicholas James	London (ICR), UK[2]	CI
~	~	Rob Jones	Glasgow, UK	
~	~	Zafar Malik	Wirral, UK	
~	~	Chris Parker	Sutton, UK	CCI
~	~	J Martin Russell	Glasgow, BOC	
~	Previous	Daniel Aebersold	Berne, Switzerland	
~	~	John Anderson	Sheffield, UK	
~	~	Johann de Bono	Sutton, UK	
~	~	Malcolm Mason	Cardiff, UK	
~	~	John Masters	London, UK	
~	~	Rick Popert	London (Guys), UK	
~	~	Alastair Ritchie	Gloucester, UK	
~	~	George Thalmann	Berne, Switzerland	
PPI	Current	David Matheson	Other	
~	~	Robin Millman	~	
~	~	Patrick Williams	~	
~	Previous	John Dwyer	~	
~	~	David Hoe-Richardson	~	
~	~	Jim Stansfeld	~	
Senior CTU	Current	Claire Amos	MRC CTU at UCL	
~	~	Kitty Chan	~	
~	~	Claire Murphy	~	
~	~	Louise Brown	~	
~	~	Duncan Gilbert	~	
~	~	Ruth Langley	~	CCI
~	~	Mahesh Parmar	~	Programme Lead[3]
~	~	Matthew Sydes	~	
~	Previous	Nafisah Atako	~	
~	~	Cheryl Pugh	~	
Clinical Fellow	Current	Hoda Abdel-Aty	MRC CTU at UCL	
~	~	Minal Padden-Modi	~	
~	~	Mahaz Kayani	~	
~	~	Sarah Howlett	~	
~	~	Hannah Rush	~	
~	Previous	Clare Gilson	~	
~	~	Archie MacNair	~	

Key: CI = Chief Investigator
CCI = Comparison CI
CoCCI = Comparison Co-CI

Note: The full list of MRC CTU at UCL staff is detailed below in a subsequent section.

[1] Previously Manchester, UK & St Gallen, Switzerland
[2] Previously Birmingham, UK & Warwick, UK
[3] Also CTU Director

TRIAL MANAGEMENT GROUP

(Listing only member external to the trials unit or senior staff at the trials unit)

INDEPENDENT DATA MONITORING COMMITTEE

(All members independent)

Member	Status	Role
Richard Emsley	Current	
Bertrand Tombal	~	Chair 3
Ronald de Wit	~	
Chris Williams	Previous	Chair 1
John Yarnold	~	Chair 2
Doug Altman	~	
Reg Hall	~	

TRIAL STEERING COMMITTEE

(Listing only independent members)

Member	Status	Role
Paula Ghaneh	Current	Chair 3
Tim Clayton	~	
Jan-Erik Dember	~	
Paul Nash	~	PPI rep
James Larkin	~	
Jonathan Ledermann	Previous	Chair 1
Richard Emsley	~	
John Fitzpatrick	~	
Alan Horwich	~	
David Kirk	~	
Jim Paul	~	

MRC CLINICAL TRIALS UNIT AT UCL STAFF

Area	Status	Name
Statisticians	Current	Louise Brown
~	~	Peter Dutey-Magni
~	~	Laura Murphy
~	~	Matthew Nankivell
~	~	Mahesh Parmar
~	Previous	Matthew Sydes
~	~	Sophie Barthel
~	~	Adrian Cook
~	~	Daniel Bratton
~	~	Christopher Brawley
~	~	Babak Choodari-Oskooei
~	~	Trinh Duong
~	~	Andrew Embleton
~	~	Melissa Gannon (nee Spears)
~	~	Fiona Ingleby
~	~	Elizabeth James
~	~	Rachel Jinks (nee Morgan)
~	~	Gordana Jovic
~	~	Patrick Royston
~	~	Melissa Spears
Project and Trial Management	Current	
~	~	Kitty Chan
~	~	Claire Amos
~	~	Charlene Carvalho
~	~	Mazna Anjun
~	~	Anna Griffiths
~	~	Fleur Hudson
~	~	Panos Maniatis
	~	Connor McAlpine
~	~	Claire Murphy
~	~	Claire Shakeshaft
~	~	Horeja Njai
	Previous	Shabinah Ali
	~	Sofeya Ishqa
~	~	Dipa Noor
~	~	Malissa Richmond
~	~	Karen Sanders
~	~	Lily Clarke
~	~	Michelle Buckner
~	~	Nafisah Atako
~	~	Alanna Brown
~	~	Joanna Calvert
~	~	Rahela Choudhury
~	~	Zoe Cotton
~	~	Adam Cursley
~	~	Tom Fairfield
~	~	Silvia Forcat
~	~	Michelle Gabriel
~	~	Charlene Green
~	~	Adam Gregory
~	~	Anna Herasimtschuk
~	~	Caroline Hogan
~	~	Brooke Jackson

MRC CLINICAL TRIALS UNIT AT UCL STAFF

Area	Status	Name
~	~	Sarah Jackson
~	~	Neil Kelk
~	~	James Latham
~	~	Dymphna Lee
~	~	Sarah Miller
~	~	Sharon Naylor
~	~	Dipa Noor
~	~	Jacqui Nuttall
~	~	Jenny Petrie
	~	Cheryl Pugh
~	~	Orla Prendiville
~	~	Karen Sanders
~	~	Francesca Schiavone
~	~	Clare Shakeshaft
~	~	Aminata Sy
~	~	Charlotte Tyson
~	~	Hannah Vaughan
~	~	Christopher Wanstall
~	~	Katie Ward
~	~	Melanie Weiss
~	~	Arlen Wilcox
Clinicians	Current	Hoda Abdel-Aty
~	~	Duncan Gilbert
~	~	Ruth Langley
~	~	Mahaz Kayani
~	~	Minal Padden-Modi
~	~	Sarah Howlett
~	Previous	Clare Gilson
~	~	Hannah Rush
~	~	Archie Macnair
~	~	Sarah Meredith
~	~	Alastair Ritchie
Data Scientists and Programmers	Current	Christina Chung
~	~	Carlos Diaz-Montana
~	~	Georgia Marley
~	~	Lindsey Masters
~	~	Mary Rauchenberger
~	~	Stephen Townsend
~	~	Nadine Van-Looy
~	Previous	Carly Au
~	~	Will Cragg
~	~	Dominic Hague
~	~	Zaheer Islam
~	~	Sajad Khan
~	~	Dominic Mounsey
~	~	Nancy Tappenden
~	~	Nadine Van-Looy
Data Management	Current	Shaan Akbar
~	~	Yumna Ali
~	~	Ify Ejizu-Allen
~	~	Daneil Clarke
~	~	Donna Dobson

MRC CLINICAL TRIALS UNIT AT UCL STAFF

Area	Status	Name
~	~	Abinayah Baskaran
~	~	Isabella Jaques
~	~	Alexander Lawton
~	Previous	Margaret Hook
~	~	Nazia Parkar
~	~	Eva Ades
~	~	Carly Au
~	~	Katherine Beaney
~	~	Nargis Begum
~	~	Katharine Bellenger
~	~	Lina Bergstrom
~	~	Veronica Birzu
~	~	Robin Carpenter
~	~	Elizabeth Clark
~	~	Emma Donoghue
~	~	Amy Fiddament
~	~	Shree Gajjar
~	~	Hannah Gardner
~	~	Jenna Grabey
~	~	Richard Gracie
~	~	Charlene Green
~	~	Adam Gregory
~	~	Dominic Hague
~	~	Shama Hassan
~	~	Jordan Hedges
~	~	Robyn Henry-Cockles
~	~	William Hudson
~	~	Sofeya Ishaq
~	~	Danielle Johnson
~	~	Saba Khan
~	~	Zohrah Khan
~	~	Linda Ly
~	~	Adele Mabley
~	~	Georgia Mannion-Krase
~	~	Jacque Millett
~	~	Myfanwy Nicholas
~	~	Rachel Ogunleye
~	~	Meghna Pandya
~	~	Reena Patel
~	~	Sara Peres
~	~	Tasmin Philips
~	~	Philip Pollock
~	~	Chathurika Rajapakse
~	~	Tim Smith
~	~	Hannah Sweeney
~	~	Arpita Upadhyaya
~	~	Laura Van Dyck
~	~	Hannah Vaughan
~	~	Peter Vaughan
~	~	Katie Ward
~	~	Steph Wetton
~	~	Andrew Whitney
~	~	Selin Yurdakul
Other Operations	Current	Fleur Hudson

MRC CLINICAL TRIALS UNIT AT UCL STAFF

Area	Status	Name
~	~	Nicola Joffe
~	~	Macey Murray
Trial Assistants	Current	Tracey Fisher
~	~	Lynda Micklewright
~	Previous	Elizabeth Adesanya
~	~	Yumna Ali
~	~	Atma Amin
~	~	Hannah Babiker
~	~	Bryony Bathie
~	~	Helen Chapman
~	~	Georgia Cowley
~	~	Leigh Dobson
~	~	James Dunn
~	~	Robbie Dunn
~	~	Amy Fiddament
~	~	Ben Forson
~	~	Adam Gregory
~	~	Nasir Jamil
~	~	Tasheeka Jeyapalan
~	~	Sherwen Kang
~	~	Harry Kitson
~	~	Rebecca Lo
~	~	Joseph Martin
~	~	Nour Merzouki
~	~	Lynda Micklewright
~	~	Meghna Pandya
~	~	Ray Phillips
~	~	Jamie Simmons
~	~	Shanaz Sohail
~	~	Jeevan Sohal
~	~	Crystallynn The
~	~	Nat Thorogood
~	~	Stephanie Tsenti
~	~	Alexandra Wadia
~	~	Stephanie Wetton
STOPCAP Meta-Analysis team	Current	Sarah Burdett
~	~	David Fisher
~	~	Peter Godolphin
~	~	Larysa Rydzewska
~	~	Jayne Tierney
~	~	Claire Vale
Administration support	Current	Sarah Banbury
~	Previous	Lesley Brempong
~	~	Emmanuel Harding
~	~	Gillian Hurst
~	~	Nishat Tasnim
~	~	Jemima Thompson

SWISS GROUP FOR CANCER CLINICAL RESARCH (SAKK) STAFF

Area	Status	Name
SAKK operations	~	Estelle Cassolly
~	~	Pierre Fustier
~	~	Eloïse Kremer
~	~	Corinne Schar

BIOLOGY AND IMAGING SUBGROUPS

(Members of translational subgroups or work packages; TMG members not repeated here)

Person	Status	Geography
Adnan Ali	Previous	Manchester, UK
Radhi Anand	Previous	London (UCL), UK
Hassan Douis	Previous	Birmingham, UK
Dan Berney	Current	London (Barts), UK
Mick Brown	~	Manchester, UK
Ros Eeles	~	London (ICR), UK
Omar El-Taji	~	Manchester, UK
Stephenie Friedrich	~	London (UCL), UK
Emily Grist	~	London (UCL), UK
Anis A Hamid	~	Melbourne, Aus & Boston (DFCI), USA
Aine Haran	~	Manchester, UK
Craig Jones	~	Manchester, UK
A M Mahedi Hassan	~	London (UCL), UK
Alex Hoyle	~	Manchester, UK
Sakunthala Kudahetti	~	London (Barts), UK
Sharanpreet Lall	~	London (UCL), UK
Gianmarco Leone	~	London (UCL), UK
Hing Leung	~	Glasgow, BOC
Stefano Lise	~	London (UCL), UK
Larissa Mendes	~	London (UCL), UK
Karolina Nowakowska-Pawelkowicz	~	London (UCL), UK
Charles Parker	~	London (UCL), UK
Marina Parry	~	London (UCL), UK
Alison Parry-Jones	~	Cardiff, UK
Ashwin Sachdeva	~	Manchester, UK
Chris Sweeney	~	Boston (DFCI), USA, Adelaide Australia
Suparna Thakali	~	London (UCL), UK
Nina Tinariu	~	London (ICR), UK
Maria Vico	~	London (UCL), UK
Sara Santos Vidal	~	London (Barts), UK
Daniel Wetterskog	~	London (UCL), UK
Anna Wingate	~	London (UCL), UK
Carla Bautista	Previous	London (UCL), UK
Paolo Cremaschi	~	London (UCL), UK
Thomas Hambrock	~	Manchester, UK
Alex Landless	~	London (UCL), UK
Nik Matthews	~	London (ICR), UK
Mariana Buongermino Pereira	~	London (UCL), UK
Kamila Sychowska	~	London (UCL), UK
David Waugh	Previous	Belfast, UK
Leila Zakka	~	London (UCL), UK

INVESTIGATORS AND COLLABORATORS: SITE STAFF

Staff on site delegation logs

City	Care site	Name	Site PI
Abergavenny	Nevill Hall Hospital	Christian Smith	
Aberystwyth	Bronglais General Hospital	Christine Kotonya	
Aberystwyth	Bronglais General Hospital	Elin Jones	PI
Aberystwyth	Bronglais General Hospital	Helen Tench	
Aberystwyth	Bronglais General Hospital	Mark Narain	
Aberystwyth	Bronglais General Hospital	Philip Jones	
Aberystwyth	Bronglais General Hospital	Russel Canavan	
Aberystwyth	Bronglais General Hospital	Sajid Durrani	
Aberystwyth	Bronglais General Hospital	Emma Nurse	Pharmacist
Aberystwyth	Bronglais General Hospital	Gwenan Parry Jones	
Aberystwyth	Bronglais General Hospital	Kirsty Marie Dennett	
Aberystwyth	Bronglais General Hospital	Rhian Elin Jones	
Aberystwyth	Bronglais General Hospital	Sandra Griffiths nee Evens	
Aberystwyth	Bronglais General Hospital	Cerith Morgan	
Aberystwyth	Bronglais General Hospital	Kenneth Richard Williams	
Aberystwyth	Bronglais General Hospital	Llinos Strange	Pharmacist
Aberystwyth	Bronglais General Hospital	Sean Thomas	
Aberystwyth	Bronglais General Hospital	Toby Frederick Trugeion-Smith	

Aberystwyth	Bronglais General Hospital	Basharat Jameel	
Aberystwyth	Bronglais General Hospital	Bleddyn Edwards	
Aberystwyth	Bronglais General Hospital	Geraint Morgan	Pharmacist
Aberystwyth	Bronglais General Hospital	John Edwards	
Aberystwyth	Bronglais General Hospital	Donna Robson	
Aberystwyth	Bronglais General Hospital	Heather McGuinness	
Aberystwyth	Bronglais General Hospital	Ronda Loosley	
Aberystwyth	Bronglais General Hospital	Claire Duggan	
Aberystwyth	Bronglais General Hospital	Sarah Jones	
Aberystwyth	Bronglais General Hospital	Abigail Hynes	
Aberystwyth	Bronglais General Hospital	Rebecca Wolf-Roberts	
Ashford	William Harvey Hospital	Albert Edwards	Co-I
Ashford	William Harvey Hospital	Charlotte Mott	
Ashford	William Harvey Hospital	Ifigenia Vasiliadou	
Ashford	William Harvey Hospital	Lavarniya Rajakumar	
Ashford	William Harvey Hospital	Mathini Sridharan	
Ashford	William Harvey Hospital	Patryk Brulinski	
Ashford	William Harvey Hospital	Rakesh Raman	Co-I
Ashford	William Harvey Hospital	Rohit Malde	
Ashford	William Harvey Hospital	Stephane Tankoua	
Ashford	William Harvey Hospital	Arafat Mizra	
Ashford	William Harvey Hospital	Carys Thomas	PI
Ashford	William Harvey Hospital	Clary Evans	
Ashford	William Harvey Hospital	Kannon Nathan	
Ashford	William Harvey Hospital	Kathryn Lees	

Ashford	William Harvey Hospital	Mathilda Cominos	
Ashford	William Harvey Hospital	Matthew Fenton	
Ashford	William Harvey Hospital	Mohammed Osman	
Ashford	William Harvey Hospital	Natasha Mithal	Co-I
Ashford	William Harvey Hospital	Sharon Beesley	
Ashford	William Harvey Hospital	Sugeeta Sukumar	
Ashford	William Harvey Hospital	Udaiveer Panwar	
Ashford	William Harvey Hospital	Coral Greenstreet	
Ashford	William Harvey Hospital	Hayley Blackgrove	
Ashford	William Harvey Hospital	Katy Taylor	
Ashford	William Harvey Hospital	Victoria Williamson	
Ashford	William Harvey Hospital	Natalie Catt	
Ashford	William Harvey Hospital	Arafat Mirza	
Ashford	William Harvey Hospital	Sam Gibson	
Ashford	William Harvey Hospital	Steve Dann	
Ashford	William Harvey Hospital	Andrew Gillian	Pharmacist
Ashford	William Harvey Hospital	Miguel Capo-Mir	Pharmacist
Ashford	William Harvey Hospital	Cindy Slater	
Ashford	William Harvey Hospital	Hasmath Marjolin	
Ashford	William Harvey Hospital	Nikki Crisp	
Ashford	William Harvey Hospital	Rachel Larkins	
Ashford	William Harvey Hospital	Sandra Holness	
Ashford	William Harvey Hospital	Sarah Lines	
Ashford	William Harvey Hospital	Susan Rogers	
Ashford	William Harvey Hospital	Tessa Hammond	Pharmacist

Ashford	William Harvey Hospital	Claire White	
Ashford	William Harvey Hospital	Julie Buckley	
Ashford	William Harvey Hospital	Laura Kehoe	
Ashford	William Harvey Hospital	Lesley Rose	
Ashford	William Harvey Hospital	Louise Gladwell	
Ashford	William Harvey Hospital	Sarah Lightfoot	Pharmacist
Ashford	William Harvey Hospital	Tracy Boakes	
Ashford	William Harvey Hospital	Alba Tubau	
Ashford	William Harvey Hospital	Bonny Appleby	
Ashford	William Harvey Hospital	Linda Wray	Pharmacist
Ashford	William Harvey Hospital	Louise Allen	
Ashford	William Harvey Hospital	Marian Wood	
Ashford	William Harvey Hospital	Adedolapo Sanni	
Ashford	William Harvey Hospital	Claire Pelham	
Ashford	William Harvey Hospital	Elizabeth Williamson	
Ashford	William Harvey Hospital	Hilary Zurakovsky	
Ashford	William Harvey Hospital	Jill Baker	
Ashford	William Harvey Hospital	Joanne Williams	
Ashford	William Harvey Hospital	Julie-Ann Davies	
Ashford	William Harvey Hospital	Karen Robinson	
Ashford	William Harvey Hospital	Kathleen (Kathy) Walsh	
Ashford	William Harvey Hospital	Kim Mears	
Ashford	William Harvey Hospital	Kim Travis	
Ashford	William Harvey Hospital	Margaret Lipsham	
Ashford	William Harvey Hospital	Paula Whichelo	

Ashford	William Harvey Hospital	Sharon Middleton	
Ashford	William Harvey Hospital	Sue Kelly	
Ashford	William Harvey Hospital	Susan Drakeley	
Ashford	William Harvey Hospital	Sydnie Loveland	
Ashford	William Harvey Hospital	Molua Young	
Ashford	William Harvey Hospital	Denise Crawford	
Aylesbury	Stoke Mandeville Hospital	Ami Sabharwal	
Aylesbury	Stoke Mandeville Hospital	Andy Theobold	
Aylesbury	Stoke Mandeville Hospital	Janice Carpenter	
Aylesbury	Stoke Mandeville Hospital	Katherine Hyde	PI
Aylesbury	Stoke Mandeville Hospital	Thinn Pwint	Co-I
Aylesbury	Stoke Mandeville Hospital	Christopher Alcock	
Aylesbury	Stoke Mandeville Hospital	Gerard Andrade	
Aylesbury	Stoke Mandeville Hospital	Joanne Brady	
Aylesbury	Stoke Mandeville Hospital	Niki Panakis	
Aylesbury	Stoke Mandeville Hospital	Philip Camilleri	Co-I
Aylesbury	Stoke Mandeville Hospital	Prabir Chakraborti	
Aylesbury	Stoke Mandeville Hospital	Sean O'Cathail	
Aylesbury	Stoke Mandeville Hospital	Jonathan Greenland	
Aylesbury	Stoke Mandeville Hospital	Moncy Mathew	
Aylesbury	Stoke Mandeville Hospital	Rahul Kurup	
Aylesbury	Stoke Mandeville Hospital	Neil Trew-Smith	
Aylesbury	Stoke Mandeville Hospital	Alice Ngumo	
Aylesbury	Stoke Mandeville Hospital	Gail Varley	
Aylesbury	Stoke Mandeville Hospital	Janet Weir	

Aylesbury	Stoke Mandeville Hospital	Manisha Joshi	
Aylesbury	Stoke Mandeville Hospital	Siobhan Gettings	
Aylesbury	Stoke Mandeville Hospital	Cheryl Padilla-Harris	
Aylesbury	Stoke Mandeville Hospital	Maggie Aldersley	
Aylesbury	Stoke Mandeville Hospital	Margaret Bowerbank	
Aylesbury	Stoke Mandeville Hospital	Michelle Taylor-Siddons	Pharmacist
Aylesbury	Stoke Mandeville Hospital	Tracey Stammers	
Aylesbury	Stoke Mandeville Hospital	Anna Osadcow	
Aylesbury	Stoke Mandeville Hospital	Kathryn Herbert	
Aylesbury	Stoke Mandeville Hospital	Rossana Mancinelli	
Aylesbury	Stoke Mandeville Hospital	Sarah Manyangadze	
Aylesbury	Stoke Mandeville Hospital	Christine Collins	
Aylesbury	Stoke Mandeville Hospital	Hazel Wynn	
Aylesbury	Stoke Mandeville Hospital	Iram Husain	
Aylesbury	Stoke Mandeville Hospital	Jasvinder Bains	
Aylesbury	Stoke Mandeville Hospital	Roisin Kavanagh	
Aylesbury	Stoke Mandeville Hospital	Andrew Protheroe	
Ayr	Ayr Hospital	Aisha Tufail	
Ayr	Ayr Hospital	David McIntosh	
Ayr	Ayr Hospital	Helena Belikova	
Ayr	Ayr Hospital	Jawaher Ansari	
Ayr	Ayr Hospital	Rebecca Muirhead	
Ayr	Ayr Hospital	Xia Ren	Co-I
Ayr	Ayr Hospital	Christina Lai	
Ayr	Ayr Hospital	Esfandiyar Khan	

Ayr	Ayr Hospital	Hilary Glen	PI
Ayr	Ayr Hospital	Lye Mun Tho	
Ayr	Ayr Hospital	Nicholas Macleod	
Ayr	Ayr Hospital	Rana Mahmood	
Ayr	Ayr Hospital	Stefan Nowich	
Ayr	Ayr Hospital	Kirsty O'Hara	
Ayr	Ayr Hospital	Kristy Ross	
Ayr	Ayr Hospital	Brian McGlynn	
Ayr	Ayr Hospital	Mark Wilson	
Ayr	Ayr Hospital	Philip Cannon	
Ayr	Ayr Hospital	Kathleen Smith	
Ayr	Ayr Hospital	Deborah Dunn	Pharmacist
Ayr	Ayr Hospital	Jane McClements	
Ayr	Ayr Hospital	Susan Walton	Pharmacist
Ayr	Ayr Hospital	Alison Murphy	Pharmacist
Ayr	Ayr Hospital	Danielle Gilmour	
Ayr	Ayr Hospital	Elaine Watson	
Ayr	Ayr Hospital	Lillian White	
Ayr	Ayr Hospital	Lynne McNeil	Pharmacist
Ayr	Ayr Hospital	Margaret McKernan	
Ayr	Ayr Hospital	Sharon Meehan	
Ayr	Ayr Hospital	Clare Love	
Ayr	Ayr Hospital	Claudia Coubrough (Turley)	
Ayr	Ayr Hospital	Danna Yorston	Pharmacist
Ayr	Ayr Hospital	Diane Woodburn	Pharmacist

Ayr	Ayr Hospital	Dianne Hunter	
Ayr	Ayr Hospital	Jenna Mitchell	
Ayr	Ayr Hospital	Kirsten Laws (nee Borthwick)	
Barnet	Barnet General Hospital	Ami Mehta	
Barnet	Barnet General Hospital	Andrew Eichholz	
Barnet	Barnet General Hospital	Anita Mitra	
Barnet	Barnet General Hospital	Annette Hawkins	
Barnet	Barnet General Hospital	Emily Scott	
Barnet	Barnet General Hospital	Gehan Soosaipillai	
Barnet	Barnet General Hospital	Gillian Marks	
Barnet	Barnet General Hospital	Kate Smith	Co-I
Barnet	Barnet General Hospital	Kimberley Durno	Co-I
Barnet	Barnet General Hospital	Magdalena Kubiak	Co-I
Barnet	Barnet General Hospital	Sarah Needleman	PI
Barnet	Barnet General Hospital	Ursula McGovern	
Barnet	Barnet General Hospital	Danielle Collier	
Barnet	Barnet General Hospital	Andie David	
Barnet	Barnet General Hospital	Panayiotis Panayiotou	
Barnet	Barnet General Hospital	Heather Hughes	
Barnet	Barnet General Hospital	Prital Patel	
Barnet	Barnet General Hospital	Sandra Faustino	
Barnet	Barnet General Hospital	Anita Amadi	
Barnet	Barnet General Hospital	Alice Coady	
Barnet	Barnet General Hospital	Christine Ellis	Pharmacist
Barnet	Barnet General Hospital	Veronica Conteh	

Barnstable	North Devon District Hospital	Becky Holbrook	
Barnstaple	North Devon District Hospital	Ajaz Lone	
Barnstaple	North Devon District Hospital	Chantal Oelofse	
Barnstaple	North Devon District Hospital	Elizabeth Kershaw	
Barnstaple	North Devon District Hospital	Faisal Hussain	
Barnstaple	North Devon District Hospital	Mohini Varughese	PI
Barnstaple	North Devon District Hospital	Peter Stephens	Co-I
Barnstaple	North Devon District Hospital	Philippa Smith	
Barnstaple	North Devon District Hospital	Sarah Park	
Barnstaple	North Devon District Hospital	Victoria Ford	Co-I
Barnstaple	North Devon District Hospital	Andy Bull	
Barnstaple	North Devon District Hospital	Denise Sheehan	
Barnstaple	North Devon District Hospital	Elizabeth Toy	
Barnstaple	North Devon District Hospital	Maria Martinez	
Barnstaple	North Devon District Hospital	Amy Thomas	
Barnstaple	North Devon District Hospital	Chloe Peters	
Barnstaple	North Devon District Hospital	Hannah Ong	
Barnstaple	North Devon District Hospital	Jenna Furse	
Barnstaple	North Devon District Hospital	Nyasha Manomano	Pharmacist
Barnstaple	North Devon District Hospital	Jeffrey man sum Chan	
Barnstaple	North Devon District Hospital	Joshua Gregory	Pharmacist
Barnstaple	North Devon District Hospital	Michal Ian Lamparski	Pharmacist
Barnstaple	North Devon District Hospital	Thomas Baddick	Pharmacist
Barnstaple	North Devon District Hospital	Eng Ong	
Barnstaple	North Devon District Hospital	Henry Goss	Pharmacist

Barnstaple	North Devon District Hospital	Martin Moody	
Barnstaple	North Devon District Hospital	Rufus Smith	
Barnstaple	North Devon District Hospital	Ashley Hanson	
Barnstaple	North Devon District Hospital	Katherine Horder	
Barnstaple	North Devon District Hospital	Maria Beaumont	Pharmacist
Barnstaple	North Devon District Hospital	Rebecca Davey	Pharmacist
Barnstaple	North Devon District Hospital	Fiona Thomas	
Barnstaple	North Devon District Hospital	Helen Black	
Barnstaple	North Devon District Hospital	Laura Hanson	
Barnstaple	North Devon District Hospital	Lynne Van-Koutrik	
Barnstaple	North Devon District Hospital	Lynsey Balmbra-Jenks	
Barnstaple	North Devon District Hospital	Natalie Kemp	Pharmacist
Barnstaple	North Devon District Hospital	Samantha Ley	
Barnstaple	North Devon District Hospital	Susan Collard	
Barnstaple	North Devon District Hospital	Alicia Leal-Santos	Pharmacist
Barnstaple	North Devon District Hospital	Faye Windsor	
Barnstaple	North Devon District Hospital	Judyta Lomza	Pharmacist
Barnstaple	North Devon District Hospital	Lynne Van Koutrik	
Basel	Universitätsspital Basel	M Timmermann	
Basel	Universitätsspital Basel	Cyrill Rentsch	PI
Basel	Universitätsspital Basel	Frank Stenner-Liewen	Co-I
Basel	Universitätsspital Basel	Nicole Ebinger	
Basel	Universitätsspital Basel	Stephen Wyler	
Basel	Universitätsspital Basel	N Ott	
Basel	Universitätsspital Basel	Cyrill Rentsch	

Basel	Universitätsspital Basel	Eloise Kremer	
Basel	Universitätsspital Basel	Heike Puschel	
Basel	Universitätsspital Basel	Heike Puschel	
Basel	Universitätsspital Basel	Mana Farsad	
Basel	Universitätsspital Basel	Nicole Neumann	
Basel	Universitätsspital Basel	Simone Marini	
Basel	Universitätsspital Basel	Bettina Seifest	
Basel	Universitätsspital Basel	Kristina Muller	
Basel	Universitätsspital Basel	Alexander Bachmann	
Basel	Universitätsspital Basel	Christoph Rochlitz	
Basingstoke	Basingstoke and North Hampshire Hospital	Dileep Soory	
Basingstoke	Basingstoke and North Hampshire Hospital	Hilawati Yusof	
Basingstoke	Basingstoke and North Hampshire Hospital	Ingrid White	
Basingstoke	Basingstoke and North Hampshire Hospital	Rosalyne Westley	
Basingstoke	Basingstoke and North Hampshire Hospital	Sangeeta Paisey	PI
Basingstoke	Basingstoke and North Hampshire Hospital	David Barlow	
Basingstoke	Basingstoke and North Hampshire Hospital	Eva Letalova	
Basingstoke	Basingstoke and North Hampshire Hospital	Jenny Nobes	
Basingstoke	Basingstoke and North Hampshire Hospital	Joanna Stokoe	
Basingstoke	Basingstoke and North Hampshire Hospital	Katharine Webb	
Basingstoke	Basingstoke and North Hampshire Hospital	Katherine Aitken	
Basingstoke	Basingstoke and North Hampshire Hospital	Katie Wood	
Basingstoke	Basingstoke and North Hampshire Hospital	Katie Wood	
Basingstoke	Basingstoke and North Hampshire Hospital	Rao Vuyyuru	
Basingstoke	Basingstoke and North Hampshire Hospital	Richard Shaffer	PI

Basingstoke	Basingstoke and North Hampshire Hospital	Sree Susaria	
Basingstoke	Basingstoke and North Hampshire Hospital	Teresa Guerrero-Urbano	
Basingstoke	Basingstoke and North Hampshire Hospital	Jo-Anna Conyngham	
Basingstoke	Basingstoke and North Hampshire Hospital	Jo-Anna Conyngham	
Basingstoke	Basingstoke and North Hampshire Hospital	Lauriane Kerwood	Pharmacist
Basingstoke	Basingstoke and North Hampshire Hospital	Lorraine Poole	
Basingstoke	Basingstoke and North Hampshire Hospital	Louise Beattie	
Basingstoke	Basingstoke and North Hampshire Hospital	Rebecca Wills	Pharmacist
Basingstoke	Basingstoke and North Hampshire Hospital	Zoe Finlay	
Basingstoke	Basingstoke and North Hampshire Hospital	Rachel Bryan	
Basingstoke	Basingstoke and North Hampshire Hospital	Duncan Cooke	
Basingstoke	Basingstoke and North Hampshire Hospital	Godfrey Bownie-Mukumbu	
Basingstoke	Basingstoke and North Hampshire Hospital	Roger Hudson	Pharmacist
Basingstoke	Basingstoke and North Hampshire Hospital	Bintha Paruthickal	
Basingstoke	Basingstoke and North Hampshire Hospital	Catherine McLean	
Basingstoke	Basingstoke and North Hampshire Hospital	Jackie Smith	
Basingstoke	Basingstoke and North Hampshire Hospital	Nanda Basker	
Basingstoke	Basingstoke and North Hampshire Hospital	Pennie Porter	
Basingstoke	Basingstoke and North Hampshire Hospital	Abigail Edwards	
Basingstoke	Basingstoke and North Hampshire Hospital	Adrienn Fazekasne Fulep	
Basingstoke	Basingstoke and North Hampshire Hospital	Angela Frith	Pharmacist
Basingstoke	Basingstoke and North Hampshire Hospital	Christine Podesta	
Basingstoke	Basingstoke and North Hampshire Hospital	Fasar Sarwar	
Basingstoke	Basingstoke and North Hampshire Hospital	Helen Richards	
Basingstoke	Basingstoke and North Hampshire Hospital	Victoria Corner	

Basingstoke	Basingstoke and North Hampshire Hospital	Carmen Wu	Pharmacist
Basingstoke	Basingstoke and North Hampshire Hospital	Catherine Rimington	
Basingstoke	Basingstoke and North Hampshire Hospital	Christina Narh	
Basingstoke	Basingstoke and North Hampshire Hospital	Claire Williams	
Basingstoke	Basingstoke and North Hampshire Hospital	Julie Gwilt	
Basingstoke	Basingstoke and North Hampshire Hospital	Kathryn Leach (nee Noake)	
Basingstoke	Basingstoke and North Hampshire Hospital	Liz Happle	
Basingstoke	Basingstoke and North Hampshire Hospital	Sara Fawcitt	
Bath	Royal United Hospital	Abigail Gee	Co-I
Bath	Royal United Hospital	Catherine McDonald	Co-I
Bath	Royal United Hospital	Claire Dyke	
Bath	Royal United Hospital	Frances Du Feu	
Bath	Royal United Hospital	Georgina Gullick	Co-I
Bath	Royal United Hospital	Ioana Fodor	
Bath	Royal United Hospital	Kathryn Falconer	
Bath	Royal United Hospital	Nathalie Webber	Co-I
Bath	Royal United Hospital	Tom Wilson	Co-I
Bath	Royal United Hospital	Abigail Jenner	
Bath	Royal United Hospital	Chris Williams	
Bath	Royal United Hospital	Christine Elwell	
Bath	Royal United Hospital	Gareth Ayre	
Bath	Royal United Hospital	Hugh Newman	
Bath	Royal United Hospital	Lorna Hawley	
Bath	Royal United Hospital	Mark Beresford	PI
Bath	Royal United Hospital	Matthew Sephton	

Bath	Royal United Hospital	Olivera Frim	Co-I
Bath	Royal United Hospital	Penny Kehagioglou	
Bath	Royal United Hospital	Susan Masson	
Bath	Royal United Hospital	Abigail Pocock	
Bath	Royal United Hospital	Claire Barron	
Bath	Royal United Hospital	Jill MacDonald-Burn	Pharmacist
Bath	Royal United Hospital	Joanne Avis	
Bath	Royal United Hospital	Leonie Harrison	
Bath	Royal United Hospital	Rachael Bolitho	
Bath	Royal United Hospital	Rachael Exley	
Bath	Royal United Hospital	Samantha Williams	Pharmacist
Bath	Royal United Hospital	Shaolin Chidavaenzi	
Bath	Royal United Hospital	Guillaume Livera	
Bath	Royal United Hospital	Joseph Needham	
Bath	Royal United Hospital	Roland Wynn-Williams	
Bath	Royal United Hospital	Michael Daly	
Bath	Royal United Hospital	Tom Tylee	
Bath	Royal United Hospital	Amy Singh	
Bath	Royal United Hospital	Joanna Wilson	
Bath	Royal United Hospital	Kristelle Vassallo	
Bath	Royal United Hospital	Rowan Appleby	
Bath	Royal United Hospital	Vicki Portingale	
Bath	Royal United Hospital	Beatrice Hamilton	
Bath	Royal United Hospital	Carey Milsom (nee Logan)	
Bath	Royal United Hospital	Christine Cox	

Bath	Royal United Hospital	Kate Moloney	
Bath	Royal United Hospital	Ruth Brydon-Hill	
Bath	Royal United Hospital	Samantha Curtis	
Bath	Royal United Hospital	Sarah Murdoch	
Bath	Royal United Hospital	Yuko Francis	
Bath	Royal United Hospital	Agata Leonarska	
Bath	Royal United Hospital	Bryony Robertson	
Bath	Royal United Hospital	Carolina Juan Chofre	
Bath	Royal United Hospital	Claire Davis	
Bath	Royal United Hospital	Eve Tomlinson	
Bath	Royal United Hospital	Jane Crozier	
Bath	Royal United Hospital	Jess White	
Bath	Royal United Hospital	Katarzyna Machura	
Bath	Royal United Hospital	Laura Cini	
Bath	Royal United Hospital	Rebecca Wassall	
Bath	Royal United Hospital	Carly Laxon-Takooree	
Bath	Royal United Hospital	Claire Craige	
Bath	Royal United Hospital	Hannah Blades	
Bath	Royal United Hospital	Jackie Davies	
Bath	Royal United Hospital	Margaret Macmillan	Pharmacist
Bath	Royal United Hospital	Vicki Clarke	
Bath	Royal United Hospital	Tania Williams (Née Allen)	
Bebington	Clatterbridge Centre for Oncology	Amir Montazeri	
Bebington	Clatterbridge Centre for Oncology	Azman Ibrahim	Co-I
Bebington	Clatterbridge Centre for Oncology	Helen Innes	

Bebington	Clatterbridge Centre for Oncology	Isabel Syndikus	
Bebington	Clatterbridge Centre for Oncology	Peter Robson	
Bebington	Clatterbridge Centre for Oncology	Shaun Tolan	
Bebington	Clatterbridge Centre for Oncology	Zafar Malik	PI
Bebington	Clatterbridge Centre for Oncology	Elizabeth Gallimore	
Bebington	Clatterbridge Centre for Oncology	Jodie Henderson	
Bebington	Clatterbridge Centre for Oncology	Rachel Pritchard	
Bebington	Clatterbridge Centre for Oncology	Sarah Dalby	
Bebington	Clatterbridge Centre for Oncology	Emma Whitby	
Bebington	Royal Liverpool University Hospital	Emma Whitby	
Bebington	Clatterbridge Centre for Oncology	Laura McAllister	
Bebington	Clatterbridge Centre for Oncology	Ian Allen	
bebington	University Hospital Aintree	Ian Allen	
Bebington	Clatterbridge Centre for Oncology	Matthew Stott	
Bebington	Clatterbridge Centre for Oncology	Paul Griffiths	
Bebington	Clatterbridge Centre for Oncology	Priyank Patel	
Bebington	Clatterbridge Centre for Oncology	Burhan Zavery	Pharmacist
Bebington	Clatterbridge Centre for Oncology	Wesley Artist	
Bebington	Clatterbridge Centre for Oncology	Annemieke Earnshaw	
Bebington	Clatterbridge Centre for Oncology	Elizabeth Harrison	
Bebington	Clatterbridge Centre for Oncology	Sharon Dunn (nee Johnson)	
Bebington	Clatterbridge Centre for Oncology	Sue Green	
Bebington	Clatterbridge Centre for Oncology	Diane Fildes	
Bebington	Clatterbridge Centre for Oncology	Helen Flint	Pharmacist
Bebington	Clatterbridge Centre for Oncology	Sandra Robinson	Pharmacist

Bebington	Clatterbridge Centre for Oncology	Caroline Dunn	
Bebington	Clatterbridge Centre for Oncology	Claire Harwood	
Bebington	Clatterbridge Centre for Oncology	Jess Hulse	
Bebington	Clatterbridge Centre for Oncology	Katie Sloan	
Bebington	Clatterbridge Centre for Oncology	Suzanne Maloney	
Bebington	Clatterbridge Centre for Oncology	Dawn Porter	
Bebington	Clatterbridge Centre for Oncology	Lisa Dobson (nee Child)	Pharmacist
Beckett Street	St James University Hospital (Leeds)	Catherine Gray	
Beckett Street	St James University Hospital (Leeds)	Claire Daisey	
Belfast	Belfast City Hospital	Claire Rooney	
Belfast	Belfast City Hospital	Laura Feeney	
Belfast	Belfast City Hospital	Laura Mooney	
Belfast	Belfast City Hospital	Michael McMahon	
Belfast	Belfast City Hospital	Nicola Hill	
Belfast	Belfast City Hospital	Orla Houlihan	
Belfast	Belfast City Hospital	Patricia Calisaya	
Belfast	Belfast City Hospital	Peter Bryson	
Belfast	Belfast City Hospital	Phil Turner	
Belfast	Belfast City Hospital	Rachel Ellis	
Belfast	Belfast City Hospital	Sai Jonnada	
Belfast	Belfast City Hospital	Seosamh McCauley	
Belfast	Belfast City Hospital	Swati Ray	Co-I
Belfast	Belfast City Hospital	Aiden Cole	
Belfast	Belfast City Hospital	Ciaran Fairmichael	
Belfast	Belfast City Hospital	Darren Mitchell	

Belfast	Belfast City Hospital	David Stewart	
Belfast	Belfast City Hospital	Jackie Harney	
Belfast	Belfast City Hospital	Jonathan McAleese	
Belfast	Belfast City Hospital	Lois Mulholland	
Belfast	Belfast City Hospital	Lucy Jellett	
Belfast	Belfast City Hospital	Melvyn Ang	
Belfast	Belfast City Hospital	Paula McCloskey	
Belfast	Belfast City Hospital	Poh Lin Shum	Co-I
Belfast	Belfast City Hospital	Prantik Das	
Belfast	Belfast City Hospital	Rebecca Goody	
Belfast	Belfast City Hospital	Rhun Evans	
Belfast	Belfast City Hospital	Ruth Eakin	
Belfast	Belfast City Hospital	Ruth Johnston	
Belfast	Belfast City Hospital	Salil Vengalil	
Belfast	Belfast City Hospital	Sarah McGahey	
Belfast	Belfast City Hospital	Stephen Stranex	
Belfast	Belfast City Hospital	Suneil Jain	Co-I
Belfast	Belfast City Hospital	Aine McKeown	
Belfast	Belfast City Hospital	Alison Logie	
Belfast	Belfast City Hospital	Jemma Robinson	
Belfast	Belfast City Hospital	Karen McKenna	
Belfast	Belfast City Hospital	Benedict Dadebo	
Belfast	Belfast City Hospital	Keith Rooney	
Belfast	Belfast City Hospital	Michael Hanna	
Belfast	Belfast City Hospital	William Snelling	

Belfast	Belfast City Hospital	Chris Hagan	
Belfast	Belfast City Hospital	Jonathan Thompson	
Belfast	Belfast City Hospital	Patrick Keane	
Belfast	Belfast City Hospital	Peter Clarke	
Belfast	Belfast City Hospital	Ciara McIlmunn	
Belfast	Belfast City Hospital	Diane Law	
Belfast	Belfast City Hospital	Ellen Brown	
Belfast	Belfast City Hospital	Fiona Tarpey	
Belfast	Belfast City Hospital	Karen Campfield	
Belfast	Belfast City Hospital	Kerry Nicholls	
Belfast	Belfast City Hospital	Margot Creighton	
Belfast	Belfast City Hospital	Eileen Dillon	
Belfast	Belfast City Hospital	Joanne McAllister	
Belfast	Belfast City Hospital	Kairen McCloy	
Belfast	Belfast City Hospital	Linda McNeice	Pharmacist
Belfast	Belfast City Hospital	Lynsey Morrow	
Belfast	Belfast City Hospital	Stacey Hetherington	
Belfast	Belfast City Hospital	Wendy Cunningham	
Belfast	Belfast City Hospital	Adrina O'Donnell	
Belfast	Belfast City Hospital	Catherine Davidson	
Belfast	Belfast City Hospital	Chryelle McAlister	
Belfast	Belfast City Hospital	Grace Lavery	
Belfast	Belfast City Hospital	Karen Allen (nee Parsons)	
Belfast	Belfast City Hospital	Sara McCusker(nee Stokes)	
Belfast	Belfast City Hospital	Sharon McClean	

Belfast	Belfast City Hospital	Sophie Lynch	
Belfast	Belfast City Hospital	Aishleen Brunton	
Belfast	Belfast City Hospital	Angela Morrison	
Belfast	Belfast City Hospital	Angela Rosbotham	
Belfast	Belfast City Hospital	Barbara Harvey	
Belfast	Belfast City Hospital	Eimear Henry	
Belfast	Belfast City Hospital	Emma Hanna	
Belfast	Belfast City Hospital	Geraldine Douris	
Belfast	Belfast City Hospital	Joanne Todd	
Belfast	Belfast City Hospital	Mairead Devine	
Belfast	Belfast City Hospital	Naomi Hill	
Belfast	Belfast City Hospital	Ruth Boyd	
Belfast	Belfast City Hospital	Sharon Hynds	
Belfast	Belfast City Hospital	Shirley McKenna	
Belfast	Belfast City Hospital	Joe O'Sullivan	PI
Bellinzona	Istituto Oncologico della Svizzera Italiana	Gianfranco Pesce	
Bellinzona	Istituto Oncologico della Svizzera Italiana	Maria Delgrande	
Bellinzona	Istituto Oncologico della Svizzera Italiana	Ngwa Che Azinwi	
Bellinzona	Istituto Oncologico della Svizzera Italiana	Ricardo Pereira Mestre	PI
Bellinzona	Istituto Oncologico della Svizzera Italiana	Vittoria Espeli	
Bellinzona	Istituto Oncologico della Svizzera Italiana	Carolina De Almeida	
Bellinzona	Istituto Oncologico della Svizzera Italiana	Eloise Kremer	
Berne	Inselspital (University Hospital Berne)	Timo Nannen	
Berne	Inselspital (University Hospital Berne)	Beat Roth	
Berne	Inselspital (University Hospital Berne)	Simone Rimoldi	

Berne	Inselspital (University Hospital Berne)	Susan Meierhans	
Berne	Inselspital (University Hospital Berne)	Anselm Lafita	
Berne	Inselspital (University Hospital Berne)	Anna-Katharina Herrmann	
Berne	Inselspital (University Hospital Berne)	Antje Ulrich	
Berne	Inselspital (University Hospital Berne)	Barbara Uhlmann	
Berne	Inselspital (University Hospital Berne)	Eloise Kremer	
Berne	Inselspital (University Hospital Berne)	Kathi Ochsner	
Berne	Inselspital (University Hospital Berne)	Daniel Aebersold	
Berne	Inselspital (University Hospital Berne)	Jörg Beyer	PI
Berne	Inselspital (University Hospital Berne)	George Thalmann	
Biel	Spitalzentrum Biel	Annette Winkler Vatter	
Biel	Spitalzentrum Biel	Béatrice Zimmerli Schwab	
Biel	Spitalzentrum Biel	Eloise Kremer	
Biel	Spitalzentrum Biel	Silvia Hanselmann	
Biel	Spitalzentrum Biel	Markus Borner	PI
Birmingham	Queen Elizabeth Hospital (Birmingham)	Amarpal Bains	
Birmingham	Queen Elizabeth Hospital (Birmingham)	Arvind Tripathy	
Birmingham	Queen Elizabeth Hospital (Birmingham)	Daniel Henderson	
Birmingham	Birmingham Heartlands Hospital	Frances Shaw	
Birmingham	Queen Elizabeth Hospital (Birmingham)	Jay Ansari	
Birmingham	City Hospital (Birmingham)	Lalit Pallan	
Birmingham	City Hospital (Birmingham)	Robert Stevenson	Co-I
Birmingham	City Hospital (Birmingham)	Sachin Trivedi	Co-I
Birmingham	Queen Elizabeth Hospital (Birmingham)	Sameed Hussain	
Birmingham	Queen Elizabeth Hospital (Birmingham)	Sudha Karanam	

Birmingham	Queen Elizabeth Hospital (Birmingham)	Syed Tirmazy	
Birmingham	Queen Elizabeth Hospital (Birmingham)	Abel Zachariah	
Birmingham	Birmingham Heartlands Hospital	Anjali Zarkar	PI
Birmingham	Queen Elizabeth Hospital (Birmingham)	Anjali Zarkar	
Birmingham	City Hospital (Birmingham)	Daniel Ford	
Birmingham	Queen Elizabeth Hospital (Birmingham)	Daniel Ford	Co-I
Birmingham	Queen Elizabeth Hospital (Birmingham)	David Fackrell	PI
Birmingham	City Hospital (Birmingham)	Emilio Porfiri	PI
Birmingham	Queen Elizabeth Hospital (Birmingham)	Emilio Porfiri	Co-I
Birmingham	Queen Elizabeth Hospital (Birmingham)	Erica Beaumont	
Birmingham	Queen Elizabeth Hospital (Birmingham)	Hannah Tween	
Birmingham	Queen Elizabeth Hospital (Birmingham)	Jenny Pascoe	
Birmingham	Queen Elizabeth Hospital (Birmingham)	Atiqa Ahmed	
Birmingham	Queen Elizabeth Hospital (Birmingham)	Charlotte Sabine	
Birmingham	Queen Elizabeth Hospital (Birmingham)	Charlotte Trinham	
Birmingham	Queen Elizabeth Hospital (Birmingham)	Daniella Lynch	
Birmingham	Queen Elizabeth Hospital (Birmingham)	Keia Spooner	
Birmingham	City Hospital (Birmingham)	Laura Butler	
Birmingham	Queen Elizabeth Hospital (Birmingham)	Laura Butler	
Birmingham	Queen Elizabeth Hospital (Birmingham)	Laura Caley	
Birmingham	Queen Elizabeth Hospital (Birmingham)	Rosie Henvey	
Birmingham	Birmingham Heartlands Hospital	Samarah Haq	
Birmingham	Birmingham Heartlands Hospital	Sanya Shafiq	
Birmingham	Queen Elizabeth Hospital (Birmingham)	Sibil Fernandez	
Birmingham	Queen Elizabeth Hospital (Birmingham)	Stephanie Palmer	

Birmingham	Queen Elizabeth Hospital (Birmingham)	Zhane Peterkin	
Birmingham	Queen Elizabeth Hospital (Birmingham)	Andrew Palmer	
Birmingham	Queen Elizabeth Hospital (Birmingham)	Biruk Asfaw	
Birmingham	Queen Elizabeth Hospital (Birmingham)	Christopher McGhee	
Birmingham	Queen Elizabeth Hospital (Birmingham)	Fahd Niaz	
Birmingham	Birmingham Heartlands Hospital	Michael Tarn	
Birmingham	Queen Elizabeth Hospital (Birmingham)	Richard Winter	
Birmingham	Queen Elizabeth Hospital (Birmingham)	Simon Horley	
Birmingham	City Hospital (Birmingham)	Steven Shanu	Pharmacist
Birmingham	Birmingham Heartlands Hospital	Adrian Kelly	
Birmingham	City Hospital (Birmingham)	Brian Gammon	
Birmingham	Birmingham Heartlands Hospital	Chen Bartlett	Pharmacist
Birmingham	Birmingham Heartlands Hospital	James Whitehouse	
Birmingham	Queen Elizabeth Hospital (Birmingham)	Vijay Patel	
Birmingham	Queen Elizabeth Hospital (Birmingham)	Vishy Veeranna	
Birmingham	Queen Elizabeth Hospital (Birmingham)	Aliyah Mannan	
Birmingham	City Hospital (Birmingham)	Amy Orme	
Birmingham	City Hospital (Birmingham)	Debbie Devonport	
Birmingham	Queen Elizabeth Hospital (Birmingham)	Fiona Evans	
Birmingham	Queen Elizabeth Hospital (Birmingham)	Golaleh McGinnell	
Birmingham	Queen Elizabeth Hospital (Birmingham)	Gullinder Jokhi	
Birmingham	Queen Elizabeth Hospital (Birmingham)	Karen Tester	
Birmingham	Queen Elizabeth Hospital (Birmingham)	Kathryn Adams	
Birmingham	Queen Elizabeth Hospital (Birmingham)	Lea Booth	
Birmingham	Birmingham Heartlands Hospital	Madhura Chandrashekara	

Birmingham	Queen Elizabeth Hospital (Birmingham)	Michelle Bates
Birmingham	Queen Elizabeth Hospital (Birmingham)	Nasreen Akhtar
Birmingham	Queen Elizabeth Hospital (Birmingham)	Nicola Betteridge
Birmingham	Queen Elizabeth Hospital (Birmingham)	Parminder Sohal
Birmingham	Birmingham Heartlands Hospital	Ellen Drew
Birmingham	City Hospital (Birmingham)	Joanne Dasgin
Birmingham	Queen Elizabeth Hospital (Birmingham)	Pamela Jones
Birmingham	City Hospital (Birmingham)	Alice Longe
Birmingham	Queen Elizabeth Hospital (Birmingham)	Alice Longe
Birmingham	Queen Elizabeth Hospital (Birmingham)	Alison Grant
Birmingham	Queen Elizabeth Hospital (Birmingham)	Amanda Davies
Birmingham	Queen Elizabeth Hospital (Birmingham)	Amisha Desai
Birmingham	Queen Elizabeth Hospital (Birmingham)	Amna Shah
Birmingham	Birmingham Heartlands Hospital	Ann Schumacher
Birmingham	Birmingham Heartlands Hospital	Arlene Oldan
Birmingham	Queen Elizabeth Hospital (Birmingham)	Emma Bruce
Birmingham	Queen Elizabeth Hospital (Birmingham)	Hannah Tolson
Birmingham	Queen Elizabeth Hospital (Birmingham)	Heather Jones
Birmingham	Queen Elizabeth Hospital (Birmingham)	Helen Jones
Birmingham	Queen Elizabeth Hospital (Birmingham)	Helen Preston
Birmingham	Birmingham Heartlands Hospital	Janet Prentice
Birmingham	City Hospital (Birmingham)	Jasbinder Kaur
Birmingham	Queen Elizabeth Hospital (Birmingham)	Jenny Hiley
Birmingham	Birmingham Heartlands Hospital	Jill Lyons
Birmingham	Birmingham Heartlands Hospital	Julia Sampson

Birmingham	Birmingham Heartlands Hospital	Kamaldeep Ajimal	
Birmingham	Queen Elizabeth Hospital (Birmingham)	Lisa Thomas	
Birmingham	Birmingham Heartlands Hospital	Lisa - Marie Brueton	
Birmingham	Queen Elizabeth Hospital (Birmingham)	Mahmoda Begum	
Birmingham	Queen Elizabeth Hospital (Birmingham)	Maria Bandeira	
Birmingham	Queen Elizabeth Hospital (Birmingham)	Nicola Anderson	
Birmingham	Queen Elizabeth Hospital (Birmingham)	Salma Afzal	
Birmingham	Queen Elizabeth Hospital (Birmingham)	Sam Hopkins (nee Poole)	
Birmingham	Queen Elizabeth Hospital (Birmingham)	Sara Diffley	
Birmingham	Queen Elizabeth Hospital (Birmingham)	Shaleen Bishop	
Birmingham	Queen Elizabeth Hospital (Birmingham)	Sharon Holmes	
Birmingham	Queen Elizabeth Hospital (Birmingham)	Tracy Soulsby	
Birmingham	Queen Elizabeth Hospital (Birmingham)	Trish Brady	
Birmingham	Birmingham Heartlands Hospital	Alison Maidment	Pharmacist
Birmingham	City Hospital (Birmingham)	Angela Williams	
Birmingham	Queen Elizabeth Hospital (Birmingham)	Claire Brown	
Birmingham	Queen Elizabeth Hospital (Birmingham)	Claire Draycott	
Birmingham	Queen Elizabeth Hospital (Birmingham)	Fiona Catherine Stead	
Birmingham	Queen Elizabeth Hospital (Birmingham)	Fiona Catherine Stead	
Birmingham	Queen Elizabeth Hospital (Birmingham)	Gemma Cole	
Birmingham	City Hospital (Birmingham)	Harriet Goddard	
Birmingham	Queen Elizabeth Hospital (Birmingham)	Helen Clarke	
Birmingham	Queen Elizabeth Hospital (Birmingham)	Jane Cook	
Birmingham	Queen Elizabeth Hospital (Birmingham)	Joanna Gray (nee Finney)	
Birmingham	City Hospital (Birmingham)	Julie Simpson	Pharmacist

Birmingham	City Hospital (Birmingham)	Marion Tatman	
Birmingham	Birmingham Heartlands Hospital	Mary (Ellen) Drew	
Birmingham	Birmingham Heartlands Hospital	Penny Goodby (nee Harbach	
Birmingham	Queen Elizabeth Hospital (Birmingham)	Rosemarie Seadon	Pharmacist
Birmingham	City Hospital (Birmingham)	Yin May Chin	
Birmingham	Queen Elizabeth Hospital (Birmingham)	Nicholas James	PI
Blackburn	Royal Blackburn Hospital	Ahmed Salah	
Blackburn	Royal Blackburn Hospital	Alexandra Ferrera	
Blackburn	Royal Blackburn Hospital	Andrew Brocklehurst	
Blackburn	Royal Blackburn Hospital	Anthea Cree	
Blackburn	Royal Blackburn Hospital	Danya Abdulwahid	
Blackburn	Royal Blackburn Hospital	Ilyas Ahmed	
Blackburn	Royal Blackburn Hospital	Jasima Latif	
Blackburn	Royal Blackburn Hospital	Jennifer King	
Blackburn	Royal Blackburn Hospital	Karan Patel	
Blackburn	Royal Blackburn Hospital	Omi Parikh	PI
Blackburn	Royal Blackburn Hospital	Parth Desai	
Blackburn	Royal Blackburn Hospital	Prasad Kellati	
Blackburn	Royal Blackburn Hospital	Richard Walshaw	
Blackburn	Royal Blackburn Hospital	Sophia Callaghan	
Blackburn	Royal Blackburn Hospital	Sophie Raby	Co-I
Blackburn	Royal Blackburn Hospital	Twesige Mugisa	
Blackburn	Royal Blackburn Hospital	William Croxford	
Blackburn	Royal Blackburn Hospital	Zhu Oong	Co-I
Blackburn	Royal Blackburn Hospital	Ajay Mehta	

Blackburn	Royal Blackburn Hospital	Deborah Williamson	
Blackburn	Royal Blackburn Hospital	Falalu Danwata	
Blackburn	Royal Blackburn Hospital	Graham Read	
Blackburn	Royal Blackburn Hospital	Imran Haidar	
Blackburn	Royal Blackburn Hospital	Marcus Wise	
Blackburn	Royal Blackburn Hospital	Ruth Conroy	
Blackburn	Royal Blackburn Hospital	Tanmay Mukhopadhyay	
Blackburn	Royal Blackburn Hospital	Win Soe	
Blackburn	Royal Blackburn Hospital	Zia Rehman	
Blackburn	Royal Blackburn Hospital	Rizwana Hussain	Pharmacist
Blackburn	Royal Blackburn Hospital	Ana Batista	Pharmacist
Blackburn	Royal Blackburn Hospital	Farrah Burrows	
Blackburn	Royal Blackburn Hospital	Fatima Butt	
Blackburn	Royal Blackburn Hospital	Humairaa Timol	
Blackburn	Royal Blackburn Hospital	Naomi Charlton	
Blackburn	Royal Blackburn Hospital	Bethany Fielding	
Blackburn	Royal Blackburn Hospital	Andrew Hunnisett	
Blackburn	Royal Blackburn Hospital	Andrew Lancaster	
Blackburn	Royal Blackburn Hospital	Darren Rusk	Pharmacist
Blackburn	Royal Blackburn Hospital	James Grunshaw	
Blackburn	Royal Blackburn Hospital	Joseph Dykes	
Blackburn	Royal Blackburn Hospital	Matthew Lovell	
Blackburn	Royal Blackburn Hospital	Nicholas Pounder	
Blackburn	Royal Blackburn Hospital	Stephen Kilroy	
Blackburn	Royal Blackburn Hospital	Dayle Squires	

Blackburn	Royal Blackburn Hospital	Hani Hanna	Pharmacist
Blackburn	Royal Blackburn Hospital	Alison Blackburn	
Blackburn	Royal Blackburn Hospital	Deborah Smith	
Blackburn	Royal Blackburn Hospital	Farzana Patel	
Blackburn	Burnley General Hospital	Jacqueline Thomas	
Blackburn	Royal Blackburn Hospital	Jacqueline Thomas	
Blackburn	Royal Blackburn Hospital	Kathryn Hayes	
Blackburn	Burnley General Hospital	Lynsey Waring	
Blackburn	Royal Blackburn Hospital	Lynsey Waring	
Blackburn	Royal Blackburn Hospital	Angela Hugill	
Blackburn	Royal Blackburn Hospital	Helen Frankland	
Blackburn	Royal Blackburn Hospital	Janet Ryan-Smith	
Blackburn	Royal Blackburn Hospital	Sue Ashworth	
Blackburn	Royal Blackburn Hospital	Alexandra McCarrick	
Blackburn	Royal Blackburn Hospital	Christina Robinson	
Blackburn	Royal Blackburn Hospital	Debbie Sutton	Pharmacist
Blackburn	Royal Blackburn Hospital	Gaynor Bowen	
Blackburn	Royal Blackburn Hospital	Jackie Carey	
Blackburn	Royal Blackburn Hospital	Jackie Nuttall	
Blackburn	Royal Blackburn Hospital	Jan Flaherty	
Blackburn	Royal Blackburn Hospital	Jennifer McCallum	
Blackburn	Royal Blackburn Hospital	Jenny Cockerill-Taylor	
Blackburn	Royal Blackburn Hospital	Jessica Whiston	
Blackburn	Royal Blackburn Hospital	Marianna Theodoulou	
Blackburn	Royal Blackburn Hospital	Maricica Zabrautanu	

Blackburn	Royal Blackburn Hospital	Philippa Springle	
Blackburn	Royal Blackburn Hospital	Rachel Bolton	
Blackburn	Royal Blackburn Hospital	Samatha Guy	
Blackburn	Royal Blackburn Hospital	Sarah Ainsworth	
Blackburn	Burnley General Hospital	Sarah Keith	
Blackburn	Royal Blackburn Hospital	Sarah Keith	
Blackburn	Royal Blackburn Hospital	Tracey Kilduff	
Blackburn	Royal Blackburn Hospital	Victoria Taylor	Pharmacist
Blackburn	Royal Blackburn Hospital	Diane Forrest	
Blackburn	Royal Blackburn Hospital	Hazel Aston	
Blackburn	Royal Blackburn Hospital	Helene Chorley	
Blackburn	Royal Blackburn Hospital	Jeanette Hargreaves	
Blackburn	Royal Blackburn Hospital	Karen Beard	
Blackburn	Royal Blackburn Hospital	Karen Jewers	
Blackburn	Royal Blackburn Hospital	Vivienne Tickle	
Bolton	Royal Bolton Hospital	Ajay Mehta	
Bolton	Royal Bolton Hospital	Tony Elliott	
Bolton	Royal Bolton Hospital	Karen Lee	Pharmacist
Bolton	Royal Bolton Hospital	Gillian Mobb	
Bolton	Royal Bolton Hospital	Ling Lee	PI
Bolton	Royal Bolton Hospital	Hemant Patel	
Bolton	Royal Bolton Hospital	Michael Pantelides	
Bolton	Royal Bolton Hospital	Richard Jones	
Bolton	Royal Bolton Hospital	Robert Hull	
Bolton	Royal Bolton Hospital	Janet Keegan	

Bolton	Royal Bolton Hospital	Shirley Cocks	
Bolton	Royal Bolton Hospital	Charlotte Lever	
Bolton	Royal Bolton Hospital	Debbie Forkin	
Bolton	Royal Bolton Hospital	Janine Hurst	
Bolton	Royal Bolton Hospital	Julie Chadwick	
Bolton	Royal Bolton Hospital	Louise Dawson	
Bolton	Royal Bolton Hospital	Raksha Mistry	
Bolton	Royal Bolton Hospital	Sally Shaw	Pharmacist
Bolton	Royal Bolton Hospital	Zoe Gall	
Bolton	Royal Bolton Hospital	Collette Hunt	Pharmacist
Bolton	Royal Bolton Hospital	Karen Jewers	
Bolton	Royal Bolton Hospital	Lindsay Rawlinson	Pharmacist
Boston	Pilgrim Hospital	Ana Fernandez-Ots	Co-I
Boston	Pilgrim Hospital	Andrew Sloan	
Boston	Pilgrim Hospital	Christian Arias	Co-I
Boston	Pilgrim Hospital	David Ballesteros-Quintail	Co-I
Boston	Pilgrim Hospital	Sekar (DV) Kittappa	Co-I
Boston	Pilgrim Hospital	Sindhu Ramamurthy	
Boston	Pilgrim Hospital	Miguel Panades	PI
Boston	Pilgrim Hospital	Prantik Das	
Boston	Pilgrim Hospital	Eileen Busby	
Boston	Pilgrim Hospital	Gunjan Phalod	
Boston	Pilgrim Hospital	Karen Metcalf	
Boston	Pilgrim Hospital	Sally Ann Molsher	
Boston	Pilgrim Hospital	Andrew Judd	

Boston	Pilgrim Hospital	Simon Archer	
Boston	Pilgrim Hospital	Beverley Mashegede	
Boston	Pilgrim Hospital	Isobel Thomas	
Boston	Pilgrim Hospital	Kimberley Netherton	
Boston	Pilgrim Hospital	Kinga Szymiczek	
Boston	Pilgrim Hospital	Laura Walsh	
Boston	Pilgrim Hospital	Kerry Pettitt	
Boston	Pilgrim Hospital	Victoria Knight (n. Sherburn)	
Boston	Pilgrim Hospital	Amanda Roper	
Boston	Pilgrim Hospital	Amy Kirkby	
Boston	Pilgrim Hospital	Giuseppe Banna	
Boston	Pilgrim Hospital	Helen Carolan	
Boston	Pilgrim Hospital	Helen Palmer	Pharmacist
Boston	Pilgrim Hospital	Jayne Borley	Pharmacist
Boston	Pilgrim Hospital	Jenny Salmon	
Boston	Pilgrim Hospital	Jo Fletcher	
Boston	Pilgrim Hospital	Rebecca Spencer	Pharmacist
Boston	Pilgrim Hospital	Tara Lawrence nee Palmer	
Boston	Pilgrim Hospital	Alice Latty	
Boston	Pilgrim Hospital	Anita Young	
Boston	Pilgrim Hospital	Bryony Saint	
Boston	Pilgrim Hospital	Carol Lockwood	
Boston	Pilgrim Hospital	Trish Tsuro	
Bournemouth	Royal Bournemouth Hospital	Kenneth Oguejiofor	
Bournemouth	Royal Bournemouth Hospital	Matthew Roberts	

Bournemouth	Royal Bournemouth Hospital	Deborah Hands	
Bournemouth	Royal Bournemouth Hospital	George Astras	
Bournemouth	Royal Bournemouth Hospital	Joe Davies	
Bournemouth	Royal Bournemouth Hospital	Rao Vuyyuru	
Bournemouth	Royal Bournemouth Hospital	Sue Brock	PI
Bournemouth	Royal Bournemouth Hospital	Tom Geldart	Co-I
Bournemouth	Royal Bournemouth Hospital	Julie Thomson	
Bournemouth	Royal Bournemouth Hospital	Laura Purandare	
Bournemouth	Royal Bournemouth Hospital	Mirela Mukaj	
Bournemouth	Royal Bournemouth Hospital	Natasha Ottley	
Bournemouth	Royal Bournemouth Hospital	Taslima Rabbi	
Bournemouth	Royal Bournemouth Hospital	Alison Hogan	Pharmacist
Bournemouth	Royal Bournemouth Hospital	Rachel Bower	
Bournemouth	Royal Bournemouth Hospital	Ben Loat	
Bournemouth	Royal Bournemouth Hospital	Cameron Huck	
Bournemouth	Royal Bournemouth Hospital	David Chrastek	
Bournemouth	Royal Bournemouth Hospital	Joseph Cavill	
Bournemouth	Royal Bournemouth Hospital	Luke Vamplew	
Bournemouth	Royal Bournemouth Hospital	Carlton Rowlands	
Bournemouth	Royal Bournemouth Hospital	Roger Hudson	
Bournemouth	Royal Bournemouth Hospital	Emma Sharland	
Bournemouth	Royal Bournemouth Hospital	Joanne Sheppard	
Bournemouth	Royal Bournemouth Hospital	Cathie Purnell	
Bournemouth	Royal Bournemouth Hospital	Linda Purandare	
Bournemouth	Royal Bournemouth Hospital	Min Wu	

Bournemouth	Royal Bournemouth Hospital	Katherine Major	
Bournemouth	Royal Bournemouth Hospital	Lynsey Houlton	
Bournemouth	Royal Bournemouth Hospital	Rebecca Miln	
Bournemouth	Royal Bournemouth Hospital	Sarah Savage	
Bournemouth	Royal Bournemouth Hospital	Sophie Jackson	
Bournemouth	Royal Bournemouth Hospital	Stephanie Jones	
Bournemouth	Royal Bournemouth Hospital	Tiffany Joyce	
Bournemouth	Royal Bournemouth Hospital	Eve Broadley	
Bournemouth	Royal Bournemouth Hospital	Kate Preece	
Bournemouth	Royal Bournemouth Hospital	Natalya Boyd	
Bradford	Bradford Royal Infirmary	Adel Jebar	Co-I
Bradford	Bradford Royal Infirmary	Andrew Viggars	Co-I
Bradford	Bradford Royal Infirmary	Ann Henry	
Bradford	Bradford Royal Infirmary	Anthi Zeniou	
Bradford	Bradford Royal Infirmary	Catherine Handforth	
Bradford	Bradford Royal Infirmary	Charlotte Richardson	
Bradford	Bradford Royal Infirmary	Christopher Williams	
Bradford	Bradford Royal Infirmary	Eldho Joseph	Co-I
Bradford	Bradford Royal Infirmary	Emily Montague	
Bradford	Bradford Royal Infirmary	Finbar Slevin	
Bradford	Bradford Royal Infirmary	Ian Boon	
Bradford	Bradford Royal Infirmary	Jessica Pearce	Co-I
Bradford	Bradford Royal Infirmary	Leila Koudsi	
Bradford	Bradford Royal Infirmary	Louise Karsera	Co-I
Bradford	Bradford Royal Infirmary	Love Goyal	

Bradford	Bradford Royal Infirmary	Lucy Jones	Co-I
Bradford	Bradford Royal Infirmary	Lucy Ward	
Bradford	Bradford Royal Infirmary	Manjusha Soni	Co-I
Bradford	Bradford Royal Infirmary	Reem Mahmood	
Bradford	Bradford Royal Infirmary	Sally Martin	Co-I
Bradford	Bradford Royal Infirmary	Samuel Briggs	
Bradford	Bradford Royal Infirmary	Simon Brown	PI
Bradford	Bradford Royal Infirmary	Sohail Mughal	Co-I
Bradford	Bradford Royal Infirmary	Sree Rodda	
Bradford	Bradford Royal Infirmary	Carmel Loughrey	
Bradford	Bradford Royal Infirmary	Dan Lee	
Bradford	Bradford Royal Infirmary	Ganesan Jeyasangar	
Bradford	Bradford Royal Infirmary	Jamal Zekri	
Bradford	Bradford Royal Infirmary	Sue Cheeseman	
Bradford	St Luke's (Bradford)	Susan Cheeseman	
Bradford	Bradford Royal Infirmary	You Yone	
Bradford	Bradford Royal Infirmary	Eleanor Waldron	
Bradford	Bradford Royal Infirmary	Jannika Lazarte	
Bradford	Bradford Royal Infirmary	Lucille Kenyon	
Bradford	Bradford Royal Infirmary	Qamar Akbar	
Bradford	Bradford Royal Infirmary	Declan Ryan-Wakeling	
Bradford	Bradford Royal Infirmary	Kelvin Stewart	
Bradford	Bradford Royal Infirmary	Leslie Masters	
Bradford	Bradford Royal Infirmary	Umair Hamid	Pharmacist
Bradford	Bradford Royal Infirmary	Mohammed Patel	

Bradford	Bradford Royal Infirmary	Osman Chohan	Pharmacist
Bradford	Bradford Royal Infirmary	Richard Benton	
Bradford	Bradford Royal Infirmary	Carol Firth	
Bradford	Bradford Royal Infirmary	Dawn McNulty	
Bradford	Bradford Royal Infirmary	Helen Wilson	
Bradford	Bradford Royal Infirmary	Kay Cockroft	
Bradford	Bradford Royal Infirmary	Manitha Vinod	
Bradford	Bradford Royal Infirmary	Wendy Cardozo	
Bradford	Bradford Royal Infirmary	Hayley Inman	
Bradford	Bradford Royal Infirmary	Jane Sewell	
Bradford	Bradford Royal Infirmary	Sarah Tinker	
Bradford	Bradford Royal Infirmary	Chandran Nallathambi	
Bradford	Bradford Royal Infirmary	Charlotte Johnson-Smith	
Bradford	Bradford Royal Infirmary	Elizabeth McIntosh	
Bradford	Bradford Royal Infirmary	Gail Opio-Te	
Bradford	Bradford Royal Infirmary	Jacqueline Quantrill	
Bradford	Bradford Royal Infirmary	Kim Storton	
Bradford	Bradford Royal Infirmary	Robina Ghulam	
Bradford	Bradford Royal Infirmary	Shefali Parikh	
Bradford	Bradford Royal Infirmary	Susan Shorter	
Bradford	Bradford Royal Infirmary	Victoria Drew	
Bradford	Bradford Royal Infirmary	Anne Marie Kay	
Bradford	Bradford Royal Infirmary	Laura Jaques	
Bradford	Bradford Royal Infirmary	Linda Bamford	
Bradford	Bradford Royal Infirmary	Sophia Khan	

Bradford	Bradford Royal Infirmary	Sophie Stephenson	
Brighton	Royal Sussex County Hospital	Ashok Nikapota	
Brighton	Royal Sussex County Hospital	Aun Mohammad	
Brighton	Royal Sussex County Hospital	George Plantaniotis	
Brighton	Royal Sussex County Hospital	Angus Robinson	PI
Brighton	Royal Sussex County Hospital	Daniel Henderson	
Brighton	Royal Sussex County Hospital	David Bloomfield	
Brighton	Royal Sussex County Hospital	George Devtsch	
Brighton	Royal Sussex County Hospital	George Plataniotis	Co-I
Brighton	Royal Sussex County Hospital	Jackie Sham	
Brighton	Royal Sussex County Hospital	Marie Wilkins	
Brighton	Royal Sussex County Hospital	Tarun Durga	
Brighton	Royal Sussex County Hospital	Vivien Tse	
Brighton	Royal Sussex County Hospital	Angela Man	
Brighton	Royal Sussex County Hospital	Catherine Hunter	
Brighton	Royal Sussex County Hospital	Chritianne Whitfield	
Brighton	Royal Sussex County Hospital	Lisa Furnival	
Brighton	Royal Sussex County Hospital	Rachel Rose Edmunds	
Brighton	Royal Sussex County Hospital	Samantha Hodges	
Brighton	Royal Sussex County Hospital	Summer Ibrahim	
Brighton	Royal Sussex County Hospital	Jean Tremlett	
Brighton	Royal Sussex County Hospital	Andrew Hart	
Brighton	Royal Sussex County Hospital	Matthew Seal	
Brighton	Royal Sussex County Hospital	Paul Frattaroli	
Brighton	Royal Sussex County Hospital	Sebastien Martin	

Brighton	Royal Sussex County Hospital	Tiago Rodrigues	
Brighton	Royal Sussex County Hospital	Simon Matthews	
Brighton	Royal Sussex County Hospital	Stephen Brown	
Brighton	Royal Sussex County Hospital	Caroline Walker	
Brighton	Royal Sussex County Hospital	Dorota Bak-Blaz	
Brighton	Royal Sussex County Hospital	Karen Walker	
Brighton	Royal Sussex County Hospital	Emma Foreman	Pharmacist
Brighton	Royal Sussex County Hospital	Alison Porges	
Brighton	Royal Sussex County Hospital	Amy Murray	
Brighton	Royal Sussex County Hospital	Annie Oliver	
Brighton	Royal Sussex County Hospital	Elizabeth Corbett	
Brighton	Royal Sussex County Hospital	Jane Peterson	
Brighton	Royal Sussex County Hospital	Joanne Magennis	
Brighton	Royal Sussex County Hospital	Jodie Smith	
Brighton	Royal Sussex County Hospital	Katie Langford	
Brighton	Royal Sussex County Hospital	Lucy Curtis	
Brighton	Royal Sussex County Hospital	Poppy Lavender	
Brighton	Royal Sussex County Hospital	Ranee Lactao	
Brighton	Royal Sussex County Hospital	Bobbie Yoong	
Brighton	Royal Sussex County Hospital	Elaine Noon	
Brighton	Royal Sussex County Hospital	Helen Mitchell	
Brighton	Royal Sussex County Hospital	Jane Dexter	
Brighton	Royal Sussex County Hospital	Jane Hanson	
Brighton	Royal Sussex County Hospital	Julie Smith	
Brighton	Royal Sussex County Hospital	Kirsty Bracewell	

Brighton	Royal Sussex County Hospital	Lisa Barrott	
Brighton	Royal Sussex County Hospital	Maggie Cole	
Brighton	Royal Sussex County Hospital	Monika Musiol	
Brighton	Royal Sussex County Hospital	Pauline Martin	
Brighton	Royal Sussex County Hospital	Sue Trotter	
Brighton	Royal Sussex County Hospital	Tamsin Kent	
Brighton	Royal Sussex County Hospital	Tenesa Sargent	
Brighton	Royal Sussex County Hospital	Victoria Sellick	
Bristol	Bristol Haematology & Oncology Centre	Amit Bahl	PI
Bristol	Bristol Haematology & Oncology Centre	Chris Herbert	
Bristol	Bristol Haematology & Oncology Centre	Emily Foulstone	
Bristol	Bristol Haematology & Oncology Centre	Hugh Newman	
Bristol	Bristol Haematology & Oncology Centre	Jyothsna Chennupati	
Bristol	Bristol Haematology & Oncology Centre	Mark Beresford	
Bristol	Bristol Haematology & Oncology Centre	Paula Wilson	
Bristol	Bristol Haematology & Oncology Centre	Serena Hilman	
Bristol	Bristol Haematology & Oncology Centre	Susan Masson	
Bristol	Bristol Haematology & Oncology Centre	Eve Watson	
Bristol	Bristol Haematology & Oncology Centre	Kay Drury	
Bristol	Bristol Haematology & Oncology Centre	Azeem Arshad	
Bristol	Bristol Haematology & Oncology Centre	Nick Robins	
Bristol	Bristol Haematology & Oncology Centre	Sibusiso Dhladhla	
Bristol	Bristol Haematology & Oncology Centre	Harvey Dymond	
Bristol	Bristol Haematology & Oncology Centre	Ian Penwarden	Pharmacist
Bristol	Bristol Haematology & Oncology Centre	Lloyd Abood	

Bristol	Bristol Haematology & Oncology Centre	Marc Coe	
Bristol	Bristol Haematology & Oncology Centre	Robert Hollister	
Bristol	Bristol Haematology & Oncology Centre	Stephen Lang	
Bristol	Bristol Haematology & Oncology Centre	Tristan Grey	
Bristol	Bristol Haematology & Oncology Centre	Shalini Mohan	
Bristol	Bristol Haematology & Oncology Centre	Beth Thorne	
Bristol	Bristol Haematology & Oncology Centre	Amy Holloway	Pharmacist
Bristol	Bristol Haematology & Oncology Centre	Bryony Parrish	
Bristol	Bristol Haematology & Oncology Centre	Helen Saldanha	
Bristol	Bristol Haematology & Oncology Centre	Hayley Jones	
Bristol	Bristol Haematology & Oncology Centre	Jayne Leonard	
Bristol	Bristol Haematology & Oncology Centre	Kimberly Rockley	
Bristol	Bristol Haematology & Oncology Centre	Lindsay Ball	Pharmacist
Bristol	Bristol Royal Infirmary	Lindsay Ball	
Bristol	Bristol Haematology & Oncology Centre	Polly Gingell	
Bristol	Bristol Haematology & Oncology Centre	Sally-Ann Hall	Pharmacist
Bristol	Bristol Haematology & Oncology Centre	Sandra Williams (nee Price)	Pharmacist
Bristol	Bristol Haematology & Oncology Centre	Sarah Bishop	
Bristol	Bristol Haematology & Oncology Centre	Seonaid Wright	
Bristol	Bristol Haematology & Oncology Centre	Sharon Short	
Burnley	Burnley General Hospital	Ahmed Salah	
Burnley	Burnley General Hospital	Andrew Brocklehurst	
Burnley	Burnley General Hospital	Anthea Cree	
Burnley	Burnley General Hospital	Danya Abdulwahid	
Burnley	Burnley General Hospital	Ilyas Ahmed	

Burnley	Burnley General Hospital	Karan Patel	
Burnley	Burnley General Hospital	Omi Parikh	PI
Burnley	Burnley General Hospital	Prasad Kellati	
Burnley	Burnley General Hospital	Richard Walshaw	
Burnley	Burnley General Hospital	Twesige Mugisa	
Burnley	Burnley General Hospital	Deborah Williamson	
Burnley	Burnley General Hospital	Imran Haidar	
Burnley	Burnley General Hospital	Marcus Wise	
Burnley	Burnley General Hospital	Ruth Conroy	
Burnley	Burnley General Hospital	Tanmay Mukhopadhyay	
Burnley	Burnley General Hospital	Win Soe	
Burnley	Burnley General Hospital	Zia Rehman	
Burnley	Burnley General Hospital	Rizwana Hussain	Pharmacist
Burnley	Burnley General Hospital	Ana Batista	Pharmacist
Burnley	Burnley General Hospital	Fatima Butt	
Burnley	Burnley General Hospital	Humairaa Timol	
Burnley	Burnley General Hospital	Bethany Fielding	
Burnley	Burnley General Hospital	Darren Rusk	
Burnley	Burnley General Hospital	Matthew Lovell	
Burnley	Burnley General Hospital	Stephen Kilroy	
Burnley	Burnley General Hospital	Dayle Squires	
Burnley	Burnley General Hospital	Hani Hanna	Pharmacist
Burnley	Burnley General Hospital	Farzana Patel	
Burnley	Burnley General Hospital	Angela Hugill	
Burnley	Burnley General Hospital	Helen Frankland	

Burnley	Burnley General Hospital	Janet Ryan-Smith	
Burnley	Burnley General Hospital	Sue Ashworth	
Burnley	Burnley General Hospital	Alexandra McCarrick	
Burnley	Burnley General Hospital	Christina Robinson	
Burnley	Burnley General Hospital	Debbie Sutton	
Burnley	Burnley General Hospital	Gaynor Bowen	
Burnley	Burnley General Hospital	Jackie Carey	
Burnley	Burnley General Hospital	Jan Flaherty	
Burnley	Burnley General Hospital	Karen Riley	
Burnley	Royal Blackburn Hospital	Karen Riley	
Burnley	Burnley General Hospital	Philippa Springle	
Burnley	Burnley General Hospital	Samatha Guy	
Burnley	Burnley General Hospital	Sarah Ainsworth	
Burnley	Burnley General Hospital	Tracey Kilduff	
Burnley	Burnley General Hospital	Victoria Taylor	Pharmacist
Burnley	Burnley General Hospital	Diane Forrest	
Burnley	Burnley General Hospital	Helene Chorley	
Burnley	Burnley General Hospital	Jeanette Hargreaves	
Burnley	Burnley General Hospital	Karen Beard	
Burnley	Burnley General Hospital	Karen Jewers	
Burnley	Burnley General Hospital	Louise Dawson	
Burnley	Burnley General Hospital	Vivienne Tickle	
Burton upon Trent	Queen's Hospital Burton	Prabir Chakraborti	
Burton upon Trent	Queen's Hospital Burton	Rosemary Corfield	
Burton-on-Trent	Queen's Hospital Burton	Anita Szita	

Burton-on-Trent	Queen's Hospital Burton	Chandrani Mallik	
Burton-on-Trent	Queen's Hospital Burton	Rohit Malde	
Burton-on-Trent	Queen's Hospital Burton	Seheli Bandyopahdyay	
Burton-on-Trent	Queen's Hospital Burton	Sudipta Datta	
Burton-on-Trent	Queen's Hospital Burton	Chris Curtis	
Burton-on-Trent	Queen's Hospital Burton	Dakshinamoorthy Muthukumar	
Burton-on-Trent	Queen's Hospital Burton	Divya Ramadasan	
Burton-on-Trent	Queen's Hospital Burton	Hanine Medani	
Burton-on-Trent	Queen's Hospital Burton	Karzan Hama	
Burton-on-Trent	Queen's Hospital Burton	Mike Smith-Howell	PI
Burton-on-Trent	Queen's Hospital Burton	Rajeev Kaushal	
Burton-on-Trent	Queen's Hospital Burton	Shahzad Ahmed	
Burton-on-Trent	Queen's Hospital Burton	Shan Chetiyawardana	
Burton-on-Trent	Queen's Hospital Burton	V Gajek	
Burton-on-Trent	Queen's Hospital Burton	Pugazhenthi Pattu	
Burton-on-Trent	Queen's Hospital Burton	Ali Mahmmod	
Burton-on-Trent	Queen's Hospital Burton	Lorraine Carter	
Burton-on-Trent	Queen's Hospital Burton	Annette Fleet	
Burton-on-Trent	Queen's Hospital Burton	Elizabeth Kemp	
Burton-on-Trent	Queen's Hospital Burton	Jennifer Moyes	Pharmacist
Burton-on-Trent	Queen's Hospital Burton	Gill Bell	
Burton-on-Trent	Queen's Hospital Burton	Jo Burns	
Burton-on-Trent	Queen's Hospital Burton	Katy English (nee Parkes)	
Burton-on-Trent	Queen's Hospital Burton	Sarah Hathaway-Lees	
Burton-on-Trent	Queen's Hospital Burton	Ann Adams	Pharmacist

Burton-on-Trent	Queen's Hospital Burton	Clare Mewies	
Burton-on-Trent	Queen's Hospital Burton	Helen Cox	
Burton-on-Trent	Queen's Hospital Burton	Helena Cox	
Burton-on-Trent	Queen's Hospital Burton	Jacqueline Elliott	
Bury St Edmunds	West Suffolk Hospital	Alex Martin	Co-I
Bury St Edmunds	West Suffolk Hospital	Yvonne Rimmer	
Bury St Edmunds	West Suffolk Hospital	David Matter	
Bury St Edmunds	West Suffolk Hospital	Fred Tuck	
Bury St Edmunds	West Suffolk Hospital	John Raja Ravendar	
Bury St Edmunds	West Suffolk Hospital	Mark Heath	
Bury St Edmunds	West Suffolk Hospital	Amanda Neal	
Bury St Edmunds	West Suffolk Hospital	Jill Thain	
Bury St Edmunds	West Suffolk Hospital	Lisa Patterson	
Bury St Edmunds	West Suffolk Hospital	Rachel Stocking	
Bury St Edmunds	West Suffolk Hospital	Susan Hale	
Bury St Edmunds	West Suffolk Hospital	Yvonne Field	
Bury St Edmunds	West Suffolk Hospital	Deborah Clements-Dimmock	
Bury St Edmunds	West Suffolk Hospital	Frances Flynn	
Bury St Edmunds	West Suffolk Hospital	Joanne Kellett	
Bury St Edmunds	West Suffolk Hospital	Elizabeth Devoy	
Bury St Edmunds	West Suffolk Hospital	Gill Brett	
Bury St Edmunds	West Suffolk Hospital	Helen Small	
Bury St Edmunds	West Suffolk Hospital	Tracey Murray	
Bury St. Edmonds	West Suffolk Hospital	Cathryn Woodward	PI
Bury St. Edmunds	West Suffolk Hospital	James Curtis	Pharmacist

Bury St. Edmunds	West Suffolk Hospital	Cherri Blades	
Camarthen	Glangwili General (formerly West Wales General)	Mau-Don Phan	PI
Camarthen	Glangwili General (formerly West Wales General)	Sonya Goriah	Co-I
Camarthen	Glangwili General (formerly West Wales General)	Sandra Griffiths nee Evens	
Camarthen	Glangwili General (formerly West Wales General)	Bryan Phillips	
Camarthen	Glangwili General (formerly West Wales General)	Bleddyn Edwards	
Camarthen	Glangwili General (formerly West Wales General)	Meena Raj	
Camarthen	Glangwili General (formerly West Wales General)	Ann Hewins	
Camarthen	Glangwili General (formerly West Wales General)	Samantha Coetzee	
Camarthen	Glangwili General (formerly West Wales General)	Rocio Riba	Pharmacist
Camarthen	Glangwili General (formerly West Wales General)	Zohra Omar	
Cambridge	Addenbrooke's Hospital	Gin Lee	
Cambridge	Addenbrooke's Hospital	Andrew Styling	
Cambridge	Addenbrooke's Hospital	Hossameldin Attia	Co-I
Cambridge	Addenbrooke's Hospital	James Jones	
Cambridge	Addenbrooke's Hospital	James Tanner	
Cambridge	Addenbrooke's Hospital	Kamarul Zaki	
Cambridge	Addenbrooke's Hospital	Mirela Hategan	
Cambridge	Addenbrooke's Hospital	Nicola Thompson	
Cambridge	Addenbrooke's Hospital	Sian Pugh	
Cambridge	Addenbrooke's Hospital	Simon Pacey	
Cambridge	Addenbrooke's Hospital	Danish Mazhar	PI
Cambridge	Addenbrooke's Hospital	Han Wong	
Cambridge	Addenbrooke's Hospital	Luke Hughes-Davies	
Cambridge	Addenbrooke's Hospital	Richard Benson	

Cambridge	Addenbrooke's Hospital	Kate Beesley	
Cambridge	Addenbrooke's Hospital	Safaa Therese	
Cambridge	Addenbrooke's Hospital	James Watson	
Cambridge	Addenbrooke's Hospital	Matthew Stone	
Cambridge	Addenbrooke's Hospital	Isaac Opara	
Cambridge	Addenbrooke's Hospital	Amy Strong n.Chandradass	
Cambridge	Addenbrooke's Hospital	Jane Bushen	
Cambridge	Addenbrooke's Hospital	Sandra Cunningham	
Cambridge	Addenbrooke's Hospital	Svitlana Iyevkova	
Cambridge	Addenbrooke's Hospital	Tatiana Hernandez	Co-I
Cambridge	Addenbrooke's Hospital	Anita Chhabra	Pharmacist
Cambridge	Addenbrooke's Hospital	Abigail Frost	
Cambridge	Addenbrooke's Hospital	Carole Hewitt	
Cambridge	Addenbrooke's Hospital	Ellie Couch	
Cambridge	Addenbrooke's Hospital	Gemma Cullen (née Godsall)	
Cambridge	Addenbrooke's Hospital	Rachel Lister	
Cambridge	Addenbrooke's Hospital	Rebecca Bradley	
Cambridge	Addenbrooke's Hospital	Amanda Walker	
Cambridge	Addenbrooke's Hospital	Debra Mansergh	Pharmacist
Cambridge	Addenbrooke's Hospital	Glynn Rolland	
Cambridge	Addenbrooke's Hospital	Vanessa Moreira	
Cantebruy	Queen Elizabeth The Queen Mother Hospital	Jessica Little	Co-I
Cantebury	Queen Elizabeth The Queen Mother Hospital	Jennifer Turner	Co-I
Cantebury	William Harvey Hospital	Jennifer Turner	Co-I
Cantebury	William Harvey Hospital	Jessica Little	Co-I

Canterbury	Kent and Canterbury Hospital	Albert Edwards	Co-I
Canterbury	Kent and Canterbury Hospital	Charlotte Mott	
Canterbury	Kent and Canterbury Hospital	Fiora Elwes	Co-I
Canterbury	Kent and Canterbury Hospital	Ifigenia Vasiliadou	
Canterbury	Kent and Canterbury Hospital	Ilyas Ahmed	
Canterbury	Kent and Canterbury Hospital	Ioannis Trigonis	Co-I
Canterbury	Kent and Canterbury Hospital	Jennifer Turner	Co-I
Canterbury	Kent and Canterbury Hospital	Jessica Gough	Co-I
Canterbury	Kent and Canterbury Hospital	Lavarniya Rajakumar	
Canterbury	Kent and Canterbury Hospital	Mathini Sridharan	
Canterbury	Kent and Canterbury Hospital	Patryk Brulinski	Co-I
Canterbury	Kent and Canterbury Hospital	Rakesh Raman	Co-I
Canterbury	Kent and Canterbury Hospital	Rohit Malde	
Canterbury	Kent and Canterbury Hospital	Sarah Beasley	
Canterbury	Kent and Canterbury Hospital	Stephane Tankoua	
Canterbury	Kent and Canterbury Hospital	Alice Rendall	Co-I
Canterbury	Kent and Canterbury Hospital	Arafat Mizra	
Canterbury	Kent and Canterbury Hospital	Carys Thomas	PI
Canterbury	Kent and Canterbury Hospital	Christos Mikropoulos	
Canterbury	Kent and Canterbury Hospital	Clary Evans	
Canterbury	Kent and Canterbury Hospital	Gemma Hegarty	
Canterbury	Kent and Canterbury Hospital	Jessica Little	Co-I
Canterbury	Kent and Canterbury Hospital	Joao Galante	Co-I
Canterbury	Kent and Canterbury Hospital	Kannon Nathan	
Canterbury	Kent and Canterbury Hospital	Kathryn Lees	

Canterbury	Kent and Canterbury Hospital	Mathilda Cominos	
Canterbury	Kent and Canterbury Hospital	Matthew Fenton	
Canterbury	Kent and Canterbury Hospital	Mohammed Osman	Co-I
Canterbury	Kent and Canterbury Hospital	Natasha Mithal	Co-I
Canterbury	Kent and Canterbury Hospital	Sharon Beesley	
Canterbury	Kent and Canterbury Hospital	Sugeeta Sukumar	
Canterbury	Kent and Canterbury Hospital	Udaiveer Panwar	
Canterbury	Kent and Canterbury Hospital	Van Sim	Co-I
Canterbury	Kent and Canterbury Hospital	Coral Greenstreet	
Canterbury	Kent and Canterbury Hospital	Hayley Blackgrove	
Canterbury	Kent and Canterbury Hospital	Katy Taylor	
Canterbury	Kent and Canterbury Hospital	Victoria Williamson	
Canterbury	Kent and Canterbury Hospital	Natalie Catt	
Canterbury	Kent and Canterbury Hospital	Arafat Mirza	
Canterbury	Kent and Canterbury Hospital	Sam Gibson	
Canterbury	Kent and Canterbury Hospital	Steve Dann	
Canterbury	Kent and Canterbury Hospital	Andrew Gillian	Pharmacist
Canterbury	Kent and Canterbury Hospital	Miguel Capo-Mir	Pharmacist
Canterbury	Kent and Canterbury Hospital	Cindy Slater	
Canterbury	Kent and Canterbury Hospital	Diane Long	
Canterbury	Kent and Canterbury Hospital	Hasmath Marjolin	
Canterbury	Kent and Canterbury Hospital	Laura Mould	
Canterbury	Kent and Canterbury Hospital	Nikki Crisp	
Canterbury	Kent and Canterbury Hospital	Rachel Larkins	
Canterbury	Kent and Canterbury Hospital	Sandra Holness	

Canterbury	Kent and Canterbury Hospital	Sarah Lines	
Canterbury	Kent and Canterbury Hospital	Susan Rogers	
Canterbury	Kent and Canterbury Hospital	Claire White	
Canterbury	Kent and Canterbury Hospital	Julie Buckley	
Canterbury	Kent and Canterbury Hospital	Laura Kehoe	
Canterbury	Kent and Canterbury Hospital	Lesley Rose	
Canterbury	Kent and Canterbury Hospital	Louise Gladwell	
Canterbury	Kent and Canterbury Hospital	Sarah Lightfoot	Pharmacist
Canterbury	Kent and Canterbury Hospital	Tracy Boakes	
Canterbury	Kent and Canterbury Hospital	Alba Tubau	
Canterbury	Kent and Canterbury Hospital	Bonny Appleby	
Canterbury	Kent and Canterbury Hospital	Caroline Sunderland	
Canterbury	Kent and Canterbury Hospital	Carolyn Hargreaves	
Canterbury	Kent and Canterbury Hospital	Linda Wray	
Canterbury	Kent and Canterbury Hospital	Louise Allen	
Canterbury	Kent and Canterbury Hospital	Marian Wood	
Canterbury	Kent and Canterbury Hospital	Adedolapo Sanni	
Canterbury	Kent and Canterbury Hospital	Claire Pelham	
Canterbury	Kent and Canterbury Hospital	Elizabeth Williamson	
Canterbury	Kent and Canterbury Hospital	Hilary Zurakovsky	
Canterbury	Kent and Canterbury Hospital	Jill Baker	
Canterbury	Kent and Canterbury Hospital	Joanne Williams	
Canterbury	Kent and Canterbury Hospital	Julie-Ann Davies	
Canterbury	Kent and Canterbury Hospital	Karen Robinson	
Canterbury	Kent and Canterbury Hospital	Kathleen (Kathy) Walsh	

Canterbury	Kent and Canterbury Hospital	Kim Mears	
Canterbury	Kent and Canterbury Hospital	Kim Travis	
Canterbury	Kent and Canterbury Hospital	Margaret Lipsham	
Canterbury	Kent and Canterbury Hospital	Paula Whichelo	
Canterbury	Kent and Canterbury Hospital	Sharon Middleton	
Canterbury	Kent and Canterbury Hospital	Sue Kelly	
Canterbury	Kent and Canterbury Hospital	Susan Drakeley	
Canterbury	Kent and Canterbury Hospital	Sydnie Loveland	
Canterbury	Kent and Canterbury Hospital	Molua Young	
Canterbury	Kent and Canterbury Hospital	Denise Crawford	
Cardiff	Velindre Hospital	Aida Hanim Kamarudin	Co-I
Cardiff	Velindre Hospital	Alok Chand	
Cardiff	Velindre Hospital	Andrew Kidd	Co-I
Cardiff	Velindre Hospital	Clair Brunner	
Cardiff	Velindre Hospital	Lisa Victoria Jane Clayton	
Cardiff	Velindre Hospital	Michael Button	Co-I
Cardiff	Velindre Hospital	Nida Hassan	
Cardiff	Velindre Hospital	Satish Kumar	Co-I
Cardiff	Velindre Hospital	Srijith Sashidharan	
Cardiff	Velindre Hospital	Diana Mort	
Cardiff	Velindre Hospital	Jacob Tanguay	PI
Cardiff	Velindre Hospital	Jason Lester	
Cardiff	Velindre Hospital	Jim Barber	Co-I
Cardiff	Velindre Hospital	John Staffurth	Co-I
Cardiff	Velindre Hospital	Louise Harris	

Cardiff	Velindre Hospital	Nachiappan Palaniappan	Co-I
Cardiff	University Hospital of Wales	Elizabeth Bois (nee Harris)	
Cardiff	Velindre Hospital	Emily Rumney	
Cardiff	Velindre Hospital	Emma Cook	
Cardiff	Velindre Hospital	Naomi Woods	
Cardiff	Velindre Hospital	Rebecca Mitchell	
Cardiff	Velindre Hospital	Sandra Greenslade	
Cardiff	Velindre Hospital	Christian Smith	
Cardiff	Velindre Hospital	Gareth Hunt	Pharmacist
Cardiff	Velindre Hospital	James Morgan	
Cardiff	University Hospital of Wales	Krishna Narahari	PI
Cardiff	Velindre Hospital	Phillip Morgan	
Cardiff	Velindre Hospital	Rashmi Jadon	
Cardiff	Velindre Hospital	Robert Henley	
Cardiff	Velindre Hospital	Ross McLeish	
Cardiff	Velindre Hospital	Toby Hiscott	
Cardiff	University Hospital of Wales	Kevin Pearse	
Cardiff	Velindre Hospital	Michael Brown	Pharmacist
Cardiff	University Hospital of Wales	Richard Coulthard	
Cardiff	Velindre Hospital	Amanda Jcekia	
Cardiff	Velindre Hospital	Gillian Willetts	
Cardiff	University Hospital of Wales	Helen Clark	
Cardiff	Velindre Hospital	Helen Clark	
Cardiff	Velindre Hospital	Jayne Richards	
Cardiff	Velindre Hospital	Lynda Holman	

Cardiff	University Hospital of Wales	Samantha Holliday	
Cardiff	Velindre Hospital	Tracy Rees	
Cardiff	Velindre Hospital	Caroline Vitolo	
Cardiff	University Hospital of Wales	Clare Geere (nee Jones)	
Cardiff	Velindre Hospital	Hana Thomas	
Cardiff	Velindre Hospital	Kay Wilson	
Cardiff	University Hospital of Wales	Lynne Harry	
Cardiff	Velindre Hospital	Alison Johnson	
Cardiff	Velindre Hospital	Catherine Sullivan	
Cardiff	Velindre Hospital	Clare Donnithorne	Pharmacist
Cardiff	University Hospital of Wales	Colette Clements	
Cardiff	Velindre Hospital	Colette Kemp	
Cardiff	Velindre Hospital	Debbie O'Connor	
Cardiff	Velindre Hospital	Gladys Makuta	
Cardiff	Velindre Hospital	Jessica Dermott (nee Platt)	
Cardiff	Velindre Hospital	Joanne Preece	
Cardiff	Velindre Hospital	Lisa Stafford	
Cardiff	Velindre Hospital	Loretta Sweeney	
Cardiff	Velindre Hospital	Louise Morgan	
Cardiff	Velindre Hospital	Lucy Chestney	
Cardiff	Velindre Hospital	Necia Jones	
Cardiff	Velindre Hospital	Renata Poole	Pharmacist
Cardiff	Velindre Hospital	Sarah Fry	
Cardiff	Velindre Hospital	Sonya Osborne	
Cardiff	Velindre Hospital	Amanda Jackson	

Cardiff	Velindre Hospital	Bethan Tranter	
Cardiff	Velindre Hospital	Catherine John	
Cardiff	Velindre Hospital	Cathy Richards	Pharmacist
Cardiff	Velindre Hospital	Charlotte Young	
Cardiff	Velindre Hospital	Clare Boobier	
Cardiff	Velindre Hospital	Donna Lear	
Cardiff	Velindre Hospital	Karen Pow	Pharmacist
Cardiff	Velindre Hospital	Kathy Bishop	
Cardiff	Velindre Hospital	Leanne Quinn	
Cardiff	Velindre Hospital	Lucy Wilbraham	
Cardiff	Velindre Hospital	Vicki Reynolds	
Cardiff	University Hospital of Wales	Howard Kynaston	
Cardiff	Velindre Hospital	Malcolm Mason	Co-I
Cardiff	Velindre Hospital	Lynette Lane	
Carlisle	Cumberland Infirmary	Fiona Douglas	
Carlisle	Cumberland Infirmary	Anil Kumar	PI
Carlisle	Cumberland Infirmary	Fergus Young	Pathologist
Carlisle	Cumberland Infirmary	Jonathan Nicoll	
Carlisle	Cumberland Infirmary	Muhammad Rahman	
Carlisle	Cumberland Infirmary	Norma Sidek	
Carlisle	Cumberland Infirmary	Jenna Wildey	
Carlisle	Cumberland Infirmary	Christopher Brewer	Pharmacist
Carlisle	Cumberland Infirmary	Ivor Hughes	Pharmacist
Carlisle	Cumberland Infirmary	Beverley Wilkinson	
Carlisle	Cumberland Infirmary	Patricia Nicholls	

Carlisle	Cumberland Infirmary	Charlotte Eyles	
Carlisle	Cumberland Infirmary	Grace Fryer	
Carlisle	Cumberland Infirmary	Angela Birt	
Carlisle	Cumberland Infirmary	Diane Donnelly	Pharmacist
Chelmsford	Broomfield Hospital	Isabella Maund	
Chelmsford	Broomfield Hospital	Abdel Hamid	PI
Chelmsford	Broomfield Hospital	Gopalakrishnan Srinivasan	Co-I
Chelmsford	Broomfield Hospital	Kiran Kancherla	
Chelmsford	Broomfield Hospital	Priscilla Leone	
Chelmsford	Broomfield Hospital	Udaiveer Panwar	
Chelmsford	Broomfield Hospital	You Yone	
Chelmsford	Broomfield Hospital	Jennifer Child	
Chelmsford	Broomfield Hospital	Emma Mitchell	
Chelmsford	Broomfield Hospital	Amon Wijunamai	
Chelmsford	Broomfield Hospital	Dane Goodere-Bennett	
Chelmsford	Broomfield Hospital	Bryan Singizi	
Chelmsford	Broomfield Hospital	Christian Barnett	
Chelmsford	Broomfield Hospital	Melanie Boxall	
Chelmsford	Broomfield Hospital	Melanie Ruben	
Chelmsford	Broomfield Hospital	Victoria Apps	
Chelmsford	Broomfield Hospital	Donna Briggs	Pharmacist
Chelmsford	Broomfield Hospital	Emma Cannon	Pharmacist
Chelmsford	Broomfield Hospital	Jane Giles	Pharmacist
Chelmsford	Broomfield Hospital	Elizabeth Dawson	
Chelmsford	Broomfield Hospital	Emma Cannon	

Chelmsford	Broomfield Hospital	Lucy Willsher	
Chelmsford	Broomfield Hospital	Nicola Cutmore	
Chelmsford	Broomfield Hospital	Victoria Scott	
Chelmsford	Broomfield Hospital	Edel Spruce	
Chelmsford	Broomfield Hospital	Frances Cairns	Pharmacist
Chelmsford	Broomfield Hospital	Lucy Cooper	Pharmacist
Chelmsford	Broomfield Hospital	Sarah Ferguson	
Chelmsford	Broomfield Hospital	Sian Gibson	
Chelmsford	Broomfield Hospital	Yvonne Lester	
Chelmsford	Broomfield Hospital	Tracey Camburn	
Chelsmford	Broomfield Hospital	Enca Parsons	
Cheltenham	Cheltenham General Hospital	Caitlin Bowden	
Cheltenham	Cheltenham General Hospital	Laura Malins	
Cheltenham	Cheltenham General Hospital	Sai Jonnada	
Cheltenham	Cheltenham General Hospital	Victoria Bell	
Cheltenham	Cheltenham General Hospital	Vishal Bhalla	
Cheltenham	Cheltenham General Hospital	Alex Williams	
Cheltenham	Cheltenham General Hospital	Audrey Cook	
Cheltenham	Cheltenham General Hospital	Duncan Stow	
Cheltenham	Cheltenham General Hospital	Jo Bowen	PI
Cheltenham	Cheltenham General Hospital	Jyothsna Chennupati	
Cheltenham	Cheltenham General Hospital	Peter Jenkins	Co-I
Cheltenham	Cheltenham General Hospital	Roger Owen	
Cheltenham	Cheltenham General Hospital	Amy Skelton	
Cheltenham	Cheltenham General Hospital	Bethan Cartwright	

Cheltenham	Cheltenham General Hospital	Louise Kidner	
Cheltenham	Cheltenham General Hospital	Lucy Blake	
Cheltenham	Cheltenham General Hospital	Madelaine Smith	
Cheltenham	Cheltenham General Hospital	Sarah Stanley	
Cheltenham	Cheltenham General Hospital	Rachel Carter	
Cheltenham	Cheltenham General Hospital	Sarah Beazer	
Cheltenham	Cheltenham General Hospital	Matthew Tan	
Cheltenham	Cheltenham General Hospital	Samuel Croly	
Cheltenham	Cheltenham General Hospital	Ian Ingledew	
Cheltenham	Cheltenham General Hospital	Richard Wallis	Pharmacist
Cheltenham	Cheltenham General Hospital	Helen Babbage	
Cheltenham	Cheltenham General Hospital	Jennifer Dewett	
Cheltenham	Cheltenham General Hospital	Jennifer Smith	
Cheltenham	Cheltenham General Hospital	Jill Chittock	
Cheltenham	Cheltenham General Hospital	Julia Hall	
Cheltenham	Cheltenham General Hospital	Julie Allen	
Cheltenham	Cheltenham General Hospital	Lin Crossley	
Cheltenham	Cheltenham General Hospital	Nicola Robinson	
Cheltenham	Cheltenham General Hospital	Rachel Durrant	Pharmacist
Cheltenham	Cheltenham General Hospital	Rachel Sayers	
Cheltenham	Cheltenham General Hospital	Chris Ford	
Cheltenham	Cheltenham General Hospital	Kate Trigg-Hogarth	
Cheltenham	Cheltenham General Hospital	Sue Wronski	
Cheltenham	Cheltenham General Hospital	Susan Anderson	
Cheltenham	Cheltenham General Hospital	Abi Stuart	

Cheltenham	Cheltenham General Hospital	Charlotte Ayrton	
Cheltenham	Cheltenham General Hospital	Elaine Sizer	
Cheltenham	Cheltenham General Hospital	Elisabeth Read	Pharmacist
Cheltenham	Cheltenham General Hospital	Rebecca Mesher	
Cheltenham	Cheltenham General Hospital	Catherine Stuart-Grumbar	
Cheltenham	Cheltenham General Hospital	Janet Forkes	
Cheltenham	Cheltenham General Hospital	Jennifer Healey-Mariano	
Cheltenham	Cheltenham General Hospital	Rehana Bakawala	
Chester	Countess of Chester Hospital	Azman Ibrahim	PI
Chester	Countess of Chester Hospital	Elizabeth Gallimore	
Chester	Countess of Chester Hospital	Emma Barry	
Chester	Countess of Chester Hospital	Grace McGrath	
Chester	Countess of Chester Hospital	Jenny Miller	
Chester	Countess of Chester Hospital	Lucy Beresford	
Chester	Countess of Chester Hospital	Shannon Spicer	
Chester	Countess of Chester Hospital	Joshua Williams	
Chester	Countess of Chester Hospital	Judith Prince	
Chester	Countess of Chester Hospital	Wesley Artist	Pharmacist
Chester	Countess of Chester Hospital	Chelcie Faulkner	
Chester	Countess of Chester Hospital	Helen Jeffrey	
Chester	Countess of Chester Hospital	Jenny Grounds	
Chester	Countess of Chester Hospital	Kathryn Cawley	
Chester	Countess of Chester Hospital	Rebecca Grogan	
Chester	Countess of Chester Hospital	Rebecca Hopcroft	
Chester	Countess of Chester Hospital	Sarah Illingworth	

Chester	Countess of Chester Hospital	Sue Green	
Chester	Countess of Chester Hospital	Helen Eccleson	
Chester	Countess of Chester Hospital	Denise Archer	
Chester	Countess of Chester Hospital	Janet Spriggs	
Chester	Countess of Chester Hospital	Lisa Dobson (nee Child)	Pharmacist
Chester	Countess of Chester Hospital	Mary Aldous	
Chur	Kantonsspital Graubünden	Dirk Kienle	
Chur	Kantonsspital Graubünden	M Mark	
Chur	Kantonsspital Graubünden	Michael Schwitter	
Chur	Kantonsspital Graubünden	Raeto Strebel	PI
Chur	Kantonsspital Graubünden	Richard Cathomas	
Chur	Kantonsspital Graubünden	Roger von Moos	
Chur	Kantonsspital Graubünden	Carin Aebli	
Chur	Kantonsspital Graubünden	Eloise Kremer	
Chur	Kantonsspital Graubünden	Gabriela Manetsch	
Chur	Kantonsspital Graubünden	Radmila Moudry	Pharmacist
Colchester	Colchester General Hospital	Anita Szita	Co-I
Colchester	Colchester General Hospital	Muthar Kumar	
Colchester	Essex County Hospital	Muthar Kumar	
Colchester	Colchester General Hospital	Sunil Skaria	
Colchester	Colchester General Hospital	Bruce Sizer	Co-I
Colchester	Essex County Hospital	Bruce Sizer	
Colchester	Colchester General Hospital	Dakshinamoorthy Muthukumar	PI
Colchester	Colchester General Hospital	Devy Basu	Co-I
Colchester	Essex County Hospital	Devy Basu	

Colchester	Colchester General Hospital	Rana Mahmood	Co-I
Colchester	Colchester General Hospital	Nicola Taylor	
Colchester	Essex County Hospital	Pugazhenthi Pattu	
Colchester	Colchester General Hospital	Richard Gant	Pharmacist
Colchester	Colchester General Hospital	Louies Mabelin	
Colchester	Colchester General Hospital	Liz Hunting	
Colchester	Essex County Hospital	Liz Hunting	
Colchester	Colchester General Hospital	Lucy Thorogood	
Colchester	Essex County Hospital	Lucy Thorogood	
Colchester	Colchester General Hospital	Katrina Cooke	
Colchester	Colchester General Hospital	Michelle Fisher	
Colchester	Colchester General Hospital	Nicola Cutmore	
Colchester	Colchester General Hospital	Celine Driscoll	
Colchester	Essex County Hospital	Celine Driscoll	
Colchester	Colchester General Hospital	Daisuke Takeuchi	
Colchester	Colchester General Hospital	Hayley Hewer	
Colchester	Essex County Hospital	Hayley Hewer	
Colchester	Colchester General Hospital	Jane Ketley-O'Donel	Pharmacist
Colchester	Essex County Hospital	Jane Ketley-O'Donel	
Colchester	Essex County Hospital	Lorna Dewar	
Colchester	Colchester General Hospital	Michelle Marshall	
Colchester	Essex County Hospital	Michelle Marshall	
Cookridge	Cookridge Hospital	Carmel Loughrey	
Cosham	Queen Alexandra Hospital	Robert Keating	
Cottingham	Castle Hill Hospital	Dulani Ranatunge	

Cottingham	Castle Hill Hospital	George Bozat	
Cottingham	Castle Hill Hospital	Iqtedar Muazzam	
Cottingham	Castle Hill Hospital	Jenny Marsden	
Cottingham	Castle Hill Hospital	Khawaje Zahid	
Cottingham	Castle Hill Hospital	Louise Karsera	
Cottingham	Castle Hill Hospital	Mohan Hingorani	Co-I
Cottingham	Castle Hill Hospital	Faheem Bashir	Co-I
Cottingham	Castle Hill Hospital	Mateen Akhtar	
Cottingham	Castle Hill Hospital	Mohammad Butt	Co-I
Cottingham	Castle Hill Hospital	Bob Bush	Pharmacist
Cottingham	Castle Hill Hospital	A Yousuff	
Cottingham	Castle Hill Hospital	Adam Wolstencroft	Pharmacist
Cottingham	Castle Hill Hospital	Ian Beckley	
Cottingham	Castle Hill Hospital	John Hetherington	
Cottingham	Castle Hill Hospital	Jonathan Gill	
Cottingham	Castle Hill Hospital	Kristian Plowman	
Cottingham	Castle Hill Hospital	Matthew Simms	PI
Cottingham	Castle Hill Hospital	Karen Stubbs	
Cottingham	Castle Hill Hospital	Linzi Bone	
Cottingham	Castle Hill Hospital	Paula O'Reilly	
Cottingham	Castle Hill Hospital	Julie Rawlings	
Cottingham	Castle Hill Hospital	Lucy Richardson	
Cottingham	Castle Hill Hospital	Mary Garthwaite	
Cottingham	Castle Hill Hospital	Sarah Moffat	
Cottingham	Castle Hill Hospital	Suzy Bunton	

Cottingham	Castle Hill Hospital	Carol Hodson	
Cottingham	Castle Hill Hospital	Linda Hoggarth	
Cottingham	Castle Hill Hospital	Sarah Brown	
Cottingham	Castle Hill Hospital	Sarah Palmer	
Cottingham	Castle Hill Hospital	Vicki Lowthorpe	
Coventry	University Hospital Coventry and Warwickshire	Michael Tilby	PI
Coventry	University Hospital Coventry and Warwickshire	Shah Rafique	Co-I
Coventry	University Hospital Coventry and Warwickshire	Andrew Chan	
Coventry	University Hospital Coventry and Warwickshire	Andrew Stockdale	
Coventry	University Hospital Coventry and Warwickshire	Jane Worlding	
Coventry	University Hospital Coventry and Warwickshire	Joanna Hamilton	Co-I
Coventry	University Hospital Coventry and Warwickshire	Senthil Kumar Athmanathan	
Coventry	University Hospital Coventry and Warwickshire	Yakhub Khan	PI
Coventry	University Hospital Coventry and Warwickshire	Fiona McGurk	Pharmacist
Coventry	University Hospital Coventry and Warwickshire	Lucy Miller	
Coventry	University Hospital Coventry and Warwickshire	Mariam Bharuchi	
Coventry	University Hospital Coventry and Warwickshire	Rajbinder Deol	
Coventry	University Hospital Coventry and Warwickshire	Vicky Sturgess	
Coventry	University Hospital Coventry and Warwickshire	Donald Macdonald	
Coventry	University Hospital Coventry and Warwickshire	Mohamed Mooradun	
Coventry	University Hospital Coventry and Warwickshire	Mohammed Khan	Pharmacist
Coventry	University Hospital Coventry and Warwickshire	Albert Mislang	
Coventry	University Hospital Coventry and Warwickshire	Jason Allen	
Coventry	University Hospital Coventry and Warwickshire	Karandeepu Pachoo	Pharmacist
Coventry	University Hospital Coventry and Warwickshire	Kieran Jefferson	

Coventry	University Hospital Coventry and Warwickshire	Mark Whitmore	
Coventry	University Hospital Coventry and Warwickshire	Sukhbinder Salh	Pharmacist
Coventry	Coventry and Warwickshire Hospital	Kathleen Rose	
Coventry	University Hospital Coventry and Warwickshire	Kathleen Rose	
Coventry	University Hospital Coventry and Warwickshire	Laura Stanley	
Coventry	Coventry and Warwickshire Hospital	Leila Fortunato	
Coventry	University Hospital Coventry and Warwickshire	Noor Ayesha Shah	
Coventry	University Hospital Coventry and Warwickshire	Zoe O'Neill	
Coventry	University Hospital Coventry and Warwickshire	Elaine Simmons	
Coventry	University Hospital Coventry and Warwickshire	Rachel Bazeley	
Coventry	University Hospital Coventry and Warwickshire	Sonia Powell	
Coventry	University Hospital Coventry and Warwickshire	Theresa Griffiths	
Coventry	University Hospital Coventry and Warwickshire	Vikki Browne	
Coventry	University Hospital Coventry and Warwickshire	Charlie-marie Suddens	
Coventry	University Hospital Coventry and Warwickshire	Dannielle Burgess	
Coventry	University Hospital Coventry and Warwickshire	Fiona Tranter	
Coventry	University Hospital Coventry and Warwickshire	Jenny Warmington	
Coventry	University Hospital Coventry and Warwickshire	Luanne Carey	
Coventry	University Hospital Coventry and Warwickshire	Padama Singh	Pharmacist
Coventry	University Hospital Coventry and Warwickshire	Sarah O'Toole	
Coventry	University Hospital Coventry and Warwickshire	Stacey Clarke	Pharmacist
Coventry	University Hospital Coventry and Warwickshire	Su Ngwenya	
Coventry	University Hospital Coventry and Warwickshire	Sue Robinson	
Coventry	University Hospital Coventry and Warwickshire	Tammi-Lea Beeby	
Coventry	University Hospital Coventry and Warwickshire	Linda Wimbush	

Coventry	University Hospital Coventry and Warwickshire	Maggie Brown	
Coventry	University Hospital Coventry and Warwickshire	Rachel Thompson	Pharmacist
Coventry	University Hospital Coventry and Warwickshire	Rebecca Aaron	Pharmacist
Crewe	Leighton Hospital	William Croxford	
Crewe	Leighton Hospital	Anna Tran	PI
Crewe	Leighton Hospital	Catherine Thompson	
Crewe	Leighton Hospital	David Butterworth	
Crewe	Leighton Hospital	James Wylie	
Crewe	Leighton Hospital	Michael Braun	
Crewe	Leighton Hospital	Adele Hough	
Crewe	Leighton Hospital	Annabel Tomlinson	
Crewe	Leighton Hospital	Gemma Nash	
Crewe	Leighton Hospital	Karen Hillyer	
Crewe	Leighton Hospital	Katherine Hampton	
Crewe	Leighton Hospital	Sarah Tinsley	Pharmacist
Crewe	Leighton Hospital	Andrew Ritchings	Pharmacist
Crewe	Leighton Hospital	Osman Chohan	
Crewe	Leighton Hospital	P Irwin	
Crewe	Leighton Hospital	P Javle	
Crewe	Leighton Hospital	Carole Bennion	
Crewe	Leighton Hospital	Caroline Walker	
Crewe	Leighton Hospital	Joanne Hughes	
Crewe	Leighton Hospital	Julie Meir	
Crewe	Leighton Hospital	Karen Wilson	
Crewe	Leighton Hospital	Kim Best	

Crewe	Leighton Hospital	Leanne Everall	
Crewe	Leighton Hospital	Lydia Buxton	
Crewe	Leighton Hospital	Nicola Ritchings	
Crewe	Leighton Hospital	Chris Hough	
Crewe	Leighton Hospital	Bethan Roberts	
Crewe	Leighton Hospital	Emma Margerum	
Crewe	Leighton Hospital	Jane Sellman	
Crewe	Leighton Hospital	Karen Gilbert	
Crewe	Leighton Hospital	Rachel Smith	
Crewe	Leighton Hospital	Taya Jones	Pharmacist
Crewe	Leighton Hospital	Thiraviyam Elumalai	
Crewe	Leighton Hospital	Tracy Larcombe	
Crewe	Leighton Hospital	Carolyn Mansfield	
Crewe	Leighton Hospital	Julia Gemmell	
Crewe	Leighton Hospital	Sarah Hoswell	
Crewe	Leighton Hospital	Vanessa Adamson	
Croydon	Croydon University Hospital	Yvonne Campbell	
Croydon	Croydon University Hospital	Ann Payne	
Croydon	Croydon University Hospital	Anne Haldeos	
Croydon	Croydon University Hospital	Cheryl Batish	
Darlington	Darlington Memorial Hospital	Mohammed Kagzi	PI
Darlington	Darlington Memorial Hospital	Clive Peedell	
Darlington	Darlington Memorial Hospital	John Hardman	
Darlington	Darlington Memorial Hospital	Julia McBride	
Darlington	Darlington Memorial Hospital	Steven Pratt	

Darlington	Darlington Memorial Hospital	Tanmay Mukhopadhyay	
Darlington	Darlington Memorial Hospital	Rachel Chatt	
Darlington	Darlington Memorial Hospital	Jonathan Stoddard	
Darlington	Darlington Memorial Hospital	Richard Nendick	Pharmacist
Darlington	Darlington Memorial Hospital	Calum Polwart	Pharmacist
Darlington	Darlington Memorial Hospital	Hyder Latif	
Darlington	Darlington Memorial Hospital	John Vickers	
Darlington	Darlington Memorial Hospital	Asia Sarwar	
Darlington	Darlington Memorial Hospital	Helen Haley	
Darlington	Darlington Memorial Hospital	Kimberly Stamp	
Darlington	Darlington Memorial Hospital	Lynsey Stephenson	Pharmacist
Darlington	Darlington Memorial Hospital	Alison Chilvers	
Darlington	Darlington Memorial Hospital	Claire Henderson	
Darlington	Darlington Memorial Hospital	Susan Wadd	
Darlington	Darlington Memorial Hospital	Lorna Morgan	
Darlington	Darlington Memorial Hospital	Fiona Strong	
Darlington	Darlington Memorial Hospital	Jane Shaw	
Darlington	Darlington Memorial Hospital	Penny Gamble	
Dartford	Darent Valley Hospital	Louise Lacey	
Derb	Queen's Hospital Burton	Christopher Kent	
Derby	Royal Derby Hospital	Ajith Gopinathan Nair	
Derby	Royal Derby Hospital	Alastair McCabe	
Derby	Royal Derby Hospital	Ayman Ramadan	
Derby	Royal Derby Hospital	Chin-Hiong Chong	
Derby	Royal Derby Hospital	Jun Hao Lim	

Derby	Royal Derby Hospital	Kiran Das	
Derby	Royal Derby Hospital	Lauren Jones	
Derby	Royal Derby Hospital	Maeve Pomeroy	
Derby	Royal Derby Hospital	Peter Mason	
Derby	Royal Derby Hospital	Sadia Abdullah	
Derby	Royal Derby Hospital	Seheli Bandyopahdyay	
Derby	Royal Derby Hospital	Thangarajah Mugunthan	
Derby	Royal Derby Hospital	Timothy Podd	
Derby	Royal Derby Hospital	Virgil Sivoglo	
Derby	Royal Derby Hospital	Christopher Kent	
Derby	London Road Community Hospital	Dakshinamoorthy Muthukumar	
Derby	Royal Derby Hospital	Dakshinamoorthy Muthukumar	
Derby	Royal Derby Hospital	Jessica Davies	
Derby	Royal Derby Hospital	Lokesh Puttarachaiah	
Derby	Royal Derby Hospital	Louise Brookes	
Derby	Royal Derby Hospital	Mike Smith-Howell	
Derby	London Road Community Hospital	Prabir Chakraborti	
Derby	Royal Derby Hospital	Prabir Chakraborti	
Derby	Royal Derby Hospital	Prantik Das	PI
Derby	Royal Derby Hospital	Rania Mohammed	
Derby	Royal Derby Hospital	Sarah Taylor	
Derby	Royal Derby Hospital	Sathan Boonyaprapa	
Derby	Royal Derby Hospital	Shahzad Ahmed	
Derby	Royal Derby Hospital	Caroline Coulson	
Derby	Royal Derby Hospital	Georgia Wright	

Derby	Royal Derby Hospital	Helen Beveridge	
Derby	Royal Derby Hospital	Marie Ann Goldsworthy	Pharmacist
Derby	London Road Community Hospital	Heini Jussila	
Derby	London Road Community Hospital	Keeley Smith	
Derby	Royal Derby Hospital	Chris Worth	
Derby	Royal Derby Hospital	James Aldous	
Derby	Royal Derby Hospital	Pugazhenthi Pattu	
Derby	Royal Derby Hospital	Aaron Gallagher	
Derby	London Road Community Hospital	Colin Ward	
Derby	Royal Derby Hospital	Colin Ward	Pharmacist
Derby	Royal Derby Hospital	Fanuel Magaya	
Derby	Royal Derby Hospital	Alison Carrick	
Derby	Royal Derby Hospital	Charlotte Downes	
Derby	Royal Derby Hospital	Donna Beal	
Derby	Royal Derby Hospital	Elizabeth Nadin	
Derby	Royal Derby Hospital	Joely Morgan	
Derby	Royal Derby Hospital	Kashmira Subramanian	
Derby	Royal Derby Hospital	Margaret Harper	
Derby	London Road Community Hospital	Wendy Morrisroe	
Derby	Royal Derby Hospital	Wendy Morrisroe	
Derby	Royal Derby Hospital	Elizabeth Bedford	
Derby	Royal Derby Hospital	Ellie Piggott	
Derby	Royal Derby Hospital	Josephine Chmiel	
Derby	Royal Derby Hospital	Liz Bedford	
Derby	Royal Derby Hospital	Lucy McCandless	

Derby	Royal Derby Hospital	Manni Sandhu	
Derby	Royal Derby Hospital	Nicole McKee	
Derby	Royal Derby Hospital	Nicole McKee (nee Isitt)	
Derby	Royal Derby Hospital	Wendy Abbott	Pharmacist
Derby	Royal Derby Hospital	Claire Wintle	
Derby	Royal Derby Hospital	Emily Mignott	
Derby	Royal Derby Hospital	Emma Brooks	
Derby	Royal Derby Hospital	Hege Strand	
Derby	Royal Derby Hospital	Janet Tomlinson	
Derby	Royal Derby Hospital	Jennifer Mitchell	
Derby	Royal Derby Hospital	Jodie Fitzgerald	
Derby	Royal Derby Hospital	Julie Edmonds	
Derby	London Road Community Hospital	Sarah Hathaway-Lees	
Derby	Royal Derby Hospital	Sarah Longhurst	
Derby	London Road Community Hospital	Debbie Davis	
Derby	Royal Derby Hospital	Gemma Irvine	
Derby	Royal Derby Hospital	Gemma Redfern	
Derby	London Road Community Hospital	Jane Lawrie	
Derby	Royal Derby Hospital	Julie Dockree	
Derby	London Road Community Hospital	Karen Simmonds	
Derby	Royal Derby Hospital	Karen Simmonds	
Derby	London Road Community Hospital	Kay Bowdler	
Derby	London Road Community Hospital	Kristina Duggleby	
Derby	Royal Derby Hospital	Lorraine McDonald	
Derby	Royal Derby Hospital	Mishelle Fanuncio	

Derby	London Road Community Hospital	Sarah Hare	
Derby	Royal Derby Hospital	Sarah Hare	
Derby	Royal Derby Hospital	Shobha Saravanasuthan	
Derby	Royal Derby Hospital	Sonya Bradshaw	
Derby	Royal Derby Hospital	Susan Smith	
Devon	Royal Devon and Exeter Hospital	Anna Lydon	
Doncaster	Doncaster Royal Infirmary	Jessica Tay	Co-I
Doncaster	Doncaster Royal Infirmary	Muneeb Qureshi	
Doncaster	Doncaster Royal Infirmary	Pooja Iyer	
Doncaster	Doncaster Royal Infirmary	Virgil Sivoglo	PI
Doncaster	Doncaster Royal Infirmary	Carmel Pezaro	PI
Doncaster	Doncaster Royal Infirmary	Catherine Ferguson	
Doncaster	Doncaster Royal Infirmary	Georgia Hooton	
Doncaster	Doncaster Royal Infirmary	Janet Field	
Doncaster	Doncaster Royal Infirmary	Jennifer Taylor	
Doncaster	Doncaster Royal Infirmary	Alexandra Firth	
Doncaster	Doncaster Royal Infirmary	Robert Chadwick	
Doncaster	Doncaster Royal Infirmary	Ben East	
Doncaster	Doncaster Royal Infirmary	Amy Neal	
Doncaster	Doncaster Royal Infirmary	Deborah Walstow	
Doncaster	Doncaster Royal Infirmary	Lisa Warren	
Doncaster	Doncaster Royal Infirmary	Meredyth Harris	
Doncaster	Doncaster Royal Infirmary	Nicola Wilkinson	
Doncaster	Doncaster Royal Infirmary	Nicole Jeffcutt	
Doncaster	Doncaster Royal Infirmary	Sharon Ann Allen	

Doncaster	Doncaster Royal Infirmary	Janine Smedley (nee McCabe)	
Doncaster	Doncaster Royal Infirmary	Joanne McNally	Pharmacist
Doncaster	Doncaster Royal Infirmary	Rachel Codling	
Doncaster	Doncaster Royal Infirmary	Barbara Burlace	
Doncaster	Doncaster Royal Infirmary	Joanne Derx	
Doncaster	Doncaster Royal Infirmary	Laura Ellis	
Doncaster	Doncaster Royal Infirmary	Lucy Smith	Co-I
Doncaster	Doncaster Royal Infirmary	Sarah Brown	
Doncaster	Doncaster Royal Infirmary	Kim Wood	
Dorchester	Dorset County Hospital	Benjamin Masters	PI
Dorchester	Dorset County Hospital	Perric Crellin	
Dorchester	Dorset County Hospital	Kate Taylor	
Dorchester	Dorset County Hospital	Ananda Chakrabarti	
Dorchester	Dorset County Hospital	Andrew Rees	
Dorchester	Dorset County Hospital	Naveed Afzal	
Dorchester	Dorset County Hospital	Robert Blegay	
Dorchester	Dorset County Hospital	Stephen Andrews	
Dorchester	Dorset County Hospital	Andrew Cornaby	
Dorchester	Dorset County Hospital	Andrew Gibbins	
Dorchester	Dorset County Hospital	Piet Bakker	
Dorchester	Dorset County Hospital	Simon Sharpe	
Dorchester	Dorset County Hospital	Louise O'Shea	
Dorchester	Dorset County Hospital	Sally Love	
Dorchester	Dorset County Hospital	Sarah Williams	
Dorchester	Dorset County Hospital	Susan Carr	

Dorchester	Dorset County Hospital	Jackie Gibbins	
Dorchester	Dorset County Hospital	Josie Goodsell	
Dorchester	Dorset County Hospital	Beverley Anderson	
Dorchester	Dorset County Hospital	Delia Whiteman	
Dorchester	Dorset County Hospital	Laura Bough	
Dorchester	Dorset County Hospital	Stephanie Jones	
Dorchester	Dorset County Hospital	Tracy Glen	
Dorchester	Dorset County Hospital	Lynn Billett	
Dorchester	Dorset County Hospital	Pauline Ashcroft	Pharmacist
Dorchester	Dorset County Hospital	Sally Breakspear	
Dorchester	Dorset County Hospital	Sarah Horton	
Dorchester	Dorset County Hospital	Suzy Wignall	
Duckworth Lane, Bradford	Bradford Royal Infirmary	Helen Robertshaw	
Dudley	Russells Hall Hospital	Georgi Georgiev	
Dudley	Russells Hall Hospital	Joseph Mano	Co-I
Dudley	Russells Hall Hospital	Pek Keng-Koh	PI
Dudley	Russells Hall Hospital	Syed Tirmazy	
Dudley	Russells Hall Hospital	Abel Zachariah	
Dudley	Russells Hall Hospital	Mano Joseph	Co-I
Dudley	Russells Hall Hospital	Prakash Ramachandra	
Dudley	Russells Hall Hospital	Emily McDonald	
Dudley	Russells Hall Hospital	Joann Atkinson	
Dudley	Russells Hall Hospital	Julie Matthews	
Dudley	Russells Hall Hospital	Andrew Moores	
Dudley	Russells Hall Hospital	David Edwards	

Dudley	Russells Hall Hospital	Lawrence Emtage	
Dudley	Russells Hall Hospital	Manesh Patel	
Dudley	Russells Hall Hospital	Paul Anderson	
Dudley	Russells Hall Hospital	Irene Gardner	
Dudley	Russells Hall Hospital	Vanessa Moore	Pharmacist
Dudley	Russells Hall Hospital	Ruckie Kahlon	
Dudley	Russells Hall Hospital	Anna Summerfield	
Dudley	Russells Hall Hospital	Ellen Shirley	
Dudley	Russells Hall Hospital	Heather McClure	
Dudley	Russells Hall Hospital	Karen Pearson	
Dudley	Russells Hall Hospital	Nadira Jilani	
Dudley	Russells Hall Hospital	Angela Watts	
Dudley	Russells Hall Hospital	Dee Harris	
Dudley	Russells Hall Hospital	Ellie Traverse	
Dudley	Russells Hall Hospital	Hayley Pearson	
Dudley	Russells Hall Hospital	Jayne Kanwar	
Dudley	Russells Hall Hospital	Jenny O'Grady	
Dudley	Russells Hall Hospital	Karen Kanyi	
Dudley	Russells Hall Hospital	Karen McGarry	
Dudley	Russells Hall Hospital	Lesley Edwards	
Dudley	Russells Hall Hospital	Lucie Smith (nee Williams)	Pharmacist
Dudley	Russells Hall Hospital	Sally Keates-Porter	
Dudley	Russells Hall Hospital	Sara Smith	
Dudley	Russells Hall Hospital	Kath Harrow	
Dundee	Ninewells Hospital	Sangeetha Ponnusamy	

Durham	University Hospital of North Durham	Sarah Welsh	
Durham	University Hospital of North Durham	Rhona McMenemin	
Durham	University Hospital of North Durham	Andrew Parker	
Durham	University Hospital of North Durham	Lorna Morgan	
Durham	University Hospital of North Durham	Dorothy Turnbull	
Durham	University Hospital of North Durham	Jean Dent	
Durham	University Hospital of North Durham	Jeanette Maughan	
Durham	University Hospital of North Durham	Julie Elliot	Pharmacist
Durham	University Hospital of North Durham	Julie Elliott	Pharmacist
East Bournemouth	Royal Bournemouth Hospital	Nicky Naraine	
East Sussex	Eastbourne District General Hospital	Graham Watson	
Eastbourne	Eastbourne District General Hospital	Aspasia Soultati	
Eastbourne	Conquest Hospital	Caroline Manetta	PI
Eastbourne	Eastbourne District General Hospital	Caroline Manetta	PI
Eastbourne	Conquest Hospital	Duncan Gilbert	
Eastbourne	Eastbourne District General Hospital	Duncan Gilbert	
Eastbourne	Eastbourne District General Hospital	Fiona McKinna	
Eastbourne	Eastbourne District General Hospital	Peter Rimington	
Eastbourne	Eastbourne District General Hospital	David Sharp	
Eastbourne	Eastbourne District General Hospital	Mark Whitfield	
Eastbourne	Eastbourne District General Hospital	Neville Sharma	Pharmacist
Eastbourne	Eastbourne District General Hospital	William Lawrence	
Eastbourne	Eastbourne District General Hospital	Jo-Anne Taylor	
Eastbourne	Eastbourne District General Hospital	Kay Jones-Skipper	
Eastbourne	Eastbourne District General Hospital	Angie Bowey	

Eastbourne	Eastbourne District General Hospital	Lauren McCrisken	
Eastbourne	Eastbourne District General Hospital	Prudence Hobbs	Pharmacist
Eastbourne	Eastbourne District General Hospital	Shelley Baumber	
Eastbourne	Eastbourne District General Hospital	Theresa Baumber	
Eastbourne	Eastbourne District General Hospital	Joanna Howard	
Edinburgh	Western General Hospital	Alistair Law	Co-I
Edinburgh	Western General Hospital	Amy Cooper	
Edinburgh	Western General Hospital	Dr Aravindhan Sundaramurthy	PI
Edinburgh	Western General Hospital	Archie Macnar	
Edinburgh	Western General Hospital	Caroline Bruce	
Edinburgh	Western General Hospital	Mark Stares	
Edinburgh	Western General Hospital	Martin Doak	
Edinburgh	Western General Hospital	Olvsola Faluyi	
Edinburgh	Western General Hospital	Claire Arthur	
Edinburgh	Western General Hospital	Duncan McLaren	
Edinburgh	Western General Hospital	Ewan Brown	
Edinburgh	Western General Hospital	Grahame Howard	
Edinburgh	Western General Hospital	Hannah Lord	
Edinburgh	Western General Hospital	John McGrane	
Edinburgh	Western General Hospital	Katie Wood	
Edinburgh	Western General Hospital	Sanjana Masinghe	
Edinburgh	Western General Hospital	Emma Lewis	
Edinburgh	Western General Hospital	Heather Howie	
Edinburgh	Western General Hospital	Nikki Gilluley	Pharmacist
Edinburgh	Western General Hospital	Ben Elliott	Pharmacist

Edinburgh	Western General Hospital	Jahangeer Malik	Co-I
Edinburgh	Western General Hospital	Richard Allan	
Edinburgh	Western General Hospital	Roland Donat	
Edinburgh	Western General Hospital	Alan McNeill	
Edinburgh	Western General Hospital	Brian Rogers	
Edinburgh	Western General Hospital	David Jeffrey	
Edinburgh	Western General Hospital	David Tulloch	
Edinburgh	Western General Hospital	Prasad Bollina	
Edinburgh	Western General Hospital	Lynn Ho	
Edinburgh	Western General Hospital	Vivienne Wilson	
Edinburgh	Western General Hospital	Barbara Mayne	
Edinburgh	Western General Hospital	Alison Clark	
Edinburgh	Western General Hospital	Alison McKinlay	
Edinburgh	Western General Hospital	Beverley Mitchell	
Edinburgh	Western General Hospital	Catherine Woods	
Edinburgh	Western General Hospital	Jennifer Baxter	
Edinburgh	Western General Hospital	Kirsty Peebles	
Edinburgh	Western General Hospital	Sarah Thompson	
Edinburgh	Western General Hospital	Tracy Brear	
Edinburgh	Western General Hospital	Ailsa Liddle	
Edinburgh	Western General Hospital	Andrea Stanton	
Edinburgh	Western General Hospital	Ann Cochrane	
Edinburgh	Western General Hospital	Fiona Gardiner	
Edinburgh	Western General Hospital	Fionagh Ross	Pharmacist
Edinburgh	Western General Hospital	Hazel Milligan	

Edinburgh	Western General Hospital	Heather Dalrymple	
Edinburgh	Western General Hospital	Heather McVicars	
Edinburgh	Western General Hospital	Lisa Egan	
Edinburgh	Western General Hospital	Lois Pollock	
Edinburgh	Western General Hospital	Susan Forman	
Edinburgh	Western General Hospital	Theresa Savage	
Edmonton	North Middlesex Hospital	Anna Thompson	
Edmonton	North Middlesex Hospital	Lucinda Melcher	
Edmonton	North Middlesex Hospital	Mausam Singhera	
Edmonton	North Middlesex Hospital	Nishi Gupta	PI
Edmonton	North Middlesex Hospital	Stephen Karp	
Edmonton	North Middlesex Hospital	Ursula McGovern	
Edmonton	North Middlesex Hospital	Ayesha Ahmed Surti	
Edmonton	North Middlesex Hospital	Chloe Van Someren	
Edmonton	North Middlesex Hospital	Sagal Kullane	
Edmonton	North Middlesex Hospital	Girish Bhome	Pharmacist
Edmonton	North Middlesex Hospital	Tom Caumont	
Edmonton	North Middlesex Hospital	Tessa Light	
Edmonton	North Middlesex Hospital	Kerri Rees	
Edmonton	North Middlesex Hospital	Beatrice Balachandran	
Edmonton	North Middlesex Hospital	Bernadette Collins	
Edmonton	North Middlesex Hospital	Ferrial Syed	
Edmonton	North Middlesex Hospital	Kathy O'Farrell	
Edmonton	North Middlesex Hospital	Pauline Lee	
Exeter	Royal Devon and Exeter Hospital	Ayman Nassar	

Exeter	Royal Devon and Exeter Hospital	David Jonathan Chambers	
Exeter	Royal Devon and Exeter Hospital	Lyndon Ridges-Jones	Co-I
Exeter	Royal Devon and Exeter Hospital	Mohini Varughese	PI
Exeter	Royal Devon and Exeter Hospital	Natalie Nityey	
Exeter	Royal Devon and Exeter Hospital	Peter Stephens	Co-I
Exeter	Royal Devon and Exeter Hospital	Shiv Uppal	
Exeter	Royal Devon and Exeter Hospital	Victoria Ford	Co-I
Exeter	Royal Devon and Exeter Hospital	Anne Hong	
Exeter	Royal Devon and Exeter Hospital	Denise Sheehan	
Exeter	Royal Devon and Exeter Hospital	Elizabeth Toy	
Exeter	Royal Devon and Exeter Hospital	Rajaguru Srinivasan	Co-I
Exeter	Royal Devon and Exeter Hospital	San Aung	Co-I
Exeter	Royal Devon and Exeter Hospital	Tim Norris	
Exeter	Royal Devon and Exeter Hospital	Ceri Davies	
Exeter	Royal Devon and Exeter Hospital	Eleonor (Alethea) Brown	
Exeter	Royal Devon and Exeter Hospital	Grace Justice	
Exeter	Royal Devon and Exeter Hospital	Kerri-ellen Oakley	
Exeter	Royal Devon and Exeter Hospital	Sophie James	
Exeter	Royal Devon and Exeter Hospital	Stephanie Ann Ellis	
Exeter	Royal Devon and Exeter Hospital	Theresa Lawless	
Exeter	Royal Devon and Exeter Hospital	Susan Downer	
Exeter	Royal Devon and Exeter Hospital	Alan Betts	
Exeter	Royal Devon and Exeter Hospital	James Leavy	
Exeter	Royal Devon and Exeter Hospital	Matt Trivett	
Exeter	Royal Devon and Exeter Hospital	Petar Hitev	

Exeter	Royal Devon and Exeter Hospital	Christoph Lohan	
Exeter	Royal Devon and Exeter Hospital	John Anderson	
Exeter	Royal Devon and Exeter Hospital	Ross Curwen	
Exeter	Royal Devon and Exeter Hospital	Elaine Vandcandelaere	
Exeter	Royal Devon and Exeter Hospital	Emma Guerin	
Exeter	Royal Devon and Exeter Hospital	Frances Hood	
Exeter	Royal Devon and Exeter Hospital	Melissa Davey	
Exeter	Royal Devon and Exeter Hospital	Alison Augstburger	
Exeter	Royal Devon and Exeter Hospital	Claire Webb	
Exeter	Royal Devon and Exeter Hospital	Emma Robjohns	
Exeter	Royal Devon and Exeter Hospital	Ingrid Seath	
Exeter	Royal Devon and Exeter Hospital	Kate O'Connor	
Exeter	Royal Devon and Exeter Hospital	Alison Roantree	
Exeter	Royal Devon and Exeter Hospital	Jane Piper	
Exeter	Royal Devon and Exeter Hospital	Rosie Mew	Co-I
Exeter	Royal Devon and Exeter Hospital	Sophie Warren	
Exeter	Royal Devon and Exeter Hospital	Susan Davenport	Pharmacist
Exeter	Royal Devon and Exeter Hospital	Tracey Foss	
Exeter	Royal Devon and Exeter Hospital	Beverley Kemp	
Exeter	Royal Devon and Exeter Hospital	Claire Ridler	
Exeter	Royal Devon and Exeter Hospital	Dawn Edwards	
Exeter	Royal Devon and Exeter Hospital	Elizabeth Davey	
Exeter	Royal Devon and Exeter Hospital	Fiona Walters (nee Hall)	Pharmacist
Exeter	Royal Devon and Exeter Hospital	Kizzy Baines	
Exeter	Royal Devon and Exeter Hospital	Lyndel Moore	

Exeter	Royal Devon and Exeter Hospital	Shirley Todd	Pharmacist
Exeter	Royal Devon and Exeter Hospital	Suzy Tasker	
Exeter	Royal Devon and Exeter Hospital	Tamika Chapter	Pharmacist
Fulwood	Blackburn Royal Infirmary	Natalie Charnley	
Fulwood	Burnley General Hospital	Natalie Charnley	
Fulwood	Royal Preston Hospital	Hazel Aston	
Gillingham	Medway Maritime Hospital	Afroditi Karathanasi	
Gillingham	Medway Maritime Hospital	Diletta Bianchini	
Gillingham	Medway Maritime Hospital	Stergios Boussios	PI
Gillingham	Medway Maritime Hospital	Swapna Thomas	
Gillingham	Medway Maritime Hospital	Charlotte Abson	Co-I
Gillingham	Medway Maritime Hospital	Christos Mikropoulos	Co-I
Gillingham	Medway Maritime Hospital	Henry Taylor	PI
Gillingham	Medway Maritime Hospital	Tessa Lawrence	
Gillingham	Medway Maritime Hospital	Agne Sadauskaite	
Gillingham	Medway Maritime Hospital	Corinne Borley	
Gillingham	Medway Maritime Hospital	Durga Maya Gurung	
Gillingham	Medway Maritime Hospital	Jodie Seymour	
Gillingham	Medway Maritime Hospital	Mary Everett	
Gillingham	Medway Maritime Hospital	Charles Davis	
Gillingham	Medway Maritime Hospital	James Sawyer	Pharmacist
Gillingham	Medway Maritime Hospital	Kevin Naicker	
Gillingham	Medway Maritime Hospital	Khalid Abdalla	Pharmacist
Gillingham	Medway Maritime Hospital	Peter Milverton	
Gillingham	Medway Maritime Hospital	Philip Adeniran	

Gillingham	Medway Maritime Hospital	Simon Wan	
Gillingham	Medway Maritime Hospital	Parool Darbar	Pharmacist
Gillingham	Medway Maritime Hospital	Richard Thornton	
Gillingham	Medway Maritime Hospital	Alba Tuban	
Gillingham	Medway Maritime Hospital	Alison Richards	
Gillingham	Medway Maritime Hospital	Clarissa Madla	
Gillingham	Medway Maritime Hospital	Deirdre Cooke	
Gillingham	Medway Maritime Hospital	Elizabeth Newman-Horne	
Gillingham	Medway Maritime Hospital	Gayzel Vallejera	
Gillingham	Medway Maritime Hospital	Katarzyna Urbanczyk	
Gillingham	Medway Maritime Hospital	Kay Jones	
Gillingham	Medway Maritime Hospital	Lisa Parker	
Gillingham	Medway Maritime Hospital	Louise Black	
Gillingham	Medway Maritime Hospital	Louise Brassington	
Gillingham	Medway Maritime Hospital	Marie Louise Hollands	
Gillingham	Medway Maritime Hospital	Nicola Southwell	
Gillingham	Medway Maritime Hospital	Emma Sutton	
Gillingham	Medway Maritime Hospital	Judy Filmer	
Gillingham	Medway Maritime Hospital	Suzie Reyner	
Gillingham	Medway Maritime Hospital	Adedolapo Sanni	
Gillingham	Medway Maritime Hospital	Carol Mayger	Pharmacist
Gillingham	Medway Maritime Hospital	Tamara Diamond	
Glasgow	Beatson West of Scotland Cancer Centre	Aisha Tufail	
Glasgow	Beatson West of Scotland Cancer Centre	Almudena Cascales	
Glasgow	Beatson West of Scotland Cancer Centre	Ashleigh Kerr	

Glasgow	Beatson West of Scotland Cancer Centre	Brendan McCann	
Glasgow	Beatson West of Scotland Cancer Centre	Cicely Cunningham	
Glasgow	Beatson West of Scotland Cancer Centre	Derek Grose	Co-I
Glasgow	Beatson West of Scotland Cancer Centre	Ian Sanders	
Glasgow	Beatson West of Scotland Cancer Centre	Jawaher Ansari	
Glasgow	Beatson West of Scotland Cancer Centre	Kathryn Banfill	Co-I
Glasgow	Beatson West of Scotland Cancer Centre	Miranda Ashton	
Glasgow	Beatson West of Scotland Cancer Centre	Rebecca Muirhead	
Glasgow	Beatson West of Scotland Cancer Centre	Stephen McKay	
Glasgow	Beatson West of Scotland Cancer Centre	Abdulla Al-hasso	
Glasgow	Beatson West of Scotland Cancer Centre	Aqilah Othman	
Glasgow	Beatson West of Scotland Cancer Centre	Awris Jalil	
Glasgow	Beatson West of Scotland Cancer Centre	Azmat Sadozye	
Glasgow	Beatson West of Scotland Cancer Centre	Balaji Venugopal	
Glasgow	Beatson West of Scotland Cancer Centre	Ben Fulton	
Glasgow	Beatson West of Scotland Cancer Centre	Carolynn Lamb	Co-I
Glasgow	Beatson West of Scotland Cancer Centre	Christina Wilson	
Glasgow	Beatson West of Scotland Cancer Centre	David Dodds	
Glasgow	Beatson West of Scotland Cancer Centre	Esfandiyar Khan	
Glasgow	Beatson West of Scotland Cancer Centre	Hilary Glen	
Glasgow	Beatson West of Scotland Cancer Centre	Husam Marashi	
Glasgow	Beatson West of Scotland Cancer Centre	Jan Wallace	
Glasgow	Beatson West of Scotland Cancer Centre	Janet Graham	
Glasgow	Beatson West of Scotland Cancer Centre	John Graham	PI
Glasgow	Beatson West of Scotland Cancer Centre	Kathryn Graham	

Glasgow	Beatson West of Scotland Cancer Centre	Lye Mun Tho	
Glasgow	Beatson West of Scotland Cancer Centre	Martin Russell	
Glasgow	Beatson West of Scotland Cancer Centre	Maryon Hardie	
Glasgow	Beatson West of Scotland Cancer Centre	Mohammed Alfayez	
Glasgow	Beatson West of Scotland Cancer Centre	Nicholas Macleod	
Glasgow	Beatson West of Scotland Cancer Centre	Norma Sidek	
Glasgow	Beatson West of Scotland Cancer Centre	Patricia Roxburgh	
Glasgow	Beatson West of Scotland Cancer Centre	Paula Henry-Stephenson	
Glasgow	Beatson West of Scotland Cancer Centre	Rana Mahmood	
Glasgow	Beatson West of Scotland Cancer Centre	Rob Jones	PI
Glasgow	Beatson West of Scotland Cancer Centre	Sally Hall	
Glasgow	Beatson West of Scotland Cancer Centre	Saranya Kakumanu	
Glasgow	Beatson West of Scotland Cancer Centre	Sophie Barrett	
Glasgow	Beatson West of Scotland Cancer Centre	Stefan Nowicki	
Glasgow	Beatson West of Scotland Cancer Centre	Tareq Abdullah	
Glasgow	Beatson West of Scotland Cancer Centre	Diann Taggart	
Glasgow	Beatson West of Scotland Cancer Centre	Katie Galbraith	
Glasgow	Beatson West of Scotland Cancer Centre	Nicola Cairns	
Glasgow	Beatson West of Scotland Cancer Centre	Gerard Forrest	
Glasgow	Beatson West of Scotland Cancer Centre	Calum Innes	
Glasgow	Beatson West of Scotland Cancer Centre	Graeme Lumsden	
Glasgow	Beatson West of Scotland Cancer Centre	Martin Ball	Pharmacist
Glasgow	Beatson West of Scotland Cancer Centre	Nathan Richardson	
Glasgow	Beatson West of Scotland Cancer Centre	Ross Carruthers	
Glasgow	Beatson West of Scotland Cancer Centre	Hannah Weir	

Glasgow	Beatson West of Scotland Cancer Centre	Linzi Rae	
Glasgow	Beatson West of Scotland Cancer Centre	Maureen Connolly	
Glasgow	Beatson West of Scotland Cancer Centre	Ailsa Griffen	
Glasgow	Beatson West of Scotland Cancer Centre	Jacqueline Gourlay	Pharmacist
Glasgow	Beatson West of Scotland Cancer Centre	Jan Graham	
Glasgow	Beatson West of Scotland Cancer Centre	Karen Bell	
Glasgow	Beatson West of Scotland Cancer Centre	Alice Coy	
Glasgow	Beatson West of Scotland Cancer Centre	Gemma Johnson	
Glasgow	Beatson West of Scotland Cancer Centre	Gillian Barmack	
Glasgow	Beatson West of Scotland Cancer Centre	Jennifer Petrie	
Glasgow	Beatson West of Scotland Cancer Centre	Annette Charlick	
Glasgow	Beatson West of Scotland Cancer Centre	Antonia MacMillan	
Glasgow	Beatson West of Scotland Cancer Centre	Catriona Cowan	
Glasgow	Beatson West of Scotland Cancer Centre	Claire Steele	
Glasgow	Beatson West of Scotland Cancer Centre	Fiona McQueen	Pharmacist
Glasgow	Beatson West of Scotland Cancer Centre	Jenny Brown	Pharmacist
Glasgow	Beatson West of Scotland Cancer Centre	Judith Dixon	
Glasgow	Beatson West of Scotland Cancer Centre	Kirsteen Stuart	
Glasgow	Beatson West of Scotland Cancer Centre	Kirsten Laws (nee Borthwick)	
Glasgow	Beatson West of Scotland Cancer Centre	Lorraine Barwell	
Glasgow	Beatson West of Scotland Cancer Centre	Louise Bruce	
Glasgow	Beatson West of Scotland Cancer Centre	Lynne Grieve	
Glasgow	Beatson West of Scotland Cancer Centre	Maria Nicol	
Glasgow	Beatson West of Scotland Cancer Centre	Patricia Baird	
Glasgow	Beatson West of Scotland Cancer Centre	Ruth Orr	

Glasgow	Beatson West of Scotland Cancer Centre	Sai Juan Jia	
Gloucester	Gloucestershire Royal Hospital	Laura Malins	
Gloucester	Gloucestershire Royal Hospital	Sai Jonnada	
Gloucester	Gloucestershire Royal Hospital	Victoria Bell	
Gloucester	Gloucestershire Royal Hospital	Audrey Cook	
Gloucester	Gloucestershire Royal Hospital	Jo Bowen	PI
Gloucester	Gloucestershire Royal Hospital	Peter Jenkins	Co-I
Gloucester	Gloucestershire Royal Hospital	Roger Owen	
Gloucester	Gloucestershire Royal Hospital	Amy Skelton	
Gloucester	Gloucestershire Royal Hospital	Bethan Cartwright	
Gloucester	Gloucestershire Royal Hospital	Sarah Stanley	
Gloucester	Gloucestershire Royal Hospital	Sarah Beazer	
Gloucester	Gloucestershire Royal Hospital	Samuel Croly	
Gloucester	Gloucestershire Royal Hospital	Richard Wallis	
Gloucester	Gloucestershire Royal Hospital	Julia Hall	
Gloucester	Gloucestershire Royal Hospital	Julie Allen	
Gloucester	Gloucestershire Royal Hospital	Lin Crossley	
Gloucester	Gloucestershire Royal Hospital	Rachel Sayers	
Gloucester	Gloucestershire Royal Hospital	Chris Ford	
Gloucester	Gloucestershire Royal Hospital	Kate Trigg-Hogarth	
Gloucester	Gloucestershire Royal Hospital	Sue Wronski	
Gloucester	Gloucestershire Royal Hospital	Abi Stuart	
Gloucester	Gloucestershire Royal Hospital	Charlotte Ayrton	
Gloucester	Gloucestershire Royal Hospital	Claire Salter	
Gloucester	Gloucestershire Royal Hospital	Elaine Sizer	

Gloucester	Gloucestershire Royal Hospital	Elisabeth Read	
Gloucester	Gloucestershire Royal Hospital	Louise Moore	
Gloucester	Gloucestershire Royal Hospital	Sarah Matthews	
Glouster	Gloucestershire Royal Hospital	Janet Forkes	
Guildford	Royal Surrey County Hospital	Carla Perna	PI
Guildford	Royal Surrey County Hospital	Emmanuel Larbi	
Guildford	Royal Surrey County Hospital	James Lowe	
Guildford	Royal Surrey County Hospital	Leslie Cheng	Co-I
Guildford	Royal Surrey County Hospital	Mahwish Karim	Co-I
Guildford	Royal Surrey County Hospital	Melanie Boafo-Yirenkyi	
Guildford	Royal Surrey County Hospital	Sara Khaksar	
Guildford	Royal Surrey County Hospital	Charlotte Shelley	
Guildford	Royal Surrey County Hospital	Jenny Nobes	
Guildford	Royal Surrey County Hospital	Joanna Stokoe	
Guildford	Royal Surrey County Hospital	Julian Money-Kyrle	
Guildford	Royal Surrey County Hospital	Katie Wood	
Guildford	Royal Surrey County Hospital	Katie Wood	
Guildford	Royal Surrey County Hospital	Mahomed Moosa	
Guildford	Royal Surrey County Hospital	Maria Drzymala	
Guildford	Royal Surrey County Hospital	Richard Shaffer	
Guildford	Royal Surrey County Hospital	Robert Laing	
Guildford	Royal Surrey County Hospital	Sree Susaria	
Guildford	Royal Surrey County Hospital	Teresa Guerrero-Urbano	
Guildford	Royal Surrey County Hospital	Imogen Heenan	
Guildford	Royal Surrey County Hospital	Kavita Bhat	

Guildford	Royal Surrey County Hospital	Lesley Harden	
Guildford	Royal Surrey County Hospital	Nick Pilkington	
Guildford	Royal Surrey County Hospital	Richmond Abeseabe	
Guildford	Royal Surrey County Hospital	Angela Morgan	
Guildford	Royal Surrey County Hospital	Miriam White	
Guildford	Royal Surrey County Hospital	Adele Hugg	
Guildford	Royal Surrey County Hospital	Caterina Bissa	
Guildford	Royal Surrey County Hospital	Jane Woods	
Guildford	Royal Surrey County Hospital	Julie Wilkinson	
Guildford	Royal Surrey County Hospital	Veronica Davis	
Guildford	Royal Surrey County Hospital	Frances Sidi	
Guildford	Royal Surrey County Hospital	Jen Julius	
Guildford	Royal Surrey County Hospital	Julia Whittle	
Guildford	Royal Surrey County Hospital	Kate Penhaligon	
Guildford	Royal Surrey County Hospital	Kathrin Narvaez-Vega	Pharmacist
Guildford	Royal Surrey County Hospital	Lucinda Scott	
Guildford	Royal Surrey County Hospital	Marianne Dabbs	
Guildford	Royal Surrey County Hospital	Stephy Joseph	
Guildford	Royal Surrey County Hospital	Catherine Medcalf	Pharmacist
Guildford	Royal Surrey County Hospital	Celia Harris	
Guildford	Royal Surrey County Hospital	Daisy Floyd	
Guildford	Royal Surrey County Hospital	Fiona Butler	Pharmacist
Guildford	Royal Surrey County Hospital	Linda Nardone	
Guildford	Royal Surrey County Hospital	Sarah De Swert	
Guildford	Royal Surrey County Hospital	Sue Sargent	

Guildford	Royal Surrey County Hospital	Teresa Keating	
Guildford	Royal Surrey County Hospital	Zephyrine King	
Halifax	Calderdale Royal Hospital	Miranda Usher	
Halifax	Calderdale Royal Hospital	Lisa Gledhill	
Halton	Halton Hospital	Carrie Lowthian	
Hampstead	Royal Free Hospital	R Bradford	
Harlow	Princess Alexandra Hospital (Harlow)	Albert Edwards	
Harlow	Princess Alexandra Hospital (Harlow)	Hamoun Rozati	Co-I
Harlow	Princess Alexandra Hospital (Harlow)	Paul Kabuubi	
Harlow	Princess Alexandra Hospital (Harlow)	Shroma De Silva	
Harlow	Princess Alexandra Hospital (Harlow)	Tasia Aghadiuno	Co-I
Harlow	Princess Alexandra Hospital (Harlow)	Zainab Wasim	Co-I
Harlow	Princess Alexandra Hospital (Harlow)	Anna Lerner	Co-I
Harlow	Princess Alexandra Hospital (Harlow)	Lucinda Melcher	PI
Harlow	Princess Alexandra Hospital (Harlow)	Nishi Gupta	Co-I
Harlow	Princess Alexandra Hospital (Harlow)	Reena Davda	
Harlow	Princess Alexandra Hospital (Harlow)	Reena Davda	Co-I
Harlow	Princess Alexandra Hospital (Harlow)	Nikki White (nee Staines)	
Harlow	Princess Alexandra Hospital (Harlow)	Sylwia Goliaszewska	
Harlow	Princess Alexandra Hospital (Harlow)	Ahmed Hnoosh	
Harlow	Princess Alexandra Hospital (Harlow)	Ervin Shpuza	
Harlow	Princess Alexandra Hospital (Harlow)	Sunjalee Fernando	Pharmacist
Harlow	Princess Alexandra Hospital (Harlow)	Cait Rees	
Harlow	Princess Alexandra Hospital (Harlow)	Amanda Lewis	
Harlow	Princess Alexandra Hospital (Harlow)	Amelia Daniel	

Harlow	Princess Alexandra Hospital (Harlow)	Amy Lewis	
Harlow	Princess Alexandra Hospital (Harlow)	Gemma Cook	
Harlow	Princess Alexandra Hospital (Harlow)	Hana Malinkovicova	
Harlow	Princess Alexandra Hospital (Harlow)	Joanne Kellaway	
Harlow	Princess Alexandra Hospital (Harlow)	Tracey White	
Harlow	Princess Alexandra Hospital (Harlow)	Evelyn Holmes	Pharmacist
Harlow	Princess Alexandra Hospital (Harlow)	Jodie Johnson	
Harlow	Princess Alexandra Hospital (Harlow)	Teresa Light	
Haverford West	Withybush General Hospital	Sandra Griffiths nee Evens	
Headington	Churchill Hospital	James Wakelin	
Headington	Churchill Hospital	Jane Gibbard	
Headington	Churchill Hospital	Leigh Burns	
Headington	Churchill Hospital	Sandie Wellman	
Hereford	Hereford County Hospital	Caitlin Bowden	
Hereford	Hereford County Hospital	Cara Watson	
Hereford	Hereford County Hospital	David Stow	
Hereford	Hereford County Hospital	Timothy Spencer	
Hereford	Hereford County Hospital	Vishal Bhalla	
Hereford	Hereford County Hospital	Warren Grant	PI
Hereford	Hereford County Hospital	Audrey Cook	
Hereford	Hereford County Hospital	Duncan Stow	
Hereford	Hereford County Hospital	Maxine Flubacher	
Hereford	Hereford County Hospital	Nina Reeve	
Hereford	Hereford County Hospital	Bethan Richards	
Hereford	Hereford County Hospital	Jolanta Pueskacz	

Hereford	Hereford County Hospital	Linda Moseley	Pharmacist
Hereford	Hereford County Hospital	Lisa King	
Hereford	Hereford County Hospital	Nicola Williamson	
Hereford	Hereford County Hospital	Sarah Chapman	
Hereford	Hereford County Hospital	Serrafina Carini	
Hereford	Hereford County Hospital	Sophie Cooper	
Hereford	Hereford County Hospital	Stacey Turner	
Hereford	Hereford County Hospital	Harriet Taylor	
Hereford	Hereford County Hospital	Jagdish Chana	Pharmacist
Hereford	Hereford County Hospital	Terry Watson	
Hereford	Hereford County Hospital	Andy Hedges	Pharmacist
Hereford	Hereford County Hospital	Amanda Davies	
Hereford	Hereford County Hospital	Isabel Martin (Whitehouse)	
Hereford	Hereford County Hospital	June Thomas	
Hereford	Hereford County Hospital	Laura Lees	
Hereford	Hereford County Hospital	Melanie Evans	
Hereford	Hereford County Hospital	Rebecca Bengree	
Hereford	Hereford County Hospital	Susan Anderson	
Hereford	Hereford County Hospital	Zara Roberts	
Hereford	Hereford County Hospital	Bethany Wellington	
Hereford	Hereford County Hospital	Janine Jones (Birch)	
Hereford	Hereford County Hospital	Naeem Musani	
Hereford	Hereford County Hospital	Susan Anderson	
Hereford	Hereford County Hospital	Caroline Thomas	Pharmacist
Hereford	Hereford County Hospital	Catherine Reed	

Hereford	Hereford County Hospital	Claire Hughes	
Hereford	Hereford County Hospital	Gill Horsfield	
Hereford	Hereford County Hospital	Jenny Howls	Pharmacist
Hereford	Hereford County Hospital	Kate Hammerton	
Hereford	Hereford County Hospital	Rachel Lowe	
Hereford	Hereford County Hospital	Sophie Boyd (nee Evans)	
Hereford	Hereford County Hospital	Anita Ashton	
Hereford	Hereford County Hospital	Janet Forkes	
Hereford	Hereford County Hospital	Sophie Boyd	
Herts	Lister Hospital	Rachel Low	
High Wycombe	Wycombe Hospital	Ami Sabharwal	PI
High Wycombe	Wycombe Hospital	Avinash Gupta	
High Wycombe	Wycombe Hospital	Janice Carpenter	
High Wycombe	Wycombe Hospital	Katherine Hyde	PI
High Wycombe	Wycombe Hospital	Thinn Pwint	Co-I
High Wycombe	Wycombe Hospital	Benjamin Fairfax	
High Wycombe	Wycombe Hospital	Gerard Andrade	Co-I
High Wycombe	Wycombe Hospital	Niki Panakis	
High Wycombe	Wycombe Hospital	Philip Camilleri	Co-I
High Wycombe	Wycombe Hospital	Prabir Chakraborti	Co-I
High Wycombe	Wycombe Hospital	Sally Trent	
High Wycombe	Wycombe Hospital	Sean O'Cathail	Co-I
High Wycombe	Wycombe Hospital	Vivek Mohan	
High Wycombe	Wycombe Hospital	Wasiru Saka	
High Wycombe	Stoke Mandeville Hospital	Chrissie Butcher	

High Wycombe	Wycombe Hospital	Chrissie Butcher	
High Wycombe	Wycombe Hospital	Claire Fernandez	
High Wycombe	Wycombe Hospital	Amarjit Bdesha	
High Wycombe	Wycombe Hospital	Moncy Mathew	
High Wycombe	Wycombe Hospital	Neil Haldar	
High Wycombe	Wycombe Hospital	Rahul Kurup	
High Wycombe	Wycombe Hospital	John Patrick Kelleher	
High Wycombe	Wycombe Hospital	Neil Trew-Smith	
High Wycombe	Wycombe Hospital	Alice Ngumo	
High Wycombe	Wycombe Hospital	Anita Cserbane	
High Wycombe	Wycombe Hospital	Gail Varley	
High Wycombe	Wycombe Hospital	Janet Weir	
High Wycombe	Wycombe Hospital	Manisha Joshi	
High Wycombe	Wycombe Hospital	Penny Carter	
High Wycombe	Wycombe Hospital	Siobhan Gettings	
High Wycombe	Wycombe Hospital	Maggie Aldersley	
High Wycombe	Wycombe Hospital	Michelle Taylor-Siddons	Pharmacist
High Wycombe	Wycombe Hospital	Tracey Stammers	
High Wycombe	Wycombe Hospital	Ans-Mari Bester	
High Wycombe	Wycombe Hospital	Aruna Nair	
High Wycombe	Wycombe Hospital	Catherine Northey	
High Wycombe	Wycombe Hospital	Emma Hogbin	
High Wycombe	Wycombe Hospital	Erica Lieberman	Pharmacist
High Wycombe	Wycombe Hospital	Helena Stone	
High Wycombe	Wycombe Hospital	Kathryn Herbert	

High Wycombe	Wycombe Hospital	Rossana Mancinelli	
High Wycombe	Wycombe Hospital	Samantha Thomas	
High Wycombe	Wycombe Hospital	Sarah Manyangadze	
High Wycombe	Wycombe Hospital	Susan McLain-Smith	
High Wycombe	Wycombe Hospital	Tiffany Chan	Pharmacist
High Wycombe	Stoke Mandeville Hospital	Bhavna Badiani	
High Wycombe	Wycombe Hospital	Bhavna Badiani	
High Wycombe	Wycombe Hospital	Christine Collins	
High Wycombe	Wycombe Hospital	Evelyn Chan	
High Wycombe	Wycombe Hospital	Hazel Wynn	
High Wycombe	Wycombe Hospital	Ileana Nguyen	
High Wycombe	Wycombe Hospital	Jasvinder Bains	
High Wycombe	Wycombe Hospital	Nicola Bowers	
High Wycombe	Wycombe Hospital	Roisin Kavanagh	
High Wycombe	Wycombe Hospital	Andrew Protheroe	
Huddersfield	Huddersfield Royal Infirmary	Omer Babiker	Co-I
Huddersfield	Huddersfield Royal Infirmary	Rob Turner	
Huddersfield	Huddersfield Royal Infirmary	Samantha Turnbull	Co-I
Huddersfield	Huddersfield Royal Infirmary	Barbara Crosse	
Huddersfield	Huddersfield Royal Infirmary	Emma Woodward	
Huddersfield	Huddersfield Royal Infirmary	Jane Hook	
Huddersfield	Huddersfield Royal Infirmary	Uschi Hofmann	PI
Huddersfield	Huddersfield Royal Infirmary	Hannah Riley	
Huddersfield	Huddersfield Royal Infirmary	Sanya Anjum	
Huddersfield	Huddersfield Royal Infirmary	Sarah Hanley	

Huddersfield	Huddersfield Royal Infirmary	Adam Mawer	Pharmacist
Huddersfield	Huddersfield Royal Infirmary	Deivasikamani Ramanujam	Co-I
Huddersfield	Huddersfield Royal Infirmary	Mohammad Irfan Alam	
Huddersfield	Huddersfield Royal Infirmary	Nicolas Bryan	Co-I
Huddersfield	Huddersfield Royal Infirmary	Hayley Webster	Pharmacist
Huddersfield	Huddersfield Royal Infirmary	Julie Millward	
Huddersfield	Huddersfield Royal Infirmary	Kathryn Smith	
Huddersfield	Huddersfield Royal Infirmary	Kully Sandhu	
Huddersfield	Huddersfield Royal Infirmary	Lear Matapure	
Huddersfield	Huddersfield Royal Infirmary	Lee-Ann Bayo	
Huddersfield	Huddersfield Royal Infirmary	Melanie Quesne	
Huddersfield	Huddersfield Royal Infirmary	Nicky Daker	
Huddersfield	Huddersfield Royal Infirmary	Hayley Inman	
Huddersfield	Huddersfield Royal Infirmary	Lisa Shaw	
Huddersfield	Huddersfield Royal Infirmary	Miranda Usher	
Huddersfield	Huddersfield Royal Infirmary	Belinda McLean	
Huddersfield	Huddersfield Royal Infirmary	Diane Kelly	
Huddersfield	Huddersfield Royal Infirmary	Karen Bicknell	
Huddersfield	Huddersfield Royal Infirmary	Katherine Tighe	
Huddersfield	Huddersfield Royal Infirmary	Lindsay Greenhalgh	Pharmacist
Huddersfield	Huddersfield Royal Infirmary	Lucy Jones	Co-I
Huddersfield	Huddersfield Royal Infirmary	Monica Narasimham	
Huddersfield	Huddersfield Royal Infirmary	Naledi Mzwimbi	
Huddersfield	Huddersfield Royal Infirmary	Paula Gomes	Pharmacist
Huddersfield	Huddersfield Royal Infirmary	Christine Turner	

Huddersfield	Huddersfield Royal Infirmary	Denise Hancock	
Huddersfield	Huddersfield Royal Infirmary	Lisa Gledhill	
Huddersfield	Huddersfield Royal Infirmary	Mandy Madigan	
Huddersfield	Huddersfield Royal Infirmary	Rachel Parker	
Huddersfield	Huddersfield Royal Infirmary	Sharon Woolley	
Huddersfield	Huddersfield Royal Infirmary	Stacey Freeth	
Huddersfield	Huddersfield Royal Infirmary	Tracy Wood	
Huddersfield	Huddersfield Royal Infirmary	Ibrar Hussain	
Hull	Princess Royal Hospital (Hull)	Christopher Hamilton	
Hull	Princess Royal Hospital (Hull)	Robert Dealey	PI
Hull	Princess Royal Hospital (Hull)	Emma Bertram	Pharmacist
Hull	Princess Royal Hospital (Hull)	Sarah Moffat	
Hull	Princess Royal Hospital (Hull)	Suzy Bunton	
Hull	Princess Royal Hospital (Hull)	Claire Levesley	
Hull	Princess Royal Hospital (Hull)	Linda Hoggarth	
Ilford	King George Hospital	Ramachandran Subramaniam	
Ilford	King George Hospital	Neil Fisher	
Inverness	Raigmore Hospital	Alison Nicholls	
Inverness	Raigmore Hospital	Anne Marie Pollock	
Inverness	Raigmore Hospital	Charles Kodikara	
Inverness	Raigmore Hospital	Feng Yi Soh	
Inverness	Raigmore Hospital	Marion Paterson	
Inverness	Raigmore Hospital	Neil McPhail	PI
Inverness	Raigmore Hospital	Sin Ting (Amy) Chan	Co-I
Inverness	Raigmore Hospital	Aristoula Papakostidi	

Inverness	Raigmore Hospital	Azmat Sadozye	
Inverness	Raigmore Hospital	Carol Macgregor	
Inverness	Raigmore Hospital	David Whillis	
Inverness	Raigmore Hospital	Kay Kelly	
Inverness	Raigmore Hospital	Martin Russell	
Inverness	Raigmore Hospital	Steve Nicholson	
Inverness	Raigmore Hospital	Denise Campbell	
Inverness	Raigmore Hospital	Graeme Jervis	Pharmacist
Inverness	Raigmore Hospital	Ian Shread	
Inverness	Raigmore Hospital	Michael Loynd	
Inverness	Raigmore Hospital	Jude Madeleine	Pharmacist
Inverness	Raigmore Hospital	Sean Neville	
Inverness	Raigmore Hospital	Sudhir Borgaonkar	
Inverness	Raigmore Hospital	Anglise Addison	
Inverness	Raigmore Hospital	Karina McQuiston	
Inverness	Raigmore Hospital	Mary McKenzie	Pharmacist
Inverness	Raigmore Hospital	Melanie McIlroy	
Inverness	Raigmore Hospital	Rachel Mackay	
Inverness	Raigmore Hospital	Una Taylor	Pharmacist
Inverness	Raigmore Hospital	Alison Macdonald	
Inverness	Raigmore Hospital	Angela Macgregor	
Inverness	Raigmore Hospital	Florence Anderson	
Inverness	Raigmore Hospital	Georgina Simpson	
Inverness	Raigmore Hospital	Jane Campbell	
Inverness	Raigmore Hospital	Sandra Brown	

Inverness	Raigmore Hospital	Seonaid Arnott	
Inverness	Raigmore Hospital	Anna Robertson	
Inverness	Raigmore Hospital	Anna Skene	
Inverness	Raigmore Hospital	Jane Sinclair	
Inverness	Raigmore Hospital	Joan Stewart	
Inverness	Raigmore Hospital	Karen Callum	
Inverness	Raigmore Hospital	Laura Maclennan	
Inverness	Raigmore Hospital	Margaret Chisholm	
Inverness	Raigmore Hospital	Melissa Lynch	
Inverness	Raigmore Hospital	Zoe Urquhart	Pharmacist
Inverness	Raigmore Hospital	Audrey Campbell	
Inverness	Raigmore Hospital	Catriona Morrison	
Inverness	Raigmore Hospital	Fiona Campbell	
Inverness	Raigmore Hospital	Glenda Sinclair	
Inverness	Raigmore Hospital	Susan Bain	
Ipswich	Ipswich Hospital	Deborah Abrams	
Ipswich	Ipswich Hospital	Kevin Redshaw	
Ipswich	Ipswich Hospital	Sam Obay	
Ipswich	Ipswich Hospital	William Ine	Co-I
Ipswich	Ipswich Hospital	Adiba Hoodbhoy	
Ipswich	Ipswich Hospital	Christopher Scrase	
Ipswich	Ipswich Hospital	Jennifer Collins	
Ipswich	Ipswich Hospital	Ramachandran Venkitaraman	PI
Ipswich	Ipswich Hospital	Sarah Treece	
Ipswich	Ipswich Hospital	Sheen Cherian	

Ipswich	Ipswich Hospital	TJ Podd	Co-I
Ipswich	Ipswich Hospital	Debbie Austin	
Ipswich	Ipswich Hospital	Andy Dann	
Ipswich	Ipswich Hospital	Chris Rose	
Ipswich	Ipswich Hospital	Joe Wells	
Ipswich	Ipswich Hospital	Matt Mendoza	
Ipswich	Ipswich Hospital	Gautam Banerjee	
Ipswich	Ipswich Hospital	Ian Floodgate	Pharmacist
Ipswich	Ipswich Hospital	John Parry	
Ipswich	Ipswich Hospital	Mohsen Habib	
Ipswich	Ipswich Hospital	Paul Ridley	
Ipswich	Ipswich Hospital	Peter Donaldson	
Ipswich	Ipswich Hospital	Robert Brierly	PI
Ipswich	Ipswich Hospital	Charlotte Etheridge	
Ipswich	Ipswich Hospital	Karen Bass	
Ipswich	Ipswich Hospital	Kerry Howlett (nee Brown)	Pharmacist
Ipswich	Ipswich Hospital	Mandy Riley (nee Evans)	
Ipswich	Ipswich Hospital	Susan Teh (Seok San)	
Ipswich	Ipswich Hospital	Sonia Kerridge	
Ipswich	Ipswich Hospital	Susan Upson	
Ipswich	Ipswich Hospital	Yvonne Tricker	
Ipswich	Ipswich Hospital	Harriet Williami	
Ipswich	Ipswich Hospital	Jo Woor	
Ipswich	Ipswich Hospital	Julie Simpson	
Ipswich	Ipswich Hospital	Angharad Williams	

Ipswich	Ipswich Hospital	Julie Spurgeon	
Ipswich	Ipswich Hospital	Natalie Lloyd	
Isle of Wight	St Mary's Hospital (Newport)	Elizabeth Harrison	
Keighley	Airedale General Hospital	Ann Henry	
Keighley	Airedale General Hospital	Charlotte Richardson	
Keighley	Airedale General Hospital	Clara Sentamans	
Keighley	Airedale General Hospital	Nathalie Casanova	
Keighley	Airedale General Hospital	Simon Brown	
Keighley	Airedale General Hospital	Sohail Mughal	Co-I
Keighley	Airedale General Hospital	Dan Lee	
Keighley	Airedale General Hospital	Ganesan Jeyasangar	PI
Keighley	Airedale General Hospital	Michael Crawford	
Keighley	Airedale General Hospital	Sue Cheeseman	
Keighley	Airedale General Hospital	Amy Pendrill	
Keighley	Airedale General Hospital	Andrew Gash	
Keighley	Airedale General Hospital	Joseph Quinn	
Keighley	Airedale General Hospital	Satti Saggu	
Keighley	Airedale General Hospital	Carl Booth	Pharmacist
Keighley	Airedale General Hospital	Anita Ratcliffe	
Keighley	Airedale General Hospital	Josie Snell	
Keighley	Airedale General Hospital	Lisa Bullough	
Keighley	Airedale General Hospital	Liz Shenton	
Keighley	Airedale General Hospital	Pip Hill	
Keighley	Airedale General Hospital	Rachel Kennedy	
Keighley	Airedale General Hospital	Sharron Parkinson	

Keighley	Airedale General Hospital	Alison Shaw	
Keighley	Airedale General Hospital	Gillian Darnbrook	Pharmacist
Keighley	Airedale General Hospital	Jasmine Hartley	
Keighley	Airedale General Hospital	Alison Swindells	Pharmacist
Keighley	Airedale General Hospital	Hayley Bates	
Keighley	Airedale General Hospital	Louise Binns	
Keighley	Airedale General Hospital	Ruth Johnson	
Keighley	Airedale General Hospital	Fiona Farquhar	
Keighley	Airedale General Hospital	Helen Henson	
Keighley	Airedale General Hospital	Judy McAlister	
Keighley	Airedale General Hospital	Maxine Briggs	
Keighley	Airedale General Hospital	Mandy Swanepoel	
Kidderminster	Kidderminster General Hospital	Ayyaz Munawar	Co-I
Kidderminster	Kidderminster General Hospital	Kirsty Clarke	Co-I
Kidderminster	Kidderminster General Hospital	M Habib Khan	
Kidderminster	Kidderminster General Hospital	Shaikh Rana	Co-I
Kidderminster	Kidderminster General Hospital	Lisa Capaldi	PI
Kidderminster	Kidderminster General Hospital	Mark Churn	
Kidderminster	Kidderminster General Hospital	Paul Flinders	Co-I
Kidderminster	Kidderminster General Hospital	Hugh Morrow	
Kidderminster	Kidderminster General Hospital	Monica Gauntlett	Pharmacist
Kidderminster	Kidderminster General Hospital	Veronica Rowlands	
Kidderminster	Kidderminster General Hospital	Emma Marshall	
Kidderminster	Kidderminster General Hospital	Hayley Hodson	
Kidderminster	Kidderminster General Hospital	Helen Tranter	

Kidderminster	Kidderminster General Hospital	Julie Wollaston	
Kidderminster	Kidderminster General Hospital	Kate Field	
Kidderminster	Kidderminster General Hospital	Patricia Rimell	
Kidderminster	Kidderminster General Hospital	Sarah Moss	
Kidderminster	Kidderminster General Hospital	Sally Stringer (pr. Davis)	
Kidderminster	Kidderminster General Hospital	Alison Rosoman	
Kidderminster	Kidderminster General Hospital	Jayne Tyler	
Kidderminster	Kidderminster General Hospital	Linda Higgins	
Kilmarnock	Ayr Hospital	Ricky Hunter	Pharmacist
Kilmarnock	Ayr Hospital	Jennifer Keith	
Kilmarnock	Crosshouse Hospital	Margaret McKernan	
Kilmarnock	Ayr Hospital	Maureen Templeton	
Lancaster	Royal Lancaster Infirmary	Sophie Raby	
Larbert	Forth Valley Royal Hospital	Adam Peters	
Larbert	Forth Valley Royal Hospital	Amy Martin	
Larbert	Forth Valley Royal Hospital	Caroline Lowrie	
Larbert	Forth Valley Royal Hospital	John Martin Russell	
Larbert	Forth Valley Royal Hospital	Saurabh Borgaonkar	
Larbert	Forth Valley Royal Hospital	Stephen McKay	
Larbert	Forth Valley Royal Hospital	Carolynn Lamb	
Larbert	Forth Valley Royal Hospital	Nadja Melquiot	
Larbert	Forth Valley Royal Hospital	Norma Sidek	PI
Larbert	Forth Valley Royal Hospital	Eilidh Henderson	
Larbert	Forth Valley Royal Hospital	Kirsty Young	
Larbert	Forth Valley Royal Hospital	Mhari TTaylor	

Larbert	Forth Valley Royal Hospital	Stephanie Brogan (nee Roddie)	
Larbert	Forth Valley Royal Hospital	Seamus Teahan	
Larbert	Forth Valley Royal Hospital	Anna Hamilton	
Larbert	Forth Valley Royal Hospital	Anne Todd	
Larbert	Forth Valley Royal Hospital	Joanne Robinson	Pharmacist
Larbert	Forth Valley Royal Hospital	Lesley Symon	
Larbert	Forth Valley Royal Hospital	Patricia Turner	
Larbert	Forth Valley Royal Hospital	Lynn Prentice	
Larbert	Forth Valley Royal Hospital	Maureen Hamill	
Larbert	Forth Valley Royal Hospital	Sally Young	
Larbert	Forth Valley Royal Hospital	Alison Yule	
Larbert	Beatson West of Scotland Cancer Centre	Maureen Hamill	
Lausanne	Centre Hospitalier Universitaire Vaudois (CHUV)	Geert Van Driessche	
Lausanne	Centre Hospitalier Universitaire Vaudois (CHUV)	Benangene Midez	
Lausanne	Centre Hospitalier Universitaire Vaudois (CHUV)	Angela Orcurto	
Lausanne	Centre Hospitalier Universitaire Vaudois (CHUV)	Antonella Diciolla	
Lausanne	Centre Hospitalier Universitaire Vaudois (CHUV)	Claire Perrinjaquet	
Lausanne	Centre Hospitalier Universitaire Vaudois (CHUV)	Dominik Berthold	PI
Lausanne	Centre Hospitalier Universitaire Vaudois (CHUV)	Louis Parisod	
Lausanne	Centre Hospitalier Universitaire Vaudois (CHUV)	Sofiya Latifyan	
Lausanne	Centre Hospitalier Universitaire Vaudois (CHUV)	Fernanda Herrera	
Lausanne	Centre Hospitalier Universitaire Vaudois (CHUV)	Akram Farhat	
Lausanne	Centre Hospitalier Universitaire Vaudois (CHUV)	Yohan Boillat	
Lausanne	Centre Hospitalier Universitaire Vaudois (CHUV)	Alexandra Rideau	
Lausanne	Centre Hospitalier Universitaire Vaudois (CHUV)	Fabrice Lalubin	

Lausanne	Centre Hospitalier Universitaire Vaudois (CHUV)	Hans-peter Roth	
Lausanne	Centre Hospitalier Universitaire Vaudois (CHUV)	Jean-Philippe Zurcher	
Lausanne	Centre Hospitalier Universitaire Vaudois (CHUV)	Tewfik Abedlaziz	
Lausanne	Centre Hospitalier Universitaire Vaudois (CHUV)	Kaniana Ntanga Muambayi	
Lausanne	Centre Hospitalier Universitaire Vaudois (CHUV)	Sandra Toffanin	
Lausanne	Centre Hospitalier Universitaire Vaudois (CHUV)	Agnes Hiou Feige	
Lausanne	Centre Hospitalier Universitaire Vaudois (CHUV)	Alice Abdallah	
Lausanne	Centre Hospitalier Universitaire Vaudois (CHUV)	Aline Voidey	
Lausanne	Centre Hospitalier Universitaire Vaudois (CHUV)	Anabela Costa	
Lausanne	Centre Hospitalier Universitaire Vaudois (CHUV)	Anna-Sophia Briod	
Lausanne	Centre Hospitalier Universitaire Vaudois (CHUV)	Carmen Castagna	
Lausanne	Centre Hospitalier Universitaire Vaudois (CHUV)	Catherine Bender	
Lausanne	Centre Hospitalier Universitaire Vaudois (CHUV)	Celine Yerly	
Lausanne	Centre Hospitalier Universitaire Vaudois (CHUV)	Cosette Schuler	
Lausanne	Centre Hospitalier Universitaire Vaudois (CHUV)	Cynthia Leclerc	
Lausanne	Centre Hospitalier Universitaire Vaudois (CHUV)	Eloise Kremer	
Lausanne	Centre Hospitalier Universitaire Vaudois (CHUV)	Floriane Bouilly	
Lausanne	Centre Hospitalier Universitaire Vaudois (CHUV)	Margaret McLauchlan	
Lausanne	Centre Hospitalier Universitaire Vaudois (CHUV)	May-Lucie Meyer	Co-I
Lausanne	Centre Hospitalier Universitaire Vaudois (CHUV)	Nathalie Divorne	
Lausanne	Centre Hospitalier Universitaire Vaudois (CHUV)	Nicole James Faresse	
Lausanne	Centre Hospitalier Universitaire Vaudois (CHUV)	Norlene Silva	
Lausanne	Centre Hospitalier Universitaire Vaudois (CHUV)	Rebecca Oppenheim	
Lausanne	Centre Hospitalier Universitaire Vaudois (CHUV)	Sophie Voegtlin	
Lausanne	Centre Hospitalier Universitaire Vaudois (CHUV)	Sylvie Haudidier	

Lausanne	Centre Hospitalier Universitaire Vaudois (CHUV)	Veronica Aedo	
Lausanne	Centre Hospitalier Universitaire Vaudois (CHUV)	Patrice Jichlinski	
Lausanne	Centre Hospitalier Universitaire Vaudois (CHUV)	Galaad Bernard	
Lausanne	Centre Hospitalier Universitaire Vaudois (CHUV)	Marc Schnety	
Lausanne	Centre Hospitalier Universitaire Vaudois (CHUV)	Sabine Galland	
Lausanne	Centre Hospitalier Universitaire Vaudois (CHUV)	Sophia Murel	
Leeds	St James University Hospital (Leeds)	Ann Henry	
Leeds	St James University Hospital (Leeds)	Ann Henry	
Leeds	St James University Hospital (Leeds)	Christopher Williams	
Leeds	St James University Hospital (Leeds)	Christy Ralph	
Leeds	Bradford Royal Infirmary	Ee Siang Choong	
Leeds	Bradford Royal Infirmary	Emma Dugdale	
Leeds	St James University Hospital (Leeds)	Ian Boon	
Leeds	St James University Hospital (Leeds)	Joseph Joji	
Leeds	Airedale General Hospital	Katy Clarke	
Leeds	Bradford Royal Infirmary	Katy Clarke	
Leeds	Bradford Royal Infirmary	Michael Flatley	Co-I
Leeds	St James University Hospital (Leeds)	Naveen Vasudev	Co-I
Leeds	Cookridge Hospital	Anne Kiltie	
Leeds	St James University Hospital (Leeds)	Anne Kiltie	
Leeds	St James University Hospital (Leeds)	Carmel Loughrey	
Leeds	St James University Hospital (Leeds)	David Bottomley	
Leeds	St James University Hospital (Leeds)	Hima Bindu Musunuru	
Leeds	St James University Hospital (Leeds)	Janet Brown	
Leeds	St James University Hospital (Leeds)	John Chester	

Leeds	St James University Hospital (Leeds)	Kevin Franks	
Leeds	St James University Hospital (Leeds)	Krishna Shastry	
Leeds	Bradford Royal Infirmary	Lisa Owen	Co-I
Leeds	St James University Hospital (Leeds)	Luis Daverede	
Leeds	St James University Hospital (Leeds)	Richard Khafagy	
Leeds	St James University Hospital (Leeds)	Satinder Jagdev	
Leeds	St James University Hospital (Leeds)	Claire Posnett	
Leeds	St James University Hospital (Leeds)	Hannah Roberts	
Leeds	St James University Hospital (Leeds)	Javeria Akhtar	
Leeds	St James University Hospital (Leeds)	Jodene Hill	
Leeds	St James University Hospital (Leeds)	Judith Evans	
Leeds	St James University Hospital (Leeds)	Svetoslava Doshmanonska	
Leeds	St James University Hospital (Leeds)	Liz Hudson	
Leeds	St James University Hospital (Leeds)	Christopher Main	
Leeds	St James University Hospital (Leeds)	Ruiyang Yan	
Leeds	St James University Hospital (Leeds)	Sanjeev Kotwal	
Leeds	St James University Hospital (Leeds)	William Cross	PI
Leeds	Leeds General Infirmary	Adrian Joyce	
Leeds	St James University Hospital (Leeds)	Adrian Joyce	
Leeds	St James University Hospital (Leeds)	Alan Paul	
Leeds	St James University Hospital (Leeds)	James Cavanagh	
Leeds	St James University Hospital (Leeds)	Peter Whelan	PI
Leeds	St James University Hospital (Leeds)	Rafal Turo	
Leeds	St James University Hospital (Leeds)	Sam Lotfi	
Leeds	St James University Hospital (Leeds)	Stephen Prescott	

Leeds	St James University Hospital (Leeds)	Sunjay Jain	
Leeds	St James University Hospital (Leeds)	Catherine Parbutt	
Leeds	St James University Hospital (Leeds)	Jude Clarke	
Leeds	St James University Hospital (Leeds)	Lorraine Wiseman	
Leeds	St James University Hospital (Leeds)	Polapo Ajayi	
Leeds	St James University Hospital (Leeds)	Angela Morgan	
Leeds	St James University Hospital (Leeds)	Anne Crossley	
Leeds	St James University Hospital (Leeds)	Dolapo Ajayi	
Leeds	St James University Hospital (Leeds)	Hannah Wigginton	
Leeds	St James University Hospital (Leeds)	Lorraine Wiseman	
Leeds	Leeds General Infirmary	Caroline Bedford	
Leeds	St James University Hospital (Leeds)	Caroline Bedford	Pharmacist
Leeds	St James University Hospital (Leeds)	Charlotte Pool	
Leeds	St James University Hospital (Leeds)	Emily Davies	
Leeds	St James University Hospital (Leeds)	Emma Lundy	Pharmacist
Leeds	St James University Hospital (Leeds)	Gemma Austin (nee Glover)	
Leeds	St James University Hospital (Leeds)	Helen Payne	
Leeds	St James University Hospital (Leeds)	Jade McCann	
Leeds	St James University Hospital (Leeds)	Judith Chapman	
Leeds	St James University Hospital (Leeds)	Maria Hall	
Leeds	St James University Hospital (Leeds)	Mercy Kaiga	
Leeds	St James University Hospital (Leeds)	Sue Rodwell	
Leeds	St James University Hospital (Leeds)	Sue Sibson	
Leeds	Cookridge Hospital	Richard Kaplan	
Leeds	St James University Hospital (Leeds)	Richard Kaplan	

Liestal	Kantonsspital Liestal	Eloise Kremer	
Liestal	Kantonsspital Liestal	Simone Marini	
Liestal	Kantonsspital Liestal	Vanessa Fuhrer	
Liestal	Kantonsspital Liestal	Andreas Lohri	
Lincoln	Lincoln County Hospital	Ana Fernandez-Ots	Co-I
Lincoln	Lincoln County Hospital	Andrew Sloan	
Lincoln	Lincoln County Hospital	Christian Arias	
Lincoln	Lincoln County Hospital	David Ballesteros-Quintail	
Lincoln	Lincoln County Hospital	Elena Macleod	
Lincoln	Lincoln County Hospital	Sindhu Ramarwothy	Co-I
Lincoln	Lincoln County Hospital	Yogesh Nishchal	
Lincoln	Lincoln County Hospital	Alfredo Addeo	
Lincoln	Lincoln County Hospital	Karin Baria	
Lincoln	Lincoln County Hospital	Miguel Panades	PI
Lincoln	Lincoln County Hospital	Prantik Das	Co-I
Lincoln	Lincoln County Hospital	Thiagarajan Sreenivasan	
Lincoln	Pilgrim Hospital	Thiagarajan Sreenivasan	
Lincoln	Lincoln County Hospital	Gunjan Phalod	
Lincoln	Lincoln County Hospital	Andrew Judd	
Lincoln	Lincoln County Hospital	Ray McDermott	
Lincoln	Lincoln County Hospital	Stephen Audu	Pharmacist
Lincoln	Lincoln County Hospital	Simon Archer	
Lincoln	Lincoln County Hospital	Alyson Wilson	
Lincoln	Lincoln County Hospital	Diane Carey	
Lincoln	Lincoln County Hospital	Kathryn Pearson	

Lincoln	Lincoln County Hospital	Kerri Johnson	
Lincoln	Lincoln County Hospital	Laura Walsh	
Lincoln	Lincoln County Hospital	Maryanne Okubanjo	
Lincoln	Lincoln County Hospital	Olesya Francis	
Lincoln	Lincoln County Hospital	Caroline Taylor	Pharmacist
Lincoln	Lincoln County Hospital	Suzanne Archer	
Lincoln	Lincoln County Hospital	Giuseppe Banna	
Lincoln	Lincoln County Hospital	Helen Carolan	
Lincoln	Lincoln County Hospital	Janet Tomlinson	
Lincoln	Lincoln County Hospital	Jayne Borley	Pharmacist
Lincoln	Lincoln County Hospital	Jenny Salmon	
Lincoln	Lincoln County Hospital	Kathryn Hoare	
Lincoln	Lincoln County Hospital	Rebecca Spencer	
Lincoln	Lincoln County Hospital	Rhiannan Pegg	
Lincoln	Lincoln County Hospital	Sarah Bell	Pharmacist
Lincoln	Lincoln County Hospital	Sarah Coombs	
Lincoln	Lincoln County Hospital	Stephanie Barker	
Lincoln	Lincoln County Hospital	Annette Hilldrith	
Lincoln	Lincoln County Hospital	Carol Lockwood	
Lincoln	Lincoln County Hospital	Rachel Newton	
Lincoln	Lincoln County Hospital	Susie Butler	
Liverpool	Royal Liverpool University Hospital	Jasima Latif	
Liverpool	Royal Liverpool University Hospital	Chinnamani Eswar	Co-I
Liverpool	University Hospital Aintree	Paul Hill	
Liverpool	Royal Liverpool University Hospital	Peter Robson	

Liverpool	University Hospital Aintree	Peter Robson	PI
Liverpool	Royal Liverpool University Hospital	Zafar Malik	PI
Liverpool	University Hospital Aintree	Lucy Berresford	
Liverpool	University Hospital Aintree	Rachael Fergusson	
Liverpool	Royal Liverpool University Hospital	Katy Treherne	
Liverpool	Royal Liverpool University Hospital	Kevin McDonald	
Liverpool	Royal Liverpool University Hospital	Paul Griffiths	
Liverpool	Royal Liverpool University Hospital	Philip Reynolds	
Liverpool	University Hospital Aintree	Wesley Artist	
Liverpool	University Hospital Aintree	Lorraine Lancaster	
Liverpool	Royal Liverpool University Hospital	Nidhi Sibal	
Liverpool	Royal Liverpool University Hospital	Sharon Dunn (nee Johnson)	
Liverpool	Royal Liverpool University Hospital	Sue Green	
Liverpool	Royal Liverpool University Hospital	Sandra Robinson	
Liverpool	University Hospital Aintree	Sandra Robinson	Pharmacist
Liverpool	Royal Liverpool University Hospital	Nicola Bermingham	
Liverpool	Royal Liverpool University Hospital	Pembe Yesildag	
Liverpool	Royal Liverpool University Hospital	Dawn Porter	
Liverpool	University Hospital Aintree	Haley McCulloch	
Liverpool	Royal Liverpool University Hospital	Julie Griffiths	
Liverpool	University Hospital Aintree	Julie Griffiths	
Liverpool	University Hospital Aintree	Leigh Pauls	
Liverpool	Royal Liverpool University Hospital	Lisa Dobson (nee Child)	
Liverpool	University Hospital Aintree	Lisa Dobson (nee Child)	Pharmacist
Liverpool	Royal Liverpool University Hospital	Lizzie Dale	

Liverpool	Royal Liverpool University Hospital	Pauline Pilkington	
London	Royal Marsden Hospital (London)	Adnan Akhtar	
London	Guy's Hospital (London)	Ajay Aggarwal	
London	University College Hospital	Anita Mitra	
London	Royal Marsden Hospital (London)	Anna Wilkins	
London	University College Hospital	Anuradha Jayaram	
London	University College Hospital	Bianca Tryillo	
London	University College Hospital	Bihani Kularatne	
London	Guy's Hospital (London)	Caterina Aversa	
London	St Bartholomews Hospital (London)	Cavitha Vivekananthan	
London	Guy's Hospital (London)	Chara Stavraka	Co-I
London	Guy's Hospital (London)	Charalampos Gousis	
London	Guy's Hospital (London)	Charleen Chan Wah Hak	
London	Guy's Hospital (London)	Clare Gilson	
London	Guy's Hospital (London)	Deborah Enting	
London	Guy's Hospital (London)	Delali Adjogatse	
London	University College Hospital	Dieo Ottaviani	
London	Guy's Hospital (London)	Eirini Tsotra	
London	Guy's Hospital (London)	Eleni Josephides	
London	Guy's Hospital (London)	Elias Pintus	
London	Queen Elizabeth Hospital (Woolwich)	Elias Pintus	Co-I
London	Royal Marsden Hospital (London)	Emily Durie	
London	Royal Free Hospital	Emily Scott	Co-I
London	Royal Marsden Hospital (London)	Ewan Chapman	Co-I
London	St Georges Hospital (London)	Gelareh Eslamian	

London	University College Hospital	Gianmarco Leone	
London	Royal Free Hospital	Grant Stewart	Co-I
London	Guy's Hospital (London)	Hannah Rush	
London	Royal Marsden Hospital (London)	James Lowe	
London	Royal Marsden Hospital (London)	Jana McHugh	
London	Guy's Hospital (London)	Jennifer Turner	
London	Royal Marsden Hospital (London)	Kallol Bhadra	
London	Guy's Hospital (London)	Kamarul Zaki	
London	St Bartholomews Hospital (London)	Karen Tipples	PI
London	Royal Free Hospital	Kate Smith	Co-I
London	Guy's Hospital (London)	Kiruthikah Thillai	
London	St Georges Hospital (London)	Laura Camburn	Co-I
London	Royal Marsden Hospital (London)	Leron Okonta	
London	Royal Free Hospital	Magdalena Kubiak	Co-I
London	University College Hospital	Maise Albakir	
London	Guy's Hospital (London)	Mark Voskoboynik	
London	Guy's Hospital (London)	Matthaius Kapiris	
London	St Georges Hospital (London)	Mohammed Mahgoub	
London	Guy's Hospital (London)	Muhammad Khan	
London	Royal Marsden Hospital (London)	Nicholas Vanas	
London	Royal Free Hospital	Nicola Rosenfelder	Co-I
London	Guy's Hospital (London)	Nikolaos Tsoukalas	
London	Charing Cross Hospital	Paul Kabuubi	
London	Guy's Hospital (London)	Rosalind Kieran	
London	St Georges Hospital (London)	Roxane Mather	

London	Royal Free Hospital	Ruochen Li	
London	Guy's Hospital (London)	Sabeeh Butt	
London	Guy's Hospital (London)	Sarah Howiett	Co-I
London	Royal Free Hospital	Sarah Needleman	PI
London	Charing Cross Hospital	Stephen Mangar	Co-I
London	Hammersmith Hospital	Stephen Mangar	Co-I
London	St Marys Hospital (London)	Stephen Mangar	
London	Guy's Hospital (London)	Stephen Morris	
London	Guy's Hospital (London)	Thomas Bird	
London	Guy's Hospital (London)	Thubeena Manickavasagar	
London	Royal Marsden Hospital (London)	Tzveta porrovska	
London	Royal Marsden Hospital (London)	Vedang Murthy	
London	Guy's Hospital (London)	Vinod Mullassery	
London	Queen Elizabeth Hospital (Woolwich)	Vinod Mullassery	Co-I
London	Guy's Hospital (London)	Vishal Manik	Co-I
London	Guy's Hospital (London)	Yin Wu	
London	Charing Cross Hospital	Alison Falconer	PI
London	Hammersmith Hospital	Alison Falconer	PI
London	St Marys Hospital (London)	Alison Falconer	PI
London	Royal Marsden Hospital (London)	Alison Reid	
London	Guy's Hospital (London)	Angel Garcia-Imhof	
London	Guy's Hospital (London)	Anna Karpathakis	
London	Guy's Hospital (London)	Archie Macnair	
London	Queen Elizabeth Hospital (Woolwich)	Arunansu Kar	
London	North Middlesex Hospital	Asim Ray	

London	Royal Free Hospital	Daniel Smith	
London	Guy's Hospital (London)	Daniel Tong	
London	Guy's Hospital (London)	Danielle Crawley	
London	Guy's Hospital (London)	Debra Josephs	
London	Charing Cross Hospital	Ethna Mannion	
London	North Middlesex Hospital	Farhad Neave	
London	Guy's Hospital (London)	Hartmut Kristeleit	
London	Queen Elizabeth Hospital (Woolwich)	Hartmut Kristeleit	
London	University College Hospital	Heather Payne	Co-I
London	North Middlesex Hospital	Jackie Newby	
London	St Georges Hospital (London)	Jason Chow	Co-I
London	St Bartholomews Hospital (London)	Jonathon Shamash	
London	University College Hospital	Judith Cave	
London	Royal Marsden Hospital (London)	Karen Chan	
London	Royal Free Hospital	Katherine Pigott	
London	Guy's Hospital (London)	Lawrence Krieger	
London	St Georges Hospital (London)	Lisa Pickering	
London	Guy's Hospital (London)	Lucy Juggins	
London	Royal Free Hospital	Maria Vilarino-Varela	PI
London	University College Hospital	Mark Linch	Co-I
London	St Georges Hospital (London)	Mehran Afshar	PI
London	Charing Cross Hospital	Naveed Sarwar	
London	Royal Marsden Hospital (London)	Nicholas Van As	
London	Queen Elizabeth Hospital (Woolwich)	Nick Maisey	
London	St Bartholomews Hospital (London)	Paula Wells	Co-I

London	Guy's Hospital (London)	Ramin Ajami	
London	University College Hospital	Reena Davda	
London	Guy's Hospital (London)	Ronald Beaney	
London	Royal Marsden Hospital (London)	Rosalind Eeles	
London	Guy's Hospital (London)	Rushan Sylva	
London	Guy's Hospital (London)	Sarah Hargreaves	
London	Guy's Hospital (London)	Sarah Rudman	PI
London	Guy's Hospital (London)	Sharmistha Ghosh	
London	Guy's Hospital (London)	Sheeba Irshad	
London	Guy's Hospital (London)	Simon Chowdhury	
London	Guy's Hospital (London)	Simon Hughes	
London	Queen Elizabeth Hospital (Woolwich)	Simon Hughes	
London	St Marys Hospital (London)	Simon Stewart	
London	Queen Elizabeth Hospital (Woolwich)	Sindu Vivekanandan	PI
London	St Georges Hospital (London)	Sophie McGrath	
London	St Bartholomews Hospital (London)	Stephanie Gibbs	
London	University College Hospital	Stephen Harland	
London	Guy's Hospital (London)	Susanne Allan	
London	Guy's Hospital (London)	Teresa Guerrero-Urbano	
London	University College Hospital	Ursula McGovern	PI
London	University College Hospital	Uzma Asghar	
London	Guy's Hospital (London)	Vasiliki Michalarea	
London	Royal Marsden Hospital (London)	Vincent Khoo	PI
London	St Bartholomews Hospital (London)	Wing-Kin Liu	
London	University College Hospital	Thomas Amoaten	

London	St Georges Hospital (London)	Alice Dainty	
London	Guy's Hospital (London)	Anita Soma	Pharmacist
London	St Georges Hospital (London)	Asha Mistry	
London	St Georges Hospital (London)	Claire Gilmartin	
London	Royal Marsden Hospital (London)	Eleanor Quinn	
London	Royal Free Hospital	Emma Douch	
London	St Bartholomews Hospital (London)	Hannah Payne	
London	Royal Marsden Hospital (London)	Holly Hogan	
London	Royal Marsden Hospital (London)	Jennyfa Ali	
London	Royal Free Hospital	Jessica Hunt	
London	Charing Cross Hospital	Kerry Richards	
London	Royal Free Hospital	Kharishma Makani	
London	Guy's Hospital (London)	Louisa McDonald	
London	University College Hospital	Martha Wilson	
London	Royal Marsden Hospital (London)	Nicola Lucas	
London	University College Hospital	Nicole Bonsu	
London	St Bartholomews Hospital (London)	Olivia Bolton	
London	University College Hospital	Samirah Rokib	
London	Guy's Hospital (London)	Sarah King	
London	St Georges Hospital (London)	Serena Dover	
London	St Bartholomews Hospital (London)	Sultana Begum	
London	Guy's Hospital (London)	Tahereh Ghadimi	
London	St Georges Hospital (London)	Uforma Ogrigri	Pharmacist
London	Royal Marsden Hospital (London)	Vijitha Vijayakumar	
London	Guy's Hospital (London)	Vivien Quan	

London	Queen Elizabeth Hospital (Woolwich)	Melody Ncube	
London	Queen Elizabeth Hospital (Woolwich)	Abel Jalloh	
London	Queen Elizabeth Hospital (Woolwich)	Abhijit Jadhav	
London	Guy's Hospital (London)	Antonio Querol-Rubiera	
London	Guy's Hospital (London)	Declan Cahill	
London	Guy's Hospital (London)	Fahim Ahmed	
London	St Bartholomews Hospital (London)	Fatjon Dekaj	
London	Charing Cross Hospital	Gareth Barker	Pharmacist
London	St Marys Hospital (London)	Gareth Barker	
London	St Georges Hospital (London)	Geoffrey Howell	
London	St Georges Hospital (London)	Hakim Guessous	
London	University College Hospital	Ignacio Blanch	
London	Queen Elizabeth Hospital (Woolwich)	Jagdev Bains	Pharmacist
London	University College Hospital	Joel Watson	
London	St Bartholomews Hospital (London)	Jude Nixon	
London	St Georges Hospital (London)	Juel Tuazon	
London	University College Hospital	Kristian Warnes	Pharmacist
London	Royal Marsden Hospital (London)	Li Wancheung	
London	Queen Elizabeth Hospital (Woolwich)	Luke Maidment	
London	St Georges Hospital (London)	Michael Brown	
London	Royal Marsden Hospital (London)	Michael Moneypenny	
London	St Bartholomews Hospital (London)	Oscar Riches	
London	St Bartholomews Hospital (London)	P Cathcart	
London	Guy's Hospital (London)	Rayhan Ahmed	
London	Queen Elizabeth Hospital (Woolwich)	Rayhan Ahmed	

London	Guy's Hospital (London)	Rick Popert	
London	St Georges Hospital (London)	Robert Varro	
London	Charing Cross Hospital	Ross Dalton-Short	
London	St Georges Hospital (London)	Sam Hollingworth	
London	St Bartholomews Hospital (London)	Sebastien Martin	
London	Whittington Hospital (London)	Simon Wan	
London	Guy's Hospital (London)	Sumeet Sisodia	
London	Queen Elizabeth Hospital (Woolwich)	Vinod Mullessey	PI
London	St Bartholomews Hospital (London)	Alastair Nicholson	
London	Royal Marsden Hospital (London)	Bernard Sill	
London	Royal Marsden Hospital (London)	Bernard Siu	
London	North Middlesex Hospital	Chris Abbott	Pharmacist
London	University College Hospital	Danny Garrett	
London	Guy's Hospital (London)	Gerry Trillana	
London	Hammersmith Hospital	Ilyas Ali	
London	Guy's Hospital (London)	Jozer Calara	
London	Charing Cross Hospital	Kwame Ansu	
London	Queen Elizabeth Hospital (Woolwich)	Lee Porin	
London	St Georges Hospital (London)	Mark Quarrell	
London	Queen Elizabeth Hospital (Woolwich)	Nigel Holmes	
London	Guy's Hospital (London)	Philip Reynolds	
London	Queen Elizabeth Hospital (Woolwich)	Philip Reynolds	
London	Charing Cross Hospital	Steve Edwards	
London	Hammersmith Hospital	Steve Edwards	
London	St Marys Hospital (London)	Steve Edwards	

London	Queen Elizabeth Hospital (Woolwich)	Thomas Sarkodie	
London	Guy's Hospital (London)	Thomas Spencer	
London	Guy's Hospital (London)	Trevor Bott	
London	Royal Marsden Hospital (London)	Trevor Bott	
London	University College Hospital	Adrienne Abioye	
London	Queen Elizabeth Hospital (Woolwich)	Joyce Maravi	
London	Royal Free Hospital	Kaliyanee Ramtohul	
London	Guy's Hospital (London)	Ngozi Muoneke	
London	St Georges Hospital (London)	Nia Alsamarrai	
London	St Bartholomews Hospital (London)	Resmi Jayachandran	
London	University College Hospital	Roshni Goel	
London	Royal Marsden Hospital (London)	Ruth Stafferton	
London	St Bartholomews Hospital (London)	Samantha Chetiyawardana	
London	Queen Elizabeth Hospital (Woolwich)	Samia Pilgrim	
London	Royal Marsden Hospital (London)	Sarah Storrs	
London	Charing Cross Hospital	Zohanon Sabine Loko	
London	St Marys Hospital (London)	Zohanon Sabine Loko	
London	Charing Cross Hospital	Andrea Davis-Cook	Pharmacist
London	St Marys Hospital (London)	Andrea Davis-Cook	
London	Guy's Hospital (London)	Chi Yee Chung	Pharmacist
London	Royal Marsden Hospital (London)	Debra Townsend-Thorn	
London	University College Hospital	Holly Baker (nee. Wing)	Pharmacist
London	University College London	Holly Baker (nee. Wing)	
London	Hammersmith Hospital	Regina Storch	Pharmacist
London	Guy's Hospital (London)	Sharon McPherson	

London	Royal Marsden Hospital (London)	Sijy Pillai	
London	Royal Free Hospital	Aarti Nandani	Pharmacist
London	Queen Elizabeth Hospital (Woolwich)	Aarti Shah	
London	University Hospital Lewisham	Aarti Shah	
London	University College Hospital	Agnieska Zielonka	
London	University College Hospital	Aileen Austria	
London	Royal Free Hospital	Anna Osadcow	
London	St Georges Hospital (London)	Anne Haldeos	
London	Queen Elizabeth Hospital (Woolwich)	Anne-Marie Vindidu	Pharmacist
London	Royal Marsden Hospital (London)	Annette Musallam	
London	Royal Marsden Hospital (London)	Asma Varachia	
London	Guy's Hospital (London)	Awo Abdi	
London	Charing Cross Hospital	Bindu Chikkamuniyappa	
London	St Marys Hospital (London)	Bindu Chikkamuniyappa	
London	St Georges Hospital (London)	Chandni Patel	
London	Guy's Hospital (London)	Claire Glendon	
London	Royal Free Hospital	Claire Jarvis	
London	St Georges Hospital (London)	Deirdre Daly	
London	St Bartholomews Hospital (London)	Denise Humfress	
London	Guy's Hospital (London)	Emma O'Connor	
London	Guy's Hospital (London)	Eva Batovska	
London	Royal Marsden Hospital (Sutton)	Eva Batovska	Pharmacist
London	Royal Marsden Hospital (London)	Giulia Carlino	
London	Royal Free Hospital	Hannah Powell	
London	Royal Marsden Hospital (London)	Helen Stidwell	

London	University College Hospital	Helene Zilkha	
London	St Georges Hospital (London)	Jane Gregg	
London	University College Hospital	Jane Leach	Pharmacist
London	University College Hospital	Javeria Akhtar	
London	Queen Elizabeth Hospital (Woolwich)	Jennifer Martin	Pharmacist
London	Royal Marsden Hospital (London)	Juliet Owusu	
London	Guy's Hospital (London)	Kafui Dossa	
London	Royal Marsden Hospital (London)	Karen Brooks	
London	Royal Marsden Hospital (London)	Lexi Vick	
London	Guy's Hospital (London)	Linda Shephard	
London	Royal Free Hospital	Lorna O'Shea	
London	Guy's Hospital (London)	Louisa Fleure	
London	St Bartholomews Hospital (London)	Marina Baccarini	
London	Royal Marsden Hospital (London)	Marisa Pinto Peixoto	
London	Queen Elizabeth Hospital (Woolwich)	Martha Handousa	
London	Queen Elizabeth Hospital (Woolwich)	Miriam Cottle	
London	Queen Elizabeth Hospital (Woolwich)	Nadia El-Sayed	
London	Royal Free Hospital	Naomi Anderson	
London	University College Hospital	Natasha Aslam	
London	Royal Marsden Hospital (London)	Nicola Harman	
London	Hammersmith Hospital	Nikki Kettley	
London	Guy's Hospital (London)	Rebecca Way	
London	Royal Free Hospital	Sabina Melander	
London	Queen Elizabeth Hospital (Woolwich)	Sagira Khatun	Pharmacist
London	Guy's Hospital (London)	Sally Walker	

London	St Marys Hospital (London)	Severine Rey	
London	St Bartholomews Hospital (London)	Shahanara Ferdous	
London	Queen Elizabeth Hospital (Woolwich)	Shahreen Ahmed	
London	Queen Elizabeth Hospital (Woolwich)	Shanna Wilson	
London	Guy's Hospital (London)	Susie Slater	
London	Queen Elizabeth Hospital (Woolwich)	Suzanne Chukundah	
London	University College Hospital	Suzy Lowi	
London	Royal Free Hospital	Tesha Suddason	
London	Queen Elizabeth Hospital (Woolwich)	Theodorah Nago	
London	University College Hospital	Yemi Ilumoka	
London	Charing Cross Hospital	Akeema Paul	
London	St Marys Hospital (London)	Akeema Paul	
London	St Marys Hospital (London)	Angela Chamberlain	
London	Royal Free Hospital	Angela McCadden	
London	Guy's Hospital (London)	Anna Parker	
London	University College Hospital	Annelies Gillesen	
London	Guy's Hospital (London)	Belinda Chitando	
London	Queen Elizabeth Hospital (Woolwich)	Belinda Chitando	
London	Queen Elizabeth Hospital (Woolwich)	Bridget Kabagambe	
London	Guy's Hospital (London)	Catherine Rogers	
London	Guy's Hospital (London)	Cheryl Lawrence	
London	St Bartholomews Hospital (London)	Cheryl Lawrence	Pharmacist
London	Royal Marsden Hospital (London)	Chloe McCormack	
London	Charing Cross Hospital	Daisy Floyd	
London	St Marys Hospital (London)	Daisy Floyd	

London	North Middlesex Hospital	Debbie Blois	
London	Royal Marsden Hospital (London)	Debbie Rolfe	
London	St Georges Hospital (London)	Debbie Rolfe	
London	University College Hospital	Didem Agdiran	
London	Guy's Hospital (London)	Donna Cassidy	
London	Royal Free Hospital	Elizabeth Woodford	Pharmacist
London	Guy's Hospital (London)	Emilia Caverly	
London	Hammersmith Hospital	Emily Pickford	
London	Queen Elizabeth Hospital (Woolwich)	Eti Omoregie	
London	Guy's Hospital (London)	Francesca Curran	
London	St Marys Hospital (London)	Gillian Hornzee	
London	University College Hospital	Hannah Ansell	Pharmacist
London	Queen Elizabeth Hospital (Woolwich)	Hazel Harrop	
London	Guy's Hospital (London)	Helen Snow	
London	St Georges Hospital (London)	Helen Tighe	
London	Charing Cross Hospital	Ibiyemi Sadare (Olaleye)	
London	St Bartholomews Hospital (London)	Janet Kiff	
London	St Bartholomews Hospital (London)	Janet Oladimeji	
London	Guy's Hospital (London)	Janette Nichol	
London	Royal Marsden Hospital (London)	Jennifer Morrison	
London	Charing Cross Hospital	Jill Gallagher	
London	St Marys Hospital (London)	Joy Liao	
London	St Marys Hospital (London)	Joy Liau	
London	North Middlesex Hospital	Judy Hill	
London	Royal Free Hospital	Juniebel Cooke	Pharmacist

London	Guy's Hospital (London)	Kate Williams	
London	Royal Marsden Hospital (London)	Laillah-Crystal Banda	
London	Queen Elizabeth Hospital (Woolwich)	Laura Beschizza	
London	St Marys Hospital (London)	Laura Custins	
London	North Middlesex Hospital	Lorraine Hurl	
London	Guy's Hospital (London)	Lucy Reed	
London	Queen Elizabeth Hospital (Woolwich)	Maria Liskova	
London	Royal Free Hospital	Marisa Lanzman	
London	St Marys Hospital (London)	Melloney Allnutt	
London	Guy's Hospital (London)	Michelle Dutton	
London	Charing Cross Hospital	Najma Ahmed	
London	University College Hospital	Noan-Minh Chau	
London	University College Hospital	Patricia Danaswamy	
London	Queen Elizabeth Hospital (Woolwich)	Rachel Harper	
London	Guy's Hospital (London)	Rebecca Todd	
London	Guy's Hospital (London)	Ruth Johnson	
London	Charing Cross Hospital	Samantha Weller	
London	Royal Free Hospital	Sara Fawcitt	
London	Queen Elizabeth Hospital (Woolwich)	Sharai Chitando	
London	Guy's Hospital (London)	Sharon Clovis	
London	St Georges Hospital (London)	Sophie Golden	
London	Guy's Hospital (London)	Srivani Kandasamy	
London	Guy's Hospital (London)	Stephanie Argue	
London	Charing Cross Hospital	Stephanie Steadman	
London	Royal Free Hospital	Su Fung Lo	Pharmacist

London	St Georges Hospital (London)	Sue Cromarty	
London	Royal Marsden Hospital (London)	Suraya Quadir	
London	Guy's Hospital (London)	Suzanne Vizor	
London	Guy's Hospital (London)	Temi Olusi	Pharmacist
London	Guy's Hospital (London)	Ursula Kirwan	
London	University College Hospital	John Masters	
London	Royal Marsden Hospital (London)	Nicholas James	
London	Guy's Hospital (London)	Peter Harper	
London	University College Hospital	Richard Kaplan	Co-I
Maidstone	Maidstone Hospital	Amanda Clarke	
Maidstone	Maidstone Hospital	Claire Baldry	
Maidstone	Maidstone Hospital	Delali Adjogatse	
Maidstone	Maidstone Hospital	Patryk Brulinski	PI
Maidstone	Maidstone Hospital	Alicia Synowiec	
Maidstone	Maidstone Hospital	Clary Evans	
Maidstone	Maidstone Hospital	Emma Kipps	
Maidstone	Maidstone Hospital	Henry Taylor	
Maidstone	Maidstone Hospital	Jess Brady	
Maidstone	Maidstone Hospital	Kathryn Lees	
Maidstone	Maidstone Hospital	Matthew Fittall	
Maidstone	Maidstone Hospital	Romaana Mir	
Maidstone	Maidstone Hospital	Sharon Beesley	
Maidstone	Maidstone Hospital	Amie Thomas	
Maidstone	Maidstone Hospital	Anna English	
Maidstone	Maidstone Hospital	Katy Taylor	

Maidstone	Maidstone Hospital	Gavin Fossey	
Maidstone	Maidstone Hospital	Ian Pamphlett	
Maidstone	Maidstone Hospital	Alison Davison	
Maidstone	Maidstone Hospital	Ann Phillips	
Maidstone	Maidstone Hospital	Jane Murray	
Maidstone	Maidstone Hospital	Louise Hooper-Gilham	
Maidstone	Maidstone Hospital	Alison Richards	
Maidstone	Maidstone Hospital	Carmel Jope	
Maidstone	Maidstone Hospital	Clare Calvert	
Maidstone	Maidstone Hospital	Jane Brown	
Maidstone	Maidstone Hospital	Julia Sunnucks	
Maidstone	Maidstone Hospital	Laura Clayton	
Maidstone	Maidstone Hospital	Verity Roberts	
Maidstone	Maidstone Hospital	Claudia Woodger	
Maidstone	Maidstone Hospital	Emma Craske	
Maidstone	Maidstone Hospital	Joanne Patterson	Pharmacist
Maidstone	Maidstone Hospital	Joanne Williams	
Maidstone	Maidstone Hospital	Lisa Tribe	
Maidstone	Maidstone Hospital	Sarah Martins	
Maidstone	Maidstone Hospital	Su Burrage	
Maidstone	Maidstone Hospital	Vivienne Breen	
Manchester	Christie Hospital	Andrew Hudson	
Manchester	Christie Hospital	Ather Kazmi	
Manchester	Christie Hospital	Christoph Oing	Co-I
Manchester	Christie Hospital	Hebalalla Abdelaal	

Manchester	Christie Hospital	Martin Swinton	
Manchester	Christie Hospital	Robin Portner	
Manchester	Christie Hospital	Sreeja Aruketty	
Manchester	Christie Hospital	Stefanie Fisder	
Manchester	Christie Hospital	Stephen Chin	
Manchester	Christie Hospital	Yee Pei Song	
Manchester	Christie Hospital	A Jegannathen	
Manchester	Christie Hospital	Ananya Choudhury	
Manchester	Christie Hospital	Anna Bruzzan	
Manchester	Christie Hospital	Anna Tran	
Manchester	Withington Hospital	Beatriz Duran Jimenez	Pharmacist
Manchester	Wythenshawe Hospital	Beatriz Duran Jimenez	
Manchester	Christie Hospital	Catherine Coyle	
Manchester	Christie Hospital	Clara Chan	
Manchester	Christie Hospital	David Thompson	
Manchester	Christie Hospital	Jacqueline Livsey	
Manchester	Christie Hospital	James Wylie	
Manchester	Withington Hospital	James Wylie	
Manchester	Christie Hospital	John Logue	
Manchester	Christie Hospital	Michael Braun	
Manchester	Christie Hospital	Richard Cowan	
Manchester	Christie Hospital	Ruth Conroy	Co-I
Manchester	Christie Hospital	Shaun Tolan	
Manchester	Christie Hospital	Silke Gillessen	
Manchester	Christie Hospital	Tony Elliott	

Manchester	Christie Hospital	You Yone	
Manchester	Christie Hospital	Amber Hart	
Manchester	Withington Hospital	Anna Gipson	
Manchester	Christie Hospital	Charlotte Heywood	
Manchester	Christie Hospital	Kim Fair	
Manchester	Christie Hospital	Laura Flanagan	
Manchester	Christie Hospital	Maria Petsa	
Manchester	Christie Hospital	Samah Mughal	
Manchester	Christie Hospital	Sarah Green	
Manchester	Christie Hospital	Sarah-Ellen Ellen (née McCarthy)	
Manchester	Withington Hospital	Tania Cutts	
Manchester	Christie Hospital	Willemijn Spoor	
Manchester	Christie Hospital	Alkesh Patel	
Manchester	Christie Hospital	Kamlesh Patel	
Manchester	Christie Hospital	Tony Elliott	
Manchester	Withington Hospital	A. Emara	
Manchester	Wythenshawe Hospital	A. Emara	
Manchester	Christie Hospital	Ali Al-Hashimi	Pharmacist
Manchester	Christie Hospital	Damian McCall	
Manchester	Withington Hospital	Damian McCall	
Manchester	Christie Hospital	Damian McCaul	
Manchester	Christie Hospital	Ekugbe Onogbe	
Manchester	Christie Hospital	Ekugbe Onoge	
Manchester	Christie Hospital	Ian Bottomley	
Manchester	Christie Hospital	Vijay Ramani	

Manchester	Withington Hospital	Vijay Ramani	Co-I
Manchester	Christie Hospital	Vijay Sangar	
Manchester	Withington Hospital	Vijay Sangar	PI
Manchester	Wythenshawe Hospital	Vijay Sangar	PI
Manchester	Withington Hospital	Helen Haydock	
Manchester	Withington Hospital	Vivienne Benson	
Manchester	Wythenshawe Hospital	Vivienne Benson	
Manchester	Christie Hospital	Kate O'Connor	
Manchester	Christie Hospital	Sue Davison	
Manchester	Withington Hospital	Annie Duffy	
Manchester	Wythenshawe Hospital	Annie Duffy	
Manchester	Christie Hospital	Catherine Pettersen	
Manchester	Wythenshawe Hospital	Claire McGuire	
Manchester	Withington Hospital	Humera Ahmed	
Manchester	Wythenshawe Hospital	Julie Fielding	
Manchester	Withington Hospital	Kathryn Fellows	
Manchester	Wythenshawe Hospital	Kathryn Slevin	
Manchester	Wythenshawe Hospital	Kirsty Melia	
Manchester	Withington Hospital	Linda Bailey	
Manchester	Wythenshawe Hospital	Linda Bailey	
Manchester	Christie Hospital	Lucy Worsley	
Manchester	Christie Hospital	Megan Bunce	
Manchester	Wythenshawe Hospital	Molly Bennett	
Manchester	Withington Hospital	Rebecca Corless	
Manchester	Christie Hospital	Roonak Nazari	

Manchester	Christie Hospital	salina tsui	
Manchester	Wythenshawe Hospital	Sarah Liptrott	
Manchester	Christie Hospital	Sharon Capper	
Manchester	Withington Hospital	Stephanie Hargreaves	
Manchester	Christie Hospital	Thiraviyam Elumalai	
Manchester	Christie Hospital	Trishna Uttamlal	
Manchester	Wythenshawe Hospital	Wendy Guest	
Manchester	Christie Hospital	Zhara Mahmood	
Manchester	Wythenshawe Hospital	Angela Chrisopoulou	
Manchester	Wythenshawe Hospital	Angela Gowrie	
Manchester	Christie Hospital	Anna Bowron	
Manchester	Christie Hospital	Catherine Redshaw	
Manchester	Withington Hospital	Catherine Redshaw	
Manchester	Christie Hospital	Cathryn James	
Manchester	Christie Hospital	Cathryn Jones	
Manchester	Christie Hospital	Emma Burke	
Manchester	Christie Hospital	Emma Lowther	
Manchester	Withington Hospital	Fiona Murtagh	
Manchester	Withington Hospital	Janet Smith	
Manchester	Christie Hospital	Jeanette Lyons	
Manchester	Christie Hospital	Joanne Oliver	
Manchester	Withington Hospital	Julie Bramley	
Manchester	Withington Hospital	Karen Robb	
Manchester	Withington Hospital	Lillian Partington	Pharmacist
Manchester	Withington Hospital	Lindsay Piper	

Manchester	Wythenshawe Hospital	Lindsay Piper	
Manchester	Withington Hospital	Lorraine Turner	
Manchester	Christie Hospital	Lydia Sutherland	Pharmacist
Manchester	Christie Hospital	Lynne Gilmore	
Manchester	Christie Hospital	Marie Woolley	
Manchester	Christie Hospital	Sarah-Ellen Smith	
Manchester	Christie Hospital	Sue Seifi	
Manchester	Christie Hospital	Susan Arrand	
Manchester	Withington Hospital	Tarnya Hulme	
Manchester	Withington Hospital	Thobekile Mthethwa	
Manchester	Wythenshawe Hospital	Thobekile Mthethwa	
Manchester	Withington Hospital	Tracey Platt	
Manchester	Wythenshawe Hospital	Tracey Platt	
Manchester	Christie Hospital	Noel Clarke	PI
Manchester	Christie Hospital	Jackie O'Dwyer	
Manchester	Christie Hospital	Viv Thomas	
Margate	Queen Elizabeth The Queen Mother Hospital	Albert Edwards	Co-I
Margate	Queen Elizabeth The Queen Mother Hospital	Charlotte Mott	
Margate	Queen Elizabeth The Queen Mother Hospital	Ifigenia Vasiliadou	Co-I
Margate	Queen Elizabeth The Queen Mother Hospital	Lavarniya Rajakumar	
Margate	Queen Elizabeth The Queen Mother Hospital	Mathini Sridharan	
Margate	Queen Elizabeth The Queen Mother Hospital	Patryk Brulinski	
Margate	Queen Elizabeth The Queen Mother Hospital	Rakesh Raman	Co-I
Margate	Queen Elizabeth The Queen Mother Hospital	Rohit Malde	
Margate	Queen Elizabeth The Queen Mother Hospital	Stephane Tankoua	

Margate	Queen Elizabeth The Queen Mother Hospital	Arafat Mizra	
Margate	Queen Elizabeth The Queen Mother Hospital	Carys Thomas	PI
Margate	Queen Elizabeth The Queen Mother Hospital	Clary Evans	
Margate	Queen Elizabeth The Queen Mother Hospital	Kannon Nathan	
Margate	Queen Elizabeth The Queen Mother Hospital	Kathryn Lees	
Margate	Queen Elizabeth The Queen Mother Hospital	Mathilda Cominos	
Margate	Queen Elizabeth The Queen Mother Hospital	Matthew Fenton	
Margate	Queen Elizabeth The Queen Mother Hospital	Mohammed Osman	
Margate	Queen Elizabeth The Queen Mother Hospital	Natasha Mithal	Co-I
Margate	Queen Elizabeth The Queen Mother Hospital	Sharon Beesley	
Margate	Queen Elizabeth The Queen Mother Hospital	Sugeeta Sukumar	
Margate	Queen Elizabeth The Queen Mother Hospital	Udaiveer Panwar	
Margate	Queen Elizabeth The Queen Mother Hospital	Coral Greenstreet	
Margate	Queen Elizabeth The Queen Mother Hospital	Hayley Blackgrove	
Margate	Queen Elizabeth The Queen Mother Hospital	Katy Taylor	
Margate	Queen Elizabeth The Queen Mother Hospital	Victoria Williamson	
Margate	Queen Elizabeth The Queen Mother Hospital	Natalie Catt	
Margate	Queen Elizabeth The Queen Mother Hospital	Arafat Mirza	
Margate	Queen Elizabeth The Queen Mother Hospital	Sam Gibson	
Margate	Queen Elizabeth The Queen Mother Hospital	Steve Dann	
Margate	Queen Elizabeth The Queen Mother Hospital	Andrew Gillian	Pharmacist
Margate	Queen Elizabeth The Queen Mother Hospital	Miguel Capo-Mir	Pharmacist
Margate	Queen Elizabeth The Queen Mother Hospital	Cindy Slater	
Margate	Queen Elizabeth The Queen Mother Hospital	Hasmath Marjolin	
Margate	Queen Elizabeth The Queen Mother Hospital	Nikki Crisp	

Margate	Queen Elizabeth The Queen Mother Hospital	Rachel Larkins	
Margate	Queen Elizabeth The Queen Mother Hospital	Sandra Holness	
Margate	Queen Elizabeth The Queen Mother Hospital	Sarah Lines	
Margate	Queen Elizabeth The Queen Mother Hospital	Susan Rogers	
Margate	Queen Elizabeth The Queen Mother Hospital	Claire White	
Margate	Queen Elizabeth The Queen Mother Hospital	Julie Buckley	
Margate	Queen Elizabeth The Queen Mother Hospital	Laura Kehoe	
Margate	Queen Elizabeth The Queen Mother Hospital	Lesley Rose	
Margate	Queen Elizabeth The Queen Mother Hospital	Louise Gladwell	
Margate	Queen Elizabeth The Queen Mother Hospital	Sarah Lightfoot	Pharmacist
Margate	Queen Elizabeth The Queen Mother Hospital	Tracy Boakes	
Margate	Queen Elizabeth The Queen Mother Hospital	Alba Tubau	
Margate	Queen Elizabeth The Queen Mother Hospital	Bonny Appleby	
Margate	Queen Elizabeth The Queen Mother Hospital	Jo Williams	
Margate	Queen Elizabeth The Queen Mother Hospital	Linda Wray	Pharmacist
Margate	Queen Elizabeth The Queen Mother Hospital	Louise Allen	
Margate	Queen Elizabeth The Queen Mother Hospital	Marian Wood	
Margate	Queen Elizabeth The Queen Mother Hospital	Adedolapo Sanni	
Margate	Queen Elizabeth The Queen Mother Hospital	Claire Pelham	
Margate	Queen Elizabeth The Queen Mother Hospital	Elizabeth Williamson	
Margate	Queen Elizabeth The Queen Mother Hospital	Hilary Zurakovsky	
Margate	Queen Elizabeth The Queen Mother Hospital	Jill Baker	
Margate	Queen Elizabeth The Queen Mother Hospital	Joanne Williams	
Margate	Queen Elizabeth The Queen Mother Hospital	Julie-Ann Davies	
Margate	Queen Elizabeth The Queen Mother Hospital	Karen Robinson	

Margate	Queen Elizabeth The Queen Mother Hospital	Kathleen (Kathy) Walsh	
Margate	Queen Elizabeth The Queen Mother Hospital	Kim Mears	
Margate	Queen Elizabeth The Queen Mother Hospital	Kim Travis	
Margate	Queen Elizabeth The Queen Mother Hospital	Margaret Lipsham	
Margate	Queen Elizabeth The Queen Mother Hospital	Paula Whichelo	
Margate	Queen Elizabeth The Queen Mother Hospital	Sharon Middleton	
Margate	Queen Elizabeth The Queen Mother Hospital	Sue Kelly	
Margate	Queen Elizabeth The Queen Mother Hospital	Susan Drakeley	
Margate	Queen Elizabeth The Queen Mother Hospital	Sydnie Loveland	
Margate	Queen Elizabeth The Queen Mother Hospital	Molua Young	
Margate	Queen Elizabeth The Queen Mother Hospital	Denise Crawford	
Merseyside	Southport and Formby District General Hospital	Julie Griffiths	
Middlesborough	James Cook University Hospital	Julia McBride	
Middlesborough	James Cook University Hospital	Fiona Rowling	Pharmacist
Middlesbrough	James Cook University Hospital	Cheng Lee Chaw	
Middlesbrough	James Cook University Hospital	Maha Zarroug	
Middlesbrough	James Cook University Hospital	Mohammed Kagzi	
Middlesbrough	James Cook University Hospital	Clive Peedell	PI
Middlesbrough	James Cook University Hospital	David Wilson	
Middlesbrough	James Cook University Hospital	Devadasan Shakespeare	
Middlesbrough	James Cook University Hospital	Hans Van der Voet	
Middlesbrough	James Cook University Hospital	Jason Wong	
Middlesbrough	James Cook University Hospital	John Hardman	
Middlesbrough	James Cook University Hospital	Steven Pratt	
Middlesbrough	James Cook University Hospital	Anne Hardwick	Pharmacist

Middlesbrough	James Cook University Hospital	Emma Pringleton
Middlesbrough	James Cook University Hospital	Jo Atkinson
Middlesbrough	James Cook University Hospital	Joanne Atkinson
Middlesbrough	James Cook University Hospital	Kate Rees
Middlesbrough	James Cook University Hospital	Michaela Davenport
Middlesbrough	James Cook University Hospital	Michaela Devenport
Middlesbrough	James Cook University Hospital	Rebecca Richards
Middlesbrough	James Cook University Hospital	Andrew Vaux
Middlesbrough	James Cook University Hospital	Craig Mower
Middlesbrough	James Cook University Hospital	Luca Settimo
Middlesbrough	James Cook University Hospital	Piers Loxley Winder
Middlesbrough	James Cook University Hospital	Piers Winders
Middlesbrough	James Cook University Hospital	David Chadwick
Middlesbrough	James Cook University Hospital	Keith Harland
Middlesbrough	James Cook University Hospital	Paul Jones-King
Middlesbrough	James Cook University Hospital	Alison Barnes
Middlesbrough	James Cook University Hospital	Claire Elliott
Middlesbrough	James Cook University Hospital	Emanuela Mahmoud
Middlesbrough	James Cook University Hospital	Alison Chilvers
Middlesbrough	James Cook University Hospital	Carol Long
Middlesbrough	James Cook University Hospital	Helen Carver
Middlesbrough	James Cook University Hospital	Jane Thompson
Middlesbrough	James Cook University Hospital	Julie Potts
Middlesbrough	James Cook University Hospital	Lisa Peacock (nee Wayman)
Middlesbrough	James Cook University Hospital	Paula Milne

Middlesbrough	James Cook University Hospital	Emma Thompson	
Middlesbrough	James Cook University Hospital	Helen Dunn	Pharmacist
Middlesbrough	James Cook University Hospital	Katherine Tyler	
Middlesbrough	James Cook University Hospital	Lynne Naylor	
Middlesbrough	James Cook University Hospital	Sarah Kiddell	
Middlesbrough	James Cook University Hospital	Sarah McAuliffe	
Middlesbrough	James Cook University Hospital	Agnieszka Skotnicka	Pharmacist
Middlesbrough	James Cook University Hospital	Andrea Watson	
Middlesbrough	James Cook University Hospital	Charlotte Jacobs(née Kitching)	
Middlesbrough	James Cook University Hospital	Rita Mohan	
Middlesbrough	James Cook University Hospital	Vicky Hanlon	Pharmacist
Newcastle upon Tyne	Newcastle General Hospital	Judith Moore	
Newcastle-upon-Tyne	Freeman Hospital	Alex Mitchell	
Newcastle-upon-Tyne	Freeman Hospital	Elle Cameron	
Newcastle-upon-Tyne	Freeman Hospital	John Frew	Co-I
Newcastle-upon-Tyne	Freeman Hospital	Nicola Hannaway	
Newcastle-upon-Tyne	Freeman Hospital	Noor Harris	
Newcastle-upon-Tyne	Freeman Hospital	Robert Chandler	
Newcastle-upon-Tyne	Freeman Hospital	Shahid Iqbal	Co-I
Newcastle-upon-Tyne	Freeman Hospital	Ashraf Azzabi	PI
Newcastle-upon-Tyne	Freeman Hospital	Ian Pedley	Co-I
Newcastle-upon-Tyne	Freeman Hospital	Rhona McMenemin	Co-I
Newcastle-upon-Tyne	Freeman Hospital	Amanda Henderson	
Newcastle-upon-Tyne	Freeman Hospital	Catherine Marsh	
Newcastle-upon-Tyne	Freeman Hospital	Emma King	

Newcastle-upon-Tyne	Freeman Hospital	Gemma O'Neill	
Newcastle-upon-Tyne	Freeman Hospital	Georgia Ross	
Newcastle-upon-Tyne	Freeman Hospital	Hannah Downs	
Newcastle-upon-Tyne	Freeman Hospital	Janine Tate	
Newcastle-upon-Tyne	Freeman Hospital	Katie Bain	
Newcastle-upon-Tyne	Freeman Hospital	Lucy Blackwell	
Newcastle-upon-Tyne	Freeman Hospital	Marianne Smith	
Newcastle-upon-Tyne	Freeman Hospital	Victoria Thomas	
Newcastle-upon-Tyne	Freeman Hospital	Andrew Herridge	
Newcastle-upon-Tyne	Freeman Hospital	Craig Alderson	Pharmacist
Newcastle-upon-Tyne	Freeman Hospital	Edgar Paez	
Newcastle-upon-Tyne	Freeman Hospital	Gerard Oakes	
Newcastle-upon-Tyne	Freeman Hospital	Ian Campbell	Pharmacist
Newcastle-upon-Tyne	Freeman Hospital	Roger Carr	
Newcastle-upon-Tyne	Freeman Hospital	Thomas Jarvis	
Newcastle-upon-Tyne	Freeman Hospital	Ben Hood	
Newcastle-upon-Tyne	Freeman Hospital	Mark Johnson	
Newcastle-upon-Tyne	Freeman Hospital	Naeem Soomro	
Newcastle-upon-Tyne	Freeman Hospital	Peter Murphy	
Newcastle-upon-Tyne	Freeman Hospital	Dianne Turner	
Newcastle-upon-Tyne	Freeman Hospital	Elizabeth Reay	
Newcastle-upon-Tyne	Freeman Hospital	Kristine Hawkins	
Newcastle-upon-Tyne	Freeman Hospital	Ruth Latter	
Newcastle-upon-Tyne	Freeman Hospital	Sarah Osborne	
Newcastle-upon-Tyne	Freeman Hospital	Sarah Rowling	

Newcastle-upon-Tyne	Freeman Hospital	Sarah Wright	
Newcastle-upon-Tyne	Freeman Hospital	Nichola Waugh	
Newcastle-upon-Tyne	Freeman Hospital	Ann Hudson	
Newcastle-upon-Tyne	Freeman Hospital	Caroline Dobeson	
Newcastle-upon-Tyne	Freeman Hospital	Diane Conner	
Newcastle-upon-Tyne	Freeman Hospital	Diane Connor	
Newcastle-upon-Tyne	Freeman Hospital	Dianne Wake	
Newcastle-upon-Tyne	Freeman Hospital	Elaine Greaves	
Newcastle-upon-Tyne	Freeman Hospital	Hazel Forsyth	
Newcastle-upon-Tyne	Freeman Hospital	Hazel Masson	
Newcastle-upon-Tyne	Freeman Hospital	Irene Jobson	
Newcastle-upon-Tyne	Freeman Hospital	Jenny Smith	
Newcastle-upon-Tyne	Freeman Hospital	Julie Thohig	
Newcastle-upon-Tyne	Freeman Hospital	Laura Jameson	
Newcastle-upon-Tyne	Freeman Hospital	Lauren Boal	
Newcastle-upon-Tyne	Freeman Hospital	Lavanya Mariappan	
Newcastle-upon-Tyne	Freeman Hospital	Penny Bradley	
Newcastle-upon-Tyne	Freeman Hospital	Ruth Sinnott	
Newcastle-upon-Tyne	Freeman Hospital	Sue Farrell	
Newcastle-upon-Tyne	Freeman Hospital	Sunita Kholi	
Newcastle-upon-Tyne	Freeman Hospital	Sunita Kollu	
Newcastle-upon-Tyne	Freeman Hospital	Xue Jiang	
Newcastle-upon-Tyne	Freeman Hospital	Carole Stobbart	
Newcastle-upon-Tyne	Freeman Hospital	Kay Carson	Pharmacist
Newcastle-upon-Tyne	Freeman Hospital	Lesley Naik	

Newport	St Mary's Hospital (Newport)	Kudingila Madhava	
Newport	St Mary's Hospital (Newport)	Alison Brown	
Newport	St Mary's Hospital (Newport)	Cindy Whitbread	
Newport	St Mary's Hospital (Newport)	Tracey Tidbury	
North Shields	North Tyneside General Hospital	Mark Johnson	
Northampton	Northampton General Hospital	Rachel Gabitass	
Northwood	Mount Vernon Hospital	Neel Bhuva	
Northwood	Mount Vernon Hospital	Adam Mitchell	Co-I
Northwood	Mount Vernon Hospital	Charlotte Westbury	
Northwood	Mount Vernon Hospital	David Woolf	
Northwood	Mount Vernon Hospital	Hamoun Rozati	Co-I
Northwood	Mount Vernon Hospital	Hannah Tharmalingam	
Northwood	Mount Vernon Hospital	Janaka Cooray	
Northwood	Mount Vernon Hospital	Joanne Kosmin	
Northwood	Mount Vernon Hospital	Lai Cheng Yew	
Northwood	Mount Vernon Hospital	Mohammed Abdul-Latif	
Northwood	Mount Vernon Hospital	Russell Moule	
Northwood	Mount Vernon Hospital	Sara Kashani	Co-I
Northwood	Mount Vernon Hospital	Claire Zane	
Northwood	Mount Vernon Hospital	Dolan Basak	
Northwood	Mount Vernon Hospital	Henry Mandeville	
Northwood	Mount Vernon Hospital	Huiqi Yang	
Northwood	Mount Vernon Hospital	Jeanette Dickson	
Northwood	Mount Vernon Hospital	Jennifer Chard	
Northwood	Mount Vernon Hospital	Kasia Owczarczyk	

Northwood	Mount Vernon Hospital	Katie Wood	
Northwood	Mount Vernon Hospital	Kent Yip	
Northwood	Mount Vernon Hospital	Lai-Cheng Yew	
Northwood	Mount Vernon Hospital	Linda Swaney	
Northwood	Mount Vernon Hospital	M Williams	
Northwood	Mount Vernon Hospital	Mausam Singhera	
Northwood	Mount Vernon Hospital	Nicola Anyamene	
Northwood	Mount Vernon Hospital	Olivia Hatcher	
Northwood	Mount Vernon Hospital	Paolo De Jesu	
Northwood	Mount Vernon Hospital	Peter Ostler	
Northwood	Mount Vernon Hospital	Rachael Khong	
Northwood	Mount Vernon Hospital	Robert Hughes	
Northwood	Mount Vernon Hospital	Roberto Alonzi	
Northwood	Mount Vernon Hospital	Shaista Harpeer	
Northwood	Mount Vernon Hospital	Shiv Gayadeen	
Northwood	Mount Vernon Hospital	Sonia Li	
Northwood	Mount Vernon Hospital	Viwod Mullassery	PI
Northwood	Mount Vernon Hospital	Aamna Rashid	
Northwood	Mount Vernon Hospital	Freya Ball	
Northwood	Mount Vernon Hospital	Harmeet Sangha	
Northwood	Mount Vernon Hospital	Juhee Kong	
Northwood	Mount Vernon Hospital	Lucy Collins	
Northwood	Mount Vernon Hospital	Rose Bell	
Northwood	Mount Vernon Hospital	Tahmina Shakil	
Northwood	Mount Vernon Hospital	Alice Ramsden	

Northwood	Mount Vernon Hospital	David Tan	
Northwood	Mount Vernon Hospital	Farhan Ahmed	Pharmacist
Northwood	Mount Vernon Hospital	Sam Bosompem	Pharmacist
Northwood	Mount Vernon Hospital	Heidi Rana	
Northwood	Mount Vernon Hospital	Jessica Finch	
Northwood	Mount Vernon Hospital	Kari Evans	
Northwood	Mount Vernon Hospital	Nalini Shah	
Northwood	Mount Vernon Hospital	Rakhi Jain	Pharmacist
Northwood	Mount Vernon Hospital	Shakeda Lakha	
Northwood	Mount Vernon Hospital	Bhanthi Kanagaratnam	
Northwood	Mount Vernon Hospital	Hannah Phillips	
Northwood	Mount Vernon Hospital	Harsha Vara	Pharmacist
Northwood	Mount Vernon Hospital	Helen Cladd	
Northwood	Mount Vernon Hospital	Justina Kailey	
Northwood	Mount Vernon Hospital	Lesley Mitchell	
Northwood	Mount Vernon Hospital	Nazma Damani	
Northwood	Mount Vernon Hospital	Nicola Cutmore	
Northwood	Mount Vernon Hospital	Paulina Kowalewska	
Northwood	Mount Vernon Hospital	Sapna Kaur	
Northwood	Mount Vernon Hospital	Elaine Lousley	
Northwood	Mount Vernon Hospital	Hameeda Sultany	Pharmacist
Northwood	Mount Vernon Hospital	Jessica Milner	
Northwood	Mount Vernon Hospital	Sara Abbassi	
Northwood	Mount Vernon Hospital	Suzanne Jenkins	
Northwood	Mount Vernon Hospital	Peter Hoskin	PI

Nottingham	Nottingham University Hospitals, City Campus	Adam Fullagar	
Nottingham	Nottingham University Hospitals, City Campus	Charlotte Kamlow	
Nottingham	Nottingham University Hospitals, City Campus	Chin Chong	
Nottingham	Nottingham University Hospitals, City Campus	Ewan Shawcroft	
Nottingham	Nottingham University Hospitals, City Campus	Junhao Lim	Co-I
Nottingham	Nottingham University Hospitals, City Campus	Lauren Jones	
Nottingham	Nottingham University Hospitals, City Campus	Maeve Pomeroy	
Nottingham	Nottingham University Hospitals, City Campus	Melanie Boafo-Yirenkyi	
Nottingham	Nottingham University Hospitals, City Campus	Rebekah Webb	
Nottingham	Nottingham University Hospitals, City Campus	Rohan Tharaka	Co-I
Nottingham	Nottingham University Hospitals, City Campus	Rohan Tharakan	
Nottingham	Nottingham University Hospitals, City Campus	Sadia Abdullah	Co-I
Nottingham	Nottingham University Hospitals, City Campus	Sarah Taylor	
Nottingham	Nottingham University Hospitals, City Campus	Shiv Uppal	
Nottingham	Nottingham University Hospitals, City Campus	Thomas Moore	
Nottingham	Nottingham University Hospitals, City Campus	Wai Hou Sam	
Nottingham	Nottingham University Hospitals, City Campus	Ananth Sivanandan	
Nottingham	Nottingham University Hospitals, City Campus	Charlotte Ellis	
Nottingham	Nottingham University Hospitals, City Campus	Daniel Saunders	
Nottingham	Nottingham University Hospitals, City Campus	Eliot Chadwick	Co-I
Nottingham	Nottingham University Hospitals, City Campus	Georgina Walker	Co-I
Nottingham	Nottingham University Hospitals, City Campus	Ian Sayers	
Nottingham	Nottingham University Hospitals, City Campus	Jamie Mills	
Nottingham	Nottingham University Hospitals, City Campus	Louise Brookes	
Nottingham	Nottingham University Hospitals, City Campus	Santhanam Sundar	PI

Nottingham	Nottingham University Hospitals, City Campus	Adele Malson	
Nottingham	Nottingham University Hospitals, City Campus	Asmaa Sa Omer	
Nottingham	Nottingham University Hospitals, City Campus	Camille Hutchinson	
Nottingham	Nottingham University Hospitals, City Campus	Hannah Thurlow	
Nottingham	Nottingham University Hospitals, City Campus	Jade Eggleton	
Nottingham	Nottingham University Hospitals, City Campus	Kayleigh Mills	
Nottingham	Nottingham University Hospitals, City Campus	Lucy Howard	
Nottingham	Nottingham University Hospitals, City Campus	Leanne Alder	
Nottingham	Nottingham University Hospitals, City Campus	Alex Blades	
Nottingham	Nottingham University Hospitals, City Campus	Jacob Szolin-Jones	
Nottingham	Nottingham University Hospitals, City Campus	Daniel Kumar	
Nottingham	Nottingham University Hospitals, City Campus	Matthew Brazkiewicz	
Nottingham	Nottingham University Hospitals, City Campus	Owen Cole	
Nottingham	Nottingham University Hospitals, City Campus	Tin Sang-Tsang	Pharmacist
Nottingham	Nottingham University Hospitals, City Campus	Anita Stevenson	Pharmacist
Nottingham	Nottingham University Hospitals, City Campus	Katie Carter	
Nottingham	Nottingham University Hospitals, City Campus	Phillipa Sum	
Nottingham	Nottingham University Hospitals, City Campus	Samantha Chetiyawardana	
Nottingham	Nottingham University Hospitals, City Campus	Tania Slater	
Nottingham	Nottingham University Hospitals, City Campus	Carol Gooch	
Nottingham	Nottingham University Hospitals, City Campus	Kathryn Moore	
Nottingham	Nottingham University Hospitals, City Campus	Susan Elliott	
Nottingham	Nottingham University Hospitals, City Campus	Cody Jevons	
Nottingham	Nottingham University Hospitals, City Campus	Rachael Chivers	
Nottingham	Nottingham University Hospitals, City Campus	Sarah Widdowson	

Nottingham	Nottingham University Hospitals, City Campus	Stephanie McGonagle	
Nottingham	Nottingham University Hospitals, City Campus	Caitlin Todd	
Nottingham	Nottingham University Hospitals, City Campus	Rena Chauhan	Pharmacist
Nottingham	Nottingham University Hospitals, City Campus	Stacey Green	
Nuneaton	George Eliot Hospital	Andrew White	
Nuneaton	George Eliot Hospital	Ellanna Guithi	
Nuneaton	George Eliot Hospital	Michael Tilby	Co-I
Nuneaton	George Eliot Hospital	Andrew Chan	
Nuneaton	George Eliot Hospital	Yakhub Khan	PI
Nuneaton	George Eliot Hospital	Damilola Jayeoba	Pharmacist
Nuneaton	George Eliot Hospital	Holly Lawrence	
Nuneaton	George Eliot Hospital	Kerry Flahive	
Nuneaton	George Eliot Hospital	sara Taylor	
Nuneaton	George Eliot Hospital	Arandeep Hayer	
Nuneaton	George Eliot Hospital	Albert Mislang	
Nuneaton	George Eliot Hospital	Jacob Bourne	
Nuneaton	George Eliot Hospital	Andrea Mills	
Nuneaton	George Eliot Hospital	Inderjit Atwal	
Nuneaton	George Eliot Hospital	Jenna Williams	
Nuneaton	George Eliot Hospital	Alison McCallum	
Nuneaton	George Eliot Hospital	Jessica Gunn	
Nuneaton	George Eliot Hospital	Karen Shorthose	
Nuneaton	George Eliot Hospital	Michaela Hill	
Nuneaton	George Eliot Hospital	Pritpal Klear	
Nuneaton	George Eliot Hospital	Rachael Oates	

Nuneaton	George Eliot Hospital	Rachel Fergusson	
Nuneaton	George Eliot Hospital	Sarah Fergusson	
Nuneaton	George Eliot Hospital	Winni Singh	
Nuneaton	George Eliot Hospital	Jeanette Knapp	
Nuneaton	George Eliot Hospital	Melanie Taylor	Pharmacist
Nuneaton	George Eliot Hospital	Sabiya Nasima	
Nuneaton	George Eliot Hospital	Judith Lake	
Oldham	Royal Oldham Hospital	Agata Rembielak	
Oldham	Royal Oldham Hospital	Anthea Cree	
Oldham	Royal Oldham Hospital	Ehab Ibrahim	Co-I
Oldham	Royal Oldham Hospital	Helen Joyce	
Oldham	Royal Oldham Hospital	Hwoeifen Soohoo	
Oldham	Royal Oldham Hospital	Kanal Gupta	
Oldham	Royal Oldham Hospital	Mohammad Abutarb	
Oldham	Royal Oldham Hospital	Parth Desai	Co-I
Oldham	Royal Oldham Hospital	Richard Walshaw	
Oldham	Royal Oldham Hospital	Shabaz Hussain	
Oldham	Royal Oldham Hospital	Shazril Imran Shaukat	
Oldham	Royal Oldham Hospital	Stephen Kennedy	
Oldham	Royal Oldham Hospital	Victoria Lavin	
Oldham	Royal Oldham Hospital	Ananya Choudhury	Co-I
Oldham	Royal Oldham Hospital	Anna Tran	Co-I
Oldham	Royal Oldham Hospital	Jacqueline Livsey	
Oldham	Royal Oldham Hospital	Peter Mbanu	
Oldham	Royal Oldham Hospital	Ruth Conroy	PI

Oldham	Royal Oldham Hospital	Shaveta Mehta	Co-I
Oldham	Royal Oldham Hospital	Dellesa Robinson	
Oldham	Royal Oldham Hospital	Sophie Hovenden	
Oldham	Royal Oldham Hospital	Mark Livingstone	Pharmacist
Oldham	Royal Oldham Hospital	Farhan Karim	
Oldham	Royal Oldham Hospital	Richard Jones	
Oldham	Royal Oldham Hospital	Terence Hinton	
Oldham	Royal Oldham Hospital	Sarah Warran	
Oldham	Royal Oldham Hospital	Udeme Ohia	Pharmacist
Oldham	Royal Oldham Hospital	Leena Mistry	
Oldham	Royal Oldham Hospital	Amy Slack	
Oldham	Royal Oldham Hospital	Anna Pracz	Pharmacist
Oldham	Royal Oldham Hospital	Dawn Johnstone	
Oldham	Royal Oldham Hospital	Joanne Allsop	
Oldham	Royal Oldham Hospital	Joanne Johnson	
Oldham	Royal Oldham Hospital	Joanne Reed	
Oldham	Royal Oldham Hospital	Kamala Ramatar	
Oldham	Royal Oldham Hospital	Kirstie Smith	
Oldham	Royal Oldham Hospital	Lyndsay Scarratt	
Oldham	Royal Oldham Hospital	Ruth Halford	
Oldham	Royal Oldham Hospital	Suzanne Bland	
Oldham	Royal Oldham Hospital	Wendy Cook	
Oldham	Royal Oldham Hospital	Hadia Ashraf	
Oldham	Royal Oldham Hospital	Jemma McLaughlin	Pharmacist
Oxford	Churchill Hospital	Ami Sabharwal	Co-I

Oxford	Churchill Hospital	Avinash Gupta	Co-I
Oxford	Churchill Hospital	Katherine Hyde	Co-I
Oxford	Churchill Hospital	Laura Robledo	
Oxford	Churchill Hospital	Silke Hahnewald	
Oxford	Churchill Hospital	Simon Wyatt	
Oxford	Churchill Hospital	Tessa Greenhalgh	
Oxford	Churchill Hospital	Thinn Pwint	
Oxford	Churchill Hospital	Benjamin Fairfax	Co-I
Oxford	Churchill Hospital	David J Cole	Co-I
Oxford	Churchill Hospital	Elaine Sugden	
Oxford	Churchill Hospital	Gerard Andrade	Co-I
Oxford	Churchill Hospital	Mark Prentice	
Oxford	Churchill Hospital	Meenali Chitnis	Co-I
Oxford	Churchill Hospital	Paul Colin Miller	
Oxford	Churchill Hospital	Philip Camilleri	Co-I
Oxford	Churchill Hospital	Robert Stuart	Co-I
Oxford	Churchill Hospital	Robert Watson	
Oxford	Churchill Hospital	Elizabeth Hadley	
Oxford	Churchill Hospital	Kelly Wigglesworth	
Oxford	Churchill Hospital	Lauren Booker	
Oxford	Churchill Hospital	Patrycja Jastrzebska	
Oxford	Churchill Hospital	Sophia Shahzad	
Oxford	Churchill Hospital	Swapna Thummala	
Oxford	Churchill Hospital	Sywlia Bekulart	
Oxford	Churchill Hospital	Daniel Ajzensztejn	Co-I

Oxford	Churchill Hospital	Henry Chesson	
Oxford	Churchill Hospital	Hugo De La Pena	
Oxford	Churchill Hospital	Mark Tuthill	
Oxford	Churchill Hospital	Naveen Sankighatta	
Oxford	Churchill Hospital	Richard Cousins	
Oxford	Churchill Hospital	Will Goodman	
Oxford	Churchill Hospital	Matthew Mooney	
Oxford	Churchill Hospital	Martha Woodward	
Oxford	Churchill Hospital	Rachel Hart	
Oxford	Churchill Hospital	Rosita Broderick	
Oxford	Churchill Hospital	Ann Murphy	
Oxford	Churchill Hospital	Charlotte Davies	
Oxford	Churchill Hospital	Jo Wilson	
Oxford	Churchill Hospital	Kerrie Marston	
Oxford	Churchill Hospital	Julie Pinder	
Oxford	Churchill Hospital	Katherine Jacob	Pharmacist
Oxford	Churchill Hospital	Magdalena Benysek	
Oxford	Churchill Hospital	Sandra Mukkath	
Oxford	Churchill Hospital	Sarah Markus	
Oxford	Churchill Hospital	Ana De Veciana	Pharmacist
Oxford	Churchill Hospital	Anju Chalin	
Oxford	Churchill Hospital	Evanthia Komninidou	
Oxford	Churchill Hospital	Gabriela Kuzmycha	
Oxford	Churchill Hospital	Gemma Austin (nee Glover)	
Oxford	Churchill Hospital	Hazel Wynn	

Oxford	Churchill Hospital	Jane Boutflower	
Oxford	Churchill Hospital	Sarah Lawrey	
Oxford	Churchill Hospital	Trish Green	
Oxford	Churchill Hospital	Usharani Devi Wahengbam	
Oxford	Churchill Hospital	Andrew Protheroe	PI
Paisley	Royal Alexandra Hospital	Tiago Rodrigues	
Poole	Poole Hospital	Fiona Mellor	
Poole	Poole Hospital	Joseph Davies	Co-I
Poole	Poole Hospital	Yogesh Nishchal	Co-I
Poole	Poole Hospital	Joe Davies	
Poole	Poole Hospital	Maxine Flubacher	
Poole	Poole Hospital	May Lwin	
Poole	Poole Hospital	Perric Crellin	Co-I
Poole	Poole Hospital	Sue Brock	PI
Poole	Poole Hospital	Becky Troke	
Poole	Poole Hospital	Felicity Clapp	
Poole	Poole Hospital	Sarah Patch	
Poole	Poole Hospital	Elizabeth Woodward	
Poole	Poole Hospital	Nichola Downs	
Poole	Poole Hospital	Craig Vincent	
Poole	Poole Hospital	Neal Beamish	
Poole	Poole Hospital	Roger Wheelwright	
Poole	Poole Hospital	Deryck Burton	
Poole	Poole Hospital	Lee Tbaily	
Poole	Poole Hospital	Emma Wesley	

Poole	Poole Hospital	Emma Williams	
Poole	Poole Hospital	Helen Morling	
Poole	Poole Hospital	Kate Mutendera	
Poole	Poole Hospital	Kate Urquhart	
Poole	Poole Hospital	Louise Heckford	
Poole	Poole Hospital	Savina Elitova	Pharmacist
Poole	Poole Hospital	Delia Whiteman	
Poole	Poole Hospital	Elizabeth Clarke	
Poole	Poole Hospital	Lyn Jackson	
Poole	Poole Hospital	Sally Munden	
Poole	Poole Hospital	Sharon Power	Pharmacist
Poole	Poole Hospital	Sophie Rix	
Poole	Poole Hospital	Stephanie Jones	
Poole	Poole Hospital	Amanda Iskender	
Poole	Poole Hospital	Hilary Blaney	
Poole	Poole Hospital	Sally Gillespie	
Poole	Poole Hospital	Sandy Pressdee	
Poole	Poole Hospital	Sara Orford	
Poole	Poole Hospital	Seonaid Wright	
Poole	Poole Hospital	Susan Saxby	
Poole	Poole Hospital	Teresa Coffin	
Portadown	Craigavon Area Hospital	Fionnuala Houghton	Co-I
Portadown	Craigavon Area Hospital	Judith Carser	PI
Portadown	Craigavon Area Hospital	Leanne McCourt	
Portsmouth	Queen Alexandra Hospital	Akash Maniam	

Portsmouth	Queen Alexandra Hospital	Akash Maniam	
Portsmouth	Queen Alexandra Hospital	Alice White	
Portsmouth	Queen Alexandra Hospital	Alisha Damani	
Portsmouth	Queen Alexandra Hospital	Azarel Virgo	
Portsmouth	Queen Alexandra Hospital	Caroline Chau	
Portsmouth	Queen Alexandra Hospital	Charlotte Davies	
Portsmouth	Queen Alexandra Hospital	Chloe Holden	
Portsmouth	Queen Alexandra Hospital	Eleanor Jones	
Portsmouth	Queen Alexandra Hospital	Harliana Mohd Yusof	Co-I
Portsmouth	Queen Alexandra Hospital	Jack Broadfoot	
Portsmouth	Queen Alexandra Hospital	Jeng Heng Ching	
Portsmouth	Queen Alexandra Hospital	Joanna Hack	Co-I
Portsmouth	Queen Alexandra Hospital	Joni Howells	
Portsmouth	Queen Alexandra Hospital	Kudingila Madhava	
Portsmouth	Queen Alexandra Hospital	Mark Noble	
Portsmouth	Queen Alexandra Hospital	Matthew Moe	
Portsmouth	Queen Alexandra Hospital	Megan Rowley	
Portsmouth	Queen Alexandra Hospital	Mona Hassan	
Portsmouth	Queen Alexandra Hospital	Nataliya Martynyuk	
Portsmouth	Queen Alexandra Hospital	Oluwatobi Adeagbo	Co-I
Portsmouth	Queen Alexandra Hospital	Shyamkia Acharige	Co-I
Portsmouth	Queen Alexandra Hospital	Syed Shah	Co-I
Portsmouth	Queen Alexandra Hospital	Umapathy Hombaiah	Co-I
Portsmouth	Queen Alexandra Hospital	Vara Prasad Devara	
Portsmouth	Queen Alexandra Hospital	Victoria True	

Portsmouth	Queen Alexandra Hospital	Yvonne Mangan	
Portsmouth	Queen Alexandra Hospital	Joanna Gale	PI
Portsmouth	Queen Alexandra Hospital	Khalid Hameed	
Portsmouth	Queen Alexandra Hospital	Mario Uccello	
Portsmouth	Queen Alexandra Hospital	May Lwin	
Portsmouth	Queen Alexandra Hospital	Mya Gyi	
Portsmouth	Queen Alexandra Hospital	Sarah Ellis	
Portsmouth	Queen Alexandra Hospital	Yoodhvir Nagar	
Portsmouth	Queen Alexandra Hospital	Daniel Bloomfield	
Portsmouth	Queen Alexandra Hospital	Dominic Hodgson	
Portsmouth	Queen Alexandra Hospital	Robert Williams	
Portsmouth	Queen Alexandra Hospital	Jennifer Hale	
Portsmouth	Queen Alexandra Hospital	Jillian Andrews	
Portsmouth	Queen Alexandra Hospital	Tracey Dobson	
Portsmouth	Queen Alexandra Hospital	Tracy Callen	
Portsmouth	Queen Alexandra Hospital	Wendy Golding	Pharmacist
Portsmouth	Queen Alexandra Hospital	Wendy Stacey	
Portsmouth	Queen Alexandra Hospital	Catherine Tolentino	
Portsmouth	Queen Alexandra Hospital	Giuseppe Banna	Co-I
Portsmouth	Queen Alexandra Hospital	Heather Cuell	
Portsmouth	Queen Alexandra Hospital	Kathy Blight	Pharmacist
Portsmouth	Queen Alexandra Hospital	Lisa Murray	
Portsmouth	Queen Alexandra Hospital	Mila Roca	
Portsmouth	Queen Alexandra Hospital	Anna Stephenson	
Portsmouth	Queen Alexandra Hospital	Badrriyya Mohamedali	

Portsmouth	Queen Alexandra Hospital	Catrin Watkinson	
Portsmouth	Queen Alexandra Hospital	Lorna Meadows	
Portsmouth	Queen Alexandra Hospital	Mary Wands	Pharmacist
Postfach 834	Kantonsspital Winterthur	Claudia Langer	
Postfach 834	Kantonsspital Winterthur	Miklos Pless	
Postfach 834	Kantonsspital Winterthur	Natalie Fisher	
Postfach 834	Kantonsspital Winterthur	Sabina Schacher	
Postfach 834	Kantonsspital Winterthur	SusyAnn Shaw	
Postfach 834	Kantonsspital Winterthur	Veronika Nagy	
Postfach 834	Kantonsspital Winterthur		
Postfach 834	Kantonsspital Winterthur	Beatrice Brinkers	
Postfach 834	Kantonsspital Winterthur	Cindy Wanger	
Postfach 834	Kantonsspital Winterthur	Martina Pfitzner	
Postfach 834	Kantonsspital Winterthur	Nicole Kradolfer	
Postfach 834	Kantonsspital Winterthur	Hubert John	PI
Prescot Street	Royal Liverpool University Hospital	Lynsey Dean	
Preston	Royal Preston Hospital	Christina Hague	Co-I
Preston	Royal Preston Hospital	Jose Rico	Co-I
Preston	Royal Preston Hospital	Martin Swinton	
Preston	Royal Preston Hospital	Natalie Charnley	Co-I
Preston	Royal Preston Hospital	Nicola Flaum	Co-I
Preston	Royal Preston Hospital	Omi Parikh	Co-I
Preston	Royal Preston Hospital	Sophie Raby	Co-I
Preston	Royal Preston Hospital	William Croxford	
Preston	Royal Preston Hospital	Yee Pei Song	Co-I

Preston	Royal Preston Hospital	Alison Birtle	PI
Preston	Royal Preston Hospital	Anna Macpherson	
Preston	Royal Preston Hospital	Catherine Thompson	
Preston	Royal Preston Hospital	Deborah Williamson	
Preston	Royal Preston Hospital	Duleer Majeed	Co-I
Preston	Royal Preston Hospital	Falalu Danwata	
Preston	Royal Preston Hospital	Marcus Wise	Co-I
Preston	Royal Preston Hospital	Norma Sidek	
Preston	Royal Preston Hospital	Shahzad Gul	
Preston	Royal Preston Hospital	Tanmay Mukhopadhyay	
Preston	Royal Preston Hospital	Win Soe	
Preston	Royal Preston Hospital	Claire Corless	
Preston	Royal Preston Hospital	Rebecca Wilby (nee Hall)	
Preston	Royal Preston Hospital	Rose Ellard	
Preston	Royal Preston Hospital	Sarah Preston	
Preston	Royal Preston Hospital	Margaret Brunton	
Preston	Royal Preston Hospital	Andrew Martyniak	
Preston	Royal Preston Hospital	Davide Garau	
Preston	Royal Preston Hospital	Dominic Mounsey	
Preston	Royal Preston Hospital	Billy Hefferon	
Preston	Royal Preston Hospital	David Barber	Pharmacist
Preston	Royal Preston Hospital	Hemant Patel	
Preston	Royal Preston Hospital	Roy Shentall	
Preston	Royal Preston Hospital	Cassandra Gleeson	
Preston	Royal Preston Hospital	Deborah Weavers	

Preston	Royal Preston Hospital	Haiyan Huang	
Preston	Royal Preston Hospital	Mandy Armstrong	
Preston	Royal Preston Hospital	Nafisa Arden	
Preston	Royal Preston Hospital	Sharon Curran	
Preston	Royal Preston Hospital	Shelia Calvert	
Preston	Royal Preston Hospital	Andrea Ashton	
Preston	Royal Preston Hospital	Louise Hough	Pharmacist
Preston	Royal Preston Hospital	Catherine Walmsley	
Preston	Royal Preston Hospital	Christina Robinson	
Preston	Royal Preston Hospital	Deepsi Khatiwada	
Preston	Royal Preston Hospital	Hazel Preston	
Preston	Royal Preston Hospital	Amanda Alty	
Preston	Royal Preston Hospital	Caroline Hatch	
Preston	Royal Preston Hospital	Claire Hennigan	
Preston	Burnley General Hospital	Helen Spickett	
Preston	Royal Blackburn Hospital	Helen Spickett	
Preston	Royal Preston Hospital	Katherine Ashton	
Preston	Royal Preston Hospital	Nina Vekaria	
Preston	Royal Preston Hospital	Patricia Knight	
Preston	Royal Preston Hospital	Stephanie Cornthwaite	
Reading	Royal Berkshire Hospital	Elizabeth Haydon	
Reading	Royal Berkshire Hospital	Emilia Bruton	Co-I
Reading	Royal Berkshire Hospital	Gagan Bhatnagar	
Reading	Royal Berkshire Hospital	Nicola Dallas	
Reading	Royal Berkshire Hospital	Osamah Alasadi	

Reading	Royal Berkshire Hospital	Osamah Al-Asadi	Co-I
Reading	Royal Berkshire Hospital	Phillip Webb	
Reading	Royal Berkshire Hospital	Phillipa Johnstone	
Reading	Royal Berkshire Hospital	Rowena Cazalet	Co-I
Reading	Royal Berkshire Hospital	Silke Hahnewald	
Reading	Royal Berkshire Hospital	Abdolnasser Aminiraouf	
Reading	Royal Berkshire Hospital	Ali Abbas	
Reading	Royal Berkshire Hospital	Georges Sinclair	
Reading	Royal Berkshire Hospital	Helen O'Donnell	
Reading	Royal Berkshire Hospital	Paul Rogers	PI
Reading	Royal Berkshire Hospital	Rebecca Johnson	Co-I
Reading	Royal Berkshire Hospital	Rebecca Varatharajah	Co-I
Reading	Royal Berkshire Hospital	Richard B Brown	
Reading	Royal Berkshire Hospital	Robert Jones	
Reading	Royal Berkshire Hospital	Sean O'Cathail	
Reading	Royal Berkshire Hospital	Shawn Ellis	
Reading	Royal Berkshire Hospital	Simon Wyatt	
Reading	Royal Berkshire Hospital	Stephen Parr	
Reading	Royal Berkshire Hospital	James Church	
Reading	Royal Berkshire Hospital	Steven Gulliver	
Reading	Royal Berkshire Hospital	Thomas Kindley	
Reading	Royal Berkshire Hospital	Geraldine Mason	Pharmacist
Reading	Royal Berkshire Hospital	Kate Preston	
Reading	Royal Berkshire Hospital	Nicole Gould	
Reading	Royal Berkshire Hospital	Wioletta Kowalczyk-Williams	

Reading	Royal Berkshire Hospital	Christina Lewis	
Reading	Royal Berkshire Hospital	Pooja Pabari	
Reading	Royal Berkshire Hospital	Royda Hadi	
Reading	Royal Berkshire Hospital	Andreia da Cruz	
Reading	Royal Berkshire Hospital	Anna Gillham	
Reading	Royal Berkshire Hospital	Claire Connolly	
Reading	Royal Berkshire Hospital	Emma Vowell	
Reading	Royal Berkshire Hospital	Jo Hand	
Reading	Royal Berkshire Hospital	Maxine Gauntlett	
Reading	Royal Berkshire Hospital	Omotola Ogunnigbo	Pharmacist
Reading	Royal Berkshire Hospital	Sanita Gurm	
Reading	Royal Berkshire Hospital	Allison Hunt	
Reading	Royal Berkshire Hospital	Catherine Deytrikh-Smith	
Reading	Royal Berkshire Hospital	Debbie Cartwright	
Reading	Royal Berkshire Hospital	Fiona Everson	
Reading	Royal Berkshire Hospital	Gabrielle Ball	
Reading	Royal Berkshire Hospital	Helen Purdon	
Reading	Royal Berkshire Hospital	Jane Atkinson	
Reading	Royal Berkshire Hospital	Juliette Dye	
Reading	Royal Berkshire Hospital	Kirsty Horwood	Pharmacist
Reading	Royal Berkshire Hospital	Kristy Coomber	
Reading	Royal Berkshire Hospital	Maryam Amole	
Reading	Royal Berkshire Hospital	Norma Shields	Pharmacist
Reading	Royal Berkshire Hospital	Sian James	
Reading	Royal Berkshire Hospital	Suzanne Foxwell	

Reading	Royal Berkshire Hospital	Tolu Okeke	
Redditch	Alexandra Hospital	Ayyaz Munawar	Co-I
Redditch	Alexandra Hospital	Bartlomeij Kurec	
Redditch	Alexandra Hospital	Menna Fonda	Co-I
Redditch	Alexandra Hospital	Mujtaba Syed-Khaja	Co-I
Redditch	Alexandra Hospital	Nge Nge Thida	Co-I
Redditch	Alexandra Hospital	Asha Sivapalasuntharam	
Redditch	Alexandra Hospital	James Best	Co-I
Redditch	Alexandra Hospital	Joanna Hamilton	
Redditch	Alexandra Hospital	Lisa Capaldi	PI
Redditch	Alexandra Hospital	Paul Flinders	
Redditch	Alexandra Hospital	Sabihya Wontumi	Co-I
Redditch	Alexandra Hospital	Thakshayini Shanthakumar	Co-I
Redditch	Alexandra Hospital	Jacob Taylor	
Redditch	Alexandra Hospital	Jonathan Davies	
Redditch	Alexandra Hospital	Alison Harrison	
Redditch	Alexandra Hospital	Amanda Holdsworth	
Redditch	Alexandra Hospital	Ann White	
Redditch	Alexandra Hospital	Jayna Thakrar	
Redditch	Alexandra Hospital	Veronica Rowlands	
Redditch	Alexandra Hospital	Hayley Hodson	
Redditch	Alexandra Hospital	Helen Tranter	
Redditch	Alexandra Hospital	Maggie Brown	
Redditch	Alexandra Hospital	Stephanie Cook	Pharmacist
Redditch	Alexandra Hospital	Jennifer Young	Pharmacist

Redditch	Alexandra Hospital	Jeanette Knapp	
Redhill	East Surrey Hospital	Eva Letalova	
Romford	Queen's Hospital (Romford)	Alexander Pawsey	Co-I
Romford	Queen's Hospital (Romford)	Danny Koroma Koroma	
Romford	Queen's Hospital (Romford)	Kathryn Tarver	
Romford	Queen's Hospital (Romford)	Maria Martinou	
Romford	Queen's Hospital (Romford)	Jonathon Shamash	
Romford	Queen's Hospital (Romford)	Amy Lewis	PI
Romford	Queen's Hospital (Romford)	Ramachandran Subramaniam	
Romford	Queen's Hospital (Romford)	Stephanie Gibbs	
Romford	Queen's Hospital (Romford)	Thi Vu	
Romford	Queen's Hospital (Romford)	Mohammed Rashid Khan	
Romford	Queen's Hospital (Romford)	Revanth Jannapureddy	
Romford	Queen's Hospital (Romford)	Samuel Mugari	
Romford	Queen's Hospital (Romford)	Simerjyot Mudhar	
Romford	Queen's Hospital (Romford)	Neale O'Brien	
Romford	Oldchurch Hospital	Neil Fisher	
Romford	Queen's Hospital (Romford)	Yousaf Razzak	
Romford	Queen's Hospital (Romford)	Parveen Dugh	Pharmacist
Romford	Queen's Hospital (Romford)	Amani Chowdhury	
Romford	Queen's Hospital (Romford)	Mariha Khalid	
Romford	Queen's Hospital (Romford)	Ana-Marie Pena-Remorin	
Romford	Queen's Hospital (Romford)	Dalisay Domingo	
Romford	Queen's Hospital (Romford)	Helen Mackenzie	
Romford	Queen's Hospital (Romford)	Tina Mills-Baldock	

Runcorn	Warrington Hospital	Isabel Syndikus	PI
Runcorn	Warrington Hospital	Shaun Tolan	Co-I
Runcorn	Halton Hospital	Duncan Knowles	
Runcorn	Halton Hospital	Ian Allen	
Runcorn	Halton Hospital	Andrea Young	
Runcorn	Halton Hospital	Rebecca Madew (nee Tinker)	
Runcorn	Halton Hospital	Nemonie Marriott	
Salford	Salford Royal Hospital	Nicholas Boxall	
Salford	Salford Royal Hospital	Anna Tran	Co-I
Salford	Salford Royal Hospital	Richard Cowan	
Salford	Salford Royal Hospital	Danielle Platt	
Salford	Salford Royal Hospital	Elina Jose	
Salford	Salford Royal Hospital	Joanne Henry	
Salford	Salford Royal Hospital	Kay Goulden	
Salford	Salford Royal Hospital	Euan Green	Co-I
Salford	Salford Royal Hospital	Maurice Lau	Co-I
Salford	Salford Royal Hospital	Oliver Wadsworth	
Salford	Salford Royal Hospital	Tony Elliott	Co-I
Salford	Salford Royal Hospital	Chris Betts	
Salford	Salford Royal Hospital	David Shackley	
Salford	Salford Royal Hospital	Jason Howard	Pharmacist
Salford	Salford Royal Hospital	Kieran O'Flynn	
Salford	Salford Royal Hospital	Mark Stapleton	
Salford	Salford Royal Hospital	Richard Jones	
Salford	Salford Royal Hospital	Christine Farnworth	

Salford	Salford Royal Hospital	Claire Duncan (nee Keatley)	Pharmacist
Salford	Salford Royal Hospital	Claire Keatley	
Salford	Salford Royal Hospital	Kathryn Cawley	
Salford	Salford Royal Hospital	Siny George	
Salford	Salford Royal Hospital	Cellins Vinod	
Salford	Salford Royal Hospital	Claire Dickson	
Salford	Salford Royal Hospital	Helen Farrell	
Salford	Salford Royal Hospital	Samia Hanif	
Salford	Salford Royal Hospital	Soney Dharmaprasad	
Salford	Salford Royal Hospital	Angela Ashton	
Salford	Salford Royal Hospital	Anne-Marie Peers	Pharmacist
Salford	Salford Royal Hospital	Ashley Harris	Pharmacist
Salford	Salford Royal Hospital	Catherine Redshaw	
Salford	Salford Royal Hospital	Jean Jellicoe	
Salford	Salford Royal Hospital	Kathryn Fry	
Salford	Salford Royal Hospital	Leah Harter	
Salford	Salford Royal Hospital	Rachael Allen	
Salford	Salford Royal Hospital	Vicky Thomas	
Salford	Salford Royal Hospital	Noel Clarke	PI
Salford	Salford Royal Hospital	Jill Youd	
Salford	Salford Royal Hospital	Melanie Taylor	
Salford	Salford Royal Hospital	Sarah Kirk	
Scarborough	Scarborough General Hospital	Ian Renwick	
Scarborough	Scarborough General Hospital	Khaliq Rehman	PI
Scarborough	Scarborough General Hospital	Laith Alsaket	

Scarborough	Scarborough General Hospital	Mohan Hingorani	
Scarborough	Scarborough General Hospital	Nabil El-Mahdawi	
Scarborough	Scarborough General Hospital	Mohammad Muneeb Khan	
Scarborough	Scarborough General Hospital	Richard Khafagy	
Scarborough	Scarborough General Hospital	Russell Morgan	
Scarborough	Scarborough General Hospital	Abigail Rowbotham	
Scarborough	Scarborough General Hospital	Caroline Savage	
Scarborough	Scarborough General Hospital	Diana Ionita	
Scarborough	Scarborough General Hospital	Fizzah Asif	
Scarborough	Scarborough General Hospital	Lydia Kerr	
Scarborough	Scarborough General Hospital	Poppy Cottrell-Howe	Pharmacist
Scarborough	Scarborough General Hospital	Courtney Cole	
Scarborough	Scarborough General Hospital	Donna Anderson	
Scarborough	Scarborough General Hospital	Rachel Spooner	
Scarborough	Scarborough General Hospital	Callum Childs	
Scarborough	Scarborough General Hospital	Dominic Burns	
Scarborough	Scarborough General Hospital	Andrew Robertson	
Scarborough	Scarborough General Hospital	Arran Fletcher	
Scarborough	Scarborough General Hospital	Jordan Toohie	
Scarborough	Scarborough General Hospital	Kevin Brame	
Scarborough	Scarborough General Hospital	Paul Wood	Pharmacist
Scarborough	Scarborough General Hospital	Simon Hawkyard	Co-I
Scarborough	Scarborough General Hospital	Jacqui Smith	
Scarborough	Scarborough General Hospital	Lisa Armitage	
Scarborough	Scarborough General Hospital	Samantha Stead	

Scarborough	Scarborough General Hospital	Tania Neale	
Scarborough	Scarborough General Hospital	Tanya Hartley	
Scarborough	Scarborough General Hospital	Alison Ames	
Scarborough	Scarborough General Hospital	Alison Turnbull	
Scarborough	Scarborough General Hospital	Anne Nunn	
Scarborough	Scarborough General Hospital	Joanne Fletcher	
Scarborough	Scarborough General Hospital	Rachel Harrison	
Scarborough	Scarborough General Hospital	Sacha Honour	Pharmacist
Scarborough	Scarborough General Hospital	Adnan Kabir	
Scarborough	Scarborough General Hospital	Alicia Rodgers	
Scarborough	Scarborough General Hospital	Janine Mallinson	
Scarborough	Scarborough General Hospital	Laura Barman	
Scarborough	Scarborough General Hospital	Pippa Carlton-Rylance	
Scarborough	Scarborough General Hospital	Polly Needs	
Scarborough	Scarborough General Hospital	Amie Stewart	
Scarborough	Scarborough General Hospital	Carol Popplestone	
Scarborough	Scarborough General Hospital	Cheryl Donne	Pharmacist
Scarborough	Scarborough General Hospital	Chloe Box	Pharmacist
Scarborough	Scarborough General Hospital	Jane Taylor	Pharmacist
Scarborough	Scarborough General Hospital	Sarah Kent	
Scarborough	Scarborough General Hospital	Tracey Hawkes	
Scarborough	Scarborough General Hospital	Vic Gacek	
Sheffield	Weston Park Hospital	Lucy Walkington	
Sheffield	Doncaster Royal Infirmary	Mymoona Alzouebi	
Sheffield	Weston Park Hospital	Mymoona Alzouebi	

Sheffield	Weston Park Hospital	Prashanth Sanganalmath	
Sheffield	Weston Park Hospital	Roseleen Sheehan	
Sheffield	Weston Park Hospital	Shabbir Rawther	Co-I
Sheffield	Weston Park Hospital	Tathagata Das	
Sheffield	Weston Park Hospital	Virgil Sivoglo	Co-I
Sheffield	Weston Park Hospital	Carmel Pezaro	
Sheffield	Weston Park Hospital	Shiyam Kumar	
Sheffield	Weston Park Hospital	Catherine Ferguson	
Sheffield	Weston Park Hospital	James Lester	
Sheffield	Weston Park Hospital	Janet Brown	
Sheffield	Weston Park Hospital	Katie Bowen	
Sheffield	Weston Park Hospital	Linda Evans	
Sheffield	Weston Park Hospital	Louise Murray	
Sheffield	Weston Park Hospital	Omar Din	PI
Sheffield	Weston Park Hospital	Peter Kirkbride	
Sheffield	Weston Park Hospital	Chloe Clegg	
Sheffield	Weston Park Hospital	Georgia Douglas	
Sheffield	Weston Park Hospital	Jessica Medcalf	
Sheffield	Weston Park Hospital	Rebecca Lomax-Allen	
Sheffield	Weston Park Hospital	Steffy George	
Sheffield	Weston Park Hospital	Alexandra Firth	
Sheffield	Weston Park Hospital	Cyper Allan	
Sheffield	Weston Park Hospital	Ryan Asher	
Sheffield	Weston Park Hospital	Ryan Davies	
Sheffield	Weston Park Hospital	John Martindale	

Sheffield	Doncaster Royal Infirmary	Mark Holliday	
Sheffield	Weston Park Hospital	Mark Holliday	
Sheffield	Weston Park Hospital	Richard Brown	
Sheffield	Weston Park Hospital	Roger Burkinshaw	
Sheffield	Weston Park Hospital	Anne Smythe	
Sheffield	Weston Park Hospital	Eileen Marsh	
Sheffield	Weston Park Hospital	Gemma Dale	
Sheffield	Weston Park Hospital	Janine Smedley (nee McCabe)	
Sheffield	Weston Park Hospital	Julia Disney	Pharmacist
Sheffield	Weston Park Hospital	Kate Gibbins	
Sheffield	Weston Park Hospital	Jess Aldred	
Sheffield	Weston Park Hospital	Leigh Fiorentino	
Sheffield	Weston Park Hospital	Lucy Birch	
Sheffield	Weston Park Hospital	Ruta Segamogaite	
Sheffield	Weston Park Hospital	Catherine Spalton	
Sheffield	Weston Park Hospital	Elizabeth Hodgkinson	Pharmacist
Sheffield	Weston Park Hospital	Joanne Bird	
Sheffield	Weston Park Hospital	Katherine Williams	
Sheffield	Weston Park Hospital	Lucy Smith	
Sheffield	Weston Park Hospital	Lynne Ashmore	
Sheffield	Weston Park Hospital	Marion Hutchinson	Pharmacist
Sheffield	Weston Park Hospital	Rachel Toes	
Sheffield	Weston Park Hospital	Sarah Brown	
Sheffield	Weston Park Hospital	Susan Bishop	
Sheffield	Weston Park Hospital	Kim Wood	

Shrewsbury	Royal Shrewsbury Hospital	Gemma Searle	
Shrewsbury	Royal Shrewsbury Hospital	Shazad Aslam	
Shrewsbury	Royal Shrewsbury Hospital	Abel Zachariah	
Shrewsbury	Royal Shrewsbury Hospital	Aitzaz Qaisar	
Shrewsbury	Royal Shrewsbury Hospital	Beshar Allos	
Shrewsbury	Royal Shrewsbury Hospital	Erica Beaumont	
Shrewsbury	Royal Shrewsbury Hospital	Huzeifa Abdel	
Shrewsbury	Royal Shrewsbury Hospital	Huzeifa Gadir	
Shrewsbury	Royal Shrewsbury Hospital	James Best	
Shrewsbury	Royal Shrewsbury Hospital	Lisa Capaldi	
Shrewsbury	Royal Shrewsbury Hospital	Lucy Pennant	
Shrewsbury	Royal Shrewsbury Hospital	Mathai Varghese	
Shrewsbury	Royal Shrewsbury Hospital	Narayanan Srihari	PI
Shrewsbury	Royal Shrewsbury Hospital	Qamar Ghafoor	
Shrewsbury	Royal Shrewsbury Hospital	Rajanee Bhana	
Shrewsbury	Royal Shrewsbury Hospital	Sundus Yahya	
Shrewsbury	Royal Shrewsbury Hospital	Alison Tilley	
Shrewsbury	Royal Shrewsbury Hospital	Anna Law	
Shrewsbury	Royal Shrewsbury Hospital	Danielle Childs	
Shrewsbury	Royal Shrewsbury Hospital	Danielle Childs	
Shrewsbury	Royal Shrewsbury Hospital	Gemma Lee	
Shrewsbury	Royal Shrewsbury Hospital	Jenny Simm	
Shrewsbury	Royal Shrewsbury Hospital	Lisa Evans	
Shrewsbury	Royal Shrewsbury Hospital	Nicola Henderson	
Shrewsbury	Royal Shrewsbury Hospital	Riquella Abbott	

Shrewsbury	Royal Shrewsbury Hospital	Suzanne Pope	
Shrewsbury	Royal Shrewsbury Hospital	Andy Taylor	
Shrewsbury	Royal Shrewsbury Hospital	Michael Leigh	
Shrewsbury	Royal Shrewsbury Hospital	Ravi Prashant	Co-I
Shrewsbury	Royal Shrewsbury Hospital	Craig Pickering	
Shrewsbury	Royal Shrewsbury Hospital	Sanal Jose	
Shrewsbury	Royal Shrewsbury Hospital	Emma Weaver	
Shrewsbury	Royal Shrewsbury Hospital	Mandy Beekes	
Shrewsbury	Royal Shrewsbury Hospital	Natasha Wallbank	
Shrewsbury	Royal Shrewsbury Hospital	Rachel McGregor	
Shrewsbury	Royal Shrewsbury Hospital	Gill Ferguson	Pharmacist
Shrewsbury	Royal Shrewsbury Hospital	Harpreet Singh	
Shrewsbury	Royal Shrewsbury Hospital	Hayley Hughes	
Shrewsbury	Royal Shrewsbury Hospital	Sandra Smith	
Shrewsbury	Royal Shrewsbury Hospital	Sunita Kurian-Downer	
Shrewsbury	Royal Shrewsbury Hospital	Indukala Chennattukungu	
Shrewsbury	Royal Shrewsbury Hospital	Jenny Lakin	
Shrewsbury	Royal Shrewsbury Hospital	Angela Yeomans	Pharmacist
Shrewsbury	Royal Shrewsbury Hospital	Catherine Santiago	
Shrewsbury	Royal Shrewsbury Hospital	Elena Michael	
Shrewsbury	Royal Shrewsbury Hospital	Karen Nicholas	
Shrewsbury	Royal Shrewsbury Hospital	Mandy Bates	
Shrewsbury	Royal Shrewsbury Hospital	Nicola Jones	
Shrewsbury	Royal Shrewsbury Hospital	Rebecca Wilcox	
Shrewsbury	Royal Shrewsbury Hospital	Renee Poulsom	Pharmacist

Shrewsbury	Royal Shrewsbury Hospital	Sally Potts	
Shrewsbury	Royal Shrewsbury Hospital	Siobhan Kilbane	
Shrewsbury	Royal Shrewsbury Hospital	Verity King	
Shrewsbury	Royal Shrewsbury Hospital	Catherine Orrell	
Shrewsbury	Royal Shrewsbury Hospital	Emma Neeves	
Shrewsbury	Royal Shrewsbury Hospital	Helen Moore	
Shrewsbury	Royal Shrewsbury Hospital	Marion Adams	
South Shields	South Tyneside District Hospital	Ashraf Azzabi	PI
South Shields	South Tyneside District Hospital	Sally Hall	
South Shields	South Tyneside District Hospital	Judith Mckenna	
South Shields	South Tyneside District Hospital	Amy Burns	
South Shields	South Tyneside District Hospital	Jessica De Sousa	
South Shields	South Tyneside District Hospital	Judith Moore	
South Shields	South Tyneside District Hospital	Sue Morrison	
South Shields	South Tyneside District Hospital	Maxine Turner	Pharmacist
Southampton	Southampton General Hospital	Caroline Chan	
Southampton	Southampton General Hospital	Caroline Chau	
Southampton	Southampton General Hospital	Chloe Holden	Co-I
Southampton	Southampton General Hospital	Chris Coyle	
Southampton	Southampton General Hospital	Emma Brown	Co-I
Southampton	Southampton General Hospital	Kim Teasdale	Co-I
Southampton	Southampton General Hospital	Matthew Wheater	
Southampton	Southampton General Hospital	Robert Kemp	
Southampton	Southampton General Hospital	Simon Crabb	PI
Southampton	Southampton General Hospital	Tessa Greenhalgh	Co-I

Southampton	Southampton General Hospital	Catherine Heath	
Southampton	Southampton General Hospital	Graham Mead	
Southampton	Southampton General Hospital	Harish Reddy	Co-I
Southampton	Southampton General Hospital	Victoria McFarlane	
Southampton	Southampton General Hospital	Ivanila Atauasova	Pharmacist
Southampton	Southampton General Hospital	Anna Sieradzka	
Southampton	Southampton General Hospital	Anna Stephenson	
Southampton	Southampton General Hospital	Nithya Raj	
Southampton	Southampton General Hospital	Rajitha Kamalakshan	
Southampton	Southampton General Hospital	Rebecca Rice	
Southampton	Southampton General Hospital	Adele Ruiz	
Southampton	Southampton General Hospital	Julie Kennedy	
Southampton	Southampton General Hospital	Susan Morton	
Southampton	Southampton General Hospital	Aneta Zahorska	
Southampton	Southampton General Hospital	Annelise Haskell	
Southampton	Southampton General Hospital	Archana Gadve	
Southampton	Southampton General Hospital	Caroline Andrews	
Southampton	Southampton General Hospital	Carolyn Mitchell	Pharmacist
Southampton	Southampton General Hospital	Deborah Scott	
Southampton	Southampton General Hospital	Holly Burton	Pharmacist
Southampton	Southampton General Hospital	Leanne Reader	
Southampton	Southampton General Hospital	Liane Armstrong	
Southampton	Southampton General Hospital	Lucy Galloway	
Southampton	Southampton General Hospital	Maureen McAuley	Pharmacist
Southampton	Southampton General Hospital	Nikki Carney	

Southampton	Southampton General Hospital	Nikki Prewitt	
Southampton	Southampton General Hospital	Oyeleye Oyebola	
Southampton	Southampton General Hospital	Sarah Oliver	
Southampton	Southampton General Hospital	Sau-Mon Tsang	Pharmacist
Southampton	Southampton General Hospital	Yanli Li	
Southampton	Southampton General Hospital	Carina Mundy	
Southampton	Southampton General Hospital	Donna Kimber	Pharmacist
Southampton	Southampton General Hospital	Fabiola Morales-Azofra	
Southampton	Southampton General Hospital	Julie Gwilt	
Southampton	Southampton General Hospital	Kirsty Cumming	
Southampton	Southampton General Hospital	Lorraine Street	
Southampton	Southampton General Hospital	Lucy Elswood	
Southampton	Southampton General Hospital	Shauna Wakefield	
Southport	Southport and Formby District General Hospital	Manal Alameddine	PI
Southport	Southport and Formby District General Hospital	Neeraj Bhalla	PI
Southport	Southport and Formby District General Hospital	Asha Sivapalasuntharam	
Southport	Southport and Formby District General Hospital	Chinnamani Eswar	
Southport	Southport and Formby District General Hospital	Margaret Brunton	
Southport	Southport and Formby District General Hospital	Ken Gardner	
Southport	Southport and Formby District General Hospital	Laurie Lomax	
Southport	Southport and Formby District General Hospital	Ann Wearing	
Southport	Southport and Formby District General Hospital	Dawn Barker	
Southport	Southport and Formby District General Hospital	Marie McBride	
Southport	Southport and Formby District General Hospital	Sandra Robinson	
Southport	Southport and Formby District General Hospital	Anna Morris	

Southport	Southport and Formby District General Hospital	Heidi Moran	
Southport	Southport and Formby District General Hospital	Teresa Monahan	
Southport	Southport and Formby District General Hospital	Angela Scullion	
Southport	Southport and Formby District General Hospital	Lisa Dobson (nee Child)	
Southport	Southport and Formby District General Hospital	Linda Schinkel	
St Gallen	Kantonsspital St Gallen	Aurelius Omlin	Co-I
St Gallen	Kantonsspital St Gallen	Christoph Schwab	Co-I
St Gallen	Kantonsspital St Gallen	Dominik Abt	
St Gallen	Kantonsspital St Gallen	Christian Rothermundt	Co-I
St Gallen	Kantonsspital St Gallen	Daniel Engeler	PI
St Gallen	Kantonsspital St Gallen	Mannel Jungi	
St Gallen	Kantonsspital St Gallen	Silke Gillessen	
St Gallen	Kantonsspital St Gallen	Stefan Prensser	
St Gallen	Kantonsspital St Gallen	Sigrid Patel	
St Gallen	Kantonsspital St Gallen	Eloise Kremer	
St Gallen	Kantonsspital St Gallen	Karin Zuern	
St Gallen	Kantonsspital St Gallen	Karin Zurn	
St Gallen	Kantonsspital St Gallen	Claudia Hormann	
St Gallen	Kantonsspital St Gallen	Sibylle Schapper	
St Leonards-on-Sea	Conquest Hospital	Aspasia Soultati	
St Leonards-on-Sea	Conquest Hospital	Fiona McKinna	
St Leonards-on-Sea	Conquest Hospital	Kathryn Lees	
St Leonards-on-Sea	Conquest Hospital	Sharon Beesley	
St Leonards-on-Sea	Conquest Hospital	Claire Rutherford	
St Leonards-on-Sea	Conquest Hospital	Sarah Draper	

St Leonards-on-Sea	Conquest Hospital	Mark Whitfield	Pharmacist
St Leonards-on-Sea	Conquest Hospital	Steve Garnett	
St Leonards-on-Sea	Conquest Hospital	Gail Pottinger	
St Leonards-on-Sea	Conquest Hospital	Jo-Anne Taylor	
St Leonards-on-Sea	Conquest Hospital	Kay Jones-Skipper	
St Leonards-on-Sea	Conquest Hospital	Atikah Ayaz	
St Leonards-on-Sea	Conquest Hospital	Lauren McCrisken	
St Leonards-on-Sea	Conquest Hospital	Sarah Goodwin	
St Leonards-on-Sea	Conquest Hospital	Theresa Baumber	
St Leonards-on-Sea	Conquest Hospital	Joanna Howard	
St. Gallen	Klinik fur Urologie	Claudia Hormann	
St. Gallen	Klinik fur Urologie	Sibylle Schapper	
St.Leonards-on-Sea	Conquest Hospital	Roger Plail	
Stevenage	Lister Hospital	Anna Anosova	
Stevenage	Lister Hospital	David Woolf	
Stevenage	Lister Hospital	Mawuelikem Assoku	
Stevenage	Lister Hospital	Nikhil Oommen	
Stevenage	Lister Hospital	Alkhaldi Ashraf	
Stevenage	Lister Hospital	David Ward	
Stevenage	Lister Hospital	Jonathan Towler	
Stevenage	Lister Hospital	Leena Mukherjee	
Stevenage	Lister Hospital	Robert Hughes	PI
Stevenage	Lister Hospital	Steven Watkins	
Stevenage	Lister Hospital	Natalie Rahim	
Stevenage	Lister Hospital	Sayyida Nembhard	

Stevenage	Lister Hospital	Martin Ebon	
Stevenage	Lister Hospital	Stephen Almond	
Stevenage	Lister Hospital	Clare Collins	
Stevenage	Lister Hospital	Jemma Gilmore	
Stevenage	Lister Hospital	Sunita Gohil	
Stevenage	Lister Hospital	Sura Dabbagh	Pharmacist
Stevenage	Lister Hospital	Alice Valle	
Stevenage	Lister Hospital	Corinne Bradshaw	
Stevenage	Lister Hospital	Elen Witness	
Stevenage	Lister Hospital	Rhos Gabriel	
Stevenage	Lister Hospital	Anita Rana	Pharmacist
Stevenage	Lister Hospital	Katie Poole	
Stevenage	Lister Hospital	Roisin Schimmel	
Stevenage	Lister Hospital	Vicky Hills	
Stockport	Stepping Hill Hospital	Carmel Anandadas	
Stockport	Stepping Hill Hospital	Satish Venkateshan	
Stockport	Stepping Hill Hospital	Umi Hatimy	
Stockport	Stepping Hill Hospital	Apurna Jegannathen	
Stockport	Stepping Hill Hospital	Catherine Coyle	
Stockport	Stepping Hill Hospital	John Logue	PI
Stockport	Stepping Hill Hospital	Abigail Mackley	
Stockport	Stepping Hill Hospital	Anna Kellingray	
Stockport	Stepping Hill Hospital	Oluwademilade Odewumi	
Stockport	Stepping Hill Hospital	Sarah Smallwood	
Stockport	Stepping Hill Hospital	Aelens Brauckman	

Stockport	Stepping Hill Hospital	Benjamin Ralphs	
Stockport	Stepping Hill Hospital	Donald van Welsenes	
Stockport	Stepping Hill Hospital	John Kilmartin	
Stockport	Stepping Hill Hospital	Jonathan Wong	Pharmacist
Stockport	Stepping Hill Hospital	Paul Berry	
Stockport	Stepping Hill Hospital	Wasim Akhtar	
Stockport	Stepping Hill Hospital	Adebanji Adeyoju	
Stockport	Stepping Hill Hospital	Andrew Sinclair	
Stockport	Stepping Hill Hospital	David Ross	
Stockport	Stepping Hill Hospital	Gerald Collins	
Stockport	Stepping Hill Hospital	Patrick O'Reilly	
Stockport	Stepping Hill Hospital	Richard Brough	
Stockport	Stepping Hill Hospital	Stephen Bromage	
Stockport	Stepping Hill Hospital	Stephen CW Brown	
Stockport	Stepping Hill Hospital	Helen Haydock	
Stockport	Stepping Hill Hospital	Louise Brown	
Stockport	Stepping Hill Hospital	Susan Hopkins	
Stockport	Stepping Hill Hospital	Abigail Pemberton	
Stockport	Stepping Hill Hospital	Alissa Kent	
Stockport	Stepping Hill Hospital	Emma Goodwin	
Stockport	Stepping Hill Hospital	Emma Taylor	
Stockport	Stepping Hill Hospital	Julie Melville	
Stockport	Stepping Hill Hospital	Katrina Wade	
Stockport	Stepping Hill Hospital	Lucy Orrell	Pharmacist
Stockport	Stepping Hill Hospital	Magda Kujawa	

Stockport	Stepping Hill Hospital	Miriam Avery	
Stockport	Stepping Hill Hospital	Mkyla Reilly	
Stockport	Stepping Hill Hospital	Susan Graham	Pharmacist
Stockport	Stepping Hill Hospital	Zoe Jordan	
Stockport	Stepping Hill Hospital	Catherine Fox	Pharmacist
Stockport	Stepping Hill Hospital	Christina Gilmour	
Stockport	Stepping Hill Hospital	Eleanor Anscombe	
Stockport	Stepping Hill Hospital	Emma Hewitt	Pharmacist
Stockport	Stepping Hill Hospital	Jean Cheetham	
Stockport	Stepping Hill Hospital	Jill Taylor	
Stockport	Stepping Hill Hospital	Nicola Hermitage	
Stockport	Stepping Hill Hospital	Pat Clitheroe	
Stockport	Stepping Hill Hospital	Sam Corcoran	
Stockport	Stepping Hill Hospital	Sarah Connolly nee McKenna	
Stockport	Stepping Hill Hospital	Sheila Hodgkinson	
Stockport	Stepping Hill Hospital	Tracie Cocks	
Stockton on Tees	North Tees General Hospital	Devadasan Shakespeare	
Stockton-on-Tees	University Hospital of North Tees	Abdul Mian	
Stockton-on-Tees	University Hospital of North Tees	Darren Leaning	PI
Stockton-on-Tees	University Hospital of North Tees	Jenny Smith	
Stockton-on-Tees	University Hospital of North Tees	Devadasan Shakespeare	
Stockton-on-Tees	University Hospital of North Tees	Gaurav Kumar	
Stockton-on-Tees	University Hospital of North Tees	Gala Stancev Stevanovic	
Stockton-on-Tees	University Hospital of North Tees	Helen Wardle (nee Wilson)	
Stockton-on-Tees	University Hospital of North Tees	Moira Percival	

Stockton-on-Tees	University Hospital of North Tees	Andrew Sigsworth	
Stockton-on-Tees	University Hospital of North Tees	Bill Wetherill	Pharmacist
Stockton-on-Tees	University Hospital of North Tees	Victor Palit	
Stockton-on-Tees	University Hospital of North Tees	Hyder Latif	
Stockton-on-Tees	University Hospital of North Tees	Helen Dunn (nee Carey)	Pharmacist
Stockton-on-Tees	University Hospital of North Tees	Jeanette Naisbitt	
Stockton-on-Tees	University Hospital of North Tees	Pam Race	
Stockton-on-Tees	University Hospital of North Tees	Sarah Pitcairn	
Stockton-on-Tees	University Hospital of North Tees	Alison Chilvers	
Stockton-on-Tees	University Hospital of North Tees	Leigh Pollard	
Stockton-on-Tees	University Hospital of North Tees	Emma Jameson	
Stockton-on-Tees	University Hospital of North Tees	Lynda Poole	
Stoke-on-Trent	Royal Stoke University Hospital	Sumera Butt	
Stoke-on-Trent	Royal Stoke University Hospital	Fawzi Adab	
Stoke-on-Trent	Royal Stoke University Hospital	Rajanee Bhana	
Stoke-on-Trent	Royal Stoke University Hospital	Salil Vengalil	PI
Stoke-on-Trent	Royal Stoke University Hospital	Elizabeth Sellars	
Stoke-on-Trent	Royal Stoke University Hospital	Emma Jackson	
Stoke-on-Trent	Royal Stoke University Hospital	Christopher Luscombe	
Stoke-on-Trent	Royal Stoke University Hospital	Robert Green	
Stoke-on-Trent	Royal Stoke University Hospital	Liberty Verueco	
Stoke-on-Trent	Royal Stoke University Hospital	Sharon Rollison	
Stoke-on-Trent	Royal Stoke University Hospital	Alison Tute	Pharmacist
Stoke-on-Trent	Royal Stoke University Hospital	Angela Peake	
Stoke-on-Trent	Royal Stoke University Hospital	Angela Ward	

Stoke-on-Trent	Royal Stoke University Hospital	Marion Evans	
Stoke-on-Trent	Royal Stoke University Hospital	Eden Ball	
Stoke-on-Trent	Royal Stoke University Hospital	Alison Myatt	
Stoke-on-Trent	Royal Stoke University Hospital	Elizabeth Williamson	
Stoke-on-Trent	Royal Stoke University Hospital	Grace Gough	
Stoke-on-Trent	Royal Stoke University Hospital	Isabel Breeze	Pharmacist
Stoke-on-Trent	Royal Stoke University Hospital	Julie Storer	
Stoke-on-Trent	Royal Stoke University Hospital	Katrina Parkinson	
Stoke-on-Trent	Royal Stoke University Hospital	Rowena Smith	
Sunderland	Sunderland Royal Hospital	Rachel Pearson	Co-I
Sunderland	Sunderland Royal Hospital	Shahid Iqbal	
Sunderland	Sunderland Royal Hospital	Ashraf Azzabi	PI
Sunderland	Sunderland Royal Hospital	Ian Pedley	Co-I
Sunderland	Sunderland Royal Hospital	Kathryn Wright	Co-I
Sunderland	Sunderland Royal Hospital	Stephen Laybourne	
Sunderland	Sunderland Royal Hospital	Rod Beard	Pharmacist
Sunderland	Sunderland Royal Hospital	Stephen Butler	
Sunderland	Sunderland Royal Hospital	Amanda Howey	
Sunderland	Sunderland Royal Hospital	Fiona Wakinshaw	
Sunderland	Sunderland Royal Hospital	Jane Cole	
Sunderland	Sunderland Royal Hospital	Paula Newton	
Sunderland	Sunderland Royal Hospital	Terri Haldane	
Sunderland	Sunderland Royal Hospital	Christine Harle	
Sunderland	Sunderland Royal Hospital	Fiona Wakinshaw	
Sunderland	Sunderland Royal Hospital	Julia Scott	

Sunderland	Sunderland Royal Hospital	Karen Shield	Pharmacist
Sunderland	Sunderland Royal Hospital	Michelle Edwards	Pharmacist
Sunderland	Sunderland Royal Hospital	Vivienne Hullock	
Sutton	Royal Marsden Hospital (Sutton)	Adham Hijab	
Sutton	Royal Marsden Hospital (Sutton)	Alex Tan	
Sutton	Royal Marsden Hospital (Sutton)	Angela Pathmanathan	Co-I
Sutton	Royal Marsden Hospital (Sutton)	Gerard McVey	
Sutton	Royal Marsden Hospital (Sutton)	Nora Sundahl	Co-I
Sutton	Royal Marsden Hospital (Sutton)	Susan Lalondrelle	
Sutton	Royal Marsden Hospital (Sutton)	Alison Tree	
Sutton	Royal Marsden Hospital (Sutton)	Chris Parker	PI
Sutton	Royal Marsden Hospital (Sutton)	Ray Shepherd	
Sutton	Royal Marsden Hospital (Sutton)	Robert Huddart	
Sutton	Royal Marsden Hospital (Sutton)	Rosalind Eeles	
Sutton	Royal Marsden Hospital (Sutton)	Vincent Khoo	
Sutton	Royal Marsden Hospital (Sutton)	Lucy Featherstone	Pharmacist
Sutton	Royal Marsden Hospital (Sutton)	Victoria Sjolin	Pharmacist
Sutton	Royal Marsden Hospital (Sutton)	Martha Bullimore	
Sutton	Royal Marsden Hospital (Sutton)	Alexander Macnab	
Sutton	Royal Marsden Hospital (Sutton)	Amir El Ghazal	
Sutton	Royal Marsden Hospital (Sutton)	Douglas Brand	Co-I
Sutton	Royal Marsden Hospital (Sutton)	Nick Hunnings	Pharmacist
Sutton	Royal Marsden Hospital (Sutton)	Tiaan Jacobs	
Sutton	Royal Marsden Hospital (Sutton)	Ruth Woode-Amissah	
Sutton	Royal Marsden Hospital (Sutton)	Zaynah Gurreebun	

Sutton	Royal Marsden Hospital (Sutton)	Sally Moore	
Sutton	Royal Marsden Hospital (Sutton)	Annette Musallam	
Sutton	Royal Marsden Hospital (Sutton)	Claire Crowley	
Sutton	Royal Marsden Hospital (Sutton)	Helen Stidwell	
Sutton	Royal Marsden Hospital (Sutton)	Janine Flohr	
Sutton	Royal Marsden Hospital (Sutton)	Jenni Parmar	
Sutton	Royal Marsden Hospital (Sutton)	Kelly Jones	
Sutton	Royal Marsden Hospital (Sutton)	Kirsty Cuthbertson	
Sutton	Royal Marsden Hospital (Sutton)	Laura Hennelly	
Sutton	Royal Marsden Hospital (Sutton)	Rookmeen Alighan	
Sutton	Royal Marsden Hospital (Sutton)	Bernadette Johnson	
Sutton	Royal Marsden Hospital (Sutton)	Chloe McCormack	
Sutton	Royal Marsden Hospital (Sutton)	Fatima Ahmed	
Sutton	Royal Marsden Hospital (Sutton)	Sue Cromarty	Pharmacist
Sutton	Royal Marsden Hospital (Sutton)	Alan Horwich	
Sutton	Royal Marsden Hospital (Sutton)	David Dearnaley	
Sutton Coldfield	Good Hope Hospital	Mark O'Beirn	Co-I
Sutton Coldfield	Good Hope Hospital	Daniel Ford	PI
Sutton Coldfield	Good Hope Hospital	Lorna Swaddle	
Sutton Coldfield	Good Hope Hospital	Steve Hay	
Sutton Coldfield	Good Hope Hospital	Chen Bartlett	
Sutton Coldfield	Good Hope Hospital	James Whitehouse	
Sutton Coldfield	Good Hope Hospital	Helen Thomas	
Sutton Coldfield	Good Hope Hospital	Ellen Drew	
Sutton Coldfield	Good Hope Hospital	Ann Schumacher	

Sutton Coldfield	Good Hope Hospital	Arlene Oldan	
Sutton Coldfield	Good Hope Hospital	Beena Mistry	
Sutton Coldfield	Good Hope Hospital	Helen Taylor	
Sutton Coldfield	Good Hope Hospital	Janet Prentice	
Sutton Coldfield	Good Hope Hospital	Kamaldeep Ajimal	
Sutton Coldfield	Good Hope Hospital	Rachael O'Beney	
Sutton Coldfield	Good Hope Hospital	Sarah Rogers	
Sutton Coldfield	Good Hope Hospital	Shobit Baijal	
Sutton Coldfield	Good Hope Hospital	Sundip Sohanpal	
Sutton Coldfield	Good Hope Hospital	Alison Maidment	Pharmacist
Sutton Coldfield	Good Hope Hospital	Katy Moore	Pharmacist
Sutton Coldfield	Good Hope Hospital	Lubna Khan	
Sutton-in-Ashfield	King's Mill Hospital	Andrew Brocklehurst	
Sutton-in-Ashfield	King's Mill Hospital	Elena Macleod	
Sutton-in-Ashfield	King's Mill Hospital	Fiona Smith	
Sutton-in-Ashfield	King's Mill Hospital	James Price	
Sutton-in-Ashfield	King's Mill Hospital	Jun Lim	
Sutton-in-Ashfield	King's Mill Hospital	Lauren Jones	
Sutton-in-Ashfield	King's Mill Hospital	Michael Ocathail	
Sutton-in-Ashfield	King's Mill Hospital	Muhammad Gill	
Sutton-in-Ashfield	King's Mill Hospital	Robert Goldspring	
Sutton-in-Ashfield	King's Mill Hospital	Sadia Abdullah	
Sutton-in-Ashfield	King's Mill Hospital	Sarah Taylor	
Sutton-in-Ashfield	King's Mill Hospital	Wai Hou Sam	
Sutton-in-Ashfield	King's Mill Hospital	Benjamin Masters	Co-I

Sutton-in-Ashfield	King's Mill Hospital	Daniel Saunders	PI
Sutton-in-Ashfield	King's Mill Hospital	Eliot Chadwick	
Sutton-in-Ashfield	King's Mill Hospital	Georgina Walker	PI
Sutton-in-Ashfield	King's Mill Hospital	Louise Brookes	Co-I
Sutton-in-Ashfield	King's Mill Hospital	Andrea Palfreman	
Sutton-in-Ashfield	King's Mill Hospital	Jamie-Rae Burgoyne	
Sutton-in-Ashfield	King's Mill Hospital	Lisa Rahn	
Sutton-in-Ashfield	King's Mill Hospital	Victoria Moore	
Sutton-in-Ashfield	King's Mill Hospital	Shila Hamzepur	
Sutton-in-Ashfield	King's Mill Hospital	Terri-Ann Sewell	
Sutton-in-Ashfield	King's Mill Hospital	Wayne Lovegrove	
Sutton-in-Ashfield	King's Mill Hospital	Dominic Nash	
Sutton-in-Ashfield	King's Mill Hospital	Steve Haigh	Pharmacist
Sutton-in-Ashfield	King's Mill Hospital	Inez Wynter	
Sutton-in-Ashfield	King's Mill Hospital	Katie Slack	
Sutton-in-Ashfield	King's Mill Hospital	Sarah Shelton	
Sutton-in-Ashfield	King's Mill Hospital	Margaret Wheatley	
Sutton-in-Ashfield	King's Mill Hospital	Samantha Boam	
Sutton-in-Ashfield	King's Mill Hospital	Samantha March	Pharmacist
Sutton-in-Ashfield	King's Mill Hospital	Linda Otter	Pharmacist
Sutton-in-Ashfield	King's Mill Hospital	Lynne Wade	
Sutton-in-Ashfield	King's Mill Hospital	Rebecca Holmes	
Sutton-in-Ashfield	King's Mill Hospital	Susan Smith	
Swansea	Singleton Hospital	Ahmed Shaheen	PI
Swansea	Singleton Hospital	Aijaz Lone	Co-I

Swansea	Singleton Hospital	David Brown	
Swansea	Singleton Hospital	Delia Pudney	
Swansea	Singleton Hospital	Fiona Williams	Co-I
Swansea	Singleton Hospital	Helen Fitzgerald	Co-I
Swansea	Singleton Hospital	N Sindgi	
Swansea	Singleton Hospital	Nia Jackson	Co-I
Swansea	Singleton Hospital	Rhian Davies	Co-I
Swansea	Singleton Hospital	Ricky Fraser	
Swansea	Singleton Hospital	Russell Banner	
Swansea	Singleton Hospital	Sarah Gwynne	
Swansea	Singleton Hospital	Satish Kumar	
Swansea	Singleton Hospital	Sharath Gangadhara	
Swansea	Singleton Hospital	Sheena Lam	Co-I
Swansea	Singleton Hospital	Wael Mohamed	PI
Swansea	Singleton Hospital	Gianfilippo Bertelli	
Swansea	Singleton Hospital	Jason Lester	
Swansea	Singleton Hospital	Mau-Don Phan	Co-I
Swansea	Singleton Hospital	Nicola Davies	
Swansea	Singleton Hospital	Angharad Phillips	
Swansea	Singleton Hospital	Anne Thomas	Pharmacist
Swansea	Singleton Hospital	Bethan Williams	
Swansea	Singleton Hospital	Ellen Tait	
Swansea	Singleton Hospital	Karen Chesters	
Swansea	Singleton Hospital	Katie Tanner	
Swansea	Singleton Hospital	Naomi Woods	

Swansea	Singleton Hospital	Nicola Lemon	
Swansea	Singleton Hospital	Alex Richards	
Swansea	Singleton Hospital	Carl Ackland	
Swansea	Singleton Hospital	James Morgan	
Swansea	Singleton Hospital	Lewis Jones	
Swansea	Singleton Hospital	Mark Rogers	
Swansea	Singleton Hospital	Stuart Evans	Pharmacist
Swansea	Singleton Hospital	Ashok Kumar	
Swansea	Singleton Hospital	Brian Phillips	
Swansea	Singleton Hospital	Euan Pratt	
Swansea	Singleton Hospital	Amanda Cook	
Swansea	Singleton Hospital	Charlotte Young	
Swansea	Singleton Hospital	Dawn Withers	
Swansea	Singleton Hospital	Gillian Palmer	
Swansea	Singleton Hospital	Gillian Willetts	
Swansea	Singleton Hospital	Helen Cheley	
Swansea	Singleton Hospital	Lesley Richards	
Swansea	Singleton Hospital	Lynne Breeze-Jones	
Swansea	Singleton Hospital	Alex Franklin	
Swansea	Singleton Hospital	Gillian Jones	Pharmacist
Swansea	Singleton Hospital	Karen Phillips	
Swansea	Singleton Hospital	Mair Roberts	Pharmacist
Swansea	Singleton Hospital	Amy Quinton	
Swansea	Singleton Hospital	Chelsea Jenkins	
Swansea	Singleton Hospital	Elizabeth Evans	

Swansea	Singleton Hospital	Emily Harris (n. Marchant)	
Swansea	Singleton Hospital	Emma Dangerfield	
Swansea	Singleton Hospital	Judith Gooding	Pharmacist
Swansea	Singleton Hospital	Maria Johnstone	
Swansea	Singleton Hospital	Michelle Romano	
Swansea	Singleton Hospital	Renata Poole	Pharmacist
Swansea	Singleton Hospital	Alison Stretch	
Swansea	Singleton Hospital	Amanda Jackson	
Swansea	Singleton Hospital	Donna Lear	
Swansea	Singleton Hospital	Gail Povey	
Swansea	Singleton Hospital	Jayne Caparros	
Swansea	Singleton Hospital	Leanne Quinn	
Swansea	Singleton Hospital	Lisa Ellis	
Swansea	Singleton Hospital	Lorraine Gammon	
Swansea	Singleton Hospital	Nia Viney	
Swansea	Singleton Hospital	Susie Pitcher	
Swansea	Singleton Hospital	Tracey Ford	
Swansea	Singleton Hospital	John Wagstaff	
Swindon	Great Western Hospital	Dorota Marciniak	
Swindon	Great Western Hospital	Raj Jampana	
Swindon	Great Western Hospital	Aruna Medisetti	
Swindon	Great Western Hospital	David J Cole	
Swindon	Great Western Hospital	Dorothe Maramak	
Swindon	Great Western Hospital	Esme Hill	
Swindon	Great Western Hospital	Gerard Andrade	

Swindon	Great Western Hospital	Omar Khan	PI
Swindon	Great Western Hospital	Shiroma De Silva-Minor	
Swindon	Great Western Hospital	Victoria Gibson	
Swindon	Great Western Hospital	Emma Wakefield	Pharmacist
Swindon	Great Western Hospital	Graham Brown	
Swindon	Great Western Hospital	Joseph Stevens	
Swindon	Great Western Hospital	Ronak Patel	
Swindon	Great Western Hospital	Tim Owen	
Swindon	Great Western Hospital	Christopher Clarke	
Swindon	Great Western Hospital	David Newell	
Swindon	Great Western Hospital	Jonathan Lewis	
Swindon	Great Western Hospital	Mike Lewis	
Swindon	Great Western Hospital	Vivian Zinyemba	
Swindon	Great Western Hospital	Chanelle Meyer	
Swindon	Great Western Hospital	Karen Smith	
Swindon	Great Western Hospital	Rebecca Belcher	
Swindon	Great Western Hospital	Sarah Cotton	
Swindon	Great Western Hospital	Sue Meakin	
Swindon	Great Western Hospital	Amanda Colston	
Swindon	Great Western Hospital	Caroline Pensotti	
Swindon	Great Western Hospital	Debbie Palmer	
Swindon	Great Western Hospital	Jean Kordula	
Swindon	Great Western Hospital	Laura McCafferty	
Swindon	Great Western Hospital	Lesley Haxton	
Swindon	Great Western Hospital	Sarah Grayland	

Swindon	Great Western Hospital	Sarah Long	
Swindon	Great Western Hospital	Catherine Lewis Clarke	
Swindon	Great Western Hospital	Cerila Parajes	
Swindon	Great Western Hospital	Deborah Scott	
Swindon	Great Western Hospital	Donna Lake	
Swindon	Great Western Hospital	Fahad Fazal	
Swindon	Great Western Hospital	Rachel Messenger	
Swindon	Great Western Hospital	Suzannah Pegler	
Swindon	Great Western Hospital	Aiste Baltramaityte	
Swindon	Great Western Hospital	Ania Jones	
Swindon	Great Western Hospital	Ellen Starling	
Swindon	Great Western Hospital	Ellie Hewitt	
Swindon	Great Western Hospital	Nicola Cowling	
Swindon	Great Western Hospital	Sally-Ann Parkin (nee) Lee	
Swindon	Great Western Hospital	Tracey Sargent	
Swindon	Great Western Hospital	Helen Winter	
Swindon	Great Western Hospital	Jan Dodge	
Swindon	Great Western Hospital	Tracey Sargent	
Taunton	Musgrove Park Hospital	Clair Brunner	
Taunton	Musgrove Park Hospital	Elena Macleod	
Taunton	Musgrove Park Hospital	Hannah Berry	
Taunton	Musgrove Park Hospital	Joanne Botten	
Taunton	Musgrove Park Hospital	Manivannan Periasamy	
Taunton	Musgrove Park Hospital	Mohini Varughese	Co-I
Taunton	Musgrove Park Hospital	Nicola Cox	Co-I

Taunton	Musgrove Park Hospital	Olivia Fraser	
Taunton	Musgrove Park Hospital	Darren Brady	
Taunton	Musgrove Park Hospital	Emma Gray	PI
Taunton	Musgrove Park Hospital	George Plataniotis	
Taunton	Musgrove Park Hospital	Gihan Ratnayake	
Taunton	Musgrove Park Hospital	John Graham	PI
Taunton	Musgrove Park Hospital	Joseph Jelski	
Taunton	Musgrove Park Hospital	Manjusha Keni	
Taunton	Musgrove Park Hospital	Mary Tighe	
Taunton	Musgrove Park Hospital	Rebecca Denslow	Co-I
Taunton	Musgrove Park Hospital	Robert Zorica	
Taunton	Musgrove Park Hospital	Abby Farzaneh	
Taunton	Musgrove Park Hospital	Amy Sawyer	
Taunton	Musgrove Park Hospital	Christina Branfield	
Taunton	Musgrove Park Hospital	Corinne Pawley	
Taunton	Musgrove Park Hospital	Hayley Cornall	
Taunton	Musgrove Park Hospital	Jasmine Youens	
Taunton	Musgrove Park Hospital	Odunayo Kalejaiye	
Taunton	Musgrove Park Hospital	Rachel Coe	
Taunton	Musgrove Park Hospital	Rebecca Brown	
Taunton	Musgrove Park Hospital	Rebecca Twemlow	
Taunton	Musgrove Park Hospital	Guillermo Reina-Ruiz	
Taunton	Musgrove Park Hospital	John Allinson-Smith	
Taunton	Musgrove Park Hospital	Joshua Woollven	
Taunton	Musgrove Park Hospital	Lee Talbot	

Taunton	Musgrove Park Hospital	Luke Stephens	Pharmacist
Taunton	Musgrove Park Hospital	Tamlyn Russell	
Taunton	Musgrove Park Hospital	Ian Bodger	
Taunton	Musgrove Park Hospital	Jarrod Dunn	Pharmacist
Taunton	Taunton and Somerset Hospital	Jarrod Dunn	Pharmacist
Taunton	Musgrove Park Hospital	Richard Burgess	
Taunton	Musgrove Park Hospital	Ruaraidh MacDonagh	
Taunton	Taunton and Somerset Hospital	Ruaraidh MacDonagh	
Taunton	Musgrove Park Hospital	Simon Goldsworthy	
Taunton	Musgrove Park Hospital	Alison Snell	
Taunton	Musgrove Park Hospital	Amanda Groves	
Taunton	Musgrove Park Hospital	Ceri Poyntz-wright	
Taunton	Musgrove Park Hospital	Clair Hinton	
Taunton	Musgrove Park Hospital	Claire Sowerby	
Taunton	Musgrove Park Hospital	Fiona Goodchild	
Taunton	Musgrove Park Hospital	Francesca Allen	
Taunton	Musgrove Park Hospital	Joanne Rogers	
Taunton	Musgrove Park Hospital	Joanne Taylor	
Taunton	Musgrove Park Hospital	Lynn Leat	
Taunton	Musgrove Park Hospital	Sue Mahoney	
Taunton	Musgrove Park Hospital	Susan Crouch	
Taunton	Musgrove Park Hospital	Michelle Farrar	
Taunton	Musgrove Park Hospital	Alison Whitcher	
Taunton	Musgrove Park Hospital	Anna Masamba	
Taunton	Musgrove Park Hospital	Catherine Lane	

Taunton	Musgrove Park Hospital	Christine Webster	
Taunton	Musgrove Park Hospital	Fen Lewen	
Taunton	Musgrove Park Hospital	Joan Kemp	Pharmacist
Taunton	Musgrove Park Hospital	Karen Tanner	
Taunton	Musgrove Park Hospital	Martha Wrigley	
Taunton	Musgrove Park Hospital	Moira Tait	
Taunton	Musgrove Park Hospital	Nicola Cutmore	
Taunton	Musgrove Park Hospital	Rebecca Purnell	
Taunton	Musgrove Park Hospital	Samantha Northover	
Taunton	Musgrove Park Hospital	Sara Green	
Taunton	Musgrove Park Hospital	Sara Myers	
Taunton	Musgrove Park Hospital	Alison Chedham	
Taunton	Musgrove Park Hospital	Angela Locke	
Taunton	Musgrove Park Hospital	Jan Ashcroft	
Taunton	Taunton and Somerset Hospital	Jan Ashcroft	
Taunton	Musgrove Park Hospital	Joy Rowe	Pharmacist
Taunton	Musgrove Park Hospital	Judith Mathie	
Taunton	Taunton and Somerset Hospital	Judith Mathie	
Taunton	Musgrove Park Hospital	Lisa Bowern	
Taunton	Musgrove Park Hospital	Lucy Howell-Drewett	
Taunton	Musgrove Park Hospital	Nita Beacham	
Taunton	Musgrove Park Hospital	Rebecca Tucker	
Taunton	Taunton and Somerset Hospital	Rebecca Tucker	
Taunton	Musgrove Park Hospital	Rebecca Wallbutton	
Tessin	Istituto Oncologico della Svizzera Italiana	Anna Llado	

Tessin	Istituto Oncologico della Svizzera Italiana	Enrico Roggero	PI
Tessin	Istituto Oncologico della Svizzera Italiana	Barbara Marongiu	
Tessin	Istituto Oncologico della Svizzera Italiana	Michele Moro	
Tessin	Istituto Oncologico della Svizzera Italiana	Sabine Van Den Bosch	
Thornton Heath	Croydon University Hospital	Jane Thomson	
Torquay	Torbay District General Hospital	Helen Saxby	
Torquay	Torbay District General Hospital	Victoria Bell	
Torquay	Torbay District General Hospital	Victoria Cope	
Torquay	Torbay District General Hospital	Anna Lydon	PI
Torquay	Torbay District General Hospital	Beverley Watkins	
Torquay	Torbay District General Hospital	Erica Watts	
Torquay	Torbay District General Hospital	Fiona Roberts	Co-I
Torquay	Torbay District General Hospital	Jorg Michels	
Torquay	Torbay District General Hospital	Rajaguru Srinivasan	
Torquay	Torbay District General Hospital	Emily Flavell	
Torquay	Torbay District General Hospital	Helen Kimber	
Torquay	Torbay District General Hospital	Kirsty Jones	
Torquay	Torbay District General Hospital	Kirsty Pearce	
Torquay	Torbay District General Hospital	Sally Wells	Pharmacist
Torquay	Torbay District General Hospital	Sophie Gittus	
Torquay	Torbay District General Hospital	Stacey Davies	
Torquay	Torbay District General Hospital	Linda Welsh	
Torquay	Torbay District General Hospital	Kenneth Almedilla	
Torquay	Torbay District General Hospital	Lee Merry	
Torquay	Torbay District General Hospital	Peter Fletcher	

Torquay	Torbay District General Hospital	Peter Pugh	
Torquay	Torbay District General Hospital	Seamus McDermott	
Torquay	Torbay District General Hospital	Andrew Harford-Brown	
Torquay	Torbay District General Hospital	Magdi Kirollos	
Torquay	Torbay District General Hospital	Mark Brennan	
Torquay	Torbay District General Hospital	Martyn Blundell	Pharmacist
Torquay	Torbay District General Hospital	Robert Mason	
Torquay	Torbay District General Hospital	Angela Foulds	
Torquay	Torbay District General Hospital	Kirsty Lester	
Torquay	Torbay District General Hospital	Lauren Blunt	
Torquay	Torbay District General Hospital	Michele Allison	
Torquay	Torbay District General Hospital	Shelley Chamberlain	
Torquay	Torbay District General Hospital	Tyler Lowe	
Torquay	Torbay District General Hospital	Christine Rawlings	
Torquay	Torbay District General Hospital	Donna Cuffe	
Torquay	Torbay District General Hospital	Elaine Vandecandalaere	
Torquay	Torbay District General Hospital	Amy Millington	
Torquay	Torbay District General Hospital	Catherine Brookman	
Torquay	Torbay District General Hospital	Catherine Marshall	
Torquay	Torbay District General Hospital	Classy Lam	
Torquay	Torbay District General Hospital	Emmie Arbury	
Torquay	Torbay District General Hospital	Fleur Rogers	
Torquay	Torbay District General Hospital	Janet Palmer	
Torquay	Torbay District General Hospital	Petra Gee	
Torquay	Torbay District General Hospital	Sally Maddison	

Torquay	Torbay District General Hospital	Sarah Rees	
Torquay	Torbay District General Hospital	Sophie Norman	
Torquay	Torbay District General Hospital	Sue Forbes	
Torquay	Torbay District General Hospital	Amanda Vian	
Torquay	Torbay District General Hospital	Helen Greedus	
Torquay	Torbay District General Hospital	Ingrid Koehler	
Torquay	Torbay District General Hospital	Jo Blurton	
TORQUAY	Torbay District General Hospital	Lorraine Thornton	
Torquay	Torbay District General Hospital	Louise Paatz	Pharmacist
Torquay	Torbay District General Hospital	Lyn Micklewright	
Torquay	Torbay District General Hospital	Melody Cross	
Torquay	Torbay District General Hospital	Sarah Wright	
Torquay	Torbay District General Hospital	Victoria Bell	

INDUSTRY COLLABORATORS

Clovis Oncology
Support for the STAMPEDE trial has been provided by Clovis Oncology.

Janssen
Laurent Antoni
Joaquin Casariego garcia luben
Ilde Herrygars
Florence Lefresne
Rod Murphy
Mohamed Samir
Hind Stitou
Support for the STAMPEDE trial has been provided by Janssen.

Novartis
Support for the STAMPEDE trial has been provided by Novartis Pharmaceuticals UK Limited.

Sanofi-Aventis
Paul Cadle
Christine Geffriaud-Ricouard
Zsuzsanna Devecseri
Support for the STAMPEDE study has been provided by Sanofi-Aventis.

Pfizer
Pam Brambles
Support for the STAMPEDE study has been provided Pfizer

Astellas
John Galfin
Karla Martins
Gunther Boysen
Padraig Moran
Support for the STAMPEDE study has been provided by astellas
